# Epigenetic Features of Human Mesenchymal Stem Cells Determine Their Permissiveness for Induction of Relevant Transcriptional Changes by SYT-SSX1

**DOI:** 10.1371/journal.pone.0007904

**Published:** 2009-11-19

**Authors:** Luisa Cironi, Paolo Provero, Nicola Riggi, Michalina Janiszewska, Domizio Suva, Mario-Luca Suva, Vincent Kindler, Ivan Stamenkovic

**Affiliations:** Division of Experimental Pathology, Institute of Pathology, Centre Hospitalier Universitaire Vaudois, Faculty of Biology and Medicine, University of Lausanne, Lausanne, Switzerland; Universität Heidelberg, Germany

## Abstract

**Background:**

A characteristic SYT**–**SSX fusion gene resulting from the chromosomal translocation t(X;18)(p11;q11) is detectable in almost all synovial sarcomas, a malignant soft tissue tumor widely believed to originate from as yet unidentified pluripotent stem cells. The resulting fusion protein has no DNA binding motifs but possesses protein-protein interaction domains that are believed to mediate association with chromatin remodeling complexes. Despite recent advances in the identification of molecules that interact with SYT-SSX and with the corresponding wild type SYT and SSX proteins, the mechanisms whereby the SYT-SSX might contribute to neoplastic transformation remain unclear. Epigenetic deregulation has been suggested to be one possible mechanism.

**Methodology/Principal Findings:**

We addressed the effect of SYT/SSX expression on the transcriptome of four independent isolates of primary human bone marrow mesenchymal stem cells (hMSC). We observed transcriptional changes similar to the gene expression signature of synovial sarcoma, principally involving genes whose regulation is linked to epigenetic factors, including imprinted genes, genes with transcription start sites within a CpG island and chromatin related genes. Single population analysis revealed hMSC isolate-specific transcriptional changes involving genes that are important for biological functions of stem cells as well as genes that are considered to be molecular markers of synovial sarcoma including *IGF2*, *EPHRINS*, and *BCL2*. Methylation status analysis of sequences at the *H19*/*IGF2* imprinted locus indicated that distinct epigenetic features characterize hMSC populations and condition the transcriptional effects of SYT-SSX expression.

**Conclusions/Significance:**

Our observations suggest that epigenetic features may define the cellular microenvironment in which SYT-SSX displays its functional effects.

## Introduction

Synovial sarcoma (SS) is an aggressive soft tissue tumor that accounts for about 10% of all human sarcomas [1]–[3] and is found throughout the body. It arises in adolescents and young adults and is associated with poor prognosis despite multimodal therapy. Current opinion holds that sarcomas, including synovial sarcoma, are derived from as yet unidentified multipotent stem cells capable of mesenchymal and neuroectodermal differentiation.

More than 90% of synovial sarcomas are characterized by a specific chromosomal translocation, t(X:18)(p11.2: q11.2), that results in the fusion of the *SYT* gene on chromosome 18 to one of several *SSX* gene family members, (*SSX1*, *SSX2* or *SSX4*), on chromosome X.

The *SYT* and the *SSX* gene family encode nuclear proteins whose function has yet to be fully defined. Neither protein has DNA binding motifs but both possess protein-protein interaction domains that are believed to mediate binding to transcriptional regulators. When targeted to a reporter gene, SYT is shown to transactivate, whereas SSX is observed to repress transcription [4], [5].

SYT is a ubiquitously expressed 387 amino acid protein that colocalizes and interacts *in vitro* through its evolutionarily conserved N-terminal SNH (for SYT N-terminal Homology) domain, with human BRM and BRG1, two mutually exclusive ATPases that constitute part of the SWI/SNF complex, a global chromatin remodeling transcriptional coactivator [6]–[8]. In contrast to SYT, SSX proteins have a more restricted distribution, being expressed primarily in the testis. Their C-terminal SSX-RD domain is responsible for their nuclear localization, and for colocalization with RING1 and Bmi-1, components of histone-associated polycomb group proteins implicated in transcriptional repressor activity [5], [9].

Upon translocation, the most common fusion is generated by the replacement of the 8 C-terminal amino acids of *SYT* by the 78 C-terminal amino acids of *SSX*1, 2 or 4. With the exception of one SH2 C-terminal motif all SYT domains are retained in the SYT-SSX fusion, along with the SSX-RD domain [10]. SYT, SSX and the fusion protein appear to have distinct nuclear staining patterns, but consistent with the presence of an intact SNH and SSX-RD domain, SYT/SSX retains interaction with BRM and BRG1 as well as colocalization with the polycomb group proteins [4], [6], [8], [11], [12]. Thus, the SYT-SSX fusion encodes two distinct protein domains, which associate with chromatin remodeling complexes that display opposing functions.

SYT-SSX proteins have been found to display limited oncogenic potential in fibroblasts and various cell lines [13]. These observations suggest either that additional oncogenic events are required for malignant transformation or that SYT-SSX can display its oncogenic potential only in a specific cellular context. Generation of the first transgenic model of SS is consistent with the latter possibility [14]. Conditional expression of SYT-SSX2 in myoblast precursors but not in myocytes resulted in tumors that recapitulate many of the characteristics of human SS. These studies highlight the importance of the timing of fusion transcript expression during cell differentiation and the highly restricted cellular context that is permissive for its oncogenic properties.

Despite discoveries that SYT and SSX associate with other nuclear proteins [2], [15]–[21], it remains unclear how SYT/SSX might contribute to neoplastic transformation. Gene expression profile comparison of SS to other sarcoma types has helped identify a handful of genes that are preferentially associated with SS [22]–[24] and suggests the implication of several signaling pathways in SS pathogenesis, including receptor tyrosine kinases, Hedgehog, Notch, RAR, TGFβ and Wnt [25]–[29]. SYT/SSX has been proposed to regulate cyclin D expression in SS cells [30], [31], to induce the cyclin-dependent kinase inhibitor (CKI) p21(WAF1/CIP1) in various cell lines [32] and to negatively regulate, at least in osteosarcoma cell lines, the stability of the tumor suppressor p53 by promoting HDM2 stabilization [33]. SYT-SSX2 has been proposed to contribute to tumor development through β-catenin signaling [34] and by altering the cytoskeletal architecture in an ephrin-dependent manner [35].

Although molecular characterization of SS and the role of SYT-SSX are beginning to provide insight into events that may be important in shaping the biological behavior of the tumor, numerous questions remain, including whether or not SYT-SSX expression is sufficient for tumor formation and/or differentiation, the nature of the downstream targets of SYT-SSX, and what additional genes might be critical for the genesis of SS. Recent studies have highlighted epigenetic mechanisms as the potential basis for the effects induced by the expression of SYT-SSX [27]. The *H19/IGF2* gene pair, which is the best characterized imprinted chromatin barrier locus described to date, has been proposed as a possible SYT/SSX target [36]. Similar to other imprinted clusters, expression of *H19* and *IGF2* is jointly regulated through an imprinting control region (ICR) of approximately 5 Kb in humans, located between the two genes. This region functions by regulating interactions between *H19* and *IGF2* promoters and their shared enhancers, which are located downstream of the *H19* coding sequence and can, in the absence of a chromatin barrier, stimulate transcription of the *IGF2* gene in *cis*. The methylation status of specific conserved sequences regulates the binding of the ubiquitously expressed factor CTCF to the ICR, which functions as an insulator and enhancer blocker. In most adult tissues, binding of CTCF to the unmethylated maternal allele prevents *H19* enhancers from inducing *IGF2* expression, leaving them available to induce *H19*. Methylation of the paternal *H19* ICR abrogates CTCF binding resulting in enhancer stimulation of *IGF2* expression and *H19* silencing. Recent studies using chromosome conformation capture (3C) have shown that long range allele-specific interactions constitute part of the insulation mechanism but our understanding of these interactions is still incomplete. A complex three-dimensional, multiple-loop model organized by the CTCF-ICR complex on the maternal allele has been recently proposed [37]and both intra and inter-chromosomal interactions have been shown to involve the *H19* ICR [38].

Loss of imprinting (LOI) concurrent to hypomethylation at the *H19*/*IGF2* intergenic region has been observed in a limited number of primary synovial sarcomas [39] and SYT-SSX expression has been shown to induce *IGF2* in immortalized MRC5 fibroblasts [39] and HEK 293 cells [27]. In the latter case, *IGF2* induction could be attributed to epigenetic mechanisms: hypermethylation was observed at the *H19/IGF2* intergenic region and enrichment of histone marks associated with active chromatin was observed at the *IGF2* promoter. More recently, epigenetic effects of SYT-SSX on other targets have been reported [40]. Thus, down-regulation of *EGR1* by SYT-SSX expression in HEK 293 cells was shown to occur via histone modifications and recruitment of polycomb proteins to the *EGR1* promoter. Finally the histone deacetylase (HDAC) inhibitor FK228 has been reported to block synovial sarcoma cell growth both *in vitro* and *in vivo* [20].

Most of these studies were conducted using cell lines that may have acquired significant modifications of their epigenetic status both during transformation and during prolonged cell culture. They were therefore unlikely to fully recapitulate the biology of primary *in vivo* tumor development. Thus, despite these potentially relevant insights, it remains unclear whether the epigenetic effects of SYT-SSX are required for tumor development, maintenance and/or other biological properties of synovial sarcoma. The precise mechanism of epigenetic deregulation by the synovial sarcoma fusion protein has yet to be defined as do the epigenetic features that may render primary cells permissive for SYT-SSX functions and potential oncogenic properties.

To address these issues, we introduced the fusion gene into primary human mesenchymal stem cells (hMSC), that may constitute candidate cells of origin of SS, and assessed factors that may influence SYT-SSX-mediated gene expression profile changes.Our results show that the expression of SYT-SSX in hMSCs induces a transcriptional profile that bears significant relatedness to the synovial sarcoma expression signature. In these cells, SYT-SSX primarily affects the expression of genes whose regulation is linked to epigenetic factors, including imprinted genes, genes with transcription start site (TSS) within a CpG island, and chromatin related genes. Our results also highlight the notion that despite uniform morphology and cell surface marker expression, different MSC populations display distinct epigenetic features that appear to influence transcriptional changes induced by SYT-SSX. These observations suggest that the epigenetic status of primary cells may determine the functional effect of SYT-SSX, possibly including its transforming capacity.

## Results

### Expression of *SYT-SSX1* Fusion Protein in Mesenchymal Stem Cells

**Figure 1:**
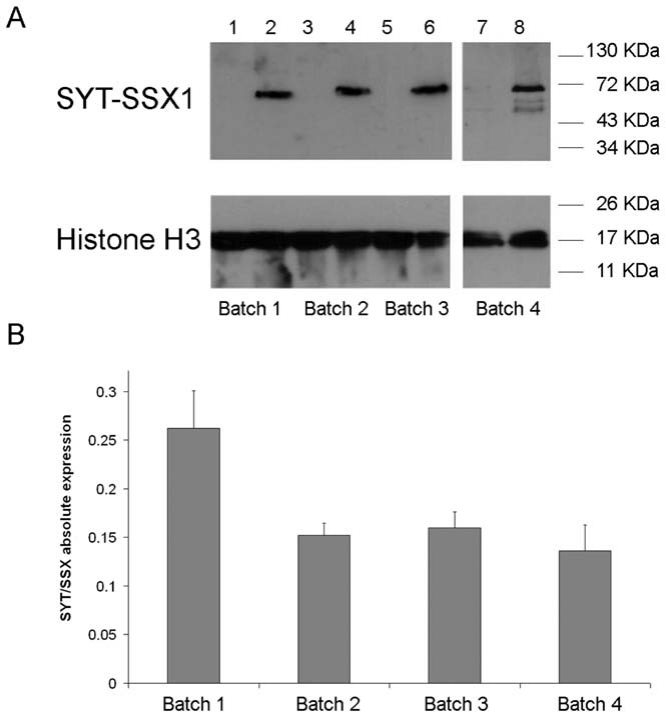
Expression of SYT-SSX1 fusion protein in human MSCs. Four different batches of human MSCs were infected with pMSCV-SYT-SSX1-v5 (lanes 2, 4, 6, 8) or an empty pMSCV neo vector (lanes 1,3, 5, 7), and cells were selected with 0.5 mg/ml G418 and harvested 12 days after infection. (A) Protein expression was assessed by western blot analysis using a mouse anti-v5 monoclonal antibody and HRP-conjugated goat anti mouse IgG. Polyclonal mouse anti-Histone H3 antibody was used as a loading control. Molecular markers are indicated. (B) Total RNA was extracted from the same cells and SYT/SSX1-specific message was measured by real time PCR using primers SYT-SSX1181-1201 F and SYT-SSX1239-1257 R and the universal probelibrary 76. Quantitation of target RNA, normalized with an endogenous control (Cyclophilin), was performed using the absolute quantitation method (Applied Biosystems). Experiments were done in triplicate. Mean values+/−SD are shown.

Human MSCs were obtained from femoral head bone marrow of pediatric and adolescent patients undergoing limb axis re-adjustment. Isolation of hMSCs was performed as previously described [41], [42]. Cells were maintained at low confluence and tested periodically for their ability to differentiate into adipocytes, chondrocytes and osteocytes in response to appropriate cytokines [41], [42]. The four independent hMSC isolates did not display any significant difference in multilineage differentiation tests, a representative result of which is shown in figure S1.The *SYT-SSX1* fusion gene was amplified by RT-PCR from RNA derived from a human synovial sarcoma specimen, ligated in-frame to a V5 tag and introduced into a retroviral expression vector. Four independent hMSC isolates were infected with corresponding retroviruses using a Retroviral Gene Transfer and Expression system. Expression of SYT-SSX1 RNA and protein were tested 12 days after infection and neomycin selection. Real time PCR amplification, using primers complementary to sequences flanking the SYT-SSX breakpoint, indicated similar levels of the corresponding messenger RNA in each population (fig. 1B). Western blot analysis using an anti-V5 tag antibody revealed comparable SYT-SSX1 protein expression among the four infected hMSC populations (fig. 1A). hMSCs expressing SYT-SSX1 (hMSC^SYT-SSX1^) displayed a survival rate comparable to that of wt cells and maintained fusion protein expression for more than 3 months (data not shown). However hMSC^SYT-SSX1^ did not undergo transformation, as assessed by their inability to form tumors upon subcutaneous and sub-capsular renal injection into immunocompromised mice.

### Genes Regulated by SYT-SSX1 Expression in hMSC Are Restricted to a Few Defined Categories

To assess the effect of SYT-SSX expression in hMSC, transcriptome analysis was performed on the 4 independent MSC^SYT-SSX1^ populations 12 days after infection and neomycin selection. The batches were first analyzed together with rank products, resulting in lists of probe sets that were differentially expressed with a false discovery rate (FDR) of 1% and a mean fold change >2 (table S2). Analysis of these lists revealed that many of the genes regulated by SYT-SSX1 expression in hMSCs belong to a restricted set of categories (table 1) that include imprinted genes, genes that encode for chromatin associated proteins, and genes having at least one transcript with a transcription start site (TSS) within a CpG island. Based on these observations, we used several databases to test the over-representation of specific gene categories and perform statistical analysis, including: http://www.geneimprint.com/ and http://igc.otago.ac.nz/home.html [43] for imprinted genes, http://www.chromdb.org/ [44] for chromatin associated protein and http://genome.ucsc.edu/ for genes containing CpG island in the TSS.

**Table 1:** List of genes (gene symbols), induced or repressed in human MSCs by SYT-SSX1 expression, belonging to selected categories or GO terms.

	GENES WITH CpGs IN THE TSS			CHROMATIN ASSOCIATED GENES	IMPRINTED (experimentally confirmed)	IMPRINTED (all)	CENTRAL NERVOUS SYSTEM DEVELOPMENT GO:0007417	NERVOUS SYSTEM DEVELOPMENT GO:0007399
	induced		repressed	induced	Induced	induced	induced	induced
expected number	52.6		18.5	1.6	0.2	0.7	1.16	3.26
observed number	65		13	4	5	10	8	14
P value	0.0051		0.98	0.083	1.60E-06	1.60E-09	2.22E-05	4.29E-06
	*				*	*	*	*
	*ABAT*	*ICAM1*	*ASPM*	*HIST1H2BG*	*CDKN1C*	*CDKN1C*	*HES1*	*GPM6B*
	*APCDD1*	*IGF2*	*C10orf54*	*HIST1H4I*	*DIRAS3*	*COL9A3*	*FGFR3*	*INA*
	*AQP3*	*INA*	*CDC20*	*LEF1*	*DLK1*	*DIRAS3*	*BMP2*	*HES1*
	*BMP2*	*ITGA9*	*CDKN3*	*SOX8*	*H19*	*DLK1*	*SOX8*	*KCNQ2*
	*CABP7*	*KCNJ2*	*COL12A1*		*IGF2*	*H19*	*NES*	*FGFR3*
	*CDKN1C*	*KCNQ2*	*FOXM1*			*HES1*	*LHX2*	*BMP2*
	*CLU*	*L1CAM*	*MOXD1*			*HSPA6*	*LEF1*	*SOX8*
	*COL9A3*	*LEF1*	*PRC1*			*IGF2*	*CXCR4*	*NES*
	*COLEC12*	*LHX2*	*SFRP4*			*SOX8*		*HEYL*
	*CPLX1*	*LIFR*	*TK1*			*SPON2*		*L1CAM*
	*CRABP2*	*LIPG*	*USP53*					*SPON2*
	*CRIP1*	*LONRF3*	*VGLL3*					*LHX2*
	*CRLF1*	*NES*	*ZNF395*					*LEF1*
	*CRTAC1*	*NPTX2*						*CXCR4*
	*CXCR4*	*PDK4*						
	*D4S234E*	*PGF*						
	*DIRAS3*	*PRKAR2B*						
	*DLK1*	*RASD1*						
	*DLL1*	*RHOU*						
	*F2RL1*	*RPESP*						
	*FAM46C*	*S1PR3*						
	*FBXL16*	*SERTAD4*						
	*FGFR3*	*SHISA2*						
	*GABBR2*	*SLC16A6*						
	*GJB2*	*SOX8*						
	*GPM6B*	*SPON2*						
	*H19*	*SULF2*						
	*HBA1*	*TLL2*						
	*HEYL*	*TNFRSF10D*						
	*HOPX*	*TPPP*						
	*HOXD1*	*VAT1L*						
	*HSPA1A*	*ZNF711*						
	*HSPA6*							

Statistical analysis for the over-representation of each category is reported.

Significant P-values (P<0.05) are marked by an asterisk.

Among the genes induced by SYT-SSX1 we found a statistically significant over-representation of transcripts whose TSS lies within a CpG island (P-value 0.0051) and a higher than expected by chance number of chromatin-associated genes, but with a non significant p-value. Analysis of both the geneimprint and otago datasets of imprinted genes revealed highly significant over-representation (p-value as low as 10^−6^) of genes induced by SYT-SSX1. Comparison to geneimprint lists restricted to experimentally confirmed imprinted genes only produced an even higher significance (p value of 10^−9^) than comparison to geneimprint lists inclusive of both experimentally confirmed and predicted imprinted genes. Over-representation of imprinted genes was not observed when the same analysis was performed on the transcriptome of MSCs infected with the Ewing's sarcoma-associated fusion gene EWS-FLI1 (data not shown).

### Analysis of SYT-SSX1-Induced and Repressed Genes According to Functional Annotation

Analysis of genes induced and repressed by SYT-SSX1 in hMSCs, according to Gene Ontology (GO) annotation [45] (http://www.geneontology.org/), revealed over-representation of few GO terms among the induced genes with a P value<10^−5^. Complete lists of GO annotation terms found to be significantly over-represented in each set of induced and repressed genes are available (supplementary data 3). Several of the GO terms, including “extracellular region” and “integral to plasma membrane” encompass a broad range of genes and have limited informative value. However, numerous induced genes were found to be associated with more specific annotations of potential functional relevance (table 1 and supplementary data 3).

Over represented GO annotation terms of potential interest among transcripts induced by SYT-SSX1 in hMSCs included “nervous system development” and “central nervous system development”, consistent with the possibility that SYT-SSX1 exerts pressure toward neuronal differentiation in pluripotent stem cells. Genes induced by SYT-SSX1 belonging to these GO terms include the predicted imprinted genes *HES1, SOX8 and SPON2*, all of which have CpG islands in their TSS. It is therefore possible that SYT-SSX may affect the expression of functionally related groups of genes through a general mechanism that perturbs methylation and imprinting.

### Single Population Transcriptome Analysis

Despite numerous studies, MSCs are still ill-defined with respect to their physical, phenotypic and functional properties [46], [47]. The four independent hMSC populations used in the present study were isolated and cultured according to standard protocols and displayed homogeneity for expression of the handful of standard markers used for their isolation. Nevertheless they were derived from donors of different ages, albeit all younger than 16 years, and functional heterogeneity among them could not be excluded. We therefore compared the effect of SYT-SSX expression in the different hMSC populations.

We first performed statistical analysis of transcriptome changes induced by SYT-SSX1 in each of the four different MSC isolates and found batch-related variability in the transcription profiles with some genes affected in some of the batches but not in others and the same genes affected to varying degrees among the batches. Complete lists of genes affected by SYT-SSX1 in each single MSCs batch are reported in table S2. Among the genes that were affected in some populations but not in others several have been shown to be related to SYT-SSX expression in other studies[35], [48], [49].

**Figure 2:**
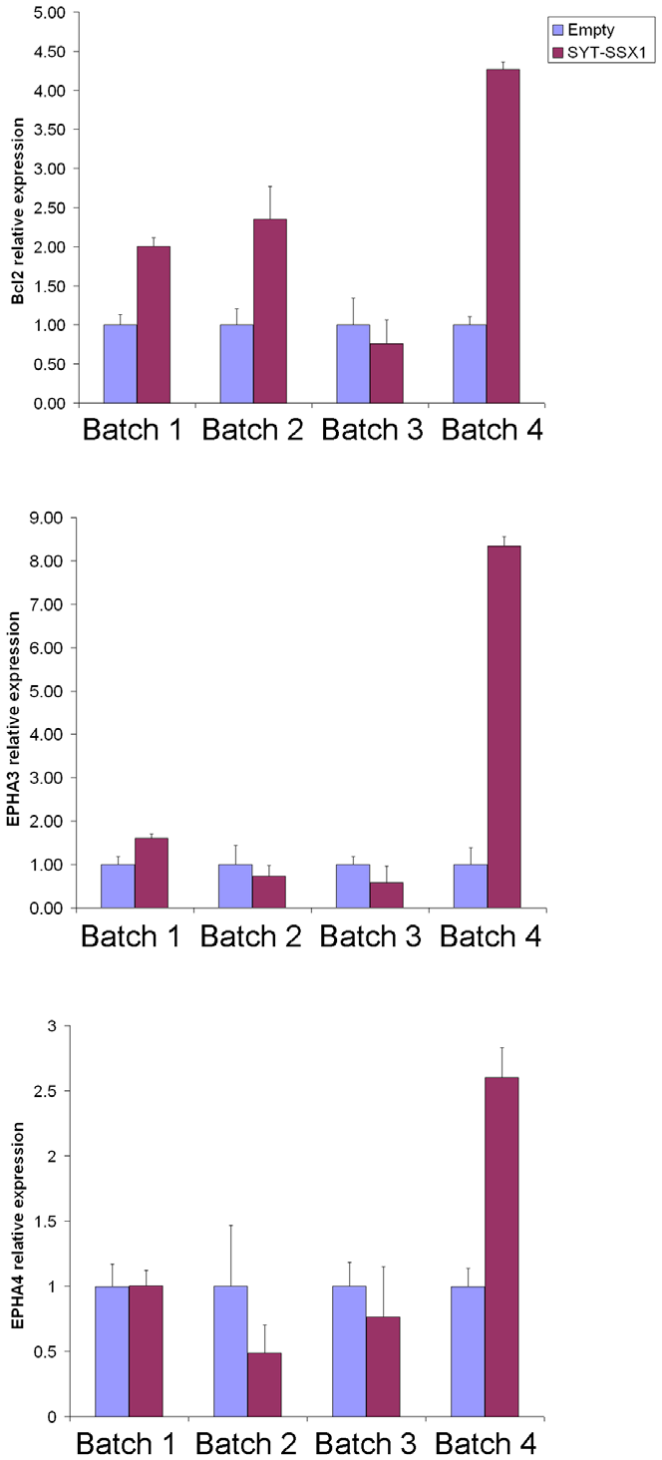
Induction by SYT-SSX1 of *BCL2*, *EPHA3* and *EPHA4* transcripts in four human MSC populations. Histogram representation changes in the expression of *BCL2*, *EPHA3* and *EPHA4* transcripts. Data are normalized to Cyclophilin, and a comparative Ct method was used for the analysis. All experiments were done in triplicate. Mean values+/−SD are shown.

Ephrins provide one example of cell batch-dependent gene regulation by SYT-SSX. Several ephrin receptor/ephrin pathway components, including ephrin receptors A4, A8, B2 and B3 and ephrins B1, A3 and A4 have been recently shown to be induced by SYT-SSX2 in NIH3T3 and other cell lines of mesenchymal and epithelial origin [35]. We observed a broad induction of ephrins and ephrin receptors in only a single hMSC population (batch 4), where ephrins A1 and B3 and ephrin receptors B1, A4 and A3 were induced by SYT-SSX1. Among the other hMSC populations, ephrin B2 was repressed in 2 batches (1 and 3) while ephrin receptor B1 was induced in batches 3 and 4 but not in the other 2 batches. Similarly, *BCL2*, one of the genes whose overexpression has even been suggested to constitute a molecular marker of synovial sarcoma [48], [49], was induced in two batches of MSCs (batches 2 and 4) but not in the other two. Changes in expression, as assessed by microarray analysis, of *BCL2*, *EPHA4* and *EPHA3* were validated by real time PCR in each MSC population and confirmed the microarray observations (Figure 2).

Cell population-related gene expression variability was observed not only for single genes but also for categories of genes that were found to be affected by SYT-SSX1 expression. Thus, overrepresentation of genes containing GpG islands differed from batch to batch with some populations showing significant overrepresentation only among induced (batch 3 and 4) and others showing highly significant overrepresentation among both induced and repressed genes (batch 1and 2). Induction of only four of the chromatin related group of genes was common to all populations whereas that of most of the others was batch-specific (table S4, table S5 and table S6).

**Figure 3:**
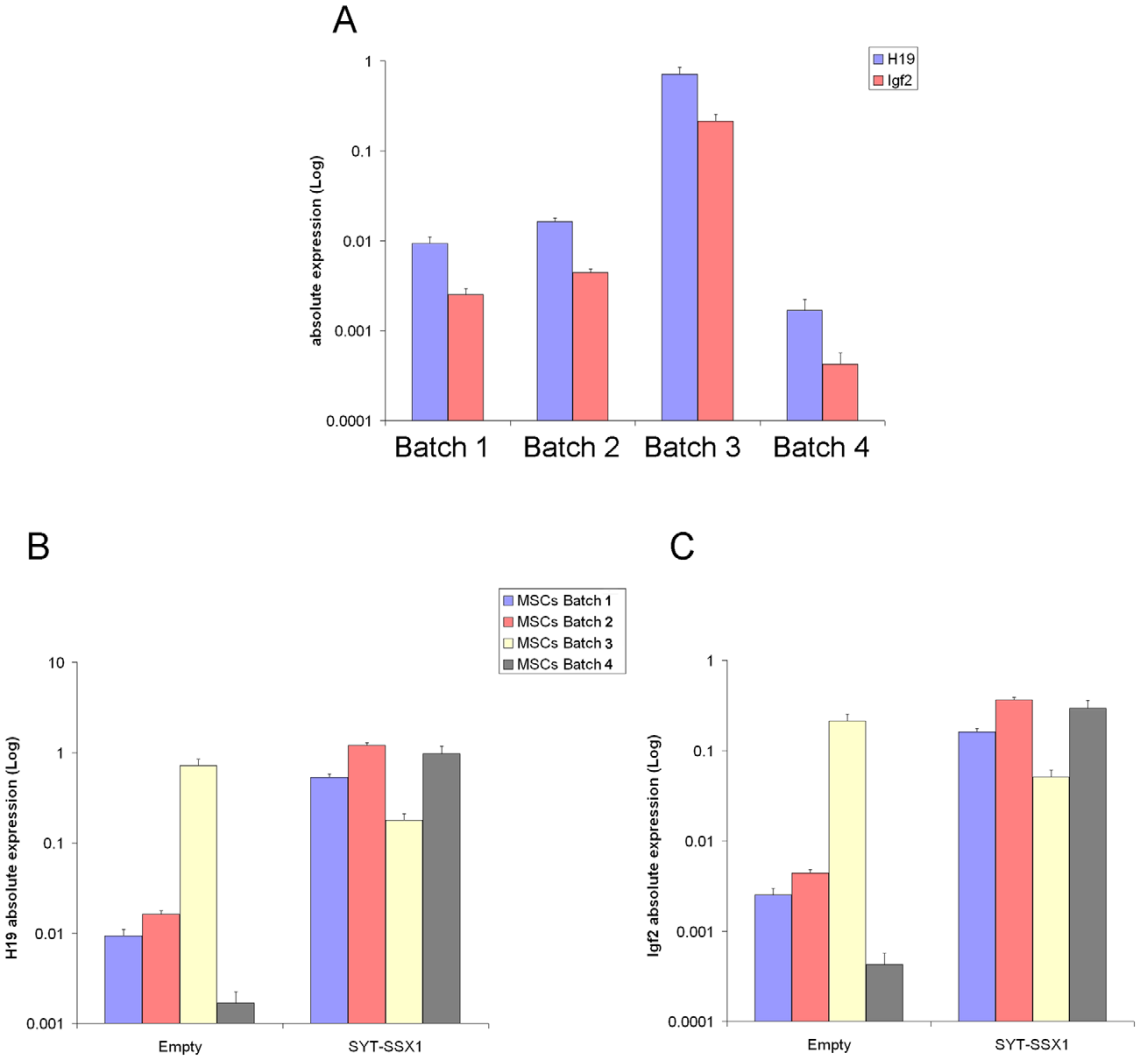
Baseline and SYT-SSX1- induced *H19* and *Igf2* RNA expression in the four populations of human MSC. (A) Baseline *H19* and *IGF2* RNA expression. Quantitative RT-PCR analysis was performed as described in materials and methods on cDNA samples obtained from each of the four populations of human MSC. A Taqman probe was used for *H19* and h*IGF2*exon8 F and h*IGF2*exon9 R primers together with universal probelibrary 60 were used to test all *IGF2* transcripts. Quantitation of target RNA, normalized to an endogenous control (Cyclophilin), was performed using the absolute quantitation method (Applied Biosystems). Results, reported on a logarithmic scale, are representative of 3 independent experiments. Error bars reflect results of triplicate PCR tests. (B and C) SYT-SSX1-mediated induction of *H19* and *IGF2* messenger RNA. Quantitative RT-PCR analysis was performed as in (A) on cDNA samples obtained from each of the four populations of human MSC infected with SYT-SSX1 or an empty pMSCV neo vector and selected for 12 days with 0.5 mg/ml of G418.

The imprinted gene class was over-represented in the lists of induced genes in all four hMSC batches. Batch-dependent variation was reflected by the significance of the induction, batches 2 and 4 having lower p values than batches 1 and 3 (table 2). A few imprinted genes were regulated in some populations only. Thus, *MEST* was repressed only in batches 1 and 3, *DLK1* only in batches 2 and 4, and *PEG3* only in batches 1 and 2. Other imprinted genes, such as *TPP12* were also regulated in only a fraction of the populations (2 and 3) but in opposite directions, being repressed in batch 2 and induced in batch 3. Global statistical analysis suggests that the experimentally confirmed imprinted genes affected by SYT-SSX1 in all MSCs include *H19, CDKN1C, DLK1, DIRAS3* and *IGF2* but that, remarkably, most of them (*IGF2, H19, CDKN1C* and *DLK1*) show different expression profiles in the four cell batches, namely, strong induction in batch 4, more moderate induction in batches 1 and 2 and no induction in batch 3 (Table 3). These observations were validated by real time PCR for *H19* and *IGF2* (figure 3).

**Table 2:** Lists of imprinted genes (gene symbols), induced or repressed by SYT-SSX1 expression in four independent MSC populations.

		MSC Batch 1		MSC Batch 2		MSC Batch 3		MSC Batch 4	
		induced	repressed	induced	repressed	induced	repressed	induced	repressed
**IMPRINTED (experimentally confirmed)**	expected number	1	1.2	0.4	0.5	0.9	0.7	0.6	
	observed number	4	2	5	1	1	1	4	0
	P value	0.015	0.34	5.10E-05	4.20E-01	0.6	0.49	0.0024	
		*		*				*	
		*DIRAS3*	*MEG*	*CDKN1C*	*TFPI2*	*TFP12*	*MEST*	*CDKN1C*	
		*IGF2*	*MEST*	*DLK1*				*DLK1*	
		*PEG3*		*H19*				*H19*	
		*SNRPN*		*IGF2*				*IGF2*	
				*PEG3*					
		MSCs Batch 1		MSCs Batch 2		MSCs Batch 3		MSCs Batch 4	
		induced	repressed	induced	repressed	induced	repressed	induced	repressed
**IMPRINTED (all)**	expected number	3.3	4.1	1.4	1.9	3.2	2.3	1.9	
	found number	11	4	11	4	8	1	8	0
	P value	0.00051	0.59	1.70E-07	1.20E-01	0.014	0.9	0.00077	
		*		*		*		*	
		*COL9A3*	*MEG*	*CDKN1C*	*C20orf82*	*COL9A3*	*MEST*	*CDKN1C*	
		*DIRAS3*	*MEST*	*COL9A3*	*IFITM1*	*E2F7*		*COL9A3*	
		*HES1*	*PHPT1*	*DLK1*	*PRDM16*	*FOXF1*		*DLK1*	
		*HOXA3*	*ZFP36L2*	*H19*	*TFPI2*	*HES1*		*H19*	
		*HOXB3*		*HES1*		*HSPA6*		*HES1*	
		*HSPA6*		*HOXB2*		*SOX8*		*IGF2*	
		*IGF2*		*HOXB3*		*TFPI2*		*SOX8*	
		*PEG3*		*IGF2*		*WDR8*		*SPON2*	
		*SNRPN*		*PEG3*					
		*SOX8*		*SOX8*					
		*ZNF738*		*SPON2*					

Statistical analysis for the over-representation is reported. Data refer to the geneimprint datasets limited to the experimentally confirmed or inclusive of predicted imprinted genes (all). Significant P values (P<0.05) are marked by an asterisk.

**Table 3:** SYT-SSX1 induced fold change (log2) of imprinted genes expression in various hMSC populations.

	MSC Batch 1	MSC Batch 2	MSC Batch 3	MSC Batch 4
*CDKN1C*	0.906	2.407	−0.819	1.598
*DLK1*	0.878	3.486	0.064	3.059
*H19(224646_x_at)*	0.346	2.153	0.069	5.071
*H19(224997_x_at)*	0.312	0.534	−0.096	4.410
*DIRAS3*	2.284	0.828	0.769	0.602
*IGF2*	4.282	3.893	−0.354	7.215

Single batch gene expression analysis according to Gene Ontology (GO) annotation, revealed remarkable variation among the batches (table S3). For example, in the list of genes induced by SYT-SSX1 in MSCs population 4, the “nervous system development” GO term was strongly over-represented (Pvalue 10^−10^) along with several other related terms including, “neurogenesis”, “neuron development”, “neuron differentiation”, “neurite development”, “ephrin receptor activity”, “axonogenesis”, “cell morphogenesis involved in neuron differentiation” and “neurite morphogenesis” (all with a p value<10^−5^). The GO term “nervous system development” was also over-represented in batch 2 (p value 10^−9^) but not in batch 1 or 3, consistent with SYT-SSX1-mediated pressure toward neuronal differentiation in some MSC populations only.

Single population analysis also suggested a variable effect of SYT-SSX on the expression of genes encoding proteins that constitute components of the extracellular matrix, mediate cell-extracellular matrix interactions, and participate in vascular development, angiogenesis, tissue remodeling and cell motion. In batch 3, GO term analysis suggests inhibition of expression of these genes. Similarly, in batch 1, the list of repressed genes included over-representation of the GO terms “extracellular matrix”, “extracellular region”, “proteinaceous extracellular matrix”, “cell adhesion”, “integrin complex”, “integrin-mediated signaling pathway”. By contrast, in batches 2 and 4, the GO terms “cell adhesion”and “extracellular matrix” were over-represented in the list of induced genes. Expression of these categories of proteins is important for the biological function of both normal and cancer stem cells, the former requiring release from their niche in the bone marrow in order to be recruited to target tissues where they undergo *in situ* differentiation and contribute to tissue regeneration [50], the latter using similar mechanisms to disseminate and form metastases. CXCR4 and integrins are among the main effectors of these functions, and induction of CXCR4 was observed in populations 2 and 4.

Because the level of SYT-SSX expression was comparable in all four MSC populations (figure 1), and because each MSC population was subjected to identical culture conditions when micorarray analysis was performed, the observed batch-related variability appeared to be independent of the expression level of the fusion protein and *in vitro* culture-related differences. On the other hand, the observed variability of the response to SYT-SSX expression did not appear to be random, as suggested by statistical analysis. Based on this notion, we hypothesized that the qualitative or quantitative variations in expression of the type and number of genes in general and within each specific category, could be attributed to differences in the initial status of each single hMSC population at the time of fusion gene introduction. These putative differences in status may determine the effect of SYT-SSX1 and may consist of epigenetic variations, possibly linked to individual traits that may be donor age and/or environmental factor-dependent.

### Comparison of the *hMSC^SYT-SSX1^* Transcriptome with that of Embryonic Stem Cells and Signatures of Multiple Pathways Associated with Stemness

To address a possible role of SYT-SSX1 in affecting hMSC plasticity and stemness, we computed the overlap between the lists of differentially expressed genes with recently published lists of stemness markers [51], [52]. The analysis was performed using datasets of embryonic stem cell identity as well as signatures of multiple « stemness » pathways, including polycomb regulated genes, target genes of *Nanog*, *Oct4*, *Sox2* and *c-Myc*, and target genes of the RNA binding protein *Nanos* (*NOS*). Both lists derived from the analysis of the four hMSC population together and lists derived from single population analysis were used, (table S7). No significant over-representation of *Nanog*, *Oct4*, *Sox2*, *c-Myc* and *NOS* target genes was observed in either analysis and only limited similarity to the embryonic stem cell signature was noted. By contrast, polycomb target genes appeared to be affected by the expression of SYT-SSX1. When using lists obtained by analysing the four hMSC batches together, a significant over-representation of polycomb target genes was observed among SYT-SSX1-induced but not among SYT-SSX1-repressed genes; higher significance was observed uon analysis of *Suz12* target genes (p value of 10–12).

In single population analysis, overrepresentation of polycomb target genes was found among the lists of SYT-SSX1-induced genes in all four hMSC populations. Nevertheless some batch-dependent variability was observed. Thus, batch 4 displayed a highly significant (p value 10–20) over-representation of polycomb target genes in the lists of SYT-SSX1-induced genes but no over-representation in the lists of SYT-SSX1-repressed genes. Other batches disclosed less significant over-representation of polycomb target genes in the lists of SYT-SSX1-induced genes, but batch 3 revealed significant polycomb target gene over-representation in the lists of SYT-SSX1 repressed genes.

Taken together these data suggest that SYT-SSX1 in pluripotent cells may participate in the induction/maintainance of stemness features by modulating polycomb activity. They support recent findings that demostrate deregulation of polycomb activity by SYT-SSX2 in the U2OS cell line [53].

### Comparison of the hMSC^SYT-SSX1^ Transcriptome with Sarcoma Gene Expression Signatures

To address possible similarities between the gene expression profiles induced by SYT-SSX1 in hMSCs and those observed in sarcomas, we computed the overlap of the lists of differentially expressed genes with the sarcoma signatures identified in a recent study [23]. Both the list derived from analysis of the 4 batches together and lists derived from single batch analysis were used and the results are summarized in tables (table S8).

Comparison of the lists of both repressed and induced genes derived from analysis of the 4 batches together revealed a significant overlap with the reported synovial sarcoma signature (p value about 10^−3^). A significant overlap was also found with genes repressed in fibrosarcomas and those induced in gastrointestinal stromal tumors (GISTs). However, the synovial sarcoma signature was the only one that displayed overlap with hMSC^SYT-SSX1^ lists of both induced and repressed genes.

Interestingly, comparison of single batches resulted, in the case of 3 out of the 4 hMSC populations, in enhanced similarity with the synovial sarcoma signature. Thus, the gene expression profile of batch 4 overlapped significantly (p<10^−7^) with both induced and repressed synovial sarcoma gene signatures, whereas that of batch 3 overlapped significantly with the list of repressed (p<10^−16^) but not with that of induced SS genes.

Common genes comprised transcripts belonging to the imprinted class, including *IGF2*, *DLK1*, *PEG3* (in some batches), chromatin related genes, including histone clusters, genes with GpG islands within the transcription start site, such as *KNNQ2* and *CXCR4*, and genes that could play a relevant role in tumor progression and survival such as *BCL2*.

Taken together, these data suggest that MSC provide a cellular context that permits SYT-SSX to induce a transcriptional profile similar to the one that characterizes synovial sarcoma but that among individual MSC populations, some are more permissive than others for these transcriptional changes. Among the observed transcriptional changes, those involving *IGF2*, appear to be particularly relevant in view of both their behavior in different hMSC isolates in response to SYT-SSX expression (fig. 3 and table 3) and the recognized role of IGF2 in the initiation and progression of several types of sarcoma. We therefore analyzed in detail baseline *IGF2* and *H19* expression and the corresponding expression changes induced by SYT-SSX1 in the four hMSC populations.

### Baseline and Allele-Specific *H19/IGF2* Expression in hMSC

Multiple transcripts of *IGF2* are produced as a result of alternate promoter usage and splicing: promoter P2-P4-dependent transcripts do not contain exons 1–3, which are incorporated in P1 promoter-dependent counterparts. Promoter P0, on the other hand, drives expression of transcripts that include exon 3 but not exons 1 and 2 [54].

Real time PCR experiments were performed using a panel of different primer/probe sets to assess the expression of several different *IGF2* transcripts and showed that both P1 and P2–P4 driven transcripts were expressed in all the four MSC populations (data not shown). Absolute quantification (shown on a logarithmic scale in figure 3A) of *H19* and *IGF2*, using primers encompassing exons 8 and 9 (all transcripts), revealed higher expression of *H19* than *IGF2* in all four populations of hMSC. However, the baseline expression level of each of the two genes varied from batch to batch (figure 3B,C). Batch 4 had the lowest level of *IGF2*, about 6 fold lower than batch 1, 10 fold lower than batch 2 and about 500 fold lower than batch 3. *H19* transcripts displayed a very similar profile, with the highest and lowest expression in batches 3 and 4, respectively.

**Figure 4:**
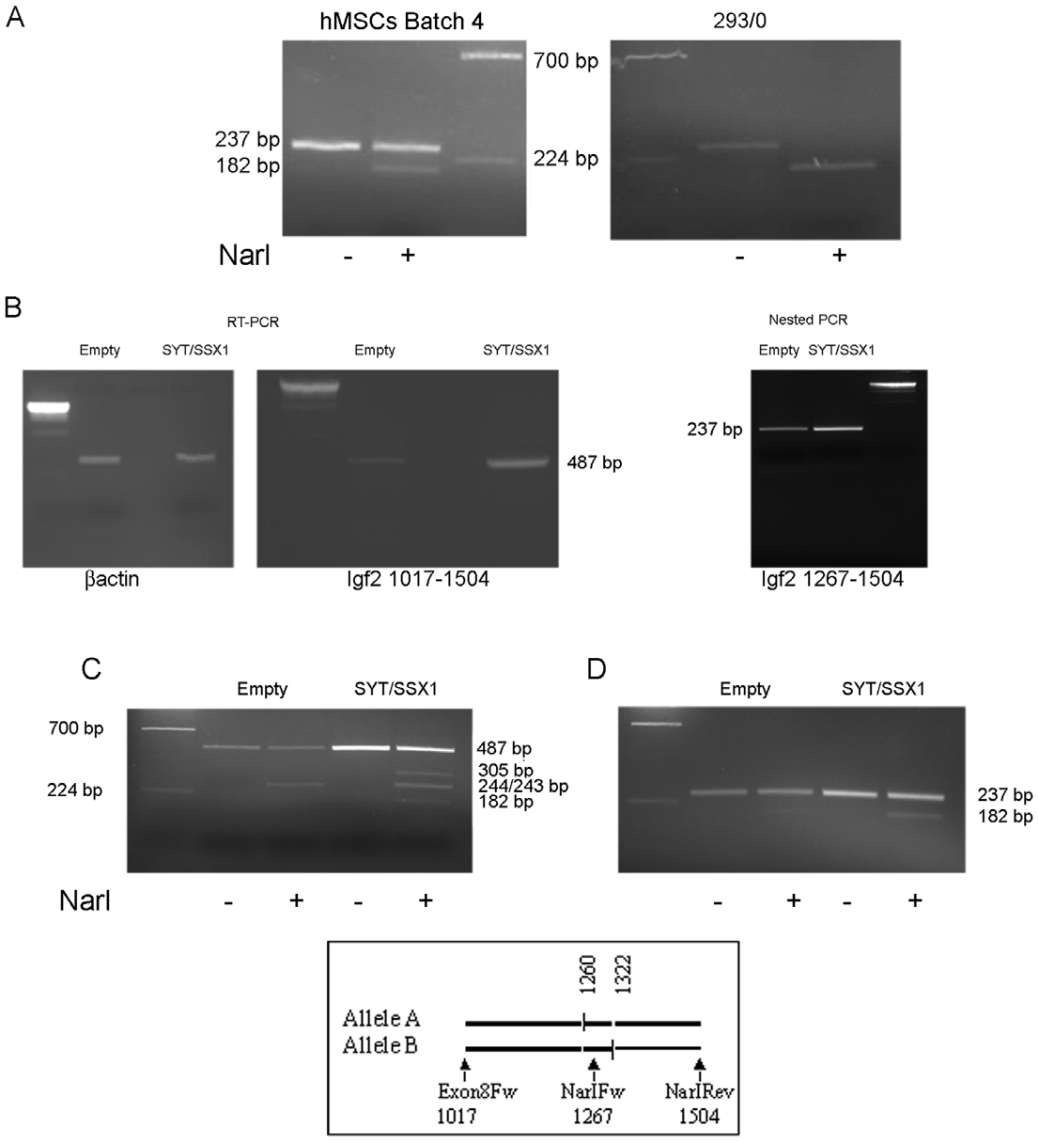
Differential *IGF2* allelic expression in hMSC population 4 and allele specific *IGF2* induction by SYT-SSX1. Differential allelic expression was investigated by RT-PCR and subsequent restriction fragment length polymorphism (RFLP). A known C/T single nucleotide polymorphism at position 114671 of the human *IGF2* gene (NCBI AC132217) (rs2230949) for which this population was informative, was analyzed. (A) Genotype analysis: genomic DNA was extracted from hMSC population 4 infected with SYT-SSX or an empty pMSCV neo vector and from a clone of HEK-293 cell line (293-0). A 237 bp fragment spanning the C/T NarI polymorphic site was amplified using NarI forward and NarI reverse primers. A heterozygous profile at this position produces fragments of 182 bp (visible on the gel) and 55 bp (not visible on the gel, from the allele carrying a C) and an undigested fragment (from the allele carrying a T). Digestion efficiency was controlled by a parallel digestion on the same fragment derived from 293/0 cell lines which show an homozygous profile with both alleles carrying a T at this site. (B) *IGF2* expression: A RNA-specific 487 bp fragment, spanning the *IGF2* C/T polymorphism was amplified from hMSC population 4 infected with SYT-SSX or an empty pMSCV neo vector, by quantitative RT-PCR using cross intron primers Ex8 forward and NarI reverse. Primers amplifying a human βactin fragment were used as a template control. The 487 bp bands were separated on a 1.5% agarose gel and, after purification, either used for RFLP analysis shown in C) or as a template for a nested quantitative PCR using NarI forward and NarI reverse primers that produce a 237 bp amplicon. (C and D) NarI restriction digestion profiling of the 487 bp fragment (C) and of the 237 bp fragment (D) obtained as described in B The schematic representation shows the position of the 2 NarI sites (vertical lanes) and the position of the primers used (arrows). The heterozygous profile shown at both sites is the only one consistent with the generation of the 3 bands at 305, 244/243 and 182 bp, observed by NarI digestion, given the heterozygosity for the NarI site at position 114671 (shown in A). DNA fragments were incubated at 37°**C** for 3 hrs in the appropriate digestion buffer with or without 4 U of NarI. Fragment size analysis was performed on a 2% agarose gel, first lane of each gel shows Lambda DNA/*Bst*E II Digest marker, fragment size is indicated. C) Bands of 305 and 182 bp are derived from one allele, whereas 244/243 bp bands are derived from the other allele; the heteroduplex undigested PCR product is also shown. D) In the 487 bp fragment, the heterozygous profile at position 114671 of the *IGF2* gene produces 3 fragments: a 182 bp (visible on the gel) and a 55 bp (not visible on gel) derived from the allele carryng a G at this position and the undigested fragment derived from the other allele or from the heteroduplex product. The differential intensity of the bands shows that induction of *IGF2* by SYT-SSX1 involves both alleles.

These observed differences in the basal expression level of *IGF2* suggest that the populations of hMSCs may have a different methylation status at the *H19/IGF2* ICR, possibly resulting in mono-allelic expression in some cases and bi-allelic expression in others. To test this hypothesis differential allelic expression analysis was performed by RT-PCR and subsequent restriction fragment length polymorphism (RFLP). A known polymorphic NarI site at position 114671 of the human *IGF2* gene was analyzed and two (4 and 1) of the four MSC populations were found to be informative (figure S2). As hypothesized, monoallelic expression of *IGF2* was observed in MSC population 4 (figure 4 and figure S2) whereas bi-allelic expression was observed in population 1 (figure S2).

### Allele-Specific *H19/IGF2* Expression Changes Induced by SYT-SSX1 in hMSC

Real time PCR assessment of the effect of SYT-SSX1 on *IGF2* and *H19* expression, revealed that 3 out of 4 hMSC populations displayed strong induction of both messages (figure 3B and C). The effect of SYT-SSX1 was inversely proportional to the baseline expression level of the two genes prior to infection: the lower their baseline expression level, the stronger was the induction by SYT-SSX. In cells with the highest *H19* and *IGF2* expression (batch 3), SYT-SSX1 introduction had no further expression inducing effect on either gene. These data were consistent with observations made using Affymetrix micorarrays (table 3) and correlated with the different degrees of induction of both *IGF2* and *H19* transcripts as quantified by real time PCR, ranging from 60–80 fold (batch 1and 2) to 600–700 fold (batch 4). Induction of *IGF2* transcripts by SYT-SSX1 within each cell population was comparable when different primer sets were used and various *IGF2* transcripts were selected. No intra-population transcript discrepancies were observed (data not shown).

Assessment of results obtained on the induction of *IGF2* limited to batch 4 is consistent with a SYT-SSX-mediated switch from mono-allelic to bi-allelic expression according to the shared enhancer model, suggesting that, in these cells, the fusion may have a selective effect on the silent allele. To verify this notion, we tested allelic *IGF2* expression changes induced by SYT-SSX in hMSC population 4, which contained the polymorphic NarI site in the *IGF2* coding sequence (figure 4A.). SYT-SSX1-induced upregulation of *IGF2* expression was measured by semi-quantitative-RT-PCR and subsequent RFLP analysis using primers corresponding to sequences located in exon 8 and 9 and spanning two NarI polymorphic sites (Figure 4B). To analyze allele specific induction, taking into account heteroduplex formation and ruling out DNA contamination, we performed RFLP analysis on both the first amplicon containing two polymorphic NarI sites (fig. 4C) and on a second fragment containing only one polymorphic site (fig. 4D). In both cases restriction fragment analysis showed that hMSC population 4 expressed *IGF2* from only a single allele and that introduction of the fusion gene induced expression of the silent allele. Nevertheless, we also observed a significant, SYT-SSX1-dependent increase in the activity of the active allele, since the 244/243 bp bands derived from digestion of this allele were more intense in SYT-SSX1-expressing cells than in cells infected with an empty vector (figure 4C). This was also visible in figure 4D (237 bp undigested bands) although, in this case the possible presence of undigested heteroduplexes must be taken into account.

These observations demonstrate that SYT-SSX1 can induce loss of imprinting in cells that show an intact imprinted status at the *H19/IGF2* locus. On the other hand, the observation that, in batch 4, the activity of the non silent allele can also be increased by SYT-SSX1 supports the notion that additional mechanisms are involved in the induction of *IGF2*, at least in some hMSCs.

### Allele Specific Methylation

**Figure 5:**
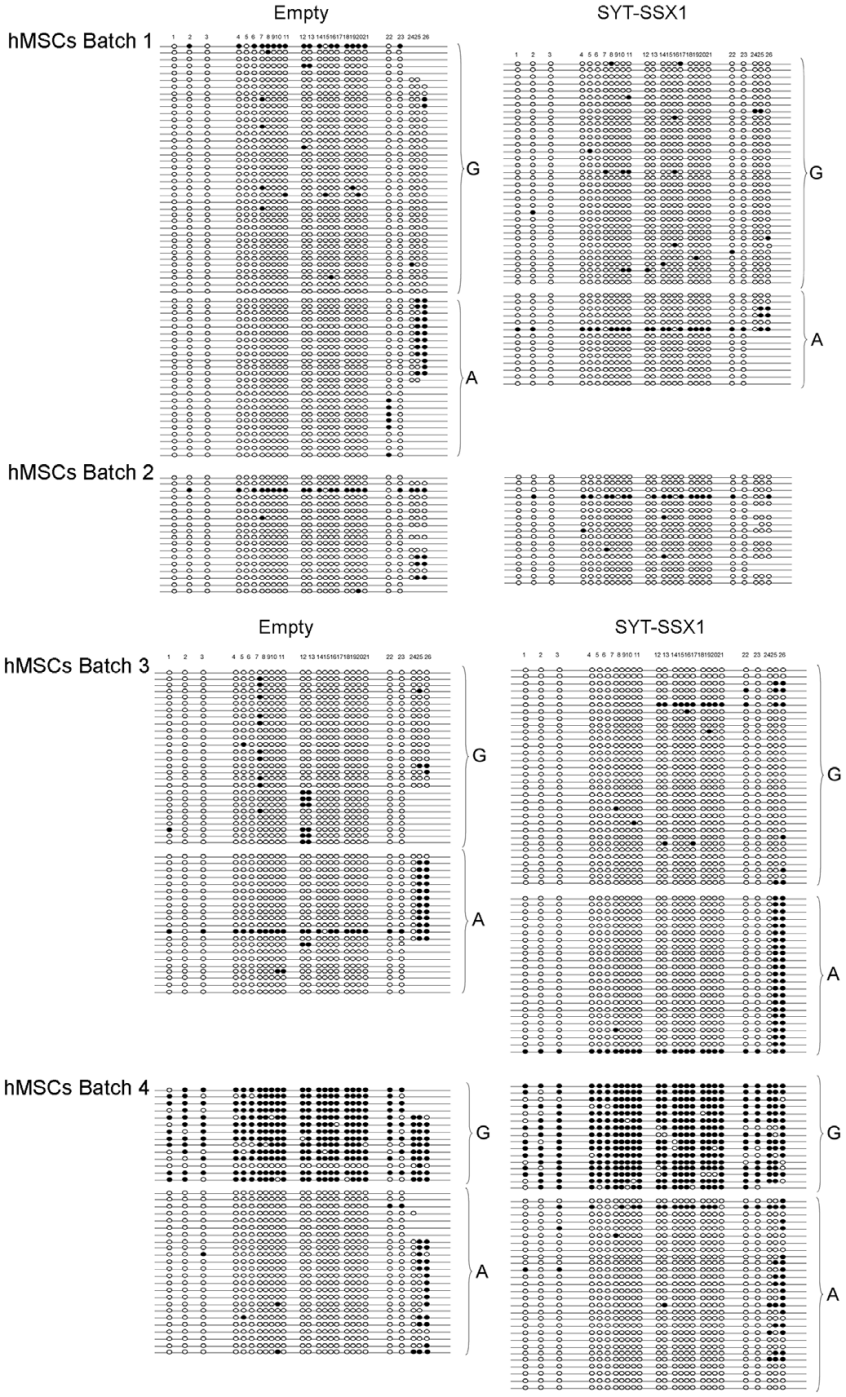
Methylation profile as determined by bisulfite transformation analysis at the *H19* ICR region amplified by primers BS-7712sense and BS-8192antisense in different human MSC populations. Each line represents one cloned segment; the 26 individual CpGs (circles) are numbered 1–26. Clones derived from each allele are grouped and indicated. White circles represent un-methylated and black circles methylated CpGs.

To gain insight into the mechanism(s) whereby SYT-SSX might induce *IGF2* in different hMSC populations, we compared the DNA methylation status twelve days following infection with SYT-SSX1 or empty vector. We first analyzed a region in the *H19* ICR (AF125183: 7712–8192), including the sixth CTCF binding site (figure 5) that has been suggested to be a key regulatory domain for switching between *H19* and *IGF2* expression. It is the only out of 7 binding sites in the human ICR that has been demonstrated to have allele specific methylation in normal human embryonic ureteral tissue [55] and been shown to be hypomethylated in human bladder cancer and some osteosarcomas [55], [56], but hypermethylated in Wilms' tumor and colon cancer [57].

We first tested DNA from non transformed cells for the presence of polymorphic sites in this region by direct sequencing of PCR products obtained using different combinations of the following forward and reverse primers: H19- 7712Fw, H19- 8192R, H19-7565Fw, H19-8298R and H19-7895R. Three polymorphic sites are known to exist within this region (rs10732516) (rs2071094) (rs2107425). DNA extracted from our four hMSC populations did not show double peaks at position 7966 (rs10732516) or at position 8008 (rs2071094). However, hMSC populations 1, 3 and 4 displayed a double G/A peak at position 8097 (fig. S3C) whereas population 2 showed a single peak (not shown).This SNP (rs2107425) affects an NlaIII restriction site (fig. S3A) and we used restriction fragment length polymorphism (RFLP) analysis to determine heterozygosity. Following NlaIII digestion of the specific PCR product obtained from each of the 3 MSC populations, we observed a heterozygous profile consisting of 2 fragments of 215 bp (from the allele containing a G) and 296 bp (from the allele containing an A) in addition to common fragments of 81, 87 (comigrating) and 17 bp (not visible on the gel, fig. S3B). Bisulfite transformation analysis, based on the presence of this polymorphic site, allowed assessment, in populations 1, 3 and 4, of allele-specific methylation at the 26 CpGs included in the region amplified by primers BS-7712sense and BS-8192antisense. In population 2 only a general assessment without allelic distinction was made.

The methylation status we found at this region was highly divergent from population to population: MSC batch 4 was the only one that showed an intact imprinting status with an overall methylation of 83% on one allele and 4.6% on the other (table 4). Populations 1 and 3 displayed a profile compatible with loss of imprinting, with no major allele-specific difference in methylation and a much lower overall methylation (around 4–6%). Although we could not discriminate between the two alleles in population 2, the overall methylation in this region was substantially lower than in batch 4 (6.4% versus 44%). Even when the closely apposed CpGs that constitute the putative sixth CTCF binding site (CpGs 7–11 in figure 5) are considered, only batch 4 showed allelic specific methylation. In all the other batches methylation of this region was lower without significant difference between alleles.

**Table 4:** Percentage of methylated CpGs at the *H19* ICR region amplified by primers BS-7712sense and BS-8192antisense in different human MSC populations.

CpG	1–23	1–23	23–26	23–26	7–11	7–11	1–26	1–26
	EMPTY	SYT-SSX	EMPTY	SYT-SSX	EMPTY	SYT-SSX	EMPTY	SYT-SSX
Batch 1 allele with A	1%	7.1%	54%	22%	0%	5.7%	4.6%	7.9%
Batch 1 allele with G	4.3%	2.1%	3%	2.9%	5.8%	4.1%	4.2%	2.2%
Batch 2	4.8%	5%	23%	2.6%	6.6%	5.9%	6.4%	5%
Batch 3 allele with A	4.8%	4.5%	61.5%	67%	6.7%	5.2%	9%	12%
Batch 3 allele with G	3.9%	2.4%	7.4%	10.4%	0.7%	1.25%	4.1%	3.4%
Batch 4 allele with A	1%	3.3%	43%	34.5%	1.7%	2.8%	4.6	6.8
Batch 4 allele with G	82%	89%	90%	82%	90%	96%	83%	88%

Specific alleles are indicated.

These data correlate with those obtained by measuring *IGF2* expression by RT-PCR although they cannot explain the *H19* expression pattern. The lower level of *IGF2* in population 4 is compatible with monoallelic expression observed by RFLP analysis that can be explained by differential DNA methylation according to the shared enhancer model. Conversely, in populations 1, 2 and 3, the higher baseline *IGF2* expression level could be explained by bi-allelic expression derived from an almost un-methylated status on both alleles at the sixth CTCF binding site. Bi-allelic *IGF2* expression was confirmed in population 1 by RFLP.

The last three CpGs included in our amplicon (CpGs 23–26 in figure 5) seemed to constitute a separate region in these cells and batch 1 and 3 displayed allele-specific differential methylation only at these sites (figure 5). In batch 4 they show conformity with the other CpGs regarding the imprinting status (1–23) but appeared more heavily methylated.

Although these last residues were not clearly readable in all of the sequenced clones (missing residues in figure 5) the number of clones in which they were determined is sufficient to reveal the higher methylation and the allelic distinction.

### Allele Specific Methylation Changes Induced by SYT-SSX1

We next compared the methylation pattern of the same *H19* IRC, including that 6^th^ CTCF binding site, among all four hMSC populations twelve days following infection with SYT-SSX1-containing retrovirus or an empty vector. Expression of SYT-SSX1 in population 4 resulted in modest hypermethylation, the effect being more marked on the methylated (paternal) allele (from 83% to 88% methylation when all 26 CpGs were included (Table 4)) than on the maternal un-methylated allele (from 4.6% to 6.8% when all 26 CpGs were considered (Table 4)). This magnitude of variation is similar to that shown by others in HEK- 293 cells [11]. Interestingly, the observed increase in methylation by SYT-SSX1 was limited to the first 23 CpGs and this was particularly the case on the maternal allele (%methylation rises from 1% to 3.3% when CpGs 1–23 only are included). By contrast, at the last three CpGs, SYT-SSX1 expression produced the opposite effect, with considerable hypomethylation on both alleles.

In hMSC batch 1, moderate hypermethylation by SYT/SSX1 limited to one allele at CpGs 1–23 accompanied by considerable hypomethylation at residues 23–26 on both alleles was also observed. In batch 2 we could not see any change in methylation at positions 1–23, which may be due to compensation between the two alleles that could not be distinguished in these cells. In either case, strong hypomethylation was observed at CpG sites 23–26. Finally, population 3 showed a slight increase in methylation on one allele (from 9% to 12%) when the entire region was assessed that was attributable not to hypermethylation of the first 23 CpGs, which remained unchanged on both alleles, but, contrary to the other populations, to hyper-methylation of the last three.

Thus, in all 4 populations, residues 1–23 or, more specifically, residues that correspond to the sixth CTCF binding site (CpGs 7–11 table 4) displayed the same trend in response to SYT-SSX1 expression. In this regard therefore, population 3, the only population in which SYT/SSX1 did not induce *IGF2* was also the only one in which the fusion did not induce an increase in methylation at the insulator binding site. Nevertheless, SYT-SSX1 significantly affected methylation of CpGs 23–26 in this population but in the opposite direction to that observed in the other populations.

**Figure 6:**
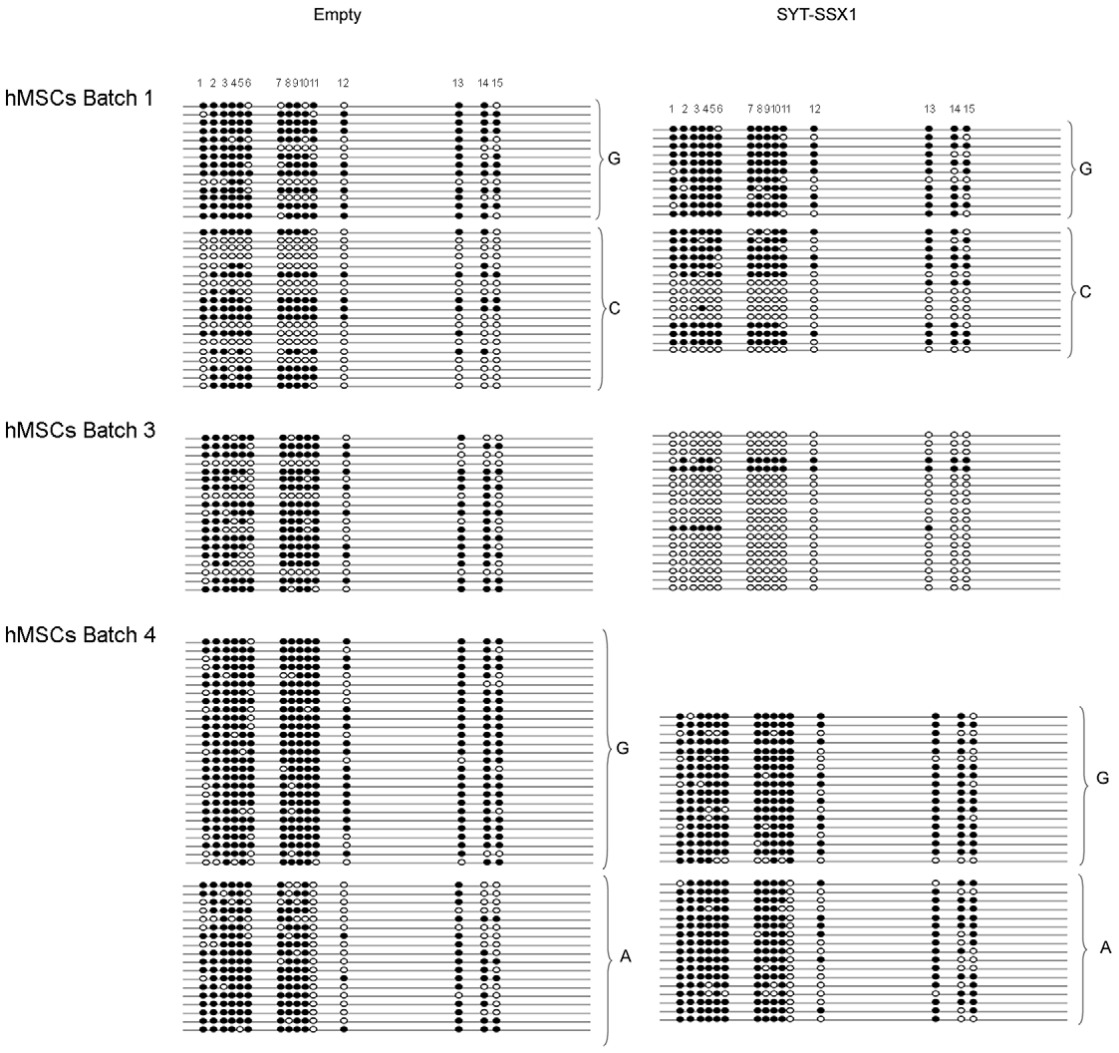
Methylation profile as determined by bisulfite transformation analysis in the region amplified by primers BS-13212sense and BS-13548 in different human MSC populations. Each line represents one cloned segment, the 15 individual CpGs (circles) are numbered 1–15. Clones derived from each allele are grouped and indicated. White circles represent un-methylated and black circles methylated CpGs.

We also assessed, by bisulfite transformation analysis, the methylation changes induced by SYT-SSX1 in a second region located outside the *H19* ICR (figure 6). This region, amplified by primers BS-13212sense and BS-13548antisense, was included in a CpG island located 3′ to the *H19* gene. Fifteen CpG sites were analyzed within this region. Allelic discrimination was possible in batches 1 and 4, which had a C/A polymorphism at position 13359 (rs 217133) (batch 4) and a C/G polymorphism at position 13270 (batch 1). Allele-specific differential methylation was also observed in this region although not as markedly as in the ICR (76% on one allele and 40% on the other in batch 1; 87% on one allele and 67% on the other in batch 4) (table 5).

**Table 5:** Percentage of methylated CpGs in the region amplified by primers BS-13212sense and BS-13548 antisense in different human MSC populations.

CpG position	1–15	1–15
	EMPTY	SYT-SSX
Batch 1 allele with G	76.6%	86%
Batch 1 allele with C	40%	52%
Batch 2	67%	11.6%
Batch 4 allele with A	67.4%	78%
Batch 4 allele with G	87%	84.8%

Specific alleles are indicated.

The effect of SYT-SSX1 introduction varied from batch to batch. Strong hypomethylation was induced in batch 3 (from 67% to 11%) whereas batch 1 and 4 showed significant hypermethylation. This increase in methylation involved both alleles in batch 1 but was limited to only one allele (the one less methylated) in batch 4.

## Discussion

We have analyzed the transcriptional effects of SYT-SSX1 expression in bone marrow derived human mesenchymal stem cells isolated from pediatric or adolescent patients. These cells may provide an appropriate model to study synovial sarcoma development, based on the generally recognized notion that SS originates from as yet unidentified pluripotent stem cells capable of mesenchymal and neuroectodermal differentiation. The only available transgenic model of SS [14] thus far, suggests that development of SS is linked to the expression of the early myogenic marker Myf5. Co-expression of early markers for different tissue lineages has been observed in MSCs [58], without necessarily being associated with loss of plasticity. Human MSC expressed *Myf5* with up to a 30-fold population-dependent transcript level variation (data not shown). However, we could not establish whether the observed differences in expression were due to varying enrichment of a specific sub-population or to homogeneous, donor specific, traits.

Human MSC^SYT-SSX1^ display a transcriptional profile with significant similarity to the gene expression signature of synovial sarcoma, supporting the notion that these cells could have features common to the pluripotent mesenchymal cell of origin of SS. Analysis of the transcriptional profile of hMSC^SYT-SSX1^ revealed overexpression of genes related to nervous system development, suggesting that SYT-SSX1 may exert some degree of pressure toward neuronal differentiation in mesenchymal stem cells. Several reports [59], including a recent proteomics-based study [60], have shown a similar gene expression profile among clear cell sarcoma, synovial sarcoma, and MPNST supporting the assumption that these three tumors may be derived from, or differentiate toward, neuroectodermal cells. Rare cases of synovial sarcoma, identified by SYT-SSX expression, have in fact been reported to express neural immunomarkers [61].

Single population analysis according to Gene Ontology (GO) annotation revealed remarkable variation among batches. The observed variation involved single genes whose overexpression has been associated with synovial sarcoma, including *BCL2* and *IGF2* as well as clusters of genes implicated in cell trafficking and differentiation. Thus, some populations of MSC appeared to be more permissive than others for SYT-SSX-induced changes in the expression of genes relevant to fundamental requirements for normal and cancer stem cell biology. Similarly, single population analysis revealed greater similarity of some MSC^SYT-SSX1^ population transcriptomes than others to SS gene expression signatures, supporting the hypothesis that features which distinguish independent hMSC isolates and contribute to their heterogeneity may be key for SYT-SSX function. Our present observations suggest that the nature of these putative features may, at least in part, be epigenetically determined.

Transcriptome analysis of hMSC^SYT-SSX1^ showed that a major effect of SYT-SSX in hMSCs involves changes in the expression of epigenetically regulated genes, including imprinted genes, genes that contain CpG island in their TSS and chromatin related genes. Epigenetic de-regulation has been suggested to be a central effect of the aberrant expression of SYT-SSX and a possible mechanism underlying synovial sarcoma formation. The present transcriptome analysis of hMSC expressing SYT-SSX strongly supports this notion.

Consistent with the variability of MSC^SYT-SSX1^ transcriptome relatedness to SS signatures and that of GO term overrepresentation, single population analysis limited to datasets of epigenetically regulated genes showed marked qualitative and quantitative differences among the four hMSC isolates, the most striking being the divergent effect of SYT-SSX on the expression of imprinted genes. It is therefore conceivable that epigenetic features displayed only by some hMSC populations permit SYT-SSX to affect expression of genes implicated in biological functions relevant to stem cells and SS. We therefore sought divergent epigenetic characteristics among the MSC populations that may explain the significant variations observed in the transcriptional effect of SYT-SSX.

Assessment of the *H19/IGF2* cluster provided support for our hypothesis. *IGF2* is considered to be one of the signature genes of SS and is part of one of the best characterized imprinted clusters. Deregulation of its expression has been suggested to play a role in the development of several types of cancer. Real time PCR experiments revealed that different hMSC isolates display highly variable levels of *IGF2* and *H19* transcripts. Although a complex network of long range interactions and multiple looping are emerging as newly recognized regulators of *H19* and *IGF2* [37], [38], the methylation status at the *H19* imprinting control region (ICR) remains a basic regulatory factor according to the shared enhancer model. Bisulfite transformation analysis revealed a highly divergent methylation pattern among hMSC populations both at the *H19* ICR and in a second region downstream of the *H19* gene. In those populations that were found to be informative, the methylation pattern at the *H19* ICR was shown to be compatible with maintenance or loss of imprinting and to correlate accordingly with a mono or bi-allelic *IGF2* expression that could explain, at least in part, the different level of mRNA measured by RT-PCR. Thus different hMSC populations displayed a different imprinting status at the *H19/IGF2* ICR. Epigenetic variation, which could be a function of numerous factors, including age and environmental conditions, has been shown to characterize stem cells and to play an important role in determining cell commitment and plasticity [62]–[64]. Consistent with this notion, the cells used in the present study were derived from different donors whose age variation, although in the pediatric/adolescent range, could conceivably explain, at least in part, their distinct epigenetic features.

Expression of SYT-SSX in the four populations produced variable epigenetic effects.

Methylation analysis at the *H19* ICR showed modest changes, hypermethylation on both alleles being induced by SYT-SSX in only one population whereas other populations displayed either no effect or opposite effects on the two alleles. The absence of methylation changes in population 3 can be reconciled with the observed absence of induction of *IGF2* expression. Conversely in population 4 the hypermethylating effect of SYT-SSX at the 6^th^ CTCF binding site may explain, in part, the induction of the *IGF2* transcripts. The observed SYT-SSX-dependent switch from monoallelic to biallelic expression of *IGF2* in this population, together with the methylation changes, is consistent with SYT-SSX-induced LOI according to the shared enhancer model. Hypermethylation of both alleles in these cells can also explain the observation that, in addition to the re-expression of the silent allele, expression of the active allele was increased.

In population 1, where a methylation pattern consistent with loss of imprinting and a corresponding baseline bi-allelic *IGF2* expression were demonstrated, the effect of SYT-SSX at the 6^th^ CTCF binding site produced modest but opposite effects on the two alleles. SYT-SSX1-mediated enhancement of *IGF2* and *H19* expression in this population must therefore have been achieved by alternative mechanisms since it cannot be explained by the reactivation of a silent allele. The involvement of alternative and/or additional regulatory factors at the *H19/IGF2* locus that may be directly or indirectly affected by SYT-SSX expression is suggested by several observations emerging from the present study. Concomitant induction of *H19* observed in all cases is not compatible with the sole perturbation by SYT-SSX1 of ICR imprinting. Furthermore the similar activation of both P1 and P2–P4 *IGF2* promoters is also suggestive of the existence of multiple regulatory mechanisms affected by the fusion protein since several independent observations suggest that not all *IGF2* promoters are regulated exclusively by the imprinting control region. It has been reported that in hepatocytes and chondrocytes, *IGF2* transcripts from promoter P1 are derived from both parental alleles, whereas transcripts from promoters P2, P3 and P4 are derived from a single parental allele [65]. These observations suggest that P1 promoter activity could be at least partly independent of the ICR. It is noteworthy that the P1 transcript is reported to be expressed from both parental alleles in postnatal liver and fetal choroid plexus/leptomeninges [66], and that P1 promoter activity was observed not to be exclusively connected to *IGF2* LOI in laryngeal squamous cell carcinoma [67].

Methylation analysis of regions outside the H19 ICR showed that SYT-SSX1 does not affect methylation specifically and exclusively at the *H19* ICR but rather at different discrete regions with even opposite effects in adjacent segments and in different hMSC populations. The exact mechanism whereby SYT-SSX affects methylation and possibly the complex network of long range interactions and multiple looping that regulate the *H19/IGF2* locus remains to be defined. Our data suggest that a specific epigenetic substrate, defined by a normal imprinting status and monoallelic expression of *IGF2* are required for a strong effect of SYT-SSX on *IGF2* expression and that changes in the baseline epigenetic status, can prevent SYT-SSX1 from exerting its effect on the *H19* ICR. On the other hand our data also suggest that the effect of SYT-SSX is not limited to methylation changes at the H19 ICR but rather affects additional, hitherto undefined, regulatory mechanisms at the *H19/IGF2* locus.

We have shown that introduction of SYT-SSX into different populations of hMSC has effects on epigenetic function that display cell-type specific qualitative and quantitative variation. We hypothesize that this variation could originate from the differences in the epigenetic context that the fusion protein encounters and that minor baseline epigenetic changes may have a relevant bearing on SYT-SSX function. It is possible that a highly specific epigenetic status is required for transformation of primary cells by SYT-SSX, which may explain, in part, the low frequency of SS. Such a permissive epigenetic status may be confined to cells at a specific stage of differentiation, as suggested by the recently reported transgenic mouse model.

## Materials and Methods

### Ethics Statement

Human mesenchymal stem cells were obtained from femoral head bone marrow of patients undergoing total hip replacement according to the guidelines of the Ethics Committee protocol 01–172 of the University of Geneva and after written informed consent of the patients.

### Cell Culture

Human mesenchymal stem cells were obtained as previously described [41], [42]. MSCs were cultured at low confluence in IMDM, 10% FCS, 10 ng/ml PDGF-BB (PeProtechEC, London,UK) and were tested for multilineage differentiation into adipocytes, chondrocytes and osteoblasts. Expression of mRNA encoding *Myf5* early myogenic differentiation marker was tested by real time PCR.

### Constructs and cDNA Cloning

cDNA clones, encoding the human *SYT-SSX1* fusion gene were amplified from surgically resected synovial sarcoma tissue by RT-PCR, using hSYTforward and hSSXreverse primers (without a stop codon). Tumor specimens were freshly frozen and stored at −80C until use. Total RNA was isolated from frozen tumors using Trizol reagent (Invitrogen, Carlsbad, CA) according to the manufacturer's recommendations and RT-PCR was performed using Super Script one step RT-PCR with the platinum Taq kit (Invitrogen) under the following cycling conditions: 1 cycle at 50°C for 30 min, and 94°C for 2 min followed by 30 cycles of 94°C for 1 min, 55°C. for 2 min, 72°C. for 3 min, and a final extension of 72°C for 10 min.

Amplified fragments were inserted, using the pEF6/V5-His TOPO TA expression kit (Invitrogen, Carlsbad, CA), into the corresponding expression vector in frame with the V5 epitope tag. This construct was used as a template for amplification of a SYT-SSX-V5 fragment with EcoRIhSYT and V5 reverse primers (including a stop codon); the resulting fragment was inserted into the pMSCV Neo retroviral expression vector into the EcoRI and the HpaI blunt site (BD Biosciences Clontech, Palo Alto, CA) and construct integrity was verified by sequencing.

### Retroviral Infection

Expression of SYT-SSX1-V5 in hMSCs was achieved using Retroviral Gene Transfer and Expression (BD Biosciences Clontech) according to the manufacturer's recommendations. Expression of the fusion genes and corresponding proteins was tested in all four batches of cells by real time-PCR and Western blot analysis using mouse anti-V5 antibody, respectively. Infected cells were selected with 0.5 µg/mL neomycin for 12 days and the bulk of the resistant cells was used in subsequent experiments.

### Western Blot

Cell lysis, SDS-PAGE and blotting, were done by standard procedures and protein bands were detected with a chemiluminescent substrate kit (Pierce) according to the manufacturer's recommendations. The antibodies used were: mouse anti–V5 monoclonal antibody (Invitrogen), anti–Histone H3 (Abcam), horseradish peroxidase–conjugated goat anti-mouse IgG (Amersham) and goat anti-rabbit IgG (Biorad).

### Real Time Quantitative RT-PCR

cDNA was obtained using an M-MLV reverse transcriptase and RNAse H minus (Promega). Typically 500 ng of template total RNA and 250 ng of random hexamers were used per reaction. Real time-PCR amplification was performed in an ABI Prism 7700 instrument (Applied Biosystems). For P1 promoter transcripts of *IGF2*, *H19* and cyclophilin, Taq Man Universal PCR mastermix and Assays-On-Demand Taq Man probes were used.

For real time quantitation of SYT-SSX RNA and for discrimination of *IGF2* transcripts, the Universal Probe Library system (Roche Rotkreuz, Switzerland) was used and primers were designed according to the ProbeFinder software (http://www.roche-applied-science.com).

Primers used were as follows: SYT-SSX1-181-1201Fw and SYT-SSX1-239-1257R were used with universal probelibrary 76. hIGF2exon8Fw and hIGF2exon9R were used with universal probelibrary 60 to test the expression of all *IGF2* transcripts including the *INS-IGF* and those derived from the activity of the P0 promoter.

hIGF2exon6Fw and hIGF2exon7R were used with universal probelibrary 63 to test the expression of P2–P4 derived *IGF2* transcripts. Taq Man probe Hs00171254_m1, spanning exons 1 and 2, was used to test *IGF2* expression driven by promoter P1.

Quantitation of target RNA, normalized with an endogenous control (Cyclophilin), was performed using the absolute quantitation method (Applied Biosystems).

Validation of microarray data relative to *BCL2, EPHA4*, *EPHA3* and *MYF5* expression was performed using both the Universal Probe Library system and sybergreen. Primers designed according to the ProbeFinder software and the corresponding probelibraries used are reported in the primers table (table S1). In these cases a comparative Ct method was used for the analysis.

### Affymetrix Microarrays and Bioinformatic Analysis

Total RNA was extracted using an RNeasy Mini Kit (QIAGEN, Valencia,CA) according to the manufacturer's recommendations. The quality of total RNA was verified by an Agilent RNA 600 nanoassay and by measuring the 260/280 absorbance ratio. The corresponding quality-tested total RNA was used by the Lausanne DNA Array Facility (DAFL) to perform gene expression profile analysis on Affymetrix HG-U133 Plus 2.0 Arrays, according to the manufacturer's recommendations (http://www.unil.ch/dafl/). Gene expression levels were obtained with RMA [68]using the Affymetrix Power Tools suite. Differential expression for the single batches was determined by requiring a fold-change greater than 2. Statistical analysis to determine differentially expressed genes across all the available batches was performed with Rank Products [69]: genes selected by rank products with a FDR of 1% or less and with mean fold change greater than 2 were retained for further analysis. Over representation of various functional categories, detailed below, was performed by first translating the lists of differentially coexpressed probesets into lists of Entrez Gene ids using the annotation files provided by the manufacturer (version na26), and then performing exact Fisher tests. Gene Ontology [45] annotations were obtained from Entrez Gene (data downloaded Oct. 26, 2008). We used version 20081012 of the Gene Ontology. The imprinted genes listed by Geneimprint (http://www.geneimprint.com) and the Catalogue of Imprinted Genes at the University of Otago [43] http://igc.otago.ac.nz were downloaded from the corresponding web sites on Dec. 12th and Nov. 11th 2008, respectively. For the Geneimprint database we separately considered all predicted imprinted genes and the experimentally confirmed ones only. Chromatin-associatedproteins were downloaded from the chromdb site [44] url on Nov. 11th, 2008. All gene symbols from these databases were converted into Entrez Gene ids using the gene_info file obtained from the Entrez Gene ftp site.

A list of CpG islands in the human genome (sequence version hg18) was obtained from the UCSC genome browser site url. We then used the TSS coordinates predicted by UCSC to determine which transcripts have their TSS lying in such CpG islands. This list was converted into a list of Entrez gene ids using the conversion table provided by UCSC.

Sarcoma signatures of reference [23] were obtained from the manuscript's supplementary material. Embryonic stem cell signatures were downloaded from the supplementary material of reference [51].

### Bisulfite Transformation of Genomic DNA

Genomic DNA from MSCs was isolated using a DNeasy Tissue Kit (Qiagen), and 200 ng of DNA were bisulfite modified using an EZ DNA Methylation Kit (Zymo Research) according to the manufacturer's instructions. About 1/5 of the eluted DNA was used as template for each PCR reaction.

### Cloning and Sequencing of Bisulfite-Treated DNA

Primers used for bisulfite-treated DNA amplification were designed using the primer design program MSPprimer (http://www.mspprimer.org/cgi-mspprimer/design.cgi). Primer sequence is reported in table S1.

Both MSPprimer and Methprimer (http://www.urogene.org/methprimer/index1.html) were used for in silico sequence analysis of bisulfite transformed DNA. The region amplified by primers BS-7712sense and BS-8192 antisense was located in the H19 ICR and included the sixth CTCF binding site. Twenty-six GpG sites were analyzed within this amplicon.

The region amplified by primers BS-13212sense and BS-13548antisense was included in a CpG island located about 3 kb downstream of the H19 TSS, and 15 CpG sites were analyzed within this amplicon.

Several PCR reactions for the amplification of the *H19* ICR or the *H19* gene downstream region from bisulfite treated DNA were performed and pooled before cloning.

To overcome bias in methylation analysis (we in fact observed preferential allelic amplification that was annealing temperature–dependent) [70], we used various annealing temperatures ranging from 55 to 60°C and employed both Go-taq (Promega) and the Fusion Taq polymerases (Finnzymes), to allow the use of maximal annealing temperature. In the latter case the obtained PCR fragments were purified and subsequently incubated at 70°C for 30 minutes in the presence of Go-Taq DNA polymerase buffer, 0.2 mM dATP and 5 units of Go-Taq DNA polymerase for subsequent TA cloning.

The following conditions were used for Go-Taq PCR: 95°C 5 minutes, 40 cycles 95°C 30 seconds, 55°C 45 seconds, 72°C 45 seconds and a final extension at 72°C for 7 minutes.

For Fusion Taq polymerase PCR, the conditions were as follows: 98°C 2 minutes, 40 cycles 98°C 20 seconds, 60°C 20 seconds, 72°C 45 seconds and a final extension at 72°C for 7 minutes. PCR products of the expected size were separated on 1.5% agarose gel and, after purification, were cloned into the pCR 4-TOPO vector using a TOPO TA Cloning Kit for sequencing (Invitrogen), and then transformed into Top10 E.Coli. For each sample the DNA of plasmid from 18 to more than 30 positive clones was sequenced for methylation analysis. Only clones with bisulfate conversion efficiency higher than 97% were taken into account.

### Allele Specific Methylation

In the region downstream of the human *H19* gene, the following polymorphic sites were considered (number refer to NCBI AF125183):

C/A at position 13359 (rs 217133) for which population 4 was heterozygous

C/G at position 13270 for which population 1 was heterozygous

In the *H19* ICR:

the G/A single nucleotide polymorphism at position 8097 for which populations 1, 3 and 4 were heterozygous.

Heterozygosity for specific polymorphic sites was determined either by direct sequencing or, when possible, by restriction fragment length polymorphism (RFLP) of specific PCR fragments. In the case of the G/A polymorphism at position 8097 a specific NlaIII restriction site was analyzed. A 481 bp fragment, spanning the NlaIII polymorphic site, was amplified by PCR from MSC genomic DNA using primers H19 7712F and H19 8192 R. After purification on a 1.5% agarose gel the fragment was incubated at 37°**C** for 3 hrs in the appropriate digestion buffer with or without 10 U of NlaIII. Fragment size analysis was performed on a 2% agarose gel.

Allelic specific methylation was determined by grouping single clones according to the relative sequence at the specific polymorphic sites.

### Allelic Expression Analysis of *IGF2*

Allelic differential expression was assessed by RT-PCR and subsequent restriction fragment length polymorphism (RFLP). A known C/T single nucleotide polymorphism (rs2230949) at position 114671 of the human *IGF2* gene (NCBI AC132217), for which populations 4 and 1 were informative, was analyzed. A semi-quantitative RT-PCR reaction was performed using superscript one step RT-PCR platinum Taq kit (Invitrogen) and intron crossing primers Exon8 F and NarI R. In pilot experiments we established conditions such that none of the RNAs analyzed reached a plateau at the end of the amplification protocol.

After separation on a 1.5% gel the 487 bp fragments, exclusively derived from RNA, were purified and either directly used for restriction fragment length polymorphism (RFLP) analysis or for nested semi-quantitative PCR. Digestion of 487 bp fragments containing two polymorphic NarI sites produced 244/243 bp fragments from one allele and 305 and 182 bp fragments from the other. The expected heteroduplexes formed during the PCR reaction remained undigested. Nested PCR was performed using primers NarI F and NarI R spanning the polymorphic site, and the 237 bp fragments obtained were used for RFLP analysis. Fragments were incubated at 37°C for 3 hrs in the appropriate digestion buffer with or without 8 U of NarI (NEB). Fragment size analysis was performed on a 2% agarose gel. Digestion produced a 182 bp fragment from one allele and the undigested form from the other, or the heteroduplex. Digestion efficiency was controlled by parallel digestion of similar fragments obtained from homozygous samples.

## Supporting Information

Figure S1 hMSCs differentiation into adipogenic, osteogenic, and chondrogenic lineages upon stimulation with the appropriate cytokines. (see Materials and Methods for details). Adipocytic differentiation, oil Red-O staining; osteoblastic differentiation, von Kossa staining; and chondrocytic differentiation, anti-collagen type II labeling counterstained with hematoxylin. Magnification: adipocytes and osteocytes, _200; chondrocytes, _100.
(1.55 MB TIF)

Figure S2 Differential Igf2 allelic expression in human mesenchymal stem cell population 1 and 4. Allelic differential expression was investigated by RT-PCR and subsequent restriction fragment length polymorphism (RFLP). A known C/T single nucleotide polymorphism at position 114671 of the human Igf2 gene (NCBI AC132217) (rs2230949) for which these populations were informative, was analyzed. Genomic DNA and RNA were extracted from hMSC populations 1 and 4. A 237 bp fragment spanning the C/T NarI polymorphic site was amplified using NarI forward and NarI reverse primers from the DNA template. Upon NarI digestion, heterozygous profile at this position produces fragments of 182 bp (visible on gel) and 55 bp (not visible on gel) (from the allele carryng C) and an undigested fragment (from the allele carryng T). A RNA specific 487 bp fragment, spanning the Igf2 C/T polymorphism was amplified by quantitative RT-PCR from RNA extracted from hMSC populations 1 and 4, using cross intron primers Ex8 forward and NarI reverse. The 487 bp bands were separated on a 1.5% agarose gel and, after purification, used as a template for a nested quantitative PCR using primers NarI forward and NarI reverse that produce the 237 bp amplicon. DNA fragments were incubated at 37°C for 3 hrs in the appropriate digestion buffer with or without 4,000 U of NarI. Fragment size analysis was performed on a 2% agarose gel. Lambda DNA/BstE II Digest marker were used, fragment size is indicated.
(0.07 MB TIF)

Figure S3 Human mesenchymal stem cell populations genotype analysis. Heterozygosity for the G/A singol nucleotide polymorphism at position 8097 of the human H19 gene (NCBI AF125183) was analysed by restriction fragment length polymorphism (RFLP) and confirmed by direct sequencing of the PCR fragments. Numbers are all referred to NCBI AF125183 A) Schematic representation showing the position of the 4 NlaIII restriction sites in the H19 gene fragment from 7712 to 8192. The polymorphic NlaIII site at position 8097 is indicated. Heterozygous profile at this position produces 2 fragments of 215 bp (from the allele carryng G) and 296 bp (from the allele carryng A) in addition to 81, 87 and 17 bp common fragments. B) NlaIII restriction digestion profiling of the H19 gene fragment 7712–8192 obtained from 3 different Human mesenchymal stem cell populations. A 481 bp fragment, spanning the NlaIII polymorphic site, was amplified by PCR from MSCs genomic DNA. After gel purification on a 1.5% agarose gel the fragment was incubated at 37°C for 3 hrs in the appropriate digestion buffer with or without 10,000 U of NlaIII. Fragment size analysis was performed on a 2% agarose gel, first lane of each gel shows Lambda DNA/BstE II Digest marker, fragment size is indicated. C) DNA sequence analysis of the same PCR products. Double G/A pick shows heterozygosity.
(1.20 MB TIF)

Table S1 List of primers used.
(0.03 MB DOC)

Table S2 List of probesets differentially expressed in human MSCs expressing SYT-SSX1 or an empty vector, 12 days after the infection and puromycin selection. A) Lists of the probesets that are differentially expressed with an FDR of 1% and a mean fold change >2. The four human MSCs batches were analyzed together with rank products (MSCs rankproduct). B) Lists of the probesets that are differentially expressed with a fold-change greater than 2 in each individual batch of human MSCs (MSCs b1–b4).
(0.49 MB XLS)

Table S3 List of Gene Ontology (GO) terms over-represented in the single-batch lists (MSCs batch 1-batch 4) and the lists derived from the rank-product analysis (MSCs rankproduct).
(1.74 MB HTM)

Table S4 Overlap of the lists of differentially expressed genes in the single-batch lists (MSCs b1–b4) and the lists derived from the rank-product analysis (MSCs rankproduct) with the databases of imprinted genes (Geneimprint and Otago).
(0.07 MB XLS)

Table S5 Overlap of the lists of differentially expressed genes in the single-batch lists (MSCs b1–b4) and the lists derived from the rank-product analysis (MSCs rankproduct) with the genes whose TSS lies within a CpG island.
(0.76 MB XLS)

Table S6 Overlap of the lists of differentially expressed genes in the single-batch lists (MSCs b1–b4) and the lists derived from the rank-product analysis (MSCs rankproduct) with the databases of chromatin-related genes (ChromoDB)
(0.05 MB XLS)

Table S7 Overlap of the lists of differentially expressed genes in the single-batch (MSCs b1–b4) and in the lists derived from the rank-product analysis (MSCs rankproduct) with the lists of various recently published stemness markers.
(0.77 MB XLS)

Table S8 Overlap of the lists of differentially expressed genes in the single-batch lists (MSCs b1–b4) and the lists derived from the rank-product analysis (MSCs rankproduct) with the sarcoma signatures identified in Francis et al, 2007, BMC Genomics 8: 73.
(0.88 MB XLS)
