# Supplementary figures and images for: Epigenetic Features of Human Mesenchymal Stem Cells Determine Their Permissiveness for Induction of Relevant Transcriptional Changes by SYT-SSX1
Source: PLoS One. 2009 Nov 19;4(11):e7904. doi: 10.1371/journal.pone.0007904 (PMC2775947; doi:10.1371/journal.pone.0007904)

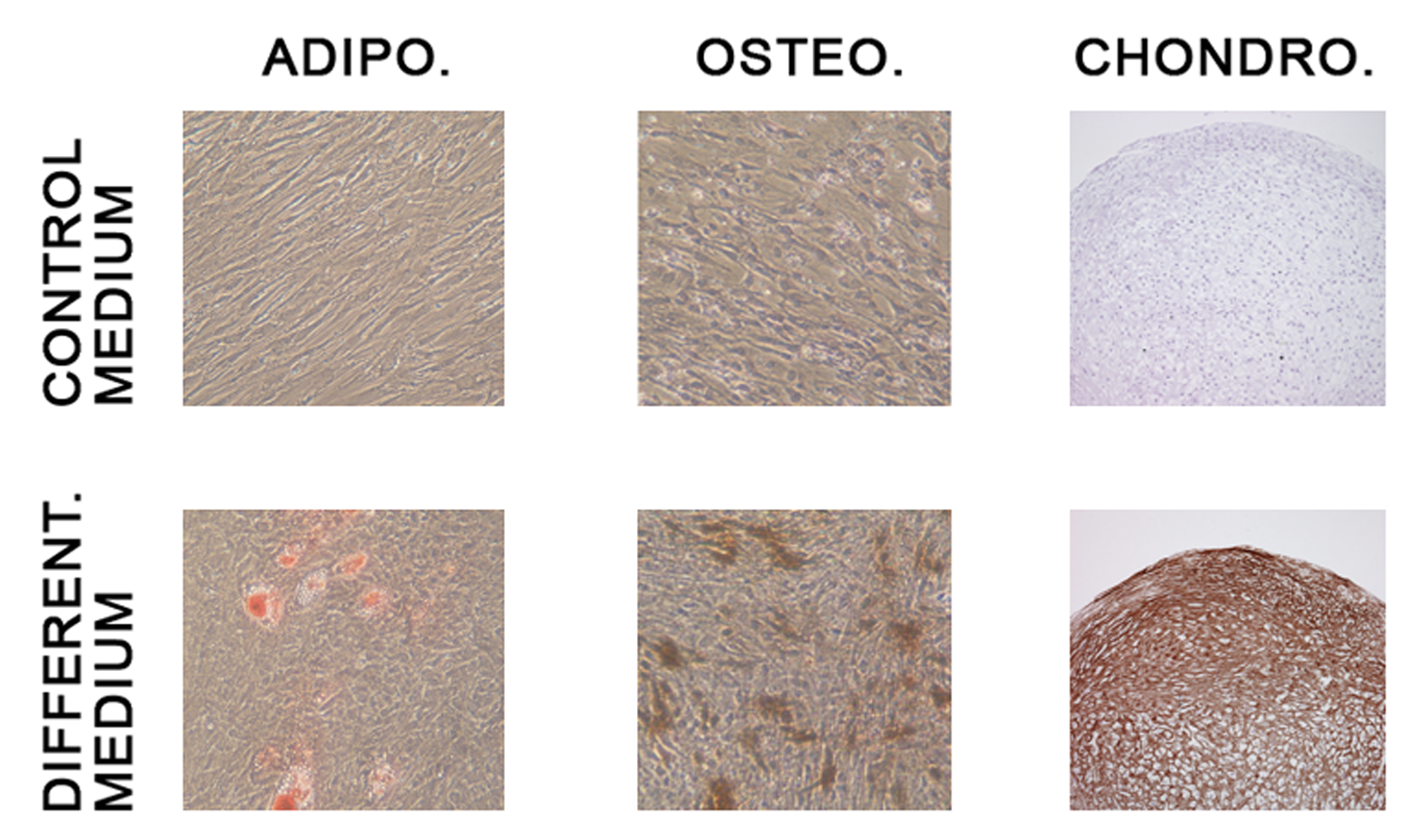

Supplement: Figure S1 — hMSCs differentiation into adipogenic, osteogenic, and chondrogenic lineages upon stimulation with the appropriate cytokines. (see Materials and Methods for details). Adipocytic differentiation, oil Red-O staining; osteoblastic differentiation, von Kossa staining; and chondrocytic differentiation, anti-collagen type II labeling counterstained with hematoxylin. Magnification: adipocytes and osteocytes, _200; chondrocytes, _100. (1.55 MB TIF) [file pone.0007904.s001.tif]

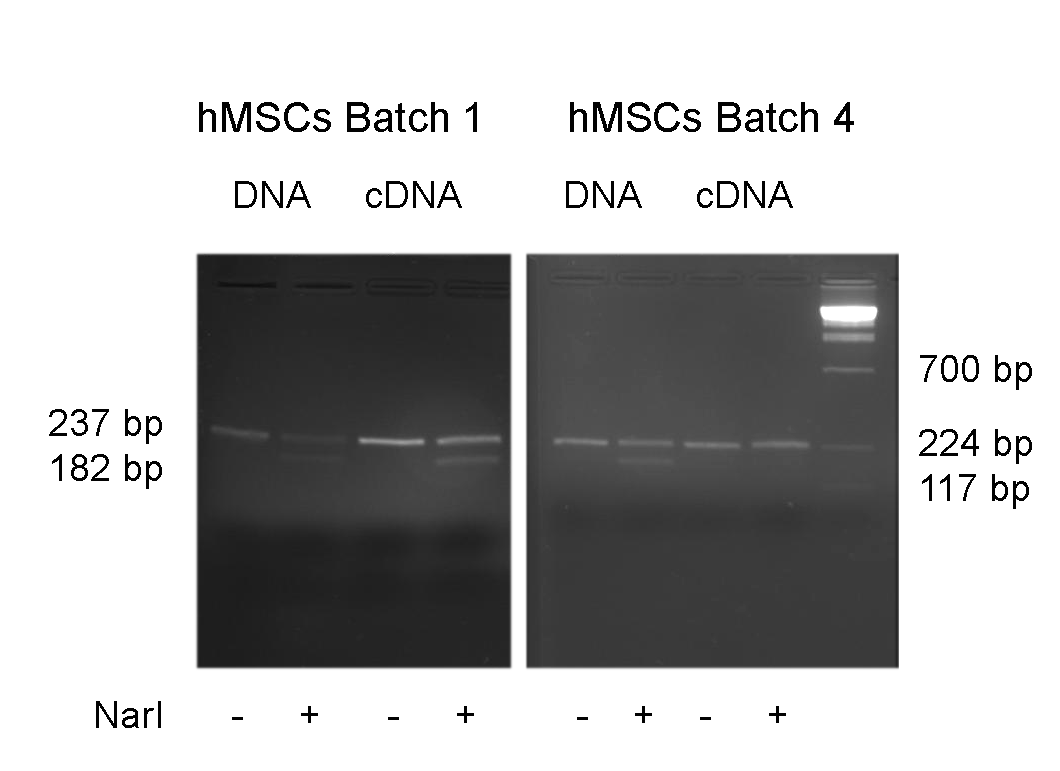

Supplement: Figure S2 — Differential Igf2 allelic expression in human mesenchymal stem cell population 1 and 4. Allelic differential expression was investigated by RT-PCR and subsequent restriction fragment length polymorphism (RFLP). A known C/T single nucleotide polymorphism at position 114671 of the human Igf2 gene (NCBI AC132217) (rs2230949) for which these populations were informative, was analyzed. Genomic DNA and RNA were extracted from hMSC populations 1 and 4. A 237 bp fragment spanning the C/T NarI polymorphic site was amplified using NarI forward and NarI reverse primers from the DNA template. Upon NarI digestion, heterozygous profile at this position produces fragments of 182 bp (visible on gel) and 55 bp (not visible on gel) (from the allele carryng C) and an undigested fragment (from the allele carryng T). A RNA specific 487 bp fragment, spanning the Igf2 C/T polymorphism was amplified by quantitative RT-PCR from RNA extracted from hMSC populations 1 and 4, using cross intron primers Ex8 forward and NarI reverse. The 487 bp bands were separated on a 1.5% agarose gel and, after purification, used as a template for a nested quantitative PCR using primers NarI forward and NarI reverse that produce the 237 bp amplicon. DNA fragments were incubated at 37°C for 3 hrs in the appropriate digestion buffer with or without 4,000 U of NarI. Fragment size analysis was performed on a 2% agarose gel. Lambda DNA/BstE II Digest marker were used, fragment size is indicated. (0.07 MB TIF) [file pone.0007904.s002.tif]

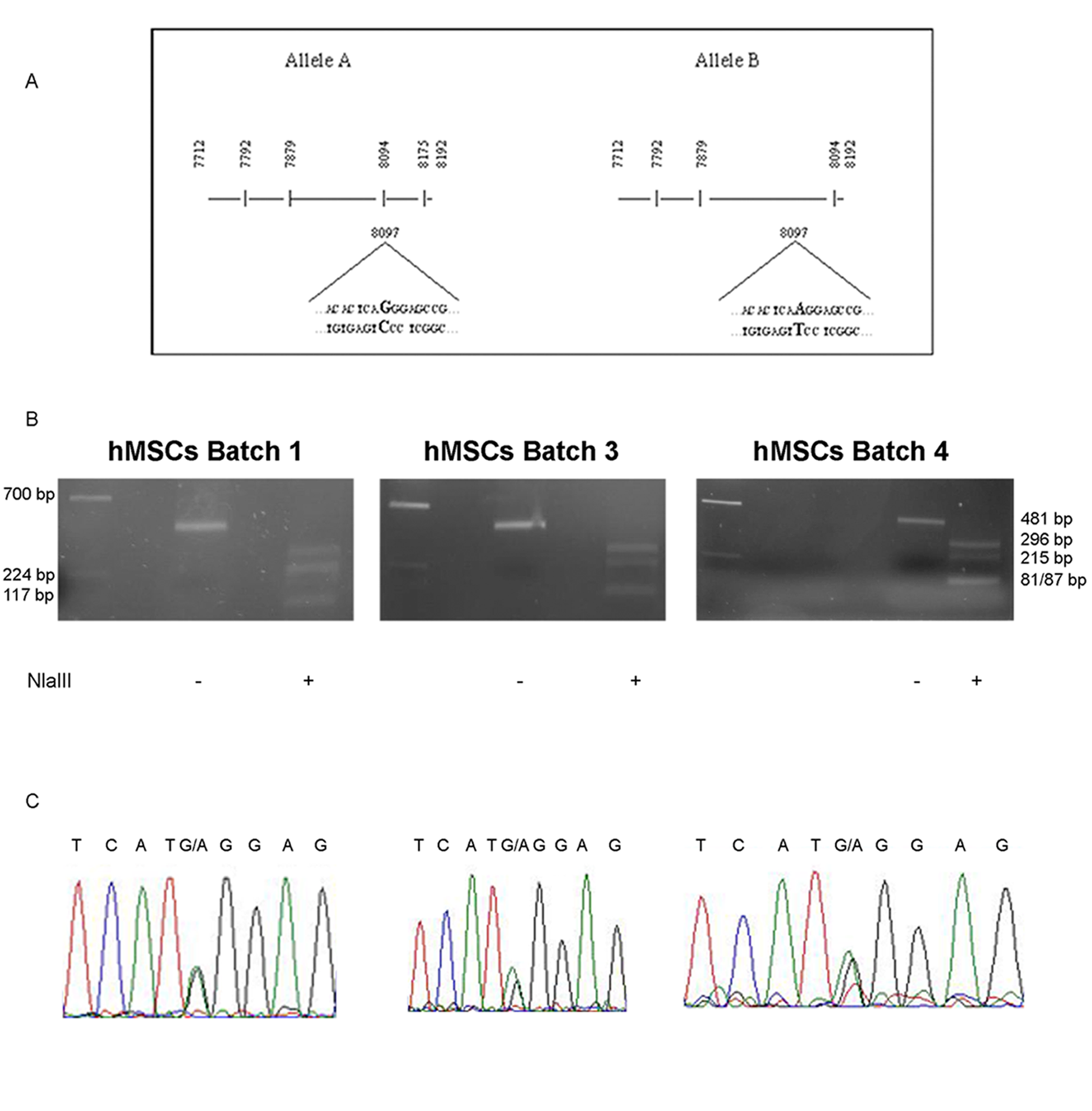

Supplement: Figure S3 — Human mesenchymal stem cell populations genotype analysis. Heterozygosity for the G/A singol nucleotide polymorphism at position 8097 of the human H19 gene (NCBI AF125183) was analysed by restriction fragment length polymorphism (RFLP) and confirmed by direct sequencing of the PCR fragments. Numbers are all referred to NCBI AF125183 A) Schematic representation showing the position of the 4 NlaIII restriction sites in the H19 gene fragment from 7712 to 8192. The polymorphic NlaIII site at position 8097 is indicated. Heterozygous profile at this position produces 2 fragments of 215 bp (from the allele carryng G) and 296 bp (from the allele carryng A) in addition to 81, 87 and 17 bp common fragments. B) NlaIII restriction digestion profiling of the H19 gene fragment 7712–8192 obtained from 3 different Human mesenchymal stem cell populations. A 481 bp fragment, spanning the NlaIII polymorphic site, was amplified by PCR from MSCs genomic DNA. After gel purification on a 1.5% agarose gel the fragment was incubated at 37°C for 3 hrs in the appropriate digestion buffer with or without 10,000 U of NlaIII. Fragment size analysis was performed on a 2% agarose gel, first lane of each gel shows Lambda DNA/BstE II Digest marker, fragment size is indicated. C) DNA sequence analysis of the same PCR products. Double G/A pick shows heterozygosity. (1.20 MB TIF) [file pone.0007904.s003.tif]
