# Supplementary material for: Epigenetic Features of Human Mesenchymal Stem Cells Determine Their Permissiveness for Induction of Relevant Transcriptional Changes by SYT-SSX1
Source: PLoS One. 2009 Nov 19;4(11):e7904. doi: 10.1371/journal.pone.0007904 (PMC2775947; doi:10.1371/journal.pone.0007904)
Supplement: Table S1 — List of primers used. (0.03 MB DOC) [file pone.0007904.s004.doc]

**LIST OF PRIMERS USED.**

| Bisulfite transformation |  |
| --- | --- |
| BS-7712sense | 5’TTTGATTTATTTTAGGGTGTATTGTTGAA3’ |
| BS-8192antisense | 5’CTTAAATCCCAAACCATAACACTA3’ |
| BS-13212sense | 5’TCTAAAAAACCCACAAAATACTCT3’ |
| BS-13548antisense | 5’GTTTTTGGAGTTTTAGTAGTAAGATTT3’ |
|  |  |
|  |  |
| Allelic expression and SNP check |  |
| NarI R | 5’GAAGATGCTGCTGTGCTTCCTCAG3’ |
| NarI Fw | 5’CCCAGAGATGGCCAGCAATCGGAAGT3’ |
| H19- 7712Fw | 5’TCTGATTCACCCCAGGGTGCACTGT3’ |
| H19- 8192R | 5’CTTGAGTCCCAGGCCATGACACTGAA3’ |
| H19-7565Fw | 5’GCAGTGCAGGCTCACACATCACAGCCTGAG3’ |
| H19-8298R | 5’ATTTGGGGGCTGTCCTTAGACGGAGT3’ |
| H19-7895R | 5’CTCCAGAAATACCCATGTGCTATGCAAGAG3’ |
|  |  |
| Expression, nicroarrays validation. |  |
| hIgf2 exon8Fw (problib. 60) | 5’ AAGTCCGAGAGGGACGTGT 3’ |
| hIgf2 exon 9R (problib. 60) | 5’ TCCAGGTGTCATATTGGAAGAAC 3’ |
| hIgf2 exon 6Fw (problib. 63) | 5’ TCTCCTGTGAAAGAGACTTCCAG 3’ |
| hIgf2 exon 7R (problib. 63) | 5’ GGGATTCCCATTGGTGTCT 3’ |
| SYT-SSX1181-1201Fw (problib. 76) | 5’ATAGACCAACACAGCCTGGAC 3’ |
| SYT-SSX1239-1257R (problib. 76) | 5’CTTCTTGGGCATGATCTGG 3’ |
| Bcl2Fw (problib. 6) | 5’TTGAGAGAGGATCATGCTGTACTT 3’ |
| Bcl2R (problib. 6) | 5’ATCTTTATTTCATGAGGCACGTT 3’ |
| EPHA4Fw (problib. 1) | 5’AAGAGGACAGGGACGGAGAG 3’ |
| EPHA4R (problib. 1) | 5’AGTTATCCTTATACCGGTCCATTTT3’ |
| EPHA3Fw (problib. 63) | 5’CATGGATTGTCAGCTCTCCA 3’ |
| EPHA3R (problib. 63) | 5’TGTTTTTGAATCCAGTAGATTGACTT 3’ |
| Mif5Fw | 5’ CTATAGCCTGCCGGGACA 3’ |
| Mif5R | 5’TGGACCAGACAGGACTGTTACAT 3’ |
|  |  |
| Cloning |  |
| hSYTforward | 5’ATGGGCGGCAACATGTCTGTGGC 3’ |
| hSSXreverse (without stop codon) | 5’CTCGTCATCTTCCTCAGGGTCACT 3’. |
| EcoRIhSYT | 5’GGAATTCCATGGGCGGCAACATGTCTGTG 3’ |
| V5 reverse | 5’TCACGTAGAATCGAGACCGAGGAGAGGGTTAGGGATAGGCTT ACC 3’ |
|  |  |
