# Supplementary material for: Epigenetic Features of Human Mesenchymal Stem Cells Determine Their Permissiveness for Induction of Relevant Transcriptional Changes by SYT-SSX1
Source: PLoS One. 2009 Nov 19;4(11):e7904. doi: 10.1371/journal.pone.0007904 (PMC2775947; doi:10.1371/journal.pone.0007904)
Supplement: Table S3 — List of Gene Ontology (GO) terms over-represented in the single-batch lists (MSCs batch 1-batch 4) and the lists derived from the rank-product analysis (MSCs rankproduct). (1.74 MB HTM) [file pone.0007904.s006.html]

# **Supplemental table S3**

  

## List of Gene Ontology (GO) terms over-represented in the single-batch analysis (MSCs batch1-batch4) and in the lists derived from the rank products analysis (MSCs rank products).

  

## MSCs batch 1 repressed

|  |  |  |  |  |  |
| --- | --- | --- | --- | --- | --- |
| id | name | genes | expect | P | adj P |
| GO:0007067 | mitosis : biological\_process | 26 | 5.61453 | 8.42571e-11 | 9.80853e-06 |
| GO:0000087 | M phase of mitotic cell cycle : biological\_process | 26 | 5.66878 | 1.04558e-10 | 1.21718e-05 |
| GO:0005975 | carbohydrate metabolic process : biological\_process | 38 | 11.663 | 1.82828e-10 | 2.12834e-05 |
| GO:0044420 | extracellular matrix part : cellular\_component | 16 | 2.33261 | 1.15636e-09 | 0.000134614 |
| GO:0022403 | cell cycle phase : biological\_process | 33 | 9.79153 | 1.26238e-09 | 0.000146956 |
| GO:0044262 | cellular carbohydrate metabolic process : biological\_process | 29 | 7.81152 | 1.42094e-09 | 0.000165415 |
| GO:0000278 | mitotic cell cycle : biological\_process | 33 | 9.95427 | 1.91723e-09 | 0.000223188 |
| GO:0051301 | cell division : biological\_process | 25 | 6.37399 | 6.41684e-09 | 0.000746997 |
| GO:0000279 | M phase : biological\_process | 28 | 7.86577 | 6.97067e-09 | 0.00081147 |
| GO:0022402 | cell cycle process : biological\_process | 35 | 13.2091 | 1.82919e-07 | 0.021294 |
| GO:0006091 | generation of precursor metabolites and energy : biological\_process | 25 | 7.62166 | 2.19383e-07 | 0.0255388 |
| GO:0005856 | cytoskeleton : cellular\_component | 62 | 31.436 | 2.47573e-07 | 0.0288204 |
| GO:0019318 | hexose metabolic process : biological\_process | 17 | 3.87864 | 3.52892e-07 | 0.0410808 |
| GO:0007049 | cell cycle : biological\_process | 48 | 22.214 | 4.88295e-07 | 0.0568434 |
| GO:0006007 | glucose catabolic process : biological\_process | 11 | 1.6274 | 5.43991e-07 | 0.0633271 |
| GO:0005996 | monosaccharide metabolic process : biological\_process | 17 | 4.01425 | 5.78354e-07 | 0.0673273 |
| GO:0005576 | extracellular region : cellular\_component | 82 | 47.3031 | 5.91648e-07 | 0.0688749 |
| GO:0005819 | spindle : cellular\_component | 13 | 2.35973 | 6.09523e-07 | 0.0709558 |
| GO:0006096 | glycolysis : biological\_process | 10 | 1.35617 | 7.68637e-07 | 0.0894785 |
| GO:0031012 | extracellular matrix : cellular\_component | 25 | 8.16413 | 7.96737e-07 | 0.0927497 |
| GO:0007346 | regulation of mitotic cell cycle : biological\_process | 14 | 2.84795 | 9.4254e-07 | 0.109723 |
| GO:0000777 | condensed chromosome kinetochore : cellular\_component | 9 | 1.08493 | 9.61085e-07 | 0.111882 |
| GO:0006006 | glucose metabolic process : biological\_process | 14 | 2.9022 | 1.18899e-06 | 0.138412 |
| GO:0000779 | condensed chromosome, centromeric region : cellular\_component | 9 | 1.11206 | 1.20201e-06 | 0.139929 |
| GO:0005578 | proteinaceous extracellular matrix : cellular\_component | 24 | 7.81152 | 1.24583e-06 | 0.145029 |
| GO:0008652 | amino acid biosynthetic process : biological\_process | 9 | 1.13918 | 1.49334e-06 | 0.173843 |
| GO:0019320 | hexose catabolic process : biological\_process | 11 | 1.87151 | 2.32247e-06 | 0.270364 |
| GO:0004720 | protein-lysine 6-oxidase activity : molecular\_function | 4 | 0.135617 | 2.61968e-06 | 0.304962 |
| GO:0046365 | monosaccharide catabolic process : biological\_process | 11 | 1.89863 | 2.68888e-06 | 0.313018 |
| GO:0044430 | cytoskeletal part : cellular\_component | 42 | 19.5017 | 2.82694e-06 | 0.32909 |
| GO:0032395 | MHC class II receptor activity : molecular\_function | 5 | 0.271233 | 3.24524e-06 | 0.377785 |
| GO:0046164 | alcohol catabolic process : biological\_process | 11 | 2.00713 | 4.71001e-06 | 0.548302 |
| GO:0044421 | extracellular region part : cellular\_component | 42 | 20.0441 | 5.58369e-06 | 0.650009 |
| GO:0042613 | MHC class II protein complex : cellular\_component | 5 | 0.298357 | 5.81674e-06 | 0.677138 |
| GO:0007155 | cell adhesion : biological\_process | 41 | 19.5559 | 7.13873e-06 | 0.831034 |
| GO:0022610 | biological adhesion : biological\_process | 41 | 19.5559 | 7.13873e-06 | 0.831034 |
| GO:0008305 | integrin complex : cellular\_component | 7 | 0.759454 | 7.49842e-06 | 0.872906 |
| GO:0005198 | structural molecule activity : molecular\_function | 38 | 17.5759 | 7.77226e-06 | 0.904784 |
| GO:0044275 | cellular carbohydrate catabolic process : biological\_process | 12 | 2.52247 | 7.94351e-06 | 0.92472 |
| GO:0015630 | microtubule cytoskeleton : cellular\_component | 30 | 12.5039 | 1.00837e-05 | 1 |
| GO:0000776 | kinetochore : cellular\_component | 9 | 1.43754 | 1.1385e-05 | 1 |
| GO:0005201 | extracellular matrix structural constituent : molecular\_function | 11 | 2.22411 | 1.29777e-05 | 1 |
| GO:0045787 | positive regulation of cell cycle : biological\_process | 7 | 0.840824 | 1.55179e-05 | 1 |
| GO:0031577 | spindle checkpoint : biological\_process | 4 | 0.189863 | 1.75571e-05 | 1 |
| GO:0007229 | integrin-mediated signaling pathway : biological\_process | 9 | 1.51891 | 1.81074e-05 | 1 |
| GO:0000775 | chromosome, centromeric region : cellular\_component | 11 | 2.30548 | 1.83777e-05 | 1 |
| GO:0016052 | carbohydrate catabolic process : biological\_process | 12 | 2.76658 | 2.06565e-05 | 1 |
| GO:0016051 | carbohydrate biosynthetic process : biological\_process | 12 | 2.7937 | 2.28142e-05 | 1 |
| GO:0002504 | antigen processing and presentation of peptide or polysaccharide antigen via MHC class II : biological\_process | 5 | 0.379727 | 2.35566e-05 | 1 |
| GO:0048513 | organ development : biological\_process | 60 | 34.745 | 2.53361e-05 | 1 |
| GO:0000226 | microtubule cytoskeleton organization and biogenesis : biological\_process | 12 | 2.84795 | 2.77247e-05 | 1 |
| GO:0006563 | L-serine metabolic process : biological\_process | 4 | 0.216987 | 3.43597e-05 | 1 |
| GO:0045840 | positive regulation of mitosis : biological\_process | 5 | 0.40685 | 3.45478e-05 | 1 |
| GO:0034637 | cellular carbohydrate biosynthetic process : biological\_process | 11 | 2.49535 | 3.90364e-05 | 1 |
| GO:0051726 | regulation of cell cycle : biological\_process | 22 | 8.5981 | 5.94159e-05 | 1 |
| GO:0044271 | nitrogen compound biosynthetic process : biological\_process | 11 | 2.65809 | 7.03091e-05 | 1 |
| GO:0005604 | basement membrane : cellular\_component | 8 | 1.41041 | 7.32348e-05 | 1 |
| GO:0055114 | oxidation reduction : biological\_process | 32 | 15.4061 | 8.66029e-05 | 1 |
| GO:0007051 | spindle organization and biogenesis : biological\_process | 6 | 0.759454 | 8.77526e-05 | 1 |
| GO:0009309 | amine biosynthetic process : biological\_process | 9 | 1.84439 | 8.82936e-05 | 1 |

## MSCs batch 1 induced

|  |  |  |  |  |  |
| --- | --- | --- | --- | --- | --- |
| id | name | genes | expect | P | adj P |
| GO:0005125 | cytokine activity : molecular\_function | 16 | 4.7258 | 2.34948e-05 | 1 |
| GO:0045595 | regulation of cell differentiation : biological\_process | 14 | 4.22491 | 9.48351e-05 | 1 |

## MSCs batch 2 repressed

|  |  |  |  |  |  |
| --- | --- | --- | --- | --- | --- |
| id | name | genes | expect | P | adj P |
| GO:0007067 | mitosis : biological\_process | 52 | 2.56137 | 1.51795e-53 | 1.76708e-48 |
| GO:0000087 | M phase of mitotic cell cycle : biological\_process | 52 | 2.58612 | 2.64222e-53 | 3.07586e-48 |
| GO:0000279 | M phase : biological\_process | 57 | 3.5884 | 3.32926e-52 | 3.87566e-47 |
| GO:0022403 | cell cycle phase : biological\_process | 59 | 4.46694 | 3.91581e-49 | 4.55848e-44 |
| GO:0000278 | mitotic cell cycle : biological\_process | 57 | 4.54118 | 3.98118e-46 | 4.63457e-41 |
| GO:0051301 | cell division : biological\_process | 49 | 2.90784 | 4.26042e-46 | 4.95964e-41 |
| GO:0022402 | cell cycle process : biological\_process | 62 | 6.02603 | 6.7434e-45 | 7.85013e-40 |
| GO:0007049 | cell cycle : biological\_process | 74 | 10.1341 | 2.5188e-43 | 2.93219e-38 |
| GO:0005694 | chromosome : cellular\_component | 43 | 4.44219 | 7.71321e-30 | 8.9791e-25 |
| GO:0044427 | chromosomal part : cellular\_component | 40 | 3.69976 | 1.143e-29 | 1.33059e-24 |
| GO:0000775 | chromosome, centromeric region : cellular\_component | 25 | 1.05177 | 6.88323e-28 | 8.01291e-23 |
| GO:0005819 | spindle : cellular\_component | 25 | 1.07652 | 1.33262e-27 | 1.55134e-22 |
| GO:0000777 | condensed chromosome kinetochore : cellular\_component | 17 | 0.494951 | 1.48737e-22 | 1.73148e-17 |
| GO:0015630 | microtubule cytoskeleton : cellular\_component | 40 | 5.70432 | 1.9056e-22 | 2.21835e-17 |
| GO:0000779 | condensed chromosome, centromeric region : cellular\_component | 17 | 0.507325 | 2.51322e-22 | 2.92569e-17 |
| GO:0006259 | DNA metabolic process : biological\_process | 40 | 5.92704 | 7.82332e-22 | 9.10729e-17 |
| GO:0000776 | kinetochore : cellular\_component | 18 | 0.655811 | 1.09651e-21 | 1.27647e-16 |
| GO:0006260 | DNA replication : biological\_process | 28 | 2.68511 | 1.52257e-20 | 1.77245e-15 |
| GO:0000793 | condensed chromosome : cellular\_component | 19 | 1.10127 | 1.50734e-18 | 1.75472e-13 |
| GO:0007346 | regulation of mitotic cell cycle : biological\_process | 20 | 1.29925 | 2.04183e-18 | 2.37694e-13 |
| GO:0044430 | cytoskeletal part : cellular\_component | 43 | 8.89675 | 6.73747e-18 | 7.84322e-13 |
| GO:0007059 | chromosome segregation : biological\_process | 16 | 0.791922 | 5.36904e-17 | 6.25021e-12 |
| GO:0007051 | spindle organization and biogenesis : biological\_process | 12 | 0.346466 | 2.5245e-16 | 2.93882e-11 |
| GO:0007017 | microtubule-based process : biological\_process | 24 | 2.85834 | 1.42104e-15 | 1.65426e-10 |
| GO:0051726 | regulation of cell cycle : biological\_process | 27 | 3.92249 | 3.26139e-15 | 3.79664e-10 |
| GO:0005856 | cytoskeleton : cellular\_component | 50 | 14.3412 | 6.22832e-15 | 7.25051e-10 |
| GO:0000070 | mitotic sister chromatid segregation : biological\_process | 11 | 0.383587 | 5.68615e-14 | 6.61936e-09 |
| GO:0043234 | protein complex : cellular\_component | 60 | 21.0602 | 6.39835e-14 | 7.44845e-09 |
| GO:0000819 | sister chromatid segregation : biological\_process | 11 | 0.395961 | 8.57067e-14 | 9.97728e-09 |
| GO:0000075 | cell cycle checkpoint : biological\_process | 13 | 0.767175 | 5.65917e-13 | 6.58795e-08 |
| GO:0031981 | nuclear lumen : cellular\_component | 46 | 13.9205 | 6.16398e-13 | 7.17562e-08 |
| GO:0005654 | nucleoplasm : cellular\_component | 35 | 8.51317 | 1.23679e-12 | 1.43978e-07 |
| GO:0000226 | microtubule cytoskeleton organization and biogenesis : biological\_process | 15 | 1.29925 | 3.3144e-12 | 3.85836e-07 |
| GO:0044428 | nuclear part : cellular\_component | 52 | 18.2018 | 4.03015e-12 | 4.69158e-07 |
| GO:0006261 | DNA-dependent DNA replication : biological\_process | 13 | 0.903286 | 5.20938e-12 | 6.06434e-07 |
| GO:0010564 | regulation of cell cycle process : biological\_process | 12 | 0.754801 | 1.0078e-11 | 1.1732e-06 |
| GO:0006974 | response to DNA damage stimulus : biological\_process | 23 | 3.93486 | 1.18848e-11 | 1.38353e-06 |
| GO:0005874 | microtubule : cellular\_component | 20 | 2.90784 | 1.47229e-11 | 1.71393e-06 |
| GO:0043233 | organelle lumen : cellular\_component | 49 | 17.6203 | 4.95298e-11 | 5.76586e-06 |
| GO:0007088 | regulation of mitosis : biological\_process | 10 | 0.507325 | 5.62741e-11 | 6.55098e-06 |
| GO:0005524 | ATP binding : molecular\_function | 47 | 16.519 | 6.16171e-11 | 7.17297e-06 |
| GO:0070013 | intracellular organelle lumen : cellular\_component | 48 | 17.2243 | 7.55935e-11 | 8.79999e-06 |
| GO:0032559 | adenyl ribonucleotide binding : molecular\_function | 47 | 16.7417 | 9.68011e-11 | 1.12688e-05 |
| GO:0031974 | membrane-enclosed lumen : cellular\_component | 49 | 18.0286 | 1.09899e-10 | 1.27936e-05 |
| GO:0051276 | chromosome organization : biological\_process | 23 | 4.54118 | 2.10004e-10 | 2.4447e-05 |
| GO:0007093 | mitotic cell cycle checkpoint : biological\_process | 9 | 0.420709 | 2.35856e-10 | 2.74564e-05 |
| GO:0051716 | cellular response to stimulus : biological\_process | 22 | 4.30608 | 4.48624e-10 | 5.22252e-05 |
| GO:0034984 | cellular response to DNA damage stimulus : biological\_process | 20 | 3.5389 | 4.97885e-10 | 5.79597e-05 |
| GO:0030554 | adenyl nucleotide binding : molecular\_function | 47 | 17.6079 | 5.17068e-10 | 6.0193e-05 |
| GO:0000922 | spindle pole : cellular\_component | 8 | 0.321718 | 6.33288e-10 | 7.37223e-05 |
| GO:0033554 | cellular response to stress : biological\_process | 21 | 4.1081 | 1.12459e-09 | 0.000130915 |
| GO:0005876 | spindle microtubule : cellular\_component | 7 | 0.247476 | 2.75979e-09 | 0.000321273 |
| GO:0032555 | purine ribonucleotide binding : molecular\_function | 50 | 20.5529 | 3.06094e-09 | 0.000356331 |
| GO:0032553 | ribonucleotide binding : molecular\_function | 50 | 20.5529 | 3.06094e-09 | 0.000356331 |
| GO:0006281 | DNA repair : biological\_process | 18 | 3.16769 | 3.45937e-09 | 0.000402712 |
| GO:0003777 | microtubule motor activity : molecular\_function | 11 | 0.977529 | 3.59778e-09 | 0.000418825 |
| GO:0007018 | microtubule-based movement : biological\_process | 12 | 1.23738 | 3.96896e-09 | 0.000462035 |
| GO:0031577 | spindle checkpoint : biological\_process | 5 | 0.0866165 | 5.73651e-09 | 0.000667798 |
| GO:0030261 | chromosome condensation : biological\_process | 7 | 0.284597 | 8.45557e-09 | 0.000984329 |
| GO:0006270 | DNA replication initiation : biological\_process | 7 | 0.284597 | 8.45557e-09 | 0.000984329 |
| GO:0017076 | purine nucleotide binding : molecular\_function | 50 | 21.4438 | 1.2359e-08 | 0.00143873 |
| GO:0051303 | establishment of chromosome localization : biological\_process | 5 | 0.0989903 | 1.51428e-08 | 0.0017628 |
| GO:0050000 | chromosome localization : biological\_process | 5 | 0.0989903 | 1.51428e-08 | 0.0017628 |
| GO:0006996 | organelle organization : biological\_process | 32 | 10.4064 | 1.68919e-08 | 0.00196641 |
| GO:0005875 | microtubule associated complex : cellular\_component | 12 | 1.41061 | 1.79116e-08 | 0.00208512 |
| GO:0006323 | DNA packaging : biological\_process | 11 | 1.16314 | 2.33482e-08 | 0.00271801 |
| GO:0030705 | cytoskeleton-dependent intracellular transport : biological\_process | 12 | 1.52198 | 4.22839e-08 | 0.00492235 |
| GO:0007076 | mitotic chromosome condensation : biological\_process | 6 | 0.222728 | 5.54073e-08 | 0.00645008 |
| GO:0000166 | nucleotide binding : molecular\_function | 52 | 24.5743 | 1.41753e-07 | 0.0165017 |
| GO:0007052 | mitotic spindle organization and biogenesis : biological\_process | 5 | 0.148485 | 2.05644e-07 | 0.0239394 |
| GO:0007094 | mitotic cell cycle spindle assembly checkpoint : biological\_process | 4 | 0.0742427 | 3.3671e-07 | 0.0391971 |
| GO:0048015 | phosphoinositide-mediated signaling : biological\_process | 10 | 1.23738 | 4.58644e-07 | 0.0533916 |
| GO:0045840 | positive regulation of mitosis : biological\_process | 5 | 0.185607 | 7.56373e-07 | 0.0880509 |
| GO:0003774 | motor activity : molecular\_function | 11 | 1.7447 | 1.4923e-06 | 0.173722 |
| GO:0005657 | replication fork : cellular\_component | 6 | 0.383587 | 1.92028e-06 | 0.223543 |
| GO:0051325 | interphase : biological\_process | 9 | 1.16314 | 2.50849e-06 | 0.292018 |
| GO:0008283 | cell proliferation : biological\_process | 27 | 9.9609 | 2.73342e-06 | 0.318203 |
| GO:0000785 | chromatin : cellular\_component | 11 | 1.93031 | 4.01098e-06 | 0.466926 |
| GO:0006950 | response to stress : biological\_process | 35 | 15.4796 | 5.20398e-06 | 0.605806 |
| GO:0007096 | regulation of exit from mitosis : biological\_process | 4 | 0.136112 | 7.05426e-06 | 0.821201 |
| GO:0000940 | outer kinetochore of condensed chromosome : cellular\_component | 3 | 0.0494951 | 7.41997e-06 | 0.863774 |
| GO:0034508 | centromere complex assembly : biological\_process | 3 | 0.0494951 | 7.41997e-06 | 0.863774 |
| GO:0051383 | kinetochore organization : biological\_process | 3 | 0.0494951 | 7.41997e-06 | 0.863774 |
| GO:0005815 | microtubule organizing center : cellular\_component | 12 | 2.52425 | 9.48666e-06 | 1 |
| GO:0010458 | exit from mitosis : biological\_process | 4 | 0.148485 | 1.04786e-05 | 1 |
| GO:0051640 | organelle localization : biological\_process | 7 | 0.767175 | 1.12476e-05 | 1 |
| GO:0051329 | interphase of mitotic cell cycle : biological\_process | 8 | 1.10127 | 1.46294e-05 | 1 |
| GO:0051310 | metaphase plate congression : biological\_process | 3 | 0.0618689 | 1.83799e-05 | 1 |
| GO:0051439 | regulation of ubiquitin-protein ligase activity during mitotic cell cycle : biological\_process | 7 | 0.829044 | 1.88718e-05 | 1 |
| GO:0051438 | regulation of ubiquitin-protein ligase activity : biological\_process | 7 | 0.890913 | 3.03331e-05 | 1 |
| GO:0045787 | positive regulation of cell cycle : biological\_process | 5 | 0.383587 | 3.6402e-05 | 1 |
| GO:0043596 | nuclear replication fork : cellular\_component | 4 | 0.197981 | 3.70528e-05 | 1 |
| GO:0051656 | establishment of organelle localization : biological\_process | 6 | 0.631063 | 3.81933e-05 | 1 |
| GO:0044454 | nuclear chromosome part : cellular\_component | 7 | 0.928034 | 3.96042e-05 | 1 |
| GO:0051340 | regulation of ligase activity : biological\_process | 7 | 0.928034 | 3.96042e-05 | 1 |
| GO:0005871 | kinesin complex : cellular\_component | 4 | 0.210354 | 4.79837e-05 | 1 |
| GO:0000228 | nuclear chromosome : cellular\_component | 8 | 1.29925 | 4.87366e-05 | 1 |
| GO:0000910 | cytokinesis : biological\_process | 5 | 0.420709 | 5.78369e-05 | 1 |
| GO:0019932 | second-messenger-mediated signaling : biological\_process | 11 | 2.72223 | 9.79473e-05 | 1 |

## MSCs batch 2 induced

|  |  |  |  |  |  |
| --- | --- | --- | --- | --- | --- |
| id | name | genes | expect | P | adj P |
| GO:0048731 | system development : biological\_process | 47 | 15.7977 | 6.28008e-12 | 7.31077e-07 |
| GO:0005576 | extracellular region : cellular\_component | 46 | 16.0554 | 4.04832e-11 | 4.71273e-06 |
| GO:0007399 | nervous system development : biological\_process | 26 | 6.67442 | 3.06728e-09 | 0.000357069 |
| GO:0009888 | tissue development : biological\_process | 17 | 3.01039 | 1.01235e-08 | 0.0011785 |
| GO:0048869 | cellular developmental process : biological\_process | 34 | 11.8206 | 2.13789e-08 | 0.00248876 |
| GO:0030154 | cell differentiation : biological\_process | 32 | 10.7711 | 2.81577e-08 | 0.00327789 |
| GO:0048729 | tissue morphogenesis : biological\_process | 8 | 0.736488 | 7.30652e-07 | 0.0850567 |
| GO:0044421 | extracellular region part : cellular\_component | 22 | 6.80331 | 1.31681e-06 | 0.153293 |
| GO:0048513 | organ development : biological\_process | 30 | 11.793 | 2.14223e-06 | 0.249381 |
| GO:0007166 | cell surface receptor linked signal transduction : biological\_process | 32 | 13.1095 | 2.24971e-06 | 0.261893 |
| GO:0008544 | epidermis development : biological\_process | 9 | 1.28885 | 6.08278e-06 | 0.708108 |
| GO:0007398 | ectoderm development : biological\_process | 9 | 1.38091 | 1.06495e-05 | 1 |
| GO:0048730 | epidermis morphogenesis : biological\_process | 6 | 0.524748 | 1.3799e-05 | 1 |
| GO:0060113 | inner ear receptor cell differentiation : biological\_process | 4 | 0.16571 | 1.92374e-05 | 1 |
| GO:0005509 | calcium ion binding : molecular\_function | 22 | 8.13819 | 2.24823e-05 | 1 |
| GO:0042490 | mechanoreceptor differentiation : biological\_process | 4 | 0.184122 | 3.00234e-05 | 1 |
| GO:0035315 | hair cell differentiation : biological\_process | 4 | 0.193328 | 3.68215e-05 | 1 |
| GO:0008283 | cell proliferation : biological\_process | 20 | 7.41091 | 5.55562e-05 | 1 |
| GO:0009913 | epidermal cell differentiation : biological\_process | 5 | 0.432687 | 7.02493e-05 | 1 |
| GO:0048839 | inner ear development : biological\_process | 5 | 0.441893 | 7.78367e-05 | 1 |
| GO:0045595 | regulation of cell differentiation : biological\_process | 9 | 1.78598 | 8.0718e-05 | 1 |

## MSCs batch 3 repressed

|  |  |  |  |  |  |
| --- | --- | --- | --- | --- | --- |
| id | name | genes | expect | P | adj P |
| GO:0005576 | extracellular region : cellular\_component | 95 | 26.1548 | 5.5823e-30 | 6.49847e-25 |
| GO:0031012 | extracellular matrix : cellular\_component | 38 | 4.51411 | 3.79761e-24 | 4.42087e-19 |
| GO:0005578 | proteinaceous extracellular matrix : cellular\_component | 37 | 4.31914 | 8.40076e-24 | 9.7795e-19 |
| GO:0044421 | extracellular region part : cellular\_component | 54 | 11.0828 | 2.21362e-22 | 2.57692e-17 |
| GO:0044420 | extracellular matrix part : cellular\_component | 18 | 1.28974 | 5.41324e-16 | 6.30166e-11 |
| GO:0005201 | extracellular matrix structural constituent : molecular\_function | 17 | 1.22976 | 4.22436e-15 | 4.91766e-10 |
| GO:0048513 | organ development : biological\_process | 59 | 19.2112 | 7.38183e-15 | 8.59334e-10 |
| GO:0048731 | system development : biological\_process | 67 | 25.7349 | 2.21107e-13 | 2.57395e-08 |
| GO:0001871 | pattern binding : molecular\_function | 18 | 1.82964 | 3.27112e-13 | 3.80798e-08 |
| GO:0030247 | polysaccharide binding : molecular\_function | 17 | 1.60468 | 4.32034e-13 | 5.02939e-08 |
| GO:0007155 | cell adhesion : biological\_process | 40 | 10.8129 | 9.4332e-13 | 1.09814e-07 |
| GO:0022610 | biological adhesion : biological\_process | 40 | 10.8129 | 9.4332e-13 | 1.09814e-07 |
| GO:0005581 | collagen : cellular\_component | 11 | 0.524896 | 2.18196e-12 | 2.54006e-07 |
| GO:0005539 | glycosaminoglycan binding : molecular\_function | 16 | 1.54469 | 3.06246e-12 | 3.56508e-07 |
| GO:0005198 | structural molecule activity : molecular\_function | 34 | 9.71808 | 2.43099e-10 | 2.82997e-05 |
| GO:0001501 | skeletal development : biological\_process | 16 | 2.47451 | 4.02851e-09 | 0.000468967 |
| GO:0009888 | tissue development : biological\_process | 22 | 4.90403 | 5.1573e-09 | 0.000600371 |
| GO:0030246 | carbohydrate binding : molecular\_function | 20 | 4.15418 | 7.96566e-09 | 0.000927298 |
| GO:0030198 | extracellular matrix organization and biogenesis : biological\_process | 10 | 0.839834 | 9.60568e-09 | 0.00111822 |
| GO:0030199 | collagen fibril organization : biological\_process | 7 | 0.299941 | 1.04421e-08 | 0.00121559 |
| GO:0008201 | heparin binding : molecular\_function | 11 | 1.21476 | 3.43722e-08 | 0.00400134 |
| GO:0001944 | vasculature development : biological\_process | 16 | 2.99941 | 6.25196e-08 | 0.00727803 |
| GO:0005583 | fibrillar collagen : cellular\_component | 5 | 0.134973 | 8.80496e-08 | 0.01025 |
| GO:0009611 | response to wounding : biological\_process | 23 | 6.28376 | 1.00396e-07 | 0.0116873 |
| GO:0009605 | response to external stimulus : biological\_process | 29 | 9.53811 | 1.17027e-07 | 0.0136233 |
| GO:0001568 | blood vessel development : biological\_process | 15 | 2.93942 | 2.89008e-07 | 0.033644 |
| GO:0005102 | receptor binding : molecular\_function | 31 | 11.2028 | 3.34319e-07 | 0.0389187 |
| GO:0043062 | extracellular structure organization : biological\_process | 11 | 1.5297 | 3.82117e-07 | 0.044483 |
| GO:0007160 | cell-matrix adhesion : biological\_process | 10 | 1.28974 | 6.42512e-07 | 0.0747961 |
| GO:0007166 | cell surface receptor linked signal transduction : biological\_process | 46 | 21.3558 | 6.64912e-07 | 0.0774038 |
| GO:0004867 | serine-type endopeptidase inhibitor activity : molecular\_function | 10 | 1.31974 | 7.97685e-07 | 0.0928602 |
| GO:0007167 | enzyme linked receptor protein signaling pathway : biological\_process | 19 | 5.039 | 8.56711e-07 | 0.0997315 |
| GO:0005604 | basement membrane : cellular\_component | 8 | 0.779846 | 9.90032e-07 | 0.115252 |
| GO:0031589 | cell-substrate adhesion : biological\_process | 10 | 1.36473 | 1.09171e-06 | 0.127088 |
| GO:0004866 | endopeptidase inhibitor activity : molecular\_function | 12 | 2.06959 | 1.17812e-06 | 0.137148 |
| GO:0030414 | protease inhibitor activity : molecular\_function | 12 | 2.12958 | 1.59648e-06 | 0.185849 |
| GO:0009887 | organ morphogenesis : biological\_process | 21 | 6.52371 | 2.87387e-06 | 0.334553 |
| GO:0019838 | growth factor binding : molecular\_function | 9 | 1.24475 | 4.23805e-06 | 0.49336 |
| GO:0004857 | enzyme inhibitor activity : molecular\_function | 15 | 3.68927 | 5.00303e-06 | 0.582413 |
| GO:0005540 | hyaluronic acid binding : molecular\_function | 5 | 0.269947 | 5.35806e-06 | 0.623743 |
| GO:0008544 | epidermis development : biological\_process | 11 | 2.09958 | 8.78675e-06 | 1 |
| GO:0005615 | extracellular space : cellular\_component | 21 | 7.0636 | 9.75746e-06 | 1 |
| GO:0048514 | blood vessel morphogenesis : biological\_process | 12 | 2.57949 | 1.16353e-05 | 1 |
| GO:0001502 | cartilage condensation : biological\_process | 4 | 0.164967 | 1.50615e-05 | 1 |
| GO:0007398 | ectoderm development : biological\_process | 11 | 2.24955 | 1.69008e-05 | 1 |
| GO:0007229 | integrin-mediated signaling pathway : biological\_process | 7 | 0.839834 | 1.96778e-05 | 1 |
| GO:0051216 | cartilage development : biological\_process | 6 | 0.599881 | 2.7058e-05 | 1 |
| GO:0005520 | insulin-like growth factor binding : molecular\_function | 5 | 0.374926 | 3.04828e-05 | 1 |
| GO:0006928 | cell motion : biological\_process | 18 | 6.16378 | 5.34203e-05 | 1 |
| GO:0051674 | localization of cell : biological\_process | 18 | 6.16378 | 5.34203e-05 | 1 |
| GO:0001525 | angiogenesis : biological\_process | 10 | 2.12958 | 5.76531e-05 | 1 |
| GO:0006959 | humoral immune response : biological\_process | 7 | 1.0048 | 6.41091e-05 | 1 |
| GO:0002526 | acute inflammatory response : biological\_process | 7 | 1.04979 | 8.50514e-05 | 1 |

## MSCs batch 3 induced

|  |  |  |  |  |  |
| --- | --- | --- | --- | --- | --- |
| id | name | genes | expect | P | adj P |
| GO:0006950 | response to stress : biological\_process | 50 | 26.0057 | 7.02667e-06 | 0.817988 |
| GO:0006986 | response to unfolded protein : biological\_process | 8 | 1.12255 | 1.46978e-05 | 1 |
| GO:0051789 | response to protein stimulus : biological\_process | 8 | 1.12255 | 1.46978e-05 | 1 |
| GO:0042221 | response to chemical stimulus : biological\_process | 27 | 11.9947 | 8.24229e-05 | 1 |
| GO:0048731 | system development : biological\_process | 59 | 35.6721 | 8.66654e-05 | 1 |
| GO:0042060 | wound healing : biological\_process | 11 | 2.70244 | 8.71762e-05 | 1 |

## MSCs batch 4 repressed

|  |  |  |  |  |  |
| --- | --- | --- | --- | --- | --- |
| id | name | genes | expect | P | adj P |
| GO:0007565 | female pregnancy : biological\_process | 6 | 0.330529 | 9.63851e-07 | 0.112204 |
| GO:0005576 | extracellular region : cellular\_component | 23 | 9.14987 | 2.94286e-05 | 1 |

## MSCs batch 4 induced

|  |  |  |  |  |  |
| --- | --- | --- | --- | --- | --- |
| id | name | genes | expect | P | adj P |
| GO:0005576 | extracellular region : cellular\_component | 71 | 22.2704 | 3.9445e-19 | 4.59187e-14 |
| GO:0044421 | extracellular region part : cellular\_component | 42 | 9.43684 | 3.40484e-16 | 3.96364e-11 |
| GO:0048731 | system development : biological\_process | 61 | 21.9129 | 1.16613e-13 | 1.35752e-08 |
| GO:0031226 | intrinsic to plasma membrane : cellular\_component | 47 | 14.6469 | 1.00692e-12 | 1.17217e-07 |
| GO:0005887 | integral to plasma membrane : cellular\_component | 46 | 14.4681 | 2.52228e-12 | 2.93623e-07 |
| GO:0044459 | plasma membrane part : cellular\_component | 57 | 22.3343 | 3.01106e-11 | 3.50523e-06 |
| GO:0007166 | cell surface receptor linked signal transduction : biological\_process | 49 | 18.1841 | 1.57437e-10 | 1.83275e-05 |
| GO:0007399 | nervous system development : biological\_process | 33 | 9.25807 | 2.53531e-10 | 2.9514e-05 |
| GO:0005615 | extracellular space : cellular\_component | 26 | 6.01455 | 4.19841e-10 | 4.88745e-05 |
| GO:0031012 | extracellular matrix : cellular\_component | 20 | 3.84369 | 2.10197e-09 | 0.000244695 |
| GO:0005578 | proteinaceous extracellular matrix : cellular\_component | 19 | 3.67769 | 6.04837e-09 | 0.000704103 |
| GO:0005509 | calcium ion binding : molecular\_function | 33 | 11.2885 | 3.40011e-08 | 0.00395813 |
| GO:0007155 | cell adhesion : biological\_process | 29 | 9.20699 | 5.12269e-08 | 0.00596342 |
| GO:0022610 | biological adhesion : biological\_process | 29 | 9.20699 | 5.12269e-08 | 0.00596342 |
| GO:0005102 | receptor binding : molecular\_function | 29 | 9.539 | 1.089e-07 | 0.0126773 |
| GO:0048699 | generation of neurons : biological\_process | 17 | 3.53722 | 1.11154e-07 | 0.0129397 |
| GO:0048468 | cell development : biological\_process | 22 | 6.19333 | 3.11133e-07 | 0.0362196 |
| GO:0022008 | neurogenesis : biological\_process | 17 | 3.80539 | 3.1443e-07 | 0.0366034 |
| GO:0048513 | organ development : biological\_process | 39 | 16.358 | 3.85273e-07 | 0.0448505 |
| GO:0004872 | receptor activity : molecular\_function | 40 | 17.0732 | 4.19603e-07 | 0.0488468 |
| GO:0004888 | transmembrane receptor activity : molecular\_function | 29 | 10.4457 | 7.13354e-07 | 0.083043 |
| GO:0048523 | negative regulation of cellular process : biological\_process | 36 | 14.9023 | 8.41039e-07 | 0.097907 |
| GO:0030154 | cell differentiation : biological\_process | 36 | 14.9406 | 8.93187e-07 | 0.103978 |
| GO:0048666 | neuron development : biological\_process | 13 | 2.42625 | 1.06192e-06 | 0.12362 |
| GO:0051093 | negative regulation of developmental process : biological\_process | 18 | 4.64819 | 1.13158e-06 | 0.13173 |
| GO:0048519 | negative regulation of biological process : biological\_process | 37 | 15.9239 | 1.46842e-06 | 0.170942 |
| GO:0007167 | enzyme linked receptor protein signaling pathway : biological\_process | 17 | 4.29064 | 1.65764e-06 | 0.19297 |
| GO:0004714 | transmembrane receptor protein tyrosine kinase activity : molecular\_function | 8 | 0.855573 | 2.1587e-06 | 0.251298 |
| GO:0048869 | cellular developmental process : biological\_process | 37 | 16.3964 | 2.90434e-06 | 0.3381 |
| GO:0030182 | neuron differentiation : biological\_process | 14 | 3.11582 | 3.30292e-06 | 0.384499 |
| GO:0009888 | tissue development : biological\_process | 16 | 4.17571 | 5.16138e-06 | 0.600846 |
| GO:0042613 | MHC class II protein complex : cellular\_component | 4 | 0.140467 | 7.98962e-06 | 0.930087 |
| GO:0031175 | neurite development : biological\_process | 11 | 2.13255 | 1.03853e-05 | 1 |
| GO:0019199 | transmembrane receptor protein kinase activity : molecular\_function | 8 | 1.05989 | 1.095e-05 | 1 |
| GO:0004871 | signal transducer activity : molecular\_function | 44 | 22.5003 | 1.24093e-05 | 1 |
| GO:0060089 | molecular transducer activity : molecular\_function | 44 | 22.5003 | 1.24093e-05 | 1 |
| GO:0050793 | regulation of developmental process : biological\_process | 27 | 10.8926 | 1.40904e-05 | 1 |
| GO:0001657 | ureteric bud development : biological\_process | 5 | 0.344783 | 2.09535e-05 | 1 |
| GO:0009790 | embryonic development : biological\_process | 15 | 4.20125 | 2.34097e-05 | 1 |
| GO:0002504 | antigen processing and presentation of peptide or polysaccharide antigen via MHC class II : biological\_process | 4 | 0.178776 | 2.35133e-05 | 1 |
| GO:0042325 | regulation of phosphorylation : biological\_process | 8 | 1.23867 | 3.44099e-05 | 1 |
| GO:0002376 | immune system process : biological\_process | 26 | 10.8415 | 3.58336e-05 | 1 |
| GO:0000904 | cell morphogenesis involved in differentiation : biological\_process | 10 | 2.03039 | 3.90361e-05 | 1 |
| GO:0001932 | regulation of protein amino acid phosphorylation : biological\_process | 7 | 0.944961 | 4.43167e-05 | 1 |
| GO:0009887 | organ morphogenesis : biological\_process | 17 | 5.55484 | 4.78001e-05 | 1 |
| GO:0007267 | cell-cell signaling : biological\_process | 20 | 7.3043 | 4.97293e-05 | 1 |
| GO:0019220 | regulation of phosphate metabolic process : biological\_process | 8 | 1.31528 | 5.30093e-05 | 1 |
| GO:0051174 | regulation of phosphorus metabolic process : biological\_process | 8 | 1.31528 | 5.30093e-05 | 1 |
| GO:0005003 | ephrin receptor activity : molecular\_function | 4 | 0.217086 | 5.42427e-05 | 1 |
| GO:0007409 | axonogenesis : biological\_process | 9 | 1.74946 | 6.79321e-05 | 1 |
| GO:0009605 | response to external stimulus : biological\_process | 21 | 8.12156 | 7.29892e-05 | 1 |
| GO:0048729 | tissue morphogenesis : biological\_process | 7 | 1.02158 | 7.33113e-05 | 1 |
| GO:0008544 | epidermis development : biological\_process | 9 | 1.78776 | 8.03361e-05 | 1 |
| GO:0032268 | regulation of cellular protein metabolic process : biological\_process | 13 | 3.63938 | 8.16538e-05 | 1 |
| GO:0048730 | epidermis morphogenesis : biological\_process | 6 | 0.727876 | 8.60499e-05 | 1 |
| GO:0001656 | metanephros development : biological\_process | 5 | 0.459711 | 8.909e-05 | 1 |
| GO:0048812 | neurite morphogenesis : biological\_process | 9 | 1.83884 | 9.97893e-05 | 1 |
| GO:0048667 | cell morphogenesis involved in neuron differentiation : biological\_process | 9 | 1.83884 | 9.97893e-05 | 1 |

## MSC rank products repressed

|  |  |  |  |  |  |
| --- | --- | --- | --- | --- | --- |
| id | name | genes | expect | P | adj P |
| GO:0005578 | proteinaceous extracellular matrix : cellular\_component | 5 | 0.456147 | 8.34973e-05 | 1 |

## MSC rank products induced

|  |  |  |  |  |  |
| --- | --- | --- | --- | --- | --- |
| id | name | genes | expect | P | adj P |
| GO:0005576 | extracellular region : cellular\_component | 23 | 7.85508 | 1.95291e-06 | 0.227342 |
| GO:0005887 | integral to plasma membrane : cellular\_component | 18 | 5.1031 | 2.51755e-06 | 0.293073 |
| GO:0031226 | intrinsic to plasma membrane : cellular\_component | 18 | 5.16616 | 2.9922e-06 | 0.348328 |
| GO:0007399 | nervous system development : biological\_process | 14 | 3.26544 | 4.28586e-06 | 0.498925 |
| GO:0048731 | system development : biological\_process | 22 | 7.72896 | 5.47141e-06 | 0.636937 |
| GO:0048869 | cellular developmental process : biological\_process | 18 | 5.78321 | 1.41663e-05 | 1 |
| GO:0030154 | cell differentiation : biological\_process | 17 | 5.26975 | 1.61311e-05 | 1 |
| GO:0009888 | tissue development : biological\_process | 9 | 1.47283 | 1.66994e-05 | 1 |
| GO:0045608 | negative regulation of auditory receptor cell differentiation : biological\_process | 2 | 0.00900812 | 2.00646e-05 | 1 |
| GO:0045632 | negative regulation of mechanoreceptor differentiation : biological\_process | 2 | 0.00900812 | 2.00646e-05 | 1 |
| GO:0007417 | central nervous system development : biological\_process | 8 | 1.16655 | 2.22183e-05 | 1 |
| GO:0044459 | plasma membrane part : cellular\_component | 21 | 7.8776 | 2.54135e-05 | 1 |
| GO:0007166 | cell surface receptor linked signal transduction : biological\_process | 18 | 6.41378 | 5.58187e-05 | 1 |
| GO:0045631 | regulation of mechanoreceptor differentiation : biological\_process | 2 | 0.0135122 | 6.0017e-05 | 1 |
| GO:0045607 | regulation of auditory receptor cell differentiation : biological\_process | 2 | 0.0135122 | 6.0017e-05 | 1 |
| GO:0060113 | inner ear receptor cell differentiation : biological\_process | 3 | 0.0810731 | 6.86791e-05 | 1 |
| GO:0042490 | mechanoreceptor differentiation : biological\_process | 3 | 0.0900812 | 9.53246e-05 | 1 |
| GO:0048518 | positive regulation of biological process : biological\_process | 16 | 5.48144 | 9.77428e-05 | 1 |

  
**MSCs batch 1 repressed and**GO:0007067**: 26 genes, expected 5.61453, P=8.42571e-11, P adjusted = 9.80853e-06

|  |  |  |
| --- | --- | --- |
| 9787 | DLGAP5 | discs, large (Drosophila) homolog-associated protein 5 |
| 701 | BUB1B | BUB1 budding uninhibited by benzimidazoles 1 homolog beta (yeast) |
| 54443 | ANLN | anillin, actin binding protein |
| 1906 | EDN1 | endothelin 1 |
| 10459 | MAD2L2 | MAD2 mitotic arrest deficient-like 2 (yeast) |
| 10615 | SPAG5 | sperm associated antigen 5 |
| 55165 | CEP55 | centrosomal protein 55kDa |
| 11004 | KIF2C | kinesin family member 2C |
| 57405 | SPC25 | SPC25, NDC80 kinetochore complex component, homolog (S. cerevisiae) |
| 332 | BIRC5 | baculoviral IAP repeat-containing 5 |
| 259266 | ASPM | asp (abnormal spindle) homolog, microcephaly associated (Drosophila) |
| 4751 | NEK2 | NIMA (never in mitosis gene a)-related kinase 2 |
| 22974 | TPX2 | TPX2, microtubule-associated, homolog (Xenopus laevis) |
| 51203 | NUSAP1 | nucleolar and spindle associated protein 1 |
| 1063 | CENPF | centromere protein F, 350/400ka (mitosin) |
| 10403 | NDC80 | NDC80 homolog, kinetochore complex component (S. cerevisiae) |
| 9133 | CCNB2 | cyclin B2 |
| 4739 | NEDD9 | neural precursor cell expressed, developmentally down-regulated 9 |
| 64151 | NCAPG | non-SMC condensin I complex, subunit G |
| 983 | CDC2 | cell division cycle 2, G1 to S and G2 to M |
| 55872 | PBK | PDZ binding kinase |
| 83540 | NUF2 | NUF2, NDC80 kinetochore complex component, homolog (S. cerevisiae) |
| 83461 | CDCA3 | cell division cycle associated 3 |
| 991 | CDC20 | cell division cycle 20 homolog (S. cerevisiae) |
| 11065 | UBE2C | ubiquitin-conjugating enzyme E2C |
| 890 | CCNA2 | cyclin A2 |

  
  
**MSCs batch 1 repressed and**GO:0000087**: 26 genes, expected 5.66878, P=1.04558e-10, P adjusted = 1.21718e-05

|  |  |  |
| --- | --- | --- |
| 9787 | DLGAP5 | discs, large (Drosophila) homolog-associated protein 5 |
| 701 | BUB1B | BUB1 budding uninhibited by benzimidazoles 1 homolog beta (yeast) |
| 54443 | ANLN | anillin, actin binding protein |
| 1906 | EDN1 | endothelin 1 |
| 10459 | MAD2L2 | MAD2 mitotic arrest deficient-like 2 (yeast) |
| 10615 | SPAG5 | sperm associated antigen 5 |
| 55165 | CEP55 | centrosomal protein 55kDa |
| 11004 | KIF2C | kinesin family member 2C |
| 57405 | SPC25 | SPC25, NDC80 kinetochore complex component, homolog (S. cerevisiae) |
| 332 | BIRC5 | baculoviral IAP repeat-containing 5 |
| 259266 | ASPM | asp (abnormal spindle) homolog, microcephaly associated (Drosophila) |
| 4751 | NEK2 | NIMA (never in mitosis gene a)-related kinase 2 |
| 22974 | TPX2 | TPX2, microtubule-associated, homolog (Xenopus laevis) |
| 51203 | NUSAP1 | nucleolar and spindle associated protein 1 |
| 1063 | CENPF | centromere protein F, 350/400ka (mitosin) |
| 10403 | NDC80 | NDC80 homolog, kinetochore complex component (S. cerevisiae) |
| 9133 | CCNB2 | cyclin B2 |
| 4739 | NEDD9 | neural precursor cell expressed, developmentally down-regulated 9 |
| 64151 | NCAPG | non-SMC condensin I complex, subunit G |
| 983 | CDC2 | cell division cycle 2, G1 to S and G2 to M |
| 55872 | PBK | PDZ binding kinase |
| 83540 | NUF2 | NUF2, NDC80 kinetochore complex component, homolog (S. cerevisiae) |
| 83461 | CDCA3 | cell division cycle associated 3 |
| 991 | CDC20 | cell division cycle 20 homolog (S. cerevisiae) |
| 11065 | UBE2C | ubiquitin-conjugating enzyme E2C |
| 890 | CCNA2 | cyclin A2 |

  
  
**MSCs batch 1 repressed and**GO:0005975**: 38 genes, expected 11.663, P=1.82828e-10, P adjusted = 2.12834e-05

|  |  |  |
| --- | --- | --- |
| 1301 | COL11A1 | collagen, type XI, alpha 1 |
| 5163 | PDK1 | pyruvate dehydrogenase kinase, isozyme 1 |
| 226 | ALDOA | aldolase A, fructose-bisphosphate |
| 5106 | PCK2 | phosphoenolpyruvate carboxykinase 2 (mitochondrial) |
| 51148 | CERCAM | cerebral endothelial cell adhesion molecule |
| 6518 | SLC2A5 | solute carrier family 2 (facilitated glucose/fructose transporter), member 5 |
| 8707 | B3GALT2 | UDP-Gal:betaGlcNAc beta 1,3-galactosyltransferase, polypeptide 2 |
| 114898 | C1QTNF2 | C1q and tumor necrosis factor related protein 2 |
| 5230 | PGK1 | phosphoglycerate kinase 1 |
| 176 | ACAN | aggrecan |
| 5210 | PFKFB4 | 6-phosphofructo-2-kinase/fructose-2,6-biphosphatase 4 |
| 3425 | IDUA | iduronidase, alpha-L- |
| 5214 | PFKP | phosphofructokinase, platelet |
| 54344 | DPM3 | dolichyl-phosphate mannosyltransferase polypeptide 3 |
| 2132 | EXT2 | exostoses (multiple) 2 |
| 7167 | TPI1 | triosephosphate isomerase 1 |
| 2023 | ENO1 | enolase 1, (alpha) |
| 5211 | PFKL | phosphofructokinase, liver |
| 5165 | PDK3 | pyruvate dehydrogenase kinase, isozyme 3 |
| 25796 | PGLS | 6-phosphogluconolactonase |
| 2597 | GAPDH | glyceraldehyde-3-phosphate dehydrogenase |
| 2821 | GPI | glucose phosphate isomerase |
| 203054 | ADCK5 | aarF domain containing kinase 5 |
| 4351 | MPI | mannose phosphate isomerase |
| 3418 | IDH2 | isocitrate dehydrogenase 2 (NADP+), mitochondrial |
| 5372 | PMM1 | phosphomannomutase 1 |
| 3421 | IDH3G | isocitrate dehydrogenase 3 (NAD+) gamma |
| 8574 | AKR7A2 | aldo-keto reductase family 7, member A2 (aflatoxin aldehyde reductase) |
| 4668 | NAGA | N-acetylgalactosaminidase, alpha- |
| 2026 | ENO2 | enolase 2 (gamma, neuronal) |
| 1116 | CHI3L1 | chitinase 3-like 1 (cartilage glycoprotein-39) |
| 3038 | HAS3 | hyaluronan synthase 3 |
| 2582 | GALE | UDP-galactose-4-epimerase |
| 11285 | B4GALT7 | xylosylprotein beta 1,4-galactosyltransferase, polypeptide 7 (galactosyltransferase I) |
| 2997 | GYS1 | glycogen synthase 1 (muscle) |
| 230 | ALDOC | aldolase C, fructose-bisphosphate |
| 6483 | ST3GAL2 | ST3 beta-galactoside alpha-2,3-sialyltransferase 2 |
| 55501 | CHST12 | carbohydrate (chondroitin 4) sulfotransferase 12 |

  
  
**MSCs batch 1 repressed and**GO:0044420**: 16 genes, expected 2.33261, P=1.15636e-09, P adjusted = 0.000134614

|  |  |  |
| --- | --- | --- |
| 1301 | COL11A1 | collagen, type XI, alpha 1 |
| 1284 | COL4A2 | collagen, type IV, alpha 2 |
| 2200 | FBN1 | fibrillin 1 |
| 4015 | LOX | lysyl oxidase |
| 1307 | COL16A1 | collagen, type XVI, alpha 1 |
| 176 | ACAN | aggrecan |
| 3339 | HSPG2 | heparan sulfate proteoglycan 2 |
| 1303 | COL12A1 | collagen, type XII, alpha 1 |
| 8076 | MFAP5 | microfibrillar associated protein 5 |
| 30008 | EFEMP2 | EGF-containing fibulin-like extracellular matrix protein 2 |
| 7078 | TIMP3 | TIMP metallopeptidase inhibitor 3 |
| 283 | ANG | angiogenin, ribonuclease, RNase A family, 5 |
| 222663 | SCUBE3 | signal peptide, CUB domain, EGF-like 3 |
| 1300 | COL10A1 | collagen, type X, alpha 1 |
| 3908 | LAMA2 | laminin, alpha 2 |
| 4237 | MFAP2 | microfibrillar-associated protein 2 |

  
  
**MSCs batch 1 repressed and**GO:0022403**: 33 genes, expected 9.79153, P=1.26238e-09, P adjusted = 0.000146956

|  |  |  |
| --- | --- | --- |
| 9787 | DLGAP5 | discs, large (Drosophila) homolog-associated protein 5 |
| 701 | BUB1B | BUB1 budding uninhibited by benzimidazoles 1 homolog beta (yeast) |
| 7272 | TTK | TTK protein kinase |
| 54443 | ANLN | anillin, actin binding protein |
| 7164 | TPD52L1 | tumor protein D52-like 1 |
| 1906 | EDN1 | endothelin 1 |
| 10459 | MAD2L2 | MAD2 mitotic arrest deficient-like 2 (yeast) |
| 10615 | SPAG5 | sperm associated antigen 5 |
| 55165 | CEP55 | centrosomal protein 55kDa |
| 8558 | CDK10 | cyclin-dependent kinase 10 |
| 9055 | PRC1 | protein regulator of cytokinesis 1 |
| 1033 | CDKN3 | cyclin-dependent kinase inhibitor 3 |
| 11004 | KIF2C | kinesin family member 2C |
| 57405 | SPC25 | SPC25, NDC80 kinetochore complex component, homolog (S. cerevisiae) |
| 332 | BIRC5 | baculoviral IAP repeat-containing 5 |
| 259266 | ASPM | asp (abnormal spindle) homolog, microcephaly associated (Drosophila) |
| 4751 | NEK2 | NIMA (never in mitosis gene a)-related kinase 2 |
| 22974 | TPX2 | TPX2, microtubule-associated, homolog (Xenopus laevis) |
| 51203 | NUSAP1 | nucleolar and spindle associated protein 1 |
| 1063 | CENPF | centromere protein F, 350/400ka (mitosin) |
| 10403 | NDC80 | NDC80 homolog, kinetochore complex component (S. cerevisiae) |
| 9133 | CCNB2 | cyclin B2 |
| 4739 | NEDD9 | neural precursor cell expressed, developmentally down-regulated 9 |
| 64151 | NCAPG | non-SMC condensin I complex, subunit G |
| 983 | CDC2 | cell division cycle 2, G1 to S and G2 to M |
| 55872 | PBK | PDZ binding kinase |
| 3855 | KRT7 | keratin 7 |
| 83540 | NUF2 | NUF2, NDC80 kinetochore complex component, homolog (S. cerevisiae) |
| 83461 | CDCA3 | cell division cycle associated 3 |
| 991 | CDC20 | cell division cycle 20 homolog (S. cerevisiae) |
| 11065 | UBE2C | ubiquitin-conjugating enzyme E2C |
| 595 | CCND1 | cyclin D1 |
| 890 | CCNA2 | cyclin A2 |

  
  
**MSCs batch 1 repressed and**GO:0044262**: 29 genes, expected 7.81152, P=1.42094e-09, P adjusted = 0.000165415

|  |  |  |
| --- | --- | --- |
| 1301 | COL11A1 | collagen, type XI, alpha 1 |
| 5163 | PDK1 | pyruvate dehydrogenase kinase, isozyme 1 |
| 226 | ALDOA | aldolase A, fructose-bisphosphate |
| 5106 | PCK2 | phosphoenolpyruvate carboxykinase 2 (mitochondrial) |
| 51148 | CERCAM | cerebral endothelial cell adhesion molecule |
| 114898 | C1QTNF2 | C1q and tumor necrosis factor related protein 2 |
| 5230 | PGK1 | phosphoglycerate kinase 1 |
| 176 | ACAN | aggrecan |
| 5210 | PFKFB4 | 6-phosphofructo-2-kinase/fructose-2,6-biphosphatase 4 |
| 3425 | IDUA | iduronidase, alpha-L- |
| 5214 | PFKP | phosphofructokinase, platelet |
| 2132 | EXT2 | exostoses (multiple) 2 |
| 7167 | TPI1 | triosephosphate isomerase 1 |
| 2023 | ENO1 | enolase 1, (alpha) |
| 5211 | PFKL | phosphofructokinase, liver |
| 5165 | PDK3 | pyruvate dehydrogenase kinase, isozyme 3 |
| 25796 | PGLS | 6-phosphogluconolactonase |
| 2597 | GAPDH | glyceraldehyde-3-phosphate dehydrogenase |
| 2821 | GPI | glucose phosphate isomerase |
| 3418 | IDH2 | isocitrate dehydrogenase 2 (NADP+), mitochondrial |
| 5372 | PMM1 | phosphomannomutase 1 |
| 2026 | ENO2 | enolase 2 (gamma, neuronal) |
| 1116 | CHI3L1 | chitinase 3-like 1 (cartilage glycoprotein-39) |
| 2582 | GALE | UDP-galactose-4-epimerase |
| 11285 | B4GALT7 | xylosylprotein beta 1,4-galactosyltransferase, polypeptide 7 (galactosyltransferase I) |
| 2997 | GYS1 | glycogen synthase 1 (muscle) |
| 230 | ALDOC | aldolase C, fructose-bisphosphate |
| 6483 | ST3GAL2 | ST3 beta-galactoside alpha-2,3-sialyltransferase 2 |
| 55501 | CHST12 | carbohydrate (chondroitin 4) sulfotransferase 12 |

  
  
**MSCs batch 1 repressed and**GO:0000278**: 33 genes, expected 9.95427, P=1.91723e-09, P adjusted = 0.000223188

|  |  |  |
| --- | --- | --- |
| 9787 | DLGAP5 | discs, large (Drosophila) homolog-associated protein 5 |
| 701 | BUB1B | BUB1 budding uninhibited by benzimidazoles 1 homolog beta (yeast) |
| 440 | ASNS | asparagine synthetase |
| 7272 | TTK | TTK protein kinase |
| 54443 | ANLN | anillin, actin binding protein |
| 7164 | TPD52L1 | tumor protein D52-like 1 |
| 1906 | EDN1 | endothelin 1 |
| 10459 | MAD2L2 | MAD2 mitotic arrest deficient-like 2 (yeast) |
| 10615 | SPAG5 | sperm associated antigen 5 |
| 55165 | CEP55 | centrosomal protein 55kDa |
| 8558 | CDK10 | cyclin-dependent kinase 10 |
| 9055 | PRC1 | protein regulator of cytokinesis 1 |
| 1033 | CDKN3 | cyclin-dependent kinase inhibitor 3 |
| 11004 | KIF2C | kinesin family member 2C |
| 57405 | SPC25 | SPC25, NDC80 kinetochore complex component, homolog (S. cerevisiae) |
| 332 | BIRC5 | baculoviral IAP repeat-containing 5 |
| 259266 | ASPM | asp (abnormal spindle) homolog, microcephaly associated (Drosophila) |
| 4751 | NEK2 | NIMA (never in mitosis gene a)-related kinase 2 |
| 22974 | TPX2 | TPX2, microtubule-associated, homolog (Xenopus laevis) |
| 51203 | NUSAP1 | nucleolar and spindle associated protein 1 |
| 1063 | CENPF | centromere protein F, 350/400ka (mitosin) |
| 10403 | NDC80 | NDC80 homolog, kinetochore complex component (S. cerevisiae) |
| 9133 | CCNB2 | cyclin B2 |
| 4739 | NEDD9 | neural precursor cell expressed, developmentally down-regulated 9 |
| 64151 | NCAPG | non-SMC condensin I complex, subunit G |
| 983 | CDC2 | cell division cycle 2, G1 to S and G2 to M |
| 55872 | PBK | PDZ binding kinase |
| 83540 | NUF2 | NUF2, NDC80 kinetochore complex component, homolog (S. cerevisiae) |
| 83461 | CDCA3 | cell division cycle associated 3 |
| 991 | CDC20 | cell division cycle 20 homolog (S. cerevisiae) |
| 11065 | UBE2C | ubiquitin-conjugating enzyme E2C |
| 595 | CCND1 | cyclin D1 |
| 890 | CCNA2 | cyclin A2 |

  
  
**MSCs batch 1 repressed and**GO:0051301**: 25 genes, expected 6.37399, P=6.41684e-09, P adjusted = 0.000746997

|  |  |  |
| --- | --- | --- |
| 4628 | MYH10 | myosin, heavy chain 10, non-muscle |
| 701 | BUB1B | BUB1 budding uninhibited by benzimidazoles 1 homolog beta (yeast) |
| 54443 | ANLN | anillin, actin binding protein |
| 55909 | BIN3 | bridging integrator 3 |
| 10459 | MAD2L2 | MAD2 mitotic arrest deficient-like 2 (yeast) |
| 10615 | SPAG5 | sperm associated antigen 5 |
| 55165 | CEP55 | centrosomal protein 55kDa |
| 9055 | PRC1 | protein regulator of cytokinesis 1 |
| 57405 | SPC25 | SPC25, NDC80 kinetochore complex component, homolog (S. cerevisiae) |
| 332 | BIRC5 | baculoviral IAP repeat-containing 5 |
| 259266 | ASPM | asp (abnormal spindle) homolog, microcephaly associated (Drosophila) |
| 4751 | NEK2 | NIMA (never in mitosis gene a)-related kinase 2 |
| 51203 | NUSAP1 | nucleolar and spindle associated protein 1 |
| 1063 | CENPF | centromere protein F, 350/400ka (mitosin) |
| 10403 | NDC80 | NDC80 homolog, kinetochore complex component (S. cerevisiae) |
| 9133 | CCNB2 | cyclin B2 |
| 4739 | NEDD9 | neural precursor cell expressed, developmentally down-regulated 9 |
| 64151 | NCAPG | non-SMC condensin I complex, subunit G |
| 983 | CDC2 | cell division cycle 2, G1 to S and G2 to M |
| 83540 | NUF2 | NUF2, NDC80 kinetochore complex component, homolog (S. cerevisiae) |
| 83461 | CDCA3 | cell division cycle associated 3 |
| 991 | CDC20 | cell division cycle 20 homolog (S. cerevisiae) |
| 11065 | UBE2C | ubiquitin-conjugating enzyme E2C |
| 595 | CCND1 | cyclin D1 |
| 890 | CCNA2 | cyclin A2 |

  
  
**MSCs batch 1 repressed and**GO:0000279**: 28 genes, expected 7.86577, P=6.97067e-09, P adjusted = 0.00081147

|  |  |  |
| --- | --- | --- |
| 9787 | DLGAP5 | discs, large (Drosophila) homolog-associated protein 5 |
| 701 | BUB1B | BUB1 budding uninhibited by benzimidazoles 1 homolog beta (yeast) |
| 7272 | TTK | TTK protein kinase |
| 54443 | ANLN | anillin, actin binding protein |
| 1906 | EDN1 | endothelin 1 |
| 10459 | MAD2L2 | MAD2 mitotic arrest deficient-like 2 (yeast) |
| 10615 | SPAG5 | sperm associated antigen 5 |
| 55165 | CEP55 | centrosomal protein 55kDa |
| 9055 | PRC1 | protein regulator of cytokinesis 1 |
| 11004 | KIF2C | kinesin family member 2C |
| 57405 | SPC25 | SPC25, NDC80 kinetochore complex component, homolog (S. cerevisiae) |
| 332 | BIRC5 | baculoviral IAP repeat-containing 5 |
| 259266 | ASPM | asp (abnormal spindle) homolog, microcephaly associated (Drosophila) |
| 4751 | NEK2 | NIMA (never in mitosis gene a)-related kinase 2 |
| 22974 | TPX2 | TPX2, microtubule-associated, homolog (Xenopus laevis) |
| 51203 | NUSAP1 | nucleolar and spindle associated protein 1 |
| 1063 | CENPF | centromere protein F, 350/400ka (mitosin) |
| 10403 | NDC80 | NDC80 homolog, kinetochore complex component (S. cerevisiae) |
| 9133 | CCNB2 | cyclin B2 |
| 4739 | NEDD9 | neural precursor cell expressed, developmentally down-regulated 9 |
| 64151 | NCAPG | non-SMC condensin I complex, subunit G |
| 983 | CDC2 | cell division cycle 2, G1 to S and G2 to M |
| 55872 | PBK | PDZ binding kinase |
| 83540 | NUF2 | NUF2, NDC80 kinetochore complex component, homolog (S. cerevisiae) |
| 83461 | CDCA3 | cell division cycle associated 3 |
| 991 | CDC20 | cell division cycle 20 homolog (S. cerevisiae) |
| 11065 | UBE2C | ubiquitin-conjugating enzyme E2C |
| 890 | CCNA2 | cyclin A2 |

  
  
**MSCs batch 1 repressed and**GO:0022402**: 35 genes, expected 13.2091, P=1.82919e-07, P adjusted = 0.021294

|  |  |  |
| --- | --- | --- |
| 9787 | DLGAP5 | discs, large (Drosophila) homolog-associated protein 5 |
| 4628 | MYH10 | myosin, heavy chain 10, non-muscle |
| 701 | BUB1B | BUB1 budding uninhibited by benzimidazoles 1 homolog beta (yeast) |
| 6300 | MAPK12 | mitogen-activated protein kinase 12 |
| 7272 | TTK | TTK protein kinase |
| 54443 | ANLN | anillin, actin binding protein |
| 7164 | TPD52L1 | tumor protein D52-like 1 |
| 1906 | EDN1 | endothelin 1 |
| 10459 | MAD2L2 | MAD2 mitotic arrest deficient-like 2 (yeast) |
| 10615 | SPAG5 | sperm associated antigen 5 |
| 55165 | CEP55 | centrosomal protein 55kDa |
| 8558 | CDK10 | cyclin-dependent kinase 10 |
| 9055 | PRC1 | protein regulator of cytokinesis 1 |
| 1033 | CDKN3 | cyclin-dependent kinase inhibitor 3 |
| 11004 | KIF2C | kinesin family member 2C |
| 57405 | SPC25 | SPC25, NDC80 kinetochore complex component, homolog (S. cerevisiae) |
| 332 | BIRC5 | baculoviral IAP repeat-containing 5 |
| 259266 | ASPM | asp (abnormal spindle) homolog, microcephaly associated (Drosophila) |
| 4751 | NEK2 | NIMA (never in mitosis gene a)-related kinase 2 |
| 22974 | TPX2 | TPX2, microtubule-associated, homolog (Xenopus laevis) |
| 51203 | NUSAP1 | nucleolar and spindle associated protein 1 |
| 1063 | CENPF | centromere protein F, 350/400ka (mitosin) |
| 10403 | NDC80 | NDC80 homolog, kinetochore complex component (S. cerevisiae) |
| 9133 | CCNB2 | cyclin B2 |
| 4739 | NEDD9 | neural precursor cell expressed, developmentally down-regulated 9 |
| 64151 | NCAPG | non-SMC condensin I complex, subunit G |
| 983 | CDC2 | cell division cycle 2, G1 to S and G2 to M |
| 55872 | PBK | PDZ binding kinase |
| 3855 | KRT7 | keratin 7 |
| 83540 | NUF2 | NUF2, NDC80 kinetochore complex component, homolog (S. cerevisiae) |
| 83461 | CDCA3 | cell division cycle associated 3 |
| 991 | CDC20 | cell division cycle 20 homolog (S. cerevisiae) |
| 11065 | UBE2C | ubiquitin-conjugating enzyme E2C |
| 595 | CCND1 | cyclin D1 |
| 890 | CCNA2 | cyclin A2 |

  
  
**MSCs batch 1 repressed and**GO:0006091**: 25 genes, expected 7.62166, P=2.19383e-07, P adjusted = 0.0255388

|  |  |  |
| --- | --- | --- |
| 226 | ALDOA | aldolase A, fructose-bisphosphate |
| 10873 | ME3 | malic enzyme 3, NADP(+)-dependent, mitochondrial |
| 114898 | C1QTNF2 | C1q and tumor necrosis factor related protein 2 |
| 5230 | PGK1 | phosphoglycerate kinase 1 |
| 5214 | PFKP | phosphofructokinase, platelet |
| 4776 | NFATC4 | nuclear factor of activated T-cells, cytoplasmic, calcineurin-dependent 4 |
| 4713 | NDUFB7 | NADH dehydrogenase (ubiquinone) 1 beta subcomplex, 7, 18kDa |
| 4714 | NDUFB8 | NADH dehydrogenase (ubiquinone) 1 beta subcomplex, 8, 19kDa |
| 55937 | APOM | apolipoprotein M |
| 7167 | TPI1 | triosephosphate isomerase 1 |
| 2023 | ENO1 | enolase 1, (alpha) |
| 5211 | PFKL | phosphofructokinase, liver |
| 2597 | GAPDH | glyceraldehyde-3-phosphate dehydrogenase |
| 2821 | GPI | glucose phosphate isomerase |
| 513 | ATP5D | ATP synthase, H+ transporting, mitochondrial F1 complex, delta subunit |
| 9605 | C16orf7 | chromosome 16 open reading frame 7 |
| 1535 | CYBA | cytochrome b-245, alpha polypeptide |
| 3418 | IDH2 | isocitrate dehydrogenase 2 (NADP+), mitochondrial |
| 3421 | IDH3G | isocitrate dehydrogenase 3 (NAD+) gamma |
| 2026 | ENO2 | enolase 2 (gamma, neuronal) |
| 3995 | FADS3 | fatty acid desaturase 3 |
| 374291 | NDUFS7 | NADH dehydrogenase (ubiquinone) Fe-S protein 7, 20kDa (NADH-coenzyme Q reductase) |
| 2997 | GYS1 | glycogen synthase 1 (muscle) |
| 230 | ALDOC | aldolase C, fructose-bisphosphate |
| 7384 | UQCRC1 | ubiquinol-cytochrome c reductase core protein I |

  
  
**MSCs batch 1 repressed and**GO:0005856**: 62 genes, expected 31.436, P=2.47573e-07, P adjusted = 0.0288204

|  |  |  |
| --- | --- | --- |
| 9787 | DLGAP5 | discs, large (Drosophila) homolog-associated protein 5 |
| 10112 | KIF20A | kinesin family member 20A |
| 9814 | SFI1 | Sfi1 homolog, spindle assembly associated (yeast) |
| 4628 | MYH10 | myosin, heavy chain 10, non-muscle |
| 701 | BUB1B | BUB1 budding uninhibited by benzimidazoles 1 homolog beta (yeast) |
| 57153 | SLC44A2 | solute carrier family 44, member 2 |
| 23265 | EXOC7 | exocyst complex component 7 |
| 59 | ACTA2 | actin, alpha 2, smooth muscle, aorta |
| 3883 | KRT33A | keratin 33A |
| 89796 | NAV1 | neuron navigator 1 |
| 26502 | NARF | nuclear prelamin A recognition factor |
| 822 | CAPG | capping protein (actin filament), gelsolin-like |
| 7272 | TTK | TTK protein kinase |
| 5411 | PNN | pinin, desmosome associated protein |
| 54443 | ANLN | anillin, actin binding protein |
| 6444 | SGCD | sarcoglycan, delta (35kDa dystrophin-associated glycoprotein) |
| 51778 | MYOZ2 | myozenin 2 |
| 6640 | SNTA1 | syntrophin, alpha 1 (dystrophin-associated protein A1, 59kDa, acidic component) |
| 6676 | SPAG4 | sperm associated antigen 4 |
| 10460 | TACC3 | transforming, acidic coiled-coil containing protein 3 |
| 57175 | CORO1B | coronin, actin binding protein, 1B |
| 55909 | BIN3 | bridging integrator 3 |
| 10615 | SPAG5 | sperm associated antigen 5 |
| 11344 | TWF2 | twinfilin, actin-binding protein, homolog 2 (Drosophila) |
| 4000 | LMNA | lamin A/C |
| 7153 | TOP2A | topoisomerase (DNA) II alpha 170kDa |
| 2316 | FLNA | filamin A, alpha (actin binding protein 280) |
| 9055 | PRC1 | protein regulator of cytokinesis 1 |
| 25802 | LMOD1 | leiomodin 1 (smooth muscle) |
| 51466 | EVL | Enah/Vasp-like |
| 7263 | TST | thiosulfate sulfurtransferase (rhodanese) |
| 6509 | SLC1A4 | solute carrier family 1 (glutamate/neutral amino acid transporter), member 4 |
| 4644 | MYO5A | myosin VA (heavy chain 12, myoxin) |
| 11004 | KIF2C | kinesin family member 2C |
| 3885 | KRT34 | keratin 34 |
| 332 | BIRC5 | baculoviral IAP repeat-containing 5 |
| 91978 | C19orf20 | chromosome 19 open reading frame 20 |
| 5531 | PPP4C | protein phosphatase 4 (formerly X), catalytic subunit |
| 4641 | MYO1C | myosin IC |
| 259266 | ASPM | asp (abnormal spindle) homolog, microcephaly associated (Drosophila) |
| 9928 | KIF14 | kinesin family member 14 |
| 4751 | NEK2 | NIMA (never in mitosis gene a)-related kinase 2 |
| 22974 | TPX2 | TPX2, microtubule-associated, homolog (Xenopus laevis) |
| 51203 | NUSAP1 | nucleolar and spindle associated protein 1 |
| 1063 | CENPF | centromere protein F, 350/400ka (mitosin) |
| 2312 | FLG | filaggrin |
| 9133 | CCNB2 | cyclin B2 |
| 4134 | MAP4 | microtubule-associated protein 4 |
| 4739 | NEDD9 | neural precursor cell expressed, developmentally down-regulated 9 |
| 8318 | CDC45L | CDC45 cell division cycle 45-like (S. cerevisiae) |
| 11078 | TRIOBP | TRIO and F-actin binding protein |
| 3315 | HSPB1 | heat shock 27kDa protein 1 |
| 983 | CDC2 | cell division cycle 2, G1 to S and G2 to M |
| 3855 | KRT7 | keratin 7 |
| 55742 | PARVA | parvin, alpha |
| 7430 | EZR | ezrin |
| 800 | CALD1 | caldesmon 1 |
| 991 | CDC20 | cell division cycle 20 homolog (S. cerevisiae) |
| 148170 | CDC42EP5 | CDC42 effector protein (Rho GTPase binding) 5 |
| 10160 | FARP1 | FERM, RhoGEF (ARHGEF) and pleckstrin domain protein 1 (chondrocyte-derived) |
| 10398 | MYL9 | myosin, light chain 9, regulatory |
| 64236 | PDLIM2 | PDZ and LIM domain 2 (mystique) |

  
  
**MSCs batch 1 repressed and**GO:0019318**: 17 genes, expected 3.87864, P=3.52892e-07, P adjusted = 0.0410808

|  |  |  |
| --- | --- | --- |
| 5163 | PDK1 | pyruvate dehydrogenase kinase, isozyme 1 |
| 226 | ALDOA | aldolase A, fructose-bisphosphate |
| 5106 | PCK2 | phosphoenolpyruvate carboxykinase 2 (mitochondrial) |
| 5230 | PGK1 | phosphoglycerate kinase 1 |
| 5210 | PFKFB4 | 6-phosphofructo-2-kinase/fructose-2,6-biphosphatase 4 |
| 5214 | PFKP | phosphofructokinase, platelet |
| 7167 | TPI1 | triosephosphate isomerase 1 |
| 2023 | ENO1 | enolase 1, (alpha) |
| 5211 | PFKL | phosphofructokinase, liver |
| 5165 | PDK3 | pyruvate dehydrogenase kinase, isozyme 3 |
| 25796 | PGLS | 6-phosphogluconolactonase |
| 2597 | GAPDH | glyceraldehyde-3-phosphate dehydrogenase |
| 2821 | GPI | glucose phosphate isomerase |
| 5372 | PMM1 | phosphomannomutase 1 |
| 2026 | ENO2 | enolase 2 (gamma, neuronal) |
| 2582 | GALE | UDP-galactose-4-epimerase |
| 230 | ALDOC | aldolase C, fructose-bisphosphate |

  
  
**MSCs batch 1 repressed and**GO:0007049**: 48 genes, expected 22.214, P=4.88295e-07, P adjusted = 0.0568434

|  |  |  |
| --- | --- | --- |
| 9787 | DLGAP5 | discs, large (Drosophila) homolog-associated protein 5 |
| 4628 | MYH10 | myosin, heavy chain 10, non-muscle |
| 701 | BUB1B | BUB1 budding uninhibited by benzimidazoles 1 homolog beta (yeast) |
| 440 | ASNS | asparagine synthetase |
| 6300 | MAPK12 | mitogen-activated protein kinase 12 |
| 4288 | MKI67 | antigen identified by monoclonal antibody Ki-67 |
| 4176 | MCM7 | minichromosome maintenance complex component 7 |
| 55215 | FANCI | Fanconi anemia, complementation group I |
| 7272 | TTK | TTK protein kinase |
| 5411 | PNN | pinin, desmosome associated protein |
| 1841 | DTYMK | deoxythymidylate kinase (thymidylate kinase) |
| 54443 | ANLN | anillin, actin binding protein |
| 10460 | TACC3 | transforming, acidic coiled-coil containing protein 3 |
| 7164 | TPD52L1 | tumor protein D52-like 1 |
| 1906 | EDN1 | endothelin 1 |
| 55909 | BIN3 | bridging integrator 3 |
| 10459 | MAD2L2 | MAD2 mitotic arrest deficient-like 2 (yeast) |
| 10615 | SPAG5 | sperm associated antigen 5 |
| 55165 | CEP55 | centrosomal protein 55kDa |
| 2132 | EXT2 | exostoses (multiple) 2 |
| 8558 | CDK10 | cyclin-dependent kinase 10 |
| 9055 | PRC1 | protein regulator of cytokinesis 1 |
| 64975 | MRPL41 | mitochondrial ribosomal protein L41 |
| 112464 | PRKCDBP | protein kinase C, delta binding protein |
| 1033 | CDKN3 | cyclin-dependent kinase inhibitor 3 |
| 11004 | KIF2C | kinesin family member 2C |
| 29108 | PYCARD | PYD and CARD domain containing |
| 57405 | SPC25 | SPC25, NDC80 kinetochore complex component, homolog (S. cerevisiae) |
| 332 | BIRC5 | baculoviral IAP repeat-containing 5 |
| 259266 | ASPM | asp (abnormal spindle) homolog, microcephaly associated (Drosophila) |
| 4751 | NEK2 | NIMA (never in mitosis gene a)-related kinase 2 |
| 22974 | TPX2 | TPX2, microtubule-associated, homolog (Xenopus laevis) |
| 51203 | NUSAP1 | nucleolar and spindle associated protein 1 |
| 1063 | CENPF | centromere protein F, 350/400ka (mitosin) |
| 10403 | NDC80 | NDC80 homolog, kinetochore complex component (S. cerevisiae) |
| 9133 | CCNB2 | cyclin B2 |
| 4739 | NEDD9 | neural precursor cell expressed, developmentally down-regulated 9 |
| 8318 | CDC45L | CDC45 cell division cycle 45-like (S. cerevisiae) |
| 64151 | NCAPG | non-SMC condensin I complex, subunit G |
| 983 | CDC2 | cell division cycle 2, G1 to S and G2 to M |
| 55872 | PBK | PDZ binding kinase |
| 3855 | KRT7 | keratin 7 |
| 83540 | NUF2 | NUF2, NDC80 kinetochore complex component, homolog (S. cerevisiae) |
| 83461 | CDCA3 | cell division cycle associated 3 |
| 991 | CDC20 | cell division cycle 20 homolog (S. cerevisiae) |
| 11065 | UBE2C | ubiquitin-conjugating enzyme E2C |
| 595 | CCND1 | cyclin D1 |
| 890 | CCNA2 | cyclin A2 |

  
  
**MSCs batch 1 repressed and**GO:0006007**: 11 genes, expected 1.6274, P=5.43991e-07, P adjusted = 0.0633271

|  |  |  |
| --- | --- | --- |
| 226 | ALDOA | aldolase A, fructose-bisphosphate |
| 5230 | PGK1 | phosphoglycerate kinase 1 |
| 5214 | PFKP | phosphofructokinase, platelet |
| 7167 | TPI1 | triosephosphate isomerase 1 |
| 2023 | ENO1 | enolase 1, (alpha) |
| 5211 | PFKL | phosphofructokinase, liver |
| 25796 | PGLS | 6-phosphogluconolactonase |
| 2597 | GAPDH | glyceraldehyde-3-phosphate dehydrogenase |
| 2821 | GPI | glucose phosphate isomerase |
| 2026 | ENO2 | enolase 2 (gamma, neuronal) |
| 230 | ALDOC | aldolase C, fructose-bisphosphate |

  
  
**MSCs batch 1 repressed and**GO:0005996**: 17 genes, expected 4.01425, P=5.78354e-07, P adjusted = 0.0673273

|  |  |  |
| --- | --- | --- |
| 5163 | PDK1 | pyruvate dehydrogenase kinase, isozyme 1 |
| 226 | ALDOA | aldolase A, fructose-bisphosphate |
| 5106 | PCK2 | phosphoenolpyruvate carboxykinase 2 (mitochondrial) |
| 5230 | PGK1 | phosphoglycerate kinase 1 |
| 5210 | PFKFB4 | 6-phosphofructo-2-kinase/fructose-2,6-biphosphatase 4 |
| 5214 | PFKP | phosphofructokinase, platelet |
| 7167 | TPI1 | triosephosphate isomerase 1 |
| 2023 | ENO1 | enolase 1, (alpha) |
| 5211 | PFKL | phosphofructokinase, liver |
| 5165 | PDK3 | pyruvate dehydrogenase kinase, isozyme 3 |
| 25796 | PGLS | 6-phosphogluconolactonase |
| 2597 | GAPDH | glyceraldehyde-3-phosphate dehydrogenase |
| 2821 | GPI | glucose phosphate isomerase |
| 5372 | PMM1 | phosphomannomutase 1 |
| 2026 | ENO2 | enolase 2 (gamma, neuronal) |
| 2582 | GALE | UDP-galactose-4-epimerase |
| 230 | ALDOC | aldolase C, fructose-bisphosphate |

  
  
**MSCs batch 1 repressed and**GO:0005576**: 82 genes, expected 47.3031, P=5.91648e-07, P adjusted = 0.0688749

|  |  |  |
| --- | --- | --- |
| 147015 | DHRS13 | dehydrogenase/reductase (SDR family) member 13 |
| 3625 | INHBB | inhibin, beta B |
| 1301 | COL11A1 | collagen, type XI, alpha 1 |
| 11341 | SCRG1 | scrapie responsive protein 1 |
| 1284 | COL4A2 | collagen, type IV, alpha 2 |
| 2200 | FBN1 | fibrillin 1 |
| 57493 | HEG1 | HEG homolog 1 (zebrafish) |
| 114898 | C1QTNF2 | C1q and tumor necrosis factor related protein 2 |
| 3486 | IGFBP3 | insulin-like growth factor binding protein 3 |
| 4015 | LOX | lysyl oxidase |
| 4016 | LOXL1 | lysyl oxidase-like 1 |
| 1307 | COL16A1 | collagen, type XVI, alpha 1 |
| 5549 | PRELP | proline/arginine-rich end leucine-rich repeat protein |
| 176 | ACAN | aggrecan |
| 151887 | CCDC80 | coiled-coil domain containing 80 |
| 3339 | HSPG2 | heparan sulfate proteoglycan 2 |
| 4017 | LOXL2 | lysyl oxidase-like 2 |
| 5605 | MAP2K2 | mitogen-activated protein kinase kinase 2 |
| 1303 | COL12A1 | collagen, type XII, alpha 1 |
| 4282 | MIF | macrophage migration inhibitory factor (glycosylation-inhibiting factor) |
| 5054 | SERPINE1 | serpin peptidase inhibitor, clade E (nexin, plasminogen activator inhibitor type 1), member 1 |
| 147372 | CCBE1 | collagen and calcium binding EGF domains 1 |
| 8076 | MFAP5 | microfibrillar associated protein 5 |
| 2202 | EFEMP1 | EGF-containing fibulin-like extracellular matrix protein 1 |
| 1906 | EDN1 | endothelin 1 |
| 7059 | THBS3 | thrombospondin 3 |
| 6038 | RNASE4 | ribonuclease, RNase A family, 4 |
| 2817 | GPC1 | glypican 1 |
| 633 | BGN | biglycan |
| 30008 | EFEMP2 | EGF-containing fibulin-like extracellular matrix protein 2 |
| 3485 | IGFBP2 | insulin-like growth factor binding protein 2, 36kDa |
| 5125 | PCSK5 | proprotein convertase subtilisin/kexin type 5 |
| 55937 | APOM | apolipoprotein M |
| 7078 | TIMP3 | TIMP metallopeptidase inhibitor 3 |
| 283 | ANG | angiogenin, ribonuclease, RNase A family, 5 |
| 4684 | NCAM1 | neural cell adhesion molecule 1 |
| 5552 | SRGN | serglycin |
| 6050 | RNH1 | ribonuclease/angiogenin inhibitor 1 |
| 3381 | IBSP | integrin-binding sialoprotein |
| 9358 | ITGBL1 | integrin, beta-like 1 (with EGF-like repeat domains) |
| 56944 | OLFML3 | olfactomedin-like 3 |
| 3575 | IL7R | interleukin 7 receptor |
| 29108 | PYCARD | PYD and CARD domain containing |
| 51368 | TEX264 | testis expressed 264 |
| 1292 | COL6A2 | collagen, type VI, alpha 2 |
| 2821 | GPI | glucose phosphate isomerase |
| 222663 | SCUBE3 | signal peptide, CUB domain, EGF-like 3 |
| 976 | CD97 | CD97 molecule |
| 4582 | MUC1 | mucin 1, cell surface associated |
| 114990 | VASN | vasorin |
| 26227 | PHGDH | phosphoglycerate dehydrogenase |
| 84171 | LOXL4 | lysyl oxidase-like 4 |
| 1893 | ECM1 | extracellular matrix protein 1 |
| 1404 | HAPLN1 | hyaluronan and proteoglycan link protein 1 |
| 3084 | NRG1 | neuregulin 1 |
| 56005 | C19orf10 | chromosome 19 open reading frame 10 |
| 2621 | GAS6 | growth arrest-specific 6 |
| 7423 | VEGFB | vascular endothelial growth factor B |
| 5764 | PTN | pleiotrophin |
| 5118 | PCOLCE | procollagen C-endopeptidase enhancer |
| 5034 | P4HB | procollagen-proline, 2-oxoglutarate 4-dioxygenase (proline 4-hydroxylase), beta polypeptide |
| 8532 | CPZ | carboxypeptidase Z |
| 25817 | FAM19A5 | family with sequence similarity 19 (chemokine (C-C motif)-like), member A5 |
| 6781 | STC1 | stanniocalcin 1 |
| 3956 | LGALS1 | lectin, galactoside-binding, soluble, 1 |
| 1300 | COL10A1 | collagen, type X, alpha 1 |
| 5176 | SERPINF1 | serpin peptidase inhibitor, clade F (alpha-2 antiplasmin, pigment epithelium derived factor), member 1 |
| 1116 | CHI3L1 | chitinase 3-like 1 (cartilage glycoprotein-39) |
| 84279 | C2orf7 | chromosome 2 open reading frame 7 |
| 8635 | RNASET2 | ribonuclease T2 |
| 6424 | SFRP4 | secreted frizzled-related protein 4 |
| 7057 | THBS1 | thrombospondin 1 |
| 6483 | ST3GAL2 | ST3 beta-galactoside alpha-2,3-sialyltransferase 2 |
| 6051 | RNPEP | arginyl aminopeptidase (aminopeptidase B) |
| 165 | AEBP1 | AE binding protein 1 |
| 3908 | LAMA2 | laminin, alpha 2 |
| 130574 | LYPD6 | LY6/PLAUR domain containing 6 |
| 4054 | LTBP3 | latent transforming growth factor beta binding protein 3 |
| 23383 | KIAA0892 | KIAA0892 |
| 710 | SERPING1 | serpin peptidase inhibitor, clade G (C1 inhibitor), member 1 |
| 4237 | MFAP2 | microfibrillar-associated protein 2 |
| 649 | BMP1 | bone morphogenetic protein 1 |

  
  
**MSCs batch 1 repressed and**GO:0005819**: 13 genes, expected 2.35973, P=6.09523e-07, P adjusted = 0.0709558

|  |  |  |
| --- | --- | --- |
| 9787 | DLGAP5 | discs, large (Drosophila) homolog-associated protein 5 |
| 701 | BUB1B | BUB1 budding uninhibited by benzimidazoles 1 homolog beta (yeast) |
| 7272 | TTK | TTK protein kinase |
| 10615 | SPAG5 | sperm associated antigen 5 |
| 9055 | PRC1 | protein regulator of cytokinesis 1 |
| 332 | BIRC5 | baculoviral IAP repeat-containing 5 |
| 259266 | ASPM | asp (abnormal spindle) homolog, microcephaly associated (Drosophila) |
| 9928 | KIF14 | kinesin family member 14 |
| 22974 | TPX2 | TPX2, microtubule-associated, homolog (Xenopus laevis) |
| 1063 | CENPF | centromere protein F, 350/400ka (mitosin) |
| 4739 | NEDD9 | neural precursor cell expressed, developmentally down-regulated 9 |
| 983 | CDC2 | cell division cycle 2, G1 to S and G2 to M |
| 991 | CDC20 | cell division cycle 20 homolog (S. cerevisiae) |

  
  
**MSCs batch 1 repressed and**GO:0006096**: 10 genes, expected 1.35617, P=7.68637e-07, P adjusted = 0.0894785

|  |  |  |
| --- | --- | --- |
| 226 | ALDOA | aldolase A, fructose-bisphosphate |
| 5230 | PGK1 | phosphoglycerate kinase 1 |
| 5214 | PFKP | phosphofructokinase, platelet |
| 7167 | TPI1 | triosephosphate isomerase 1 |
| 2023 | ENO1 | enolase 1, (alpha) |
| 5211 | PFKL | phosphofructokinase, liver |
| 2597 | GAPDH | glyceraldehyde-3-phosphate dehydrogenase |
| 2821 | GPI | glucose phosphate isomerase |
| 2026 | ENO2 | enolase 2 (gamma, neuronal) |
| 230 | ALDOC | aldolase C, fructose-bisphosphate |

  
  
**MSCs batch 1 repressed and**GO:0031012**: 25 genes, expected 8.16413, P=7.96737e-07, P adjusted = 0.0927497

|  |  |  |
| --- | --- | --- |
| 1301 | COL11A1 | collagen, type XI, alpha 1 |
| 1284 | COL4A2 | collagen, type IV, alpha 2 |
| 2200 | FBN1 | fibrillin 1 |
| 4015 | LOX | lysyl oxidase |
| 1307 | COL16A1 | collagen, type XVI, alpha 1 |
| 5549 | PRELP | proline/arginine-rich end leucine-rich repeat protein |
| 176 | ACAN | aggrecan |
| 3339 | HSPG2 | heparan sulfate proteoglycan 2 |
| 1303 | COL12A1 | collagen, type XII, alpha 1 |
| 8076 | MFAP5 | microfibrillar associated protein 5 |
| 2202 | EFEMP1 | EGF-containing fibulin-like extracellular matrix protein 1 |
| 2817 | GPC1 | glypican 1 |
| 633 | BGN | biglycan |
| 30008 | EFEMP2 | EGF-containing fibulin-like extracellular matrix protein 2 |
| 7078 | TIMP3 | TIMP metallopeptidase inhibitor 3 |
| 283 | ANG | angiogenin, ribonuclease, RNase A family, 5 |
| 1292 | COL6A2 | collagen, type VI, alpha 2 |
| 222663 | SCUBE3 | signal peptide, CUB domain, EGF-like 3 |
| 1893 | ECM1 | extracellular matrix protein 1 |
| 1404 | HAPLN1 | hyaluronan and proteoglycan link protein 1 |
| 8532 | CPZ | carboxypeptidase Z |
| 1300 | COL10A1 | collagen, type X, alpha 1 |
| 1116 | CHI3L1 | chitinase 3-like 1 (cartilage glycoprotein-39) |
| 3908 | LAMA2 | laminin, alpha 2 |
| 4237 | MFAP2 | microfibrillar-associated protein 2 |

  
  
**MSCs batch 1 repressed and**GO:0007346**: 14 genes, expected 2.84795, P=9.4254e-07, P adjusted = 0.109723

|  |  |  |
| --- | --- | --- |
| 9787 | DLGAP5 | discs, large (Drosophila) homolog-associated protein 5 |
| 701 | BUB1B | BUB1 budding uninhibited by benzimidazoles 1 homolog beta (yeast) |
| 440 | ASNS | asparagine synthetase |
| 7272 | TTK | TTK protein kinase |
| 54443 | ANLN | anillin, actin binding protein |
| 1906 | EDN1 | endothelin 1 |
| 10459 | MAD2L2 | MAD2 mitotic arrest deficient-like 2 (yeast) |
| 8558 | CDK10 | cyclin-dependent kinase 10 |
| 332 | BIRC5 | baculoviral IAP repeat-containing 5 |
| 4751 | NEK2 | NIMA (never in mitosis gene a)-related kinase 2 |
| 51203 | NUSAP1 | nucleolar and spindle associated protein 1 |
| 1063 | CENPF | centromere protein F, 350/400ka (mitosin) |
| 11065 | UBE2C | ubiquitin-conjugating enzyme E2C |
| 890 | CCNA2 | cyclin A2 |

  
  
**MSCs batch 1 repressed and**GO:0000777**: 9 genes, expected 1.08493, P=9.61085e-07, P adjusted = 0.111882

|  |  |  |
| --- | --- | --- |
| 701 | BUB1B | BUB1 budding uninhibited by benzimidazoles 1 homolog beta (yeast) |
| 10615 | SPAG5 | sperm associated antigen 5 |
| 57405 | SPC25 | SPC25, NDC80 kinetochore complex component, homolog (S. cerevisiae) |
| 79019 | CENPM | centromere protein M |
| 1063 | CENPF | centromere protein F, 350/400ka (mitosin) |
| 1058 | CENPA | centromere protein A |
| 10403 | NDC80 | NDC80 homolog, kinetochore complex component (S. cerevisiae) |
| 83540 | NUF2 | NUF2, NDC80 kinetochore complex component, homolog (S. cerevisiae) |
| 80152 | CENPT | centromere protein T |

  
  
**MSCs batch 1 repressed and**GO:0006006**: 14 genes, expected 2.9022, P=1.18899e-06, P adjusted = 0.138412

|  |  |  |
| --- | --- | --- |
| 5163 | PDK1 | pyruvate dehydrogenase kinase, isozyme 1 |
| 226 | ALDOA | aldolase A, fructose-bisphosphate |
| 5106 | PCK2 | phosphoenolpyruvate carboxykinase 2 (mitochondrial) |
| 5230 | PGK1 | phosphoglycerate kinase 1 |
| 5214 | PFKP | phosphofructokinase, platelet |
| 7167 | TPI1 | triosephosphate isomerase 1 |
| 2023 | ENO1 | enolase 1, (alpha) |
| 5211 | PFKL | phosphofructokinase, liver |
| 5165 | PDK3 | pyruvate dehydrogenase kinase, isozyme 3 |
| 25796 | PGLS | 6-phosphogluconolactonase |
| 2597 | GAPDH | glyceraldehyde-3-phosphate dehydrogenase |
| 2821 | GPI | glucose phosphate isomerase |
| 2026 | ENO2 | enolase 2 (gamma, neuronal) |
| 230 | ALDOC | aldolase C, fructose-bisphosphate |

  
  
**MSCs batch 1 repressed and**GO:0000779**: 9 genes, expected 1.11206, P=1.20201e-06, P adjusted = 0.139929

|  |  |  |
| --- | --- | --- |
| 701 | BUB1B | BUB1 budding uninhibited by benzimidazoles 1 homolog beta (yeast) |
| 10615 | SPAG5 | sperm associated antigen 5 |
| 57405 | SPC25 | SPC25, NDC80 kinetochore complex component, homolog (S. cerevisiae) |
| 79019 | CENPM | centromere protein M |
| 1063 | CENPF | centromere protein F, 350/400ka (mitosin) |
| 1058 | CENPA | centromere protein A |
| 10403 | NDC80 | NDC80 homolog, kinetochore complex component (S. cerevisiae) |
| 83540 | NUF2 | NUF2, NDC80 kinetochore complex component, homolog (S. cerevisiae) |
| 80152 | CENPT | centromere protein T |

  
  
**MSCs batch 1 repressed and**GO:0005578**: 24 genes, expected 7.81152, P=1.24583e-06, P adjusted = 0.145029

|  |  |  |
| --- | --- | --- |
| 1301 | COL11A1 | collagen, type XI, alpha 1 |
| 1284 | COL4A2 | collagen, type IV, alpha 2 |
| 2200 | FBN1 | fibrillin 1 |
| 4015 | LOX | lysyl oxidase |
| 1307 | COL16A1 | collagen, type XVI, alpha 1 |
| 5549 | PRELP | proline/arginine-rich end leucine-rich repeat protein |
| 176 | ACAN | aggrecan |
| 3339 | HSPG2 | heparan sulfate proteoglycan 2 |
| 1303 | COL12A1 | collagen, type XII, alpha 1 |
| 8076 | MFAP5 | microfibrillar associated protein 5 |
| 2202 | EFEMP1 | EGF-containing fibulin-like extracellular matrix protein 1 |
| 2817 | GPC1 | glypican 1 |
| 633 | BGN | biglycan |
| 30008 | EFEMP2 | EGF-containing fibulin-like extracellular matrix protein 2 |
| 7078 | TIMP3 | TIMP metallopeptidase inhibitor 3 |
| 283 | ANG | angiogenin, ribonuclease, RNase A family, 5 |
| 1292 | COL6A2 | collagen, type VI, alpha 2 |
| 1893 | ECM1 | extracellular matrix protein 1 |
| 1404 | HAPLN1 | hyaluronan and proteoglycan link protein 1 |
| 8532 | CPZ | carboxypeptidase Z |
| 1300 | COL10A1 | collagen, type X, alpha 1 |
| 1116 | CHI3L1 | chitinase 3-like 1 (cartilage glycoprotein-39) |
| 3908 | LAMA2 | laminin, alpha 2 |
| 4237 | MFAP2 | microfibrillar-associated protein 2 |

  
  
**MSCs batch 1 repressed and**GO:0008652**: 9 genes, expected 1.13918, P=1.49334e-06, P adjusted = 0.173843

|  |  |  |
| --- | --- | --- |
| 440 | ASNS | asparagine synthetase |
| 587 | BCAT2 | branched chain aminotransferase 2, mitochondrial |
| 29968 | PSAT1 | phosphoserine aminotransferase 1 |
| 875 | CBS | cystathionine-beta-synthase |
| 26227 | PHGDH | phosphoglycerate dehydrogenase |
| 65263 | PYCRL | pyrroline-5-carboxylate reductase-like |
| 435 | ASL | argininosuccinate lyase |
| 5351 | PLOD1 | procollagen-lysine 1, 2-oxoglutarate 5-dioxygenase 1 |
| 5831 | PYCR1 | pyrroline-5-carboxylate reductase 1 |

  
  
**MSCs batch 1 repressed and**GO:0019320**: 11 genes, expected 1.87151, P=2.32247e-06, P adjusted = 0.270364

|  |  |  |
| --- | --- | --- |
| 226 | ALDOA | aldolase A, fructose-bisphosphate |
| 5230 | PGK1 | phosphoglycerate kinase 1 |
| 5214 | PFKP | phosphofructokinase, platelet |
| 7167 | TPI1 | triosephosphate isomerase 1 |
| 2023 | ENO1 | enolase 1, (alpha) |
| 5211 | PFKL | phosphofructokinase, liver |
| 25796 | PGLS | 6-phosphogluconolactonase |
| 2597 | GAPDH | glyceraldehyde-3-phosphate dehydrogenase |
| 2821 | GPI | glucose phosphate isomerase |
| 2026 | ENO2 | enolase 2 (gamma, neuronal) |
| 230 | ALDOC | aldolase C, fructose-bisphosphate |

  
  
**MSCs batch 1 repressed and**GO:0004720**: 4 genes, expected 0.135617, P=2.61968e-06, P adjusted = 0.304962

|  |  |  |
| --- | --- | --- |
| 4015 | LOX | lysyl oxidase |
| 4016 | LOXL1 | lysyl oxidase-like 1 |
| 4017 | LOXL2 | lysyl oxidase-like 2 |
| 84171 | LOXL4 | lysyl oxidase-like 4 |

  
  
**MSCs batch 1 repressed and**GO:0046365**: 11 genes, expected 1.89863, P=2.68888e-06, P adjusted = 0.313018

|  |  |  |
| --- | --- | --- |
| 226 | ALDOA | aldolase A, fructose-bisphosphate |
| 5230 | PGK1 | phosphoglycerate kinase 1 |
| 5214 | PFKP | phosphofructokinase, platelet |
| 7167 | TPI1 | triosephosphate isomerase 1 |
| 2023 | ENO1 | enolase 1, (alpha) |
| 5211 | PFKL | phosphofructokinase, liver |
| 25796 | PGLS | 6-phosphogluconolactonase |
| 2597 | GAPDH | glyceraldehyde-3-phosphate dehydrogenase |
| 2821 | GPI | glucose phosphate isomerase |
| 2026 | ENO2 | enolase 2 (gamma, neuronal) |
| 230 | ALDOC | aldolase C, fructose-bisphosphate |

  
  
**MSCs batch 1 repressed and**GO:0044430**: 42 genes, expected 19.5017, P=2.82694e-06, P adjusted = 0.32909

|  |  |  |
| --- | --- | --- |
| 9787 | DLGAP5 | discs, large (Drosophila) homolog-associated protein 5 |
| 10112 | KIF20A | kinesin family member 20A |
| 9814 | SFI1 | Sfi1 homolog, spindle assembly associated (yeast) |
| 4628 | MYH10 | myosin, heavy chain 10, non-muscle |
| 701 | BUB1B | BUB1 budding uninhibited by benzimidazoles 1 homolog beta (yeast) |
| 57153 | SLC44A2 | solute carrier family 44, member 2 |
| 23265 | EXOC7 | exocyst complex component 7 |
| 3883 | KRT33A | keratin 33A |
| 89796 | NAV1 | neuron navigator 1 |
| 26502 | NARF | nuclear prelamin A recognition factor |
| 822 | CAPG | capping protein (actin filament), gelsolin-like |
| 7272 | TTK | TTK protein kinase |
| 5411 | PNN | pinin, desmosome associated protein |
| 54443 | ANLN | anillin, actin binding protein |
| 10460 | TACC3 | transforming, acidic coiled-coil containing protein 3 |
| 10615 | SPAG5 | sperm associated antigen 5 |
| 4000 | LMNA | lamin A/C |
| 7153 | TOP2A | topoisomerase (DNA) II alpha 170kDa |
| 9055 | PRC1 | protein regulator of cytokinesis 1 |
| 6509 | SLC1A4 | solute carrier family 1 (glutamate/neutral amino acid transporter), member 4 |
| 4644 | MYO5A | myosin VA (heavy chain 12, myoxin) |
| 11004 | KIF2C | kinesin family member 2C |
| 3885 | KRT34 | keratin 34 |
| 332 | BIRC5 | baculoviral IAP repeat-containing 5 |
| 91978 | C19orf20 | chromosome 19 open reading frame 20 |
| 5531 | PPP4C | protein phosphatase 4 (formerly X), catalytic subunit |
| 4641 | MYO1C | myosin IC |
| 259266 | ASPM | asp (abnormal spindle) homolog, microcephaly associated (Drosophila) |
| 9928 | KIF14 | kinesin family member 14 |
| 4751 | NEK2 | NIMA (never in mitosis gene a)-related kinase 2 |
| 22974 | TPX2 | TPX2, microtubule-associated, homolog (Xenopus laevis) |
| 51203 | NUSAP1 | nucleolar and spindle associated protein 1 |
| 1063 | CENPF | centromere protein F, 350/400ka (mitosin) |
| 2312 | FLG | filaggrin |
| 4134 | MAP4 | microtubule-associated protein 4 |
| 4739 | NEDD9 | neural precursor cell expressed, developmentally down-regulated 9 |
| 8318 | CDC45L | CDC45 cell division cycle 45-like (S. cerevisiae) |
| 983 | CDC2 | cell division cycle 2, G1 to S and G2 to M |
| 3855 | KRT7 | keratin 7 |
| 7430 | EZR | ezrin |
| 991 | CDC20 | cell division cycle 20 homolog (S. cerevisiae) |
| 10398 | MYL9 | myosin, light chain 9, regulatory |

  
  
**MSCs batch 1 repressed and**GO:0032395**: 5 genes, expected 0.271233, P=3.24524e-06, P adjusted = 0.377785

|  |  |  |
| --- | --- | --- |
| 3126 | HLA-DRB4 | major histocompatibility complex, class II, DR beta 4 |
| 3119 | HLA-DQB1 | major histocompatibility complex, class II, DQ beta 1 |
| 3113 | HLA-DPA1 | major histocompatibility complex, class II, DP alpha 1 |
| 3108 | HLA-DMA | major histocompatibility complex, class II, DM alpha |
| 3122 | HLA-DRA | major histocompatibility complex, class II, DR alpha |

  
  
**MSCs batch 1 repressed and**GO:0046164**: 11 genes, expected 2.00713, P=4.71001e-06, P adjusted = 0.548302

|  |  |  |
| --- | --- | --- |
| 226 | ALDOA | aldolase A, fructose-bisphosphate |
| 5230 | PGK1 | phosphoglycerate kinase 1 |
| 5214 | PFKP | phosphofructokinase, platelet |
| 7167 | TPI1 | triosephosphate isomerase 1 |
| 2023 | ENO1 | enolase 1, (alpha) |
| 5211 | PFKL | phosphofructokinase, liver |
| 25796 | PGLS | 6-phosphogluconolactonase |
| 2597 | GAPDH | glyceraldehyde-3-phosphate dehydrogenase |
| 2821 | GPI | glucose phosphate isomerase |
| 2026 | ENO2 | enolase 2 (gamma, neuronal) |
| 230 | ALDOC | aldolase C, fructose-bisphosphate |

  
  
**MSCs batch 1 repressed and**GO:0044421**: 42 genes, expected 20.0441, P=5.58369e-06, P adjusted = 0.650009

|  |  |  |
| --- | --- | --- |
| 1301 | COL11A1 | collagen, type XI, alpha 1 |
| 11341 | SCRG1 | scrapie responsive protein 1 |
| 1284 | COL4A2 | collagen, type IV, alpha 2 |
| 2200 | FBN1 | fibrillin 1 |
| 114898 | C1QTNF2 | C1q and tumor necrosis factor related protein 2 |
| 3486 | IGFBP3 | insulin-like growth factor binding protein 3 |
| 4015 | LOX | lysyl oxidase |
| 1307 | COL16A1 | collagen, type XVI, alpha 1 |
| 5549 | PRELP | proline/arginine-rich end leucine-rich repeat protein |
| 176 | ACAN | aggrecan |
| 3339 | HSPG2 | heparan sulfate proteoglycan 2 |
| 4017 | LOXL2 | lysyl oxidase-like 2 |
| 1303 | COL12A1 | collagen, type XII, alpha 1 |
| 4282 | MIF | macrophage migration inhibitory factor (glycosylation-inhibiting factor) |
| 8076 | MFAP5 | microfibrillar associated protein 5 |
| 2202 | EFEMP1 | EGF-containing fibulin-like extracellular matrix protein 1 |
| 1906 | EDN1 | endothelin 1 |
| 2817 | GPC1 | glypican 1 |
| 633 | BGN | biglycan |
| 30008 | EFEMP2 | EGF-containing fibulin-like extracellular matrix protein 2 |
| 3485 | IGFBP2 | insulin-like growth factor binding protein 2, 36kDa |
| 5125 | PCSK5 | proprotein convertase subtilisin/kexin type 5 |
| 7078 | TIMP3 | TIMP metallopeptidase inhibitor 3 |
| 283 | ANG | angiogenin, ribonuclease, RNase A family, 5 |
| 5552 | SRGN | serglycin |
| 6050 | RNH1 | ribonuclease/angiogenin inhibitor 1 |
| 1292 | COL6A2 | collagen, type VI, alpha 2 |
| 2821 | GPI | glucose phosphate isomerase |
| 222663 | SCUBE3 | signal peptide, CUB domain, EGF-like 3 |
| 1893 | ECM1 | extracellular matrix protein 1 |
| 1404 | HAPLN1 | hyaluronan and proteoglycan link protein 1 |
| 5764 | PTN | pleiotrophin |
| 8532 | CPZ | carboxypeptidase Z |
| 6781 | STC1 | stanniocalcin 1 |
| 3956 | LGALS1 | lectin, galactoside-binding, soluble, 1 |
| 1300 | COL10A1 | collagen, type X, alpha 1 |
| 5176 | SERPINF1 | serpin peptidase inhibitor, clade F (alpha-2 antiplasmin, pigment epithelium derived factor), member 1 |
| 1116 | CHI3L1 | chitinase 3-like 1 (cartilage glycoprotein-39) |
| 6424 | SFRP4 | secreted frizzled-related protein 4 |
| 3908 | LAMA2 | laminin, alpha 2 |
| 4237 | MFAP2 | microfibrillar-associated protein 2 |
| 649 | BMP1 | bone morphogenetic protein 1 |

  
  
**MSCs batch 1 repressed and**GO:0042613**: 5 genes, expected 0.298357, P=5.81674e-06, P adjusted = 0.677138

|  |  |  |
| --- | --- | --- |
| 3126 | HLA-DRB4 | major histocompatibility complex, class II, DR beta 4 |
| 3119 | HLA-DQB1 | major histocompatibility complex, class II, DQ beta 1 |
| 3113 | HLA-DPA1 | major histocompatibility complex, class II, DP alpha 1 |
| 3108 | HLA-DMA | major histocompatibility complex, class II, DM alpha |
| 3122 | HLA-DRA | major histocompatibility complex, class II, DR alpha |

  
  
**MSCs batch 1 repressed and**GO:0007155**: 41 genes, expected 19.5559, P=7.13873e-06, P adjusted = 0.831034

|  |  |  |
| --- | --- | --- |
| 1301 | COL11A1 | collagen, type XI, alpha 1 |
| 3679 | ITGA7 | integrin, alpha 7 |
| 58494 | JAM2 | junctional adhesion molecule 2 |
| 51148 | CERCAM | cerebral endothelial cell adhesion molecule |
| 6275 | S100A4 | S100 calcium binding protein A4 |
| 1307 | COL16A1 | collagen, type XVI, alpha 1 |
| 176 | ACAN | aggrecan |
| 3339 | HSPG2 | heparan sulfate proteoglycan 2 |
| 4017 | LOXL2 | lysyl oxidase-like 2 |
| 1303 | COL12A1 | collagen, type XII, alpha 1 |
| 5411 | PNN | pinin, desmosome associated protein |
| 3678 | ITGA5 | integrin, alpha 5 (fibronectin receptor, alpha polypeptide) |
| 7059 | THBS3 | thrombospondin 3 |
| 7070 | THY1 | Thy-1 cell surface antigen |
| 2022 | ENG | endoglin |
| 23114 | NFASC | neurofascin homolog (chicken) |
| 3689 | ITGB2 | integrin, beta 2 (complement component 3 receptor 3 and 4 subunit) |
| 4685 | NCAM2 | neural cell adhesion molecule 2 |
| 4684 | NCAM1 | neural cell adhesion molecule 1 |
| 3381 | IBSP | integrin-binding sialoprotein |
| 9358 | ITGBL1 | integrin, beta-like 1 (with EGF-like repeat domains) |
| 1292 | COL6A2 | collagen, type VI, alpha 2 |
| 976 | CD97 | CD97 molecule |
| 948 | CD36 | CD36 molecule (thrombospondin receptor) |
| 1404 | HAPLN1 | hyaluronan and proteoglycan link protein 1 |
| 58986 | TMEM8 | transmembrane protein 8 (five membrane-spanning domains) |
| 977 | CD151 | CD151 molecule (Raph blood group) |
| 4739 | NEDD9 | neural precursor cell expressed, developmentally down-regulated 9 |
| 9379 | NRXN2 | neurexin 2 |
| 5792 | PTPRF | protein tyrosine phosphatase, receptor type, F |
| 8515 | ITGA10 | integrin, alpha 10 |
| 3675 | ITGA3 | integrin, alpha 3 (antigen CD49C, alpha 3 subunit of VLA-3 receptor) |
| 55742 | PARVA | parvin, alpha |
| 7430 | EZR | ezrin |
| 5819 | PVRL2 | poliovirus receptor-related 2 (herpesvirus entry mediator B) |
| 57575 | PCDH10 | protocadherin 10 |
| 7057 | THBS1 | thrombospondin 1 |
| 165 | AEBP1 | AE binding protein 1 |
| 3908 | LAMA2 | laminin, alpha 2 |
| 22801 | ITGA11 | integrin, alpha 11 |
| 649 | BMP1 | bone morphogenetic protein 1 |

  
  
**MSCs batch 1 repressed and**GO:0022610**: 41 genes, expected 19.5559, P=7.13873e-06, P adjusted = 0.831034

|  |  |  |
| --- | --- | --- |
| 1301 | COL11A1 | collagen, type XI, alpha 1 |
| 3679 | ITGA7 | integrin, alpha 7 |
| 58494 | JAM2 | junctional adhesion molecule 2 |
| 51148 | CERCAM | cerebral endothelial cell adhesion molecule |
| 6275 | S100A4 | S100 calcium binding protein A4 |
| 1307 | COL16A1 | collagen, type XVI, alpha 1 |
| 176 | ACAN | aggrecan |
| 3339 | HSPG2 | heparan sulfate proteoglycan 2 |
| 4017 | LOXL2 | lysyl oxidase-like 2 |
| 1303 | COL12A1 | collagen, type XII, alpha 1 |
| 5411 | PNN | pinin, desmosome associated protein |
| 3678 | ITGA5 | integrin, alpha 5 (fibronectin receptor, alpha polypeptide) |
| 7059 | THBS3 | thrombospondin 3 |
| 7070 | THY1 | Thy-1 cell surface antigen |
| 2022 | ENG | endoglin |
| 23114 | NFASC | neurofascin homolog (chicken) |
| 3689 | ITGB2 | integrin, beta 2 (complement component 3 receptor 3 and 4 subunit) |
| 4685 | NCAM2 | neural cell adhesion molecule 2 |
| 4684 | NCAM1 | neural cell adhesion molecule 1 |
| 3381 | IBSP | integrin-binding sialoprotein |
| 9358 | ITGBL1 | integrin, beta-like 1 (with EGF-like repeat domains) |
| 1292 | COL6A2 | collagen, type VI, alpha 2 |
| 976 | CD97 | CD97 molecule |
| 948 | CD36 | CD36 molecule (thrombospondin receptor) |
| 1404 | HAPLN1 | hyaluronan and proteoglycan link protein 1 |
| 58986 | TMEM8 | transmembrane protein 8 (five membrane-spanning domains) |
| 977 | CD151 | CD151 molecule (Raph blood group) |
| 4739 | NEDD9 | neural precursor cell expressed, developmentally down-regulated 9 |
| 9379 | NRXN2 | neurexin 2 |
| 5792 | PTPRF | protein tyrosine phosphatase, receptor type, F |
| 8515 | ITGA10 | integrin, alpha 10 |
| 3675 | ITGA3 | integrin, alpha 3 (antigen CD49C, alpha 3 subunit of VLA-3 receptor) |
| 55742 | PARVA | parvin, alpha |
| 7430 | EZR | ezrin |
| 5819 | PVRL2 | poliovirus receptor-related 2 (herpesvirus entry mediator B) |
| 57575 | PCDH10 | protocadherin 10 |
| 7057 | THBS1 | thrombospondin 1 |
| 165 | AEBP1 | AE binding protein 1 |
| 3908 | LAMA2 | laminin, alpha 2 |
| 22801 | ITGA11 | integrin, alpha 11 |
| 649 | BMP1 | bone morphogenetic protein 1 |

  
  
**MSCs batch 1 repressed and**GO:0008305**: 7 genes, expected 0.759454, P=7.49842e-06, P adjusted = 0.872906

|  |  |  |
| --- | --- | --- |
| 3679 | ITGA7 | integrin, alpha 7 |
| 3678 | ITGA5 | integrin, alpha 5 (fibronectin receptor, alpha polypeptide) |
| 3689 | ITGB2 | integrin, beta 2 (complement component 3 receptor 3 and 4 subunit) |
| 9358 | ITGBL1 | integrin, beta-like 1 (with EGF-like repeat domains) |
| 8515 | ITGA10 | integrin, alpha 10 |
| 3675 | ITGA3 | integrin, alpha 3 (antigen CD49C, alpha 3 subunit of VLA-3 receptor) |
| 22801 | ITGA11 | integrin, alpha 11 |

  
  
**MSCs batch 1 repressed and**GO:0005198**: 38 genes, expected 17.5759, P=7.77226e-06, P adjusted = 0.904784

|  |  |  |
| --- | --- | --- |
| 1301 | COL11A1 | collagen, type XI, alpha 1 |
| 6193 | RPS5 | ribosomal protein S5 |
| 1284 | COL4A2 | collagen, type IV, alpha 2 |
| 2200 | FBN1 | fibrillin 1 |
| 6159 | RPL29 | ribosomal protein L29 |
| 6137 | RPL13 | ribosomal protein L13 |
| 59 | ACTA2 | actin, alpha 2, smooth muscle, aorta |
| 3883 | KRT33A | keratin 33A |
| 1307 | COL16A1 | collagen, type XVI, alpha 1 |
| 5549 | PRELP | proline/arginine-rich end leucine-rich repeat protein |
| 176 | ACAN | aggrecan |
| 1303 | COL12A1 | collagen, type XII, alpha 1 |
| 5411 | PNN | pinin, desmosome associated protein |
| 51778 | MYOZ2 | myozenin 2 |
| 6203 | RPS9 | ribosomal protein S9 |
| 8076 | MFAP5 | microfibrillar associated protein 5 |
| 6676 | SPAG4 | sperm associated antigen 4 |
| 7059 | THBS3 | thrombospondin 3 |
| 633 | BGN | biglycan |
| 30008 | EFEMP2 | EGF-containing fibulin-like extracellular matrix protein 2 |
| 4000 | LMNA | lamin A/C |
| 64975 | MRPL41 | mitochondrial ribosomal protein L41 |
| 6150 | MRPL23 | mitochondrial ribosomal protein L23 |
| 3885 | KRT34 | keratin 34 |
| 1292 | COL6A2 | collagen, type VI, alpha 2 |
| 2312 | FLG | filaggrin |
| 8218 | CLTCL1 | clathrin, heavy chain-like 1 |
| 4134 | MAP4 | microtubule-associated protein 4 |
| 1300 | COL10A1 | collagen, type X, alpha 1 |
| 3855 | KRT7 | keratin 7 |
| 1116 | CHI3L1 | chitinase 3-like 1 (cartilage glycoprotein-39) |
| 7430 | EZR | ezrin |
| 7057 | THBS1 | thrombospondin 1 |
| 51073 | MRPL4 | mitochondrial ribosomal protein L4 |
| 6122 | RPL3 | ribosomal protein L3 |
| 11316 | COPE | coatomer protein complex, subunit epsilon |
| 3908 | LAMA2 | laminin, alpha 2 |
| 10398 | MYL9 | myosin, light chain 9, regulatory |

  
  
**MSCs batch 1 repressed and**GO:0044275**: 12 genes, expected 2.52247, P=7.94351e-06, P adjusted = 0.92472

|  |  |  |
| --- | --- | --- |
| 226 | ALDOA | aldolase A, fructose-bisphosphate |
| 5230 | PGK1 | phosphoglycerate kinase 1 |
| 5214 | PFKP | phosphofructokinase, platelet |
| 7167 | TPI1 | triosephosphate isomerase 1 |
| 2023 | ENO1 | enolase 1, (alpha) |
| 5211 | PFKL | phosphofructokinase, liver |
| 25796 | PGLS | 6-phosphogluconolactonase |
| 2597 | GAPDH | glyceraldehyde-3-phosphate dehydrogenase |
| 2821 | GPI | glucose phosphate isomerase |
| 2026 | ENO2 | enolase 2 (gamma, neuronal) |
| 1116 | CHI3L1 | chitinase 3-like 1 (cartilage glycoprotein-39) |
| 230 | ALDOC | aldolase C, fructose-bisphosphate |

  
  
**MSCs batch 1 repressed and**GO:0015630**: 30 genes, expected 12.5039, P=1.00837e-05, P adjusted = 1

|  |  |  |
| --- | --- | --- |
| 9787 | DLGAP5 | discs, large (Drosophila) homolog-associated protein 5 |
| 10112 | KIF20A | kinesin family member 20A |
| 9814 | SFI1 | Sfi1 homolog, spindle assembly associated (yeast) |
| 701 | BUB1B | BUB1 budding uninhibited by benzimidazoles 1 homolog beta (yeast) |
| 57153 | SLC44A2 | solute carrier family 44, member 2 |
| 23265 | EXOC7 | exocyst complex component 7 |
| 89796 | NAV1 | neuron navigator 1 |
| 7272 | TTK | TTK protein kinase |
| 10460 | TACC3 | transforming, acidic coiled-coil containing protein 3 |
| 10615 | SPAG5 | sperm associated antigen 5 |
| 7153 | TOP2A | topoisomerase (DNA) II alpha 170kDa |
| 9055 | PRC1 | protein regulator of cytokinesis 1 |
| 4644 | MYO5A | myosin VA (heavy chain 12, myoxin) |
| 11004 | KIF2C | kinesin family member 2C |
| 332 | BIRC5 | baculoviral IAP repeat-containing 5 |
| 91978 | C19orf20 | chromosome 19 open reading frame 20 |
| 5531 | PPP4C | protein phosphatase 4 (formerly X), catalytic subunit |
| 259266 | ASPM | asp (abnormal spindle) homolog, microcephaly associated (Drosophila) |
| 9928 | KIF14 | kinesin family member 14 |
| 4751 | NEK2 | NIMA (never in mitosis gene a)-related kinase 2 |
| 22974 | TPX2 | TPX2, microtubule-associated, homolog (Xenopus laevis) |
| 51203 | NUSAP1 | nucleolar and spindle associated protein 1 |
| 1063 | CENPF | centromere protein F, 350/400ka (mitosin) |
| 9133 | CCNB2 | cyclin B2 |
| 4134 | MAP4 | microtubule-associated protein 4 |
| 4739 | NEDD9 | neural precursor cell expressed, developmentally down-regulated 9 |
| 8318 | CDC45L | CDC45 cell division cycle 45-like (S. cerevisiae) |
| 983 | CDC2 | cell division cycle 2, G1 to S and G2 to M |
| 7430 | EZR | ezrin |
| 991 | CDC20 | cell division cycle 20 homolog (S. cerevisiae) |

  
  
**MSCs batch 1 repressed and**GO:0000776**: 9 genes, expected 1.43754, P=1.1385e-05, P adjusted = 1

|  |  |  |
| --- | --- | --- |
| 701 | BUB1B | BUB1 budding uninhibited by benzimidazoles 1 homolog beta (yeast) |
| 10615 | SPAG5 | sperm associated antigen 5 |
| 57405 | SPC25 | SPC25, NDC80 kinetochore complex component, homolog (S. cerevisiae) |
| 79019 | CENPM | centromere protein M |
| 1063 | CENPF | centromere protein F, 350/400ka (mitosin) |
| 1058 | CENPA | centromere protein A |
| 10403 | NDC80 | NDC80 homolog, kinetochore complex component (S. cerevisiae) |
| 83540 | NUF2 | NUF2, NDC80 kinetochore complex component, homolog (S. cerevisiae) |
| 80152 | CENPT | centromere protein T |

  
  
**MSCs batch 1 repressed and**GO:0005201**: 11 genes, expected 2.22411, P=1.29777e-05, P adjusted = 1

|  |  |  |
| --- | --- | --- |
| 1301 | COL11A1 | collagen, type XI, alpha 1 |
| 1284 | COL4A2 | collagen, type IV, alpha 2 |
| 2200 | FBN1 | fibrillin 1 |
| 5549 | PRELP | proline/arginine-rich end leucine-rich repeat protein |
| 176 | ACAN | aggrecan |
| 1303 | COL12A1 | collagen, type XII, alpha 1 |
| 8076 | MFAP5 | microfibrillar associated protein 5 |
| 633 | BGN | biglycan |
| 30008 | EFEMP2 | EGF-containing fibulin-like extracellular matrix protein 2 |
| 1292 | COL6A2 | collagen, type VI, alpha 2 |
| 1116 | CHI3L1 | chitinase 3-like 1 (cartilage glycoprotein-39) |

  
  
**MSCs batch 1 repressed and**GO:0045787**: 7 genes, expected 0.840824, P=1.55179e-05, P adjusted = 1

|  |  |  |
| --- | --- | --- |
| 9787 | DLGAP5 | discs, large (Drosophila) homolog-associated protein 5 |
| 440 | ASNS | asparagine synthetase |
| 1906 | EDN1 | endothelin 1 |
| 332 | BIRC5 | baculoviral IAP repeat-containing 5 |
| 51203 | NUSAP1 | nucleolar and spindle associated protein 1 |
| 11065 | UBE2C | ubiquitin-conjugating enzyme E2C |
| 595 | CCND1 | cyclin D1 |

  
  
**MSCs batch 1 repressed and**GO:0031577**: 4 genes, expected 0.189863, P=1.75571e-05, P adjusted = 1

|  |  |  |
| --- | --- | --- |
| 7272 | TTK | TTK protein kinase |
| 10459 | MAD2L2 | MAD2 mitotic arrest deficient-like 2 (yeast) |
| 332 | BIRC5 | baculoviral IAP repeat-containing 5 |
| 1063 | CENPF | centromere protein F, 350/400ka (mitosin) |

  
  
**MSCs batch 1 repressed and**GO:0007229**: 9 genes, expected 1.51891, P=1.81074e-05, P adjusted = 1

|  |  |  |
| --- | --- | --- |
| 3679 | ITGA7 | integrin, alpha 7 |
| 1307 | COL16A1 | collagen, type XVI, alpha 1 |
| 3678 | ITGA5 | integrin, alpha 5 (fibronectin receptor, alpha polypeptide) |
| 3689 | ITGB2 | integrin, beta 2 (complement component 3 receptor 3 and 4 subunit) |
| 9358 | ITGBL1 | integrin, beta-like 1 (with EGF-like repeat domains) |
| 4739 | NEDD9 | neural precursor cell expressed, developmentally down-regulated 9 |
| 8515 | ITGA10 | integrin, alpha 10 |
| 3675 | ITGA3 | integrin, alpha 3 (antigen CD49C, alpha 3 subunit of VLA-3 receptor) |
| 22801 | ITGA11 | integrin, alpha 11 |

  
  
**MSCs batch 1 repressed and**GO:0000775**: 11 genes, expected 2.30548, P=1.83777e-05, P adjusted = 1

|  |  |  |
| --- | --- | --- |
| 701 | BUB1B | BUB1 budding uninhibited by benzimidazoles 1 homolog beta (yeast) |
| 10615 | SPAG5 | sperm associated antigen 5 |
| 11004 | KIF2C | kinesin family member 2C |
| 57405 | SPC25 | SPC25, NDC80 kinetochore complex component, homolog (S. cerevisiae) |
| 332 | BIRC5 | baculoviral IAP repeat-containing 5 |
| 79019 | CENPM | centromere protein M |
| 1063 | CENPF | centromere protein F, 350/400ka (mitosin) |
| 1058 | CENPA | centromere protein A |
| 10403 | NDC80 | NDC80 homolog, kinetochore complex component (S. cerevisiae) |
| 83540 | NUF2 | NUF2, NDC80 kinetochore complex component, homolog (S. cerevisiae) |
| 80152 | CENPT | centromere protein T |

  
  
**MSCs batch 1 repressed and**GO:0016052**: 12 genes, expected 2.76658, P=2.06565e-05, P adjusted = 1

|  |  |  |
| --- | --- | --- |
| 226 | ALDOA | aldolase A, fructose-bisphosphate |
| 5230 | PGK1 | phosphoglycerate kinase 1 |
| 5214 | PFKP | phosphofructokinase, platelet |
| 7167 | TPI1 | triosephosphate isomerase 1 |
| 2023 | ENO1 | enolase 1, (alpha) |
| 5211 | PFKL | phosphofructokinase, liver |
| 25796 | PGLS | 6-phosphogluconolactonase |
| 2597 | GAPDH | glyceraldehyde-3-phosphate dehydrogenase |
| 2821 | GPI | glucose phosphate isomerase |
| 2026 | ENO2 | enolase 2 (gamma, neuronal) |
| 1116 | CHI3L1 | chitinase 3-like 1 (cartilage glycoprotein-39) |
| 230 | ALDOC | aldolase C, fructose-bisphosphate |

  
  
**MSCs batch 1 repressed and**GO:0016051**: 12 genes, expected 2.7937, P=2.28142e-05, P adjusted = 1

|  |  |  |
| --- | --- | --- |
| 5106 | PCK2 | phosphoenolpyruvate carboxykinase 2 (mitochondrial) |
| 51148 | CERCAM | cerebral endothelial cell adhesion molecule |
| 8707 | B3GALT2 | UDP-Gal:betaGlcNAc beta 1,3-galactosyltransferase, polypeptide 2 |
| 114898 | C1QTNF2 | C1q and tumor necrosis factor related protein 2 |
| 176 | ACAN | aggrecan |
| 2132 | EXT2 | exostoses (multiple) 2 |
| 7167 | TPI1 | triosephosphate isomerase 1 |
| 2821 | GPI | glucose phosphate isomerase |
| 5372 | PMM1 | phosphomannomutase 1 |
| 11285 | B4GALT7 | xylosylprotein beta 1,4-galactosyltransferase, polypeptide 7 (galactosyltransferase I) |
| 2997 | GYS1 | glycogen synthase 1 (muscle) |
| 55501 | CHST12 | carbohydrate (chondroitin 4) sulfotransferase 12 |

  
  
**MSCs batch 1 repressed and**GO:0002504**: 5 genes, expected 0.379727, P=2.35566e-05, P adjusted = 1

|  |  |  |
| --- | --- | --- |
| 3126 | HLA-DRB4 | major histocompatibility complex, class II, DR beta 4 |
| 3119 | HLA-DQB1 | major histocompatibility complex, class II, DQ beta 1 |
| 3113 | HLA-DPA1 | major histocompatibility complex, class II, DP alpha 1 |
| 3108 | HLA-DMA | major histocompatibility complex, class II, DM alpha |
| 3122 | HLA-DRA | major histocompatibility complex, class II, DR alpha |

  
  
**MSCs batch 1 repressed and**GO:0048513**: 60 genes, expected 34.745, P=2.53361e-05, P adjusted = 1

|  |  |  |
| --- | --- | --- |
| 3778 | KCNMA1 | potassium large conductance calcium-activated channel, subfamily M, alpha member 1 |
| 23363 | OBSL1 | obscurin-like 1 |
| 6876 | TAGLN | transgelin |
| 3625 | INHBB | inhibin, beta B |
| 1301 | COL11A1 | collagen, type XI, alpha 1 |
| 3679 | ITGA7 | integrin, alpha 7 |
| 10014 | HDAC5 | histone deacetylase 5 |
| 1284 | COL4A2 | collagen, type IV, alpha 2 |
| 2200 | FBN1 | fibrillin 1 |
| 6300 | MAPK12 | mitogen-activated protein kinase 12 |
| 5307 | PITX1 | paired-like homeodomain 1 |
| 3486 | IGFBP3 | insulin-like growth factor binding protein 3 |
| 290 | ANPEP | alanyl (membrane) aminopeptidase |
| 4015 | LOX | lysyl oxidase |
| 5549 | PRELP | proline/arginine-rich end leucine-rich repeat protein |
| 176 | ACAN | aggrecan |
| 1303 | COL12A1 | collagen, type XII, alpha 1 |
| 93185 | IGSF8 | immunoglobulin superfamily, member 8 |
| 5054 | SERPINE1 | serpin peptidase inhibitor, clade E (nexin, plasminogen activator inhibitor type 1), member 1 |
| 6444 | SGCD | sarcoglycan, delta (35kDa dystrophin-associated glycoprotein) |
| 6640 | SNTA1 | syntrophin, alpha 1 (dystrophin-associated protein A1, 59kDa, acidic component) |
| 10460 | TACC3 | transforming, acidic coiled-coil containing protein 3 |
| 1906 | EDN1 | endothelin 1 |
| 4776 | NFATC4 | nuclear factor of activated T-cells, cytoplasmic, calcineurin-dependent 4 |
| 7070 | THY1 | Thy-1 cell surface antigen |
| 2022 | ENG | endoglin |
| 1948 | EFNB2 | ephrin-B2 |
| 2132 | EXT2 | exostoses (multiple) 2 |
| 5125 | PCSK5 | proprotein convertase subtilisin/kexin type 5 |
| 8613 | PPAP2B | phosphatidic acid phosphatase type 2B |
| 283 | ANG | angiogenin, ribonuclease, RNase A family, 5 |
| 5552 | SRGN | serglycin |
| 2067 | ERCC1 | excision repair cross-complementing rodent repair deficiency, complementation group 1 (includes overlapping antisense sequence) |
| 6050 | RNH1 | ribonuclease/angiogenin inhibitor 1 |
| 51466 | EVL | Enah/Vasp-like |
| 3381 | IBSP | integrin-binding sialoprotein |
| 1464 | CSPG4 | chondroitin sulfate proteoglycan 4 |
| 3575 | IL7R | interleukin 7 receptor |
| 972 | CD74 | CD74 molecule, major histocompatibility complex, class II invariant chain |
| 3885 | KRT34 | keratin 34 |
| 56033 | BARX1 | BARX homeobox 1 |
| 26227 | PHGDH | phosphoglycerate dehydrogenase |
| 3108 | HLA-DMA | major histocompatibility complex, class II, DM alpha |
| 259266 | ASPM | asp (abnormal spindle) homolog, microcephaly associated (Drosophila) |
| 3084 | NRG1 | neuregulin 1 |
| 1063 | CENPF | centromere protein F, 350/400ka (mitosin) |
| 5764 | PTN | pleiotrophin |
| 4232 | MEST | mesoderm specific transcript homolog (mouse) |
| 3956 | LGALS1 | lectin, galactoside-binding, soluble, 1 |
| 6604 | SMARCD3 | SWI/SNF related, matrix associated, actin dependent regulator of chromatin, subfamily d, member 3 |
| 2273 | FHL1 | four and a half LIM domains 1 |
| 1300 | COL10A1 | collagen, type X, alpha 1 |
| 5176 | SERPINF1 | serpin peptidase inhibitor, clade F (alpha-2 antiplasmin, pigment epithelium derived factor), member 1 |
| 51196 | PLCE1 | phospholipase C, epsilon 1 |
| 2997 | GYS1 | glycogen synthase 1 (muscle) |
| 5351 | PLOD1 | procollagen-lysine 1, 2-oxoglutarate 5-dioxygenase 1 |
| 3908 | LAMA2 | laminin, alpha 2 |
| 4054 | LTBP3 | latent transforming growth factor beta binding protein 3 |
| 22801 | ITGA11 | integrin, alpha 11 |
| 649 | BMP1 | bone morphogenetic protein 1 |

  
  
**MSCs batch 1 repressed and**GO:0000226**: 12 genes, expected 2.84795, P=2.77247e-05, P adjusted = 1

|  |  |  |
| --- | --- | --- |
| 701 | BUB1B | BUB1 budding uninhibited by benzimidazoles 1 homolog beta (yeast) |
| 89796 | NAV1 | neuron navigator 1 |
| 7272 | TTK | TTK protein kinase |
| 10615 | SPAG5 | sperm associated antigen 5 |
| 9055 | PRC1 | protein regulator of cytokinesis 1 |
| 11004 | KIF2C | kinesin family member 2C |
| 91978 | C19orf20 | chromosome 19 open reading frame 20 |
| 5531 | PPP4C | protein phosphatase 4 (formerly X), catalytic subunit |
| 51203 | NUSAP1 | nucleolar and spindle associated protein 1 |
| 10403 | NDC80 | NDC80 homolog, kinetochore complex component (S. cerevisiae) |
| 4134 | MAP4 | microtubule-associated protein 4 |
| 11065 | UBE2C | ubiquitin-conjugating enzyme E2C |

  
  
**MSCs batch 1 repressed and**GO:0006563**: 4 genes, expected 0.216987, P=3.43597e-05, P adjusted = 1

|  |  |  |
| --- | --- | --- |
| 6472 | SHMT2 | serine hydroxymethyltransferase 2 (mitochondrial) |
| 29968 | PSAT1 | phosphoserine aminotransferase 1 |
| 875 | CBS | cystathionine-beta-synthase |
| 26227 | PHGDH | phosphoglycerate dehydrogenase |

  
  
**MSCs batch 1 repressed and**GO:0045840**: 5 genes, expected 0.40685, P=3.45478e-05, P adjusted = 1

|  |  |  |
| --- | --- | --- |
| 9787 | DLGAP5 | discs, large (Drosophila) homolog-associated protein 5 |
| 1906 | EDN1 | endothelin 1 |
| 332 | BIRC5 | baculoviral IAP repeat-containing 5 |
| 51203 | NUSAP1 | nucleolar and spindle associated protein 1 |
| 11065 | UBE2C | ubiquitin-conjugating enzyme E2C |

  
  
**MSCs batch 1 repressed and**GO:0034637**: 11 genes, expected 2.49535, P=3.90364e-05, P adjusted = 1

|  |  |  |
| --- | --- | --- |
| 5106 | PCK2 | phosphoenolpyruvate carboxykinase 2 (mitochondrial) |
| 51148 | CERCAM | cerebral endothelial cell adhesion molecule |
| 114898 | C1QTNF2 | C1q and tumor necrosis factor related protein 2 |
| 176 | ACAN | aggrecan |
| 2132 | EXT2 | exostoses (multiple) 2 |
| 7167 | TPI1 | triosephosphate isomerase 1 |
| 2821 | GPI | glucose phosphate isomerase |
| 5372 | PMM1 | phosphomannomutase 1 |
| 11285 | B4GALT7 | xylosylprotein beta 1,4-galactosyltransferase, polypeptide 7 (galactosyltransferase I) |
| 2997 | GYS1 | glycogen synthase 1 (muscle) |
| 55501 | CHST12 | carbohydrate (chondroitin 4) sulfotransferase 12 |

  
  
**MSCs batch 1 repressed and**GO:0051726**: 22 genes, expected 8.5981, P=5.94159e-05, P adjusted = 1

|  |  |  |
| --- | --- | --- |
| 9787 | DLGAP5 | discs, large (Drosophila) homolog-associated protein 5 |
| 701 | BUB1B | BUB1 budding uninhibited by benzimidazoles 1 homolog beta (yeast) |
| 440 | ASNS | asparagine synthetase |
| 7272 | TTK | TTK protein kinase |
| 5411 | PNN | pinin, desmosome associated protein |
| 54443 | ANLN | anillin, actin binding protein |
| 10460 | TACC3 | transforming, acidic coiled-coil containing protein 3 |
| 1906 | EDN1 | endothelin 1 |
| 10459 | MAD2L2 | MAD2 mitotic arrest deficient-like 2 (yeast) |
| 2132 | EXT2 | exostoses (multiple) 2 |
| 8558 | CDK10 | cyclin-dependent kinase 10 |
| 112464 | PRKCDBP | protein kinase C, delta binding protein |
| 1033 | CDKN3 | cyclin-dependent kinase inhibitor 3 |
| 29108 | PYCARD | PYD and CARD domain containing |
| 332 | BIRC5 | baculoviral IAP repeat-containing 5 |
| 4751 | NEK2 | NIMA (never in mitosis gene a)-related kinase 2 |
| 51203 | NUSAP1 | nucleolar and spindle associated protein 1 |
| 1063 | CENPF | centromere protein F, 350/400ka (mitosin) |
| 8318 | CDC45L | CDC45 cell division cycle 45-like (S. cerevisiae) |
| 11065 | UBE2C | ubiquitin-conjugating enzyme E2C |
| 595 | CCND1 | cyclin D1 |
| 890 | CCNA2 | cyclin A2 |

  
  
**MSCs batch 1 repressed and**GO:0044271**: 11 genes, expected 2.65809, P=7.03091e-05, P adjusted = 1

|  |  |  |
| --- | --- | --- |
| 440 | ASNS | asparagine synthetase |
| 587 | BCAT2 | branched chain aminotransferase 2, mitochondrial |
| 5025 | P2RX4 | purinergic receptor P2X, ligand-gated ion channel, 4 |
| 1906 | EDN1 | endothelin 1 |
| 29968 | PSAT1 | phosphoserine aminotransferase 1 |
| 875 | CBS | cystathionine-beta-synthase |
| 26227 | PHGDH | phosphoglycerate dehydrogenase |
| 65263 | PYCRL | pyrroline-5-carboxylate reductase-like |
| 435 | ASL | argininosuccinate lyase |
| 5351 | PLOD1 | procollagen-lysine 1, 2-oxoglutarate 5-dioxygenase 1 |
| 5831 | PYCR1 | pyrroline-5-carboxylate reductase 1 |

  
  
**MSCs batch 1 repressed and**GO:0005604**: 8 genes, expected 1.41041, P=7.32348e-05, P adjusted = 1

|  |  |  |
| --- | --- | --- |
| 1284 | COL4A2 | collagen, type IV, alpha 2 |
| 2200 | FBN1 | fibrillin 1 |
| 176 | ACAN | aggrecan |
| 3339 | HSPG2 | heparan sulfate proteoglycan 2 |
| 30008 | EFEMP2 | EGF-containing fibulin-like extracellular matrix protein 2 |
| 7078 | TIMP3 | TIMP metallopeptidase inhibitor 3 |
| 283 | ANG | angiogenin, ribonuclease, RNase A family, 5 |
| 3908 | LAMA2 | laminin, alpha 2 |

  
  
**MSCs batch 1 repressed and**GO:0055114**: 32 genes, expected 15.4061, P=8.66029e-05, P adjusted = 1

|  |  |  |
| --- | --- | --- |
| 147015 | DHRS13 | dehydrogenase/reductase (SDR family) member 13 |
| 10873 | ME3 | malic enzyme 3, NADP(+)-dependent, mitochondrial |
| 84795 | C10orf33 | chromosome 10 open reading frame 33 |
| 79001 | VKORC1 | vitamin K epoxide reductase complex, subunit 1 |
| 4015 | LOX | lysyl oxidase |
| 4016 | LOXL1 | lysyl oxidase-like 1 |
| 7001 | PRDX2 | peroxiredoxin 2 |
| 6241 | RRM2 | ribonucleotide reductase M2 polypeptide |
| 4017 | LOXL2 | lysyl oxidase-like 2 |
| 645 | BLVRB | biliverdin reductase B (flavin reductase (NADPH)) |
| 4713 | NDUFB7 | NADH dehydrogenase (ubiquinone) 1 beta subcomplex, 7, 18kDa |
| 4714 | NDUFB8 | NADH dehydrogenase (ubiquinone) 1 beta subcomplex, 8, 19kDa |
| 10536 | LEPREL2 | leprecan-like 2 |
| 2597 | GAPDH | glyceraldehyde-3-phosphate dehydrogenase |
| 26227 | PHGDH | phosphoglycerate dehydrogenase |
| 84171 | LOXL4 | lysyl oxidase-like 4 |
| 1535 | CYBA | cytochrome b-245, alpha polypeptide |
| 5959 | RDH5 | retinol dehydrogenase 5 (11-cis/9-cis) |
| 55214 | LEPREL1 | leprecan-like 1 |
| 65263 | PYCRL | pyrroline-5-carboxylate reductase-like |
| 3418 | IDH2 | isocitrate dehydrogenase 2 (NADP+), mitochondrial |
| 3421 | IDH3G | isocitrate dehydrogenase 3 (NAD+) gamma |
| 8574 | AKR7A2 | aldo-keto reductase family 7, member A2 (aflatoxin aldehyde reductase) |
| 3995 | FADS3 | fatty acid desaturase 3 |
| 374291 | NDUFS7 | NADH dehydrogenase (ubiquinone) Fe-S protein 7, 20kDa (NADH-coenzyme Q reductase) |
| 23030 | JMJD2B | jumonji domain containing 2B |
| 57407 | NMRAL1 | NmrA-like family domain containing 1 |
| 7384 | UQCRC1 | ubiquinol-cytochrome c reductase core protein I |
| 5351 | PLOD1 | procollagen-lysine 1, 2-oxoglutarate 5-dioxygenase 1 |
| 9524 | GPSN2 | glycoprotein, synaptic 2 |
| 5831 | PYCR1 | pyrroline-5-carboxylate reductase 1 |
| 51734 | SEPX1 | selenoprotein X, 1 |

  
  
**MSCs batch 1 repressed and**GO:0007051**: 6 genes, expected 0.759454, P=8.77526e-05, P adjusted = 1

|  |  |  |
| --- | --- | --- |
| 701 | BUB1B | BUB1 budding uninhibited by benzimidazoles 1 homolog beta (yeast) |
| 7272 | TTK | TTK protein kinase |
| 10615 | SPAG5 | sperm associated antigen 5 |
| 9055 | PRC1 | protein regulator of cytokinesis 1 |
| 10403 | NDC80 | NDC80 homolog, kinetochore complex component (S. cerevisiae) |
| 11065 | UBE2C | ubiquitin-conjugating enzyme E2C |

  
  
**MSCs batch 1 repressed and**GO:0009309**: 9 genes, expected 1.84439, P=8.82936e-05, P adjusted = 1

|  |  |  |
| --- | --- | --- |
| 440 | ASNS | asparagine synthetase |
| 587 | BCAT2 | branched chain aminotransferase 2, mitochondrial |
| 29968 | PSAT1 | phosphoserine aminotransferase 1 |
| 875 | CBS | cystathionine-beta-synthase |
| 26227 | PHGDH | phosphoglycerate dehydrogenase |
| 65263 | PYCRL | pyrroline-5-carboxylate reductase-like |
| 435 | ASL | argininosuccinate lyase |
| 5351 | PLOD1 | procollagen-lysine 1, 2-oxoglutarate 5-dioxygenase 1 |
| 5831 | PYCR1 | pyrroline-5-carboxylate reductase 1 |

  
  
**MSCs batch 1 induced and**GO:0005125**: 16 genes, expected 4.7258, P=2.34948e-05, P adjusted = 1

|  |  |  |
| --- | --- | --- |
| 650 | BMP2 | bone morphogenetic protein 2 |
| 374 | AREG | amphiregulin |
| 2920 | CXCL2 | chemokine (C-X-C motif) ligand 2 |
| 6696 | SPP1 | secreted phosphoprotein 1 |
| 2919 | CXCL1 | chemokine (C-X-C motif) ligand 1 (melanoma growth stimulating activity, alpha) |
| 7292 | TNFSF4 | tumor necrosis factor (ligand) superfamily, member 4 |
| 3576 | IL8 | interleukin 8 |
| 6372 | CXCL6 | chemokine (C-X-C motif) ligand 6 (granulocyte chemotactic protein 2) |
| 11009 | IL24 | interleukin 24 |
| 10135 | NAMPT | nicotinamide phosphoribosyltransferase |
| 2921 | CXCL3 | chemokine (C-X-C motif) ligand 3 |
| 85480 | TSLP | thymic stromal lymphopoietin |
| 7857 | SCG2 | secretogranin II (chromogranin C) |
| 4254 | KITLG | KIT ligand |
| 6374 | CXCL5 | chemokine (C-X-C motif) ligand 5 |
| 5617 | PRL | prolactin |

  
  
**MSCs batch 1 induced and**GO:0045595**: 14 genes, expected 4.22491, P=9.48351e-05, P adjusted = 1

|  |  |  |
| --- | --- | --- |
| 650 | BMP2 | bone morphogenetic protein 2 |
| 9935 | MAFB | v-maf musculoaponeurotic fibrosarcoma oncogene homolog B (avian) |
| 360 | AQP3 | aquaporin 3 (Gill blood group) |
| 2261 | FGFR3 | fibroblast growth factor receptor 3 |
| 4853 | NOTCH2 | Notch homolog 2 (Drosophila) |
| 2138 | EYA1 | eyes absent homolog 1 (Drosophila) |
| 84525 | HOPX | HOP homeobox |
| 3280 | HES1 | hairy and enhancer of split 1, (Drosophila) |
| 658 | BMPR1B | bone morphogenetic protein receptor, type IB |
| 3981 | LIG4 | ligase IV, DNA, ATP-dependent |
| 3398 | ID2 | inhibitor of DNA binding 2, dominant negative helix-loop-helix protein |
| 604 | BCL6 | B-cell CLL/lymphoma 6 |
| 7098 | TLR3 | toll-like receptor 3 |
| 659 | BMPR2 | bone morphogenetic protein receptor, type II (serine/threonine kinase) |

  
  
**MSCs batch 2 repressed and**GO:0007067**: 52 genes, expected 2.56137, P=1.51795e-53, P adjusted = 1.76708e-48

|  |  |  |
| --- | --- | --- |
| 9735 | KNTC1 | kinetochore associated 1 |
| 57405 | SPC25 | SPC25, NDC80 kinetochore complex component, homolog (S. cerevisiae) |
| 983 | CDC2 | cell division cycle 2, G1 to S and G2 to M |
| 9212 | AURKB | aurora kinase B |
| 9787 | DLGAP5 | discs, large (Drosophila) homolog-associated protein 5 |
| 26271 | FBXO5 | F-box protein 5 |
| 3832 | KIF11 | kinesin family member 11 |
| 891 | CCNB1 | cyclin B1 |
| 995 | CDC25C | cell division cycle 25 homolog C (S. pombe) |
| 3070 | HELLS | helicase, lymphoid-specific |
| 4085 | MAD2L1 | MAD2 mitotic arrest deficient-like 1 (yeast) |
| 332 | BIRC5 | baculoviral IAP repeat-containing 5 |
| 11004 | KIF2C | kinesin family member 2C |
| 701 | BUB1B | BUB1 budding uninhibited by benzimidazoles 1 homolog beta (yeast) |
| 113130 | CDCA5 | cell division cycle associated 5 |
| 259266 | ASPM | asp (abnormal spindle) homolog, microcephaly associated (Drosophila) |
| 1062 | CENPE | centromere protein E, 312kDa |
| 4751 | NEK2 | NIMA (never in mitosis gene a)-related kinase 2 |
| 22974 | TPX2 | TPX2, microtubule-associated, homolog (Xenopus laevis) |
| 51203 | NUSAP1 | nucleolar and spindle associated protein 1 |
| 1063 | CENPF | centromere protein F, 350/400ka (mitosin) |
| 10403 | NDC80 | NDC80 homolog, kinetochore complex component (S. cerevisiae) |
| 220134 | C18orf24 | chromosome 18 open reading frame 24 |
| 23397 | NCAPH | non-SMC condensin I complex, subunit H |
| 9133 | CCNB2 | cyclin B2 |
| 9232 | PTTG1 | pituitary tumor-transforming 1 |
| 54892 | NCAPG2 | non-SMC condensin II complex, subunit G2 |
| 157313 | CDCA2 | cell division cycle associated 2 |
| 11130 | ZWINT | ZW10 interactor |
| 64151 | NCAPG | non-SMC condensin I complex, subunit G |
| 54443 | ANLN | anillin, actin binding protein |
| 6790 | AURKA | aurora kinase A |
| 990 | CDC6 | cell division cycle 6 homolog (S. cerevisiae) |
| 83461 | CDCA3 | cell division cycle associated 3 |
| 890 | CCNA2 | cyclin A2 |
| 55872 | PBK | PDZ binding kinase |
| 10615 | SPAG5 | sperm associated antigen 5 |
| 9493 | KIF23 | kinesin family member 23 |
| 55165 | CEP55 | centrosomal protein 55kDa |
| 56992 | KIF15 | kinesin family member 15 |
| 83540 | NUF2 | NUF2, NDC80 kinetochore complex component, homolog (S. cerevisiae) |
| 11113 | CIT | citron (rho-interacting, serine/threonine kinase 21) |
| 10051 | SMC4 | structural maintenance of chromosomes 4 |
| 991 | CDC20 | cell division cycle 20 homolog (S. cerevisiae) |
| 11065 | UBE2C | ubiquitin-conjugating enzyme E2C |
| 3479 | IGF1 | insulin-like growth factor 1 (somatomedin C) |
| 993 | CDC25A | cell division cycle 25 homolog A (S. pombe) |
| 699 | BUB1 | BUB1 budding uninhibited by benzimidazoles 1 homolog (yeast) |
| 55143 | CDCA8 | cell division cycle associated 8 |
| 8914 | TIMELESS | timeless homolog (Drosophila) |
| 54821 | ERCC6L | excision repair cross-complementing rodent repair deficiency, complementation group 6-like |
| 9585 | KIF20B | kinesin family member 20B |

  
  
**MSCs batch 2 repressed and**GO:0000087**: 52 genes, expected 2.58612, P=2.64222e-53, P adjusted = 3.07586e-48

|  |  |  |
| --- | --- | --- |
| 9735 | KNTC1 | kinetochore associated 1 |
| 57405 | SPC25 | SPC25, NDC80 kinetochore complex component, homolog (S. cerevisiae) |
| 983 | CDC2 | cell division cycle 2, G1 to S and G2 to M |
| 9212 | AURKB | aurora kinase B |
| 9787 | DLGAP5 | discs, large (Drosophila) homolog-associated protein 5 |
| 26271 | FBXO5 | F-box protein 5 |
| 3832 | KIF11 | kinesin family member 11 |
| 891 | CCNB1 | cyclin B1 |
| 995 | CDC25C | cell division cycle 25 homolog C (S. pombe) |
| 3070 | HELLS | helicase, lymphoid-specific |
| 4085 | MAD2L1 | MAD2 mitotic arrest deficient-like 1 (yeast) |
| 332 | BIRC5 | baculoviral IAP repeat-containing 5 |
| 11004 | KIF2C | kinesin family member 2C |
| 701 | BUB1B | BUB1 budding uninhibited by benzimidazoles 1 homolog beta (yeast) |
| 113130 | CDCA5 | cell division cycle associated 5 |
| 259266 | ASPM | asp (abnormal spindle) homolog, microcephaly associated (Drosophila) |
| 1062 | CENPE | centromere protein E, 312kDa |
| 4751 | NEK2 | NIMA (never in mitosis gene a)-related kinase 2 |
| 22974 | TPX2 | TPX2, microtubule-associated, homolog (Xenopus laevis) |
| 51203 | NUSAP1 | nucleolar and spindle associated protein 1 |
| 1063 | CENPF | centromere protein F, 350/400ka (mitosin) |
| 10403 | NDC80 | NDC80 homolog, kinetochore complex component (S. cerevisiae) |
| 220134 | C18orf24 | chromosome 18 open reading frame 24 |
| 23397 | NCAPH | non-SMC condensin I complex, subunit H |
| 9133 | CCNB2 | cyclin B2 |
| 9232 | PTTG1 | pituitary tumor-transforming 1 |
| 54892 | NCAPG2 | non-SMC condensin II complex, subunit G2 |
| 157313 | CDCA2 | cell division cycle associated 2 |
| 11130 | ZWINT | ZW10 interactor |
| 64151 | NCAPG | non-SMC condensin I complex, subunit G |
| 54443 | ANLN | anillin, actin binding protein |
| 6790 | AURKA | aurora kinase A |
| 990 | CDC6 | cell division cycle 6 homolog (S. cerevisiae) |
| 83461 | CDCA3 | cell division cycle associated 3 |
| 890 | CCNA2 | cyclin A2 |
| 55872 | PBK | PDZ binding kinase |
| 10615 | SPAG5 | sperm associated antigen 5 |
| 9493 | KIF23 | kinesin family member 23 |
| 55165 | CEP55 | centrosomal protein 55kDa |
| 56992 | KIF15 | kinesin family member 15 |
| 83540 | NUF2 | NUF2, NDC80 kinetochore complex component, homolog (S. cerevisiae) |
| 11113 | CIT | citron (rho-interacting, serine/threonine kinase 21) |
| 10051 | SMC4 | structural maintenance of chromosomes 4 |
| 991 | CDC20 | cell division cycle 20 homolog (S. cerevisiae) |
| 11065 | UBE2C | ubiquitin-conjugating enzyme E2C |
| 3479 | IGF1 | insulin-like growth factor 1 (somatomedin C) |
| 993 | CDC25A | cell division cycle 25 homolog A (S. pombe) |
| 699 | BUB1 | BUB1 budding uninhibited by benzimidazoles 1 homolog (yeast) |
| 55143 | CDCA8 | cell division cycle associated 8 |
| 8914 | TIMELESS | timeless homolog (Drosophila) |
| 54821 | ERCC6L | excision repair cross-complementing rodent repair deficiency, complementation group 6-like |
| 9585 | KIF20B | kinesin family member 20B |

  
  
**MSCs batch 2 repressed and**GO:0000279**: 57 genes, expected 3.5884, P=3.32926e-52, P adjusted = 3.87566e-47

|  |  |  |
| --- | --- | --- |
| 9735 | KNTC1 | kinetochore associated 1 |
| 57405 | SPC25 | SPC25, NDC80 kinetochore complex component, homolog (S. cerevisiae) |
| 983 | CDC2 | cell division cycle 2, G1 to S and G2 to M |
| 9212 | AURKB | aurora kinase B |
| 9787 | DLGAP5 | discs, large (Drosophila) homolog-associated protein 5 |
| 26271 | FBXO5 | F-box protein 5 |
| 3832 | KIF11 | kinesin family member 11 |
| 891 | CCNB1 | cyclin B1 |
| 995 | CDC25C | cell division cycle 25 homolog C (S. pombe) |
| 3070 | HELLS | helicase, lymphoid-specific |
| 84057 | MND1 | meiotic nuclear divisions 1 homolog (S. cerevisiae) |
| 4085 | MAD2L1 | MAD2 mitotic arrest deficient-like 1 (yeast) |
| 332 | BIRC5 | baculoviral IAP repeat-containing 5 |
| 11004 | KIF2C | kinesin family member 2C |
| 701 | BUB1B | BUB1 budding uninhibited by benzimidazoles 1 homolog beta (yeast) |
| 113130 | CDCA5 | cell division cycle associated 5 |
| 259266 | ASPM | asp (abnormal spindle) homolog, microcephaly associated (Drosophila) |
| 1062 | CENPE | centromere protein E, 312kDa |
| 4751 | NEK2 | NIMA (never in mitosis gene a)-related kinase 2 |
| 22974 | TPX2 | TPX2, microtubule-associated, homolog (Xenopus laevis) |
| 3925 | STMN1 | stathmin 1/oncoprotein 18 |
| 51203 | NUSAP1 | nucleolar and spindle associated protein 1 |
| 1063 | CENPF | centromere protein F, 350/400ka (mitosin) |
| 10403 | NDC80 | NDC80 homolog, kinetochore complex component (S. cerevisiae) |
| 220134 | C18orf24 | chromosome 18 open reading frame 24 |
| 23397 | NCAPH | non-SMC condensin I complex, subunit H |
| 9133 | CCNB2 | cyclin B2 |
| 9232 | PTTG1 | pituitary tumor-transforming 1 |
| 54892 | NCAPG2 | non-SMC condensin II complex, subunit G2 |
| 7272 | TTK | TTK protein kinase |
| 157313 | CDCA2 | cell division cycle associated 2 |
| 11130 | ZWINT | ZW10 interactor |
| 64151 | NCAPG | non-SMC condensin I complex, subunit G |
| 54443 | ANLN | anillin, actin binding protein |
| 6790 | AURKA | aurora kinase A |
| 990 | CDC6 | cell division cycle 6 homolog (S. cerevisiae) |
| 83461 | CDCA3 | cell division cycle associated 3 |
| 890 | CCNA2 | cyclin A2 |
| 55872 | PBK | PDZ binding kinase |
| 10615 | SPAG5 | sperm associated antigen 5 |
| 9493 | KIF23 | kinesin family member 23 |
| 55165 | CEP55 | centrosomal protein 55kDa |
| 56992 | KIF15 | kinesin family member 15 |
| 83540 | NUF2 | NUF2, NDC80 kinetochore complex component, homolog (S. cerevisiae) |
| 11113 | CIT | citron (rho-interacting, serine/threonine kinase 21) |
| 10051 | SMC4 | structural maintenance of chromosomes 4 |
| 991 | CDC20 | cell division cycle 20 homolog (S. cerevisiae) |
| 11065 | UBE2C | ubiquitin-conjugating enzyme E2C |
| 3479 | IGF1 | insulin-like growth factor 1 (somatomedin C) |
| 993 | CDC25A | cell division cycle 25 homolog A (S. pombe) |
| 9055 | PRC1 | protein regulator of cytokinesis 1 |
| 699 | BUB1 | BUB1 budding uninhibited by benzimidazoles 1 homolog (yeast) |
| 55143 | CDCA8 | cell division cycle associated 8 |
| 8914 | TIMELESS | timeless homolog (Drosophila) |
| 54821 | ERCC6L | excision repair cross-complementing rodent repair deficiency, complementation group 6-like |
| 9156 | EXO1 | exonuclease 1 |
| 9585 | KIF20B | kinesin family member 20B |

  
  
**MSCs batch 2 repressed and**GO:0022403**: 59 genes, expected 4.46694, P=3.91581e-49, P adjusted = 4.55848e-44

|  |  |  |
| --- | --- | --- |
| 9735 | KNTC1 | kinetochore associated 1 |
| 57405 | SPC25 | SPC25, NDC80 kinetochore complex component, homolog (S. cerevisiae) |
| 983 | CDC2 | cell division cycle 2, G1 to S and G2 to M |
| 9212 | AURKB | aurora kinase B |
| 9787 | DLGAP5 | discs, large (Drosophila) homolog-associated protein 5 |
| 26271 | FBXO5 | F-box protein 5 |
| 3832 | KIF11 | kinesin family member 11 |
| 891 | CCNB1 | cyclin B1 |
| 995 | CDC25C | cell division cycle 25 homolog C (S. pombe) |
| 3070 | HELLS | helicase, lymphoid-specific |
| 84057 | MND1 | meiotic nuclear divisions 1 homolog (S. cerevisiae) |
| 4085 | MAD2L1 | MAD2 mitotic arrest deficient-like 1 (yeast) |
| 332 | BIRC5 | baculoviral IAP repeat-containing 5 |
| 11004 | KIF2C | kinesin family member 2C |
| 701 | BUB1B | BUB1 budding uninhibited by benzimidazoles 1 homolog beta (yeast) |
| 113130 | CDCA5 | cell division cycle associated 5 |
| 259266 | ASPM | asp (abnormal spindle) homolog, microcephaly associated (Drosophila) |
| 1062 | CENPE | centromere protein E, 312kDa |
| 4751 | NEK2 | NIMA (never in mitosis gene a)-related kinase 2 |
| 22974 | TPX2 | TPX2, microtubule-associated, homolog (Xenopus laevis) |
| 3925 | STMN1 | stathmin 1/oncoprotein 18 |
| 51203 | NUSAP1 | nucleolar and spindle associated protein 1 |
| 1063 | CENPF | centromere protein F, 350/400ka (mitosin) |
| 10403 | NDC80 | NDC80 homolog, kinetochore complex component (S. cerevisiae) |
| 220134 | C18orf24 | chromosome 18 open reading frame 24 |
| 23397 | NCAPH | non-SMC condensin I complex, subunit H |
| 9133 | CCNB2 | cyclin B2 |
| 9232 | PTTG1 | pituitary tumor-transforming 1 |
| 54892 | NCAPG2 | non-SMC condensin II complex, subunit G2 |
| 7272 | TTK | TTK protein kinase |
| 157313 | CDCA2 | cell division cycle associated 2 |
| 11130 | ZWINT | ZW10 interactor |
| 64151 | NCAPG | non-SMC condensin I complex, subunit G |
| 54443 | ANLN | anillin, actin binding protein |
| 6790 | AURKA | aurora kinase A |
| 51512 | GTSE1 | G-2 and S-phase expressed 1 |
| 990 | CDC6 | cell division cycle 6 homolog (S. cerevisiae) |
| 83461 | CDCA3 | cell division cycle associated 3 |
| 890 | CCNA2 | cyclin A2 |
| 55872 | PBK | PDZ binding kinase |
| 10615 | SPAG5 | sperm associated antigen 5 |
| 9493 | KIF23 | kinesin family member 23 |
| 55165 | CEP55 | centrosomal protein 55kDa |
| 56992 | KIF15 | kinesin family member 15 |
| 83540 | NUF2 | NUF2, NDC80 kinetochore complex component, homolog (S. cerevisiae) |
| 11113 | CIT | citron (rho-interacting, serine/threonine kinase 21) |
| 10051 | SMC4 | structural maintenance of chromosomes 4 |
| 1033 | CDKN3 | cyclin-dependent kinase inhibitor 3 |
| 991 | CDC20 | cell division cycle 20 homolog (S. cerevisiae) |
| 11065 | UBE2C | ubiquitin-conjugating enzyme E2C |
| 3479 | IGF1 | insulin-like growth factor 1 (somatomedin C) |
| 993 | CDC25A | cell division cycle 25 homolog A (S. pombe) |
| 9055 | PRC1 | protein regulator of cytokinesis 1 |
| 699 | BUB1 | BUB1 budding uninhibited by benzimidazoles 1 homolog (yeast) |
| 55143 | CDCA8 | cell division cycle associated 8 |
| 8914 | TIMELESS | timeless homolog (Drosophila) |
| 54821 | ERCC6L | excision repair cross-complementing rodent repair deficiency, complementation group 6-like |
| 9156 | EXO1 | exonuclease 1 |
| 9585 | KIF20B | kinesin family member 20B |

  
  
**MSCs batch 2 repressed and**GO:0000278**: 57 genes, expected 4.54118, P=3.98118e-46, P adjusted = 4.63457e-41

|  |  |  |
| --- | --- | --- |
| 9735 | KNTC1 | kinetochore associated 1 |
| 57405 | SPC25 | SPC25, NDC80 kinetochore complex component, homolog (S. cerevisiae) |
| 983 | CDC2 | cell division cycle 2, G1 to S and G2 to M |
| 9212 | AURKB | aurora kinase B |
| 9787 | DLGAP5 | discs, large (Drosophila) homolog-associated protein 5 |
| 26271 | FBXO5 | F-box protein 5 |
| 3832 | KIF11 | kinesin family member 11 |
| 891 | CCNB1 | cyclin B1 |
| 995 | CDC25C | cell division cycle 25 homolog C (S. pombe) |
| 3070 | HELLS | helicase, lymphoid-specific |
| 4085 | MAD2L1 | MAD2 mitotic arrest deficient-like 1 (yeast) |
| 332 | BIRC5 | baculoviral IAP repeat-containing 5 |
| 11004 | KIF2C | kinesin family member 2C |
| 701 | BUB1B | BUB1 budding uninhibited by benzimidazoles 1 homolog beta (yeast) |
| 113130 | CDCA5 | cell division cycle associated 5 |
| 259266 | ASPM | asp (abnormal spindle) homolog, microcephaly associated (Drosophila) |
| 1062 | CENPE | centromere protein E, 312kDa |
| 4751 | NEK2 | NIMA (never in mitosis gene a)-related kinase 2 |
| 22974 | TPX2 | TPX2, microtubule-associated, homolog (Xenopus laevis) |
| 3925 | STMN1 | stathmin 1/oncoprotein 18 |
| 51203 | NUSAP1 | nucleolar and spindle associated protein 1 |
| 1063 | CENPF | centromere protein F, 350/400ka (mitosin) |
| 10403 | NDC80 | NDC80 homolog, kinetochore complex component (S. cerevisiae) |
| 220134 | C18orf24 | chromosome 18 open reading frame 24 |
| 23397 | NCAPH | non-SMC condensin I complex, subunit H |
| 9133 | CCNB2 | cyclin B2 |
| 9232 | PTTG1 | pituitary tumor-transforming 1 |
| 54892 | NCAPG2 | non-SMC condensin II complex, subunit G2 |
| 7272 | TTK | TTK protein kinase |
| 157313 | CDCA2 | cell division cycle associated 2 |
| 11130 | ZWINT | ZW10 interactor |
| 64151 | NCAPG | non-SMC condensin I complex, subunit G |
| 54443 | ANLN | anillin, actin binding protein |
| 6790 | AURKA | aurora kinase A |
| 51512 | GTSE1 | G-2 and S-phase expressed 1 |
| 990 | CDC6 | cell division cycle 6 homolog (S. cerevisiae) |
| 83461 | CDCA3 | cell division cycle associated 3 |
| 890 | CCNA2 | cyclin A2 |
| 55872 | PBK | PDZ binding kinase |
| 10615 | SPAG5 | sperm associated antigen 5 |
| 9493 | KIF23 | kinesin family member 23 |
| 55165 | CEP55 | centrosomal protein 55kDa |
| 56992 | KIF15 | kinesin family member 15 |
| 83540 | NUF2 | NUF2, NDC80 kinetochore complex component, homolog (S. cerevisiae) |
| 11113 | CIT | citron (rho-interacting, serine/threonine kinase 21) |
| 10051 | SMC4 | structural maintenance of chromosomes 4 |
| 1033 | CDKN3 | cyclin-dependent kinase inhibitor 3 |
| 991 | CDC20 | cell division cycle 20 homolog (S. cerevisiae) |
| 11065 | UBE2C | ubiquitin-conjugating enzyme E2C |
| 3479 | IGF1 | insulin-like growth factor 1 (somatomedin C) |
| 993 | CDC25A | cell division cycle 25 homolog A (S. pombe) |
| 9055 | PRC1 | protein regulator of cytokinesis 1 |
| 699 | BUB1 | BUB1 budding uninhibited by benzimidazoles 1 homolog (yeast) |
| 55143 | CDCA8 | cell division cycle associated 8 |
| 8914 | TIMELESS | timeless homolog (Drosophila) |
| 54821 | ERCC6L | excision repair cross-complementing rodent repair deficiency, complementation group 6-like |
| 9585 | KIF20B | kinesin family member 20B |

  
  
**MSCs batch 2 repressed and**GO:0051301**: 49 genes, expected 2.90784, P=4.26042e-46, P adjusted = 4.95964e-41

|  |  |  |
| --- | --- | --- |
| 9735 | KNTC1 | kinetochore associated 1 |
| 57405 | SPC25 | SPC25, NDC80 kinetochore complex component, homolog (S. cerevisiae) |
| 983 | CDC2 | cell division cycle 2, G1 to S and G2 to M |
| 9212 | AURKB | aurora kinase B |
| 26271 | FBXO5 | F-box protein 5 |
| 3832 | KIF11 | kinesin family member 11 |
| 891 | CCNB1 | cyclin B1 |
| 995 | CDC25C | cell division cycle 25 homolog C (S. pombe) |
| 3070 | HELLS | helicase, lymphoid-specific |
| 4085 | MAD2L1 | MAD2 mitotic arrest deficient-like 1 (yeast) |
| 332 | BIRC5 | baculoviral IAP repeat-containing 5 |
| 701 | BUB1B | BUB1 budding uninhibited by benzimidazoles 1 homolog beta (yeast) |
| 113130 | CDCA5 | cell division cycle associated 5 |
| 29127 | RACGAP1 | Rac GTPase activating protein 1 |
| 259266 | ASPM | asp (abnormal spindle) homolog, microcephaly associated (Drosophila) |
| 1062 | CENPE | centromere protein E, 312kDa |
| 4751 | NEK2 | NIMA (never in mitosis gene a)-related kinase 2 |
| 51203 | NUSAP1 | nucleolar and spindle associated protein 1 |
| 1063 | CENPF | centromere protein F, 350/400ka (mitosin) |
| 10403 | NDC80 | NDC80 homolog, kinetochore complex component (S. cerevisiae) |
| 220134 | C18orf24 | chromosome 18 open reading frame 24 |
| 23397 | NCAPH | non-SMC condensin I complex, subunit H |
| 9133 | CCNB2 | cyclin B2 |
| 9232 | PTTG1 | pituitary tumor-transforming 1 |
| 54892 | NCAPG2 | non-SMC condensin II complex, subunit G2 |
| 157313 | CDCA2 | cell division cycle associated 2 |
| 11130 | ZWINT | ZW10 interactor |
| 64151 | NCAPG | non-SMC condensin I complex, subunit G |
| 54443 | ANLN | anillin, actin binding protein |
| 990 | CDC6 | cell division cycle 6 homolog (S. cerevisiae) |
| 83461 | CDCA3 | cell division cycle associated 3 |
| 890 | CCNA2 | cyclin A2 |
| 10615 | SPAG5 | sperm associated antigen 5 |
| 9493 | KIF23 | kinesin family member 23 |
| 55165 | CEP55 | centrosomal protein 55kDa |
| 83540 | NUF2 | NUF2, NDC80 kinetochore complex component, homolog (S. cerevisiae) |
| 11113 | CIT | citron (rho-interacting, serine/threonine kinase 21) |
| 10051 | SMC4 | structural maintenance of chromosomes 4 |
| 991 | CDC20 | cell division cycle 20 homolog (S. cerevisiae) |
| 11065 | UBE2C | ubiquitin-conjugating enzyme E2C |
| 993 | CDC25A | cell division cycle 25 homolog A (S. pombe) |
| 151246 | SGOL2 | shugoshin-like 2 (S. pombe) |
| 9055 | PRC1 | protein regulator of cytokinesis 1 |
| 699 | BUB1 | BUB1 budding uninhibited by benzimidazoles 1 homolog (yeast) |
| 55143 | CDCA8 | cell division cycle associated 8 |
| 9134 | CCNE2 | cyclin E2 |
| 8914 | TIMELESS | timeless homolog (Drosophila) |
| 54821 | ERCC6L | excision repair cross-complementing rodent repair deficiency, complementation group 6-like |
| 9585 | KIF20B | kinesin family member 20B |

  
  
**MSCs batch 2 repressed and**GO:0022402**: 62 genes, expected 6.02603, P=6.7434e-45, P adjusted = 7.85013e-40

|  |  |  |
| --- | --- | --- |
| 9735 | KNTC1 | kinetochore associated 1 |
| 57405 | SPC25 | SPC25, NDC80 kinetochore complex component, homolog (S. cerevisiae) |
| 983 | CDC2 | cell division cycle 2, G1 to S and G2 to M |
| 9212 | AURKB | aurora kinase B |
| 9787 | DLGAP5 | discs, large (Drosophila) homolog-associated protein 5 |
| 26271 | FBXO5 | F-box protein 5 |
| 3832 | KIF11 | kinesin family member 11 |
| 891 | CCNB1 | cyclin B1 |
| 995 | CDC25C | cell division cycle 25 homolog C (S. pombe) |
| 3070 | HELLS | helicase, lymphoid-specific |
| 84057 | MND1 | meiotic nuclear divisions 1 homolog (S. cerevisiae) |
| 4085 | MAD2L1 | MAD2 mitotic arrest deficient-like 1 (yeast) |
| 332 | BIRC5 | baculoviral IAP repeat-containing 5 |
| 11004 | KIF2C | kinesin family member 2C |
| 701 | BUB1B | BUB1 budding uninhibited by benzimidazoles 1 homolog beta (yeast) |
| 113130 | CDCA5 | cell division cycle associated 5 |
| 29127 | RACGAP1 | Rac GTPase activating protein 1 |
| 259266 | ASPM | asp (abnormal spindle) homolog, microcephaly associated (Drosophila) |
| 1062 | CENPE | centromere protein E, 312kDa |
| 4751 | NEK2 | NIMA (never in mitosis gene a)-related kinase 2 |
| 22974 | TPX2 | TPX2, microtubule-associated, homolog (Xenopus laevis) |
| 3925 | STMN1 | stathmin 1/oncoprotein 18 |
| 51203 | NUSAP1 | nucleolar and spindle associated protein 1 |
| 1063 | CENPF | centromere protein F, 350/400ka (mitosin) |
| 10403 | NDC80 | NDC80 homolog, kinetochore complex component (S. cerevisiae) |
| 220134 | C18orf24 | chromosome 18 open reading frame 24 |
| 23397 | NCAPH | non-SMC condensin I complex, subunit H |
| 9133 | CCNB2 | cyclin B2 |
| 9232 | PTTG1 | pituitary tumor-transforming 1 |
| 54892 | NCAPG2 | non-SMC condensin II complex, subunit G2 |
| 7272 | TTK | TTK protein kinase |
| 157313 | CDCA2 | cell division cycle associated 2 |
| 11130 | ZWINT | ZW10 interactor |
| 283431 | GAS2L3 | growth arrest-specific 2 like 3 |
| 64151 | NCAPG | non-SMC condensin I complex, subunit G |
| 54443 | ANLN | anillin, actin binding protein |
| 6790 | AURKA | aurora kinase A |
| 51512 | GTSE1 | G-2 and S-phase expressed 1 |
| 990 | CDC6 | cell division cycle 6 homolog (S. cerevisiae) |
| 83461 | CDCA3 | cell division cycle associated 3 |
| 890 | CCNA2 | cyclin A2 |
| 55872 | PBK | PDZ binding kinase |
| 10615 | SPAG5 | sperm associated antigen 5 |
| 9493 | KIF23 | kinesin family member 23 |
| 55165 | CEP55 | centrosomal protein 55kDa |
| 56992 | KIF15 | kinesin family member 15 |
| 220042 | C11orf82 | chromosome 11 open reading frame 82 |
| 83540 | NUF2 | NUF2, NDC80 kinetochore complex component, homolog (S. cerevisiae) |
| 11113 | CIT | citron (rho-interacting, serine/threonine kinase 21) |
| 10051 | SMC4 | structural maintenance of chromosomes 4 |
| 1033 | CDKN3 | cyclin-dependent kinase inhibitor 3 |
| 991 | CDC20 | cell division cycle 20 homolog (S. cerevisiae) |
| 11065 | UBE2C | ubiquitin-conjugating enzyme E2C |
| 3479 | IGF1 | insulin-like growth factor 1 (somatomedin C) |
| 993 | CDC25A | cell division cycle 25 homolog A (S. pombe) |
| 9055 | PRC1 | protein regulator of cytokinesis 1 |
| 699 | BUB1 | BUB1 budding uninhibited by benzimidazoles 1 homolog (yeast) |
| 55143 | CDCA8 | cell division cycle associated 8 |
| 8914 | TIMELESS | timeless homolog (Drosophila) |
| 54821 | ERCC6L | excision repair cross-complementing rodent repair deficiency, complementation group 6-like |
| 9156 | EXO1 | exonuclease 1 |
| 9585 | KIF20B | kinesin family member 20B |

  
  
**MSCs batch 2 repressed and**GO:0007049**: 74 genes, expected 10.1341, P=2.5188e-43, P adjusted = 2.93219e-38

|  |  |  |
| --- | --- | --- |
| 9735 | KNTC1 | kinetochore associated 1 |
| 57405 | SPC25 | SPC25, NDC80 kinetochore complex component, homolog (S. cerevisiae) |
| 983 | CDC2 | cell division cycle 2, G1 to S and G2 to M |
| 9212 | AURKB | aurora kinase B |
| 2177 | FANCD2 | Fanconi anemia, complementation group D2 |
| 9787 | DLGAP5 | discs, large (Drosophila) homolog-associated protein 5 |
| 26271 | FBXO5 | F-box protein 5 |
| 3832 | KIF11 | kinesin family member 11 |
| 891 | CCNB1 | cyclin B1 |
| 995 | CDC25C | cell division cycle 25 homolog C (S. pombe) |
| 3070 | HELLS | helicase, lymphoid-specific |
| 84057 | MND1 | meiotic nuclear divisions 1 homolog (S. cerevisiae) |
| 4085 | MAD2L1 | MAD2 mitotic arrest deficient-like 1 (yeast) |
| 55215 | FANCI | Fanconi anemia, complementation group I |
| 332 | BIRC5 | baculoviral IAP repeat-containing 5 |
| 11004 | KIF2C | kinesin family member 2C |
| 701 | BUB1B | BUB1 budding uninhibited by benzimidazoles 1 homolog beta (yeast) |
| 113130 | CDCA5 | cell division cycle associated 5 |
| 29127 | RACGAP1 | Rac GTPase activating protein 1 |
| 259266 | ASPM | asp (abnormal spindle) homolog, microcephaly associated (Drosophila) |
| 1062 | CENPE | centromere protein E, 312kDa |
| 4751 | NEK2 | NIMA (never in mitosis gene a)-related kinase 2 |
| 22974 | TPX2 | TPX2, microtubule-associated, homolog (Xenopus laevis) |
| 3925 | STMN1 | stathmin 1/oncoprotein 18 |
| 51203 | NUSAP1 | nucleolar and spindle associated protein 1 |
| 4288 | MKI67 | antigen identified by monoclonal antibody Ki-67 |
| 1063 | CENPF | centromere protein F, 350/400ka (mitosin) |
| 4176 | MCM7 | minichromosome maintenance complex component 7 |
| 10403 | NDC80 | NDC80 homolog, kinetochore complex component (S. cerevisiae) |
| 4171 | MCM2 | minichromosome maintenance complex component 2 |
| 220134 | C18orf24 | chromosome 18 open reading frame 24 |
| 23397 | NCAPH | non-SMC condensin I complex, subunit H |
| 9133 | CCNB2 | cyclin B2 |
| 9232 | PTTG1 | pituitary tumor-transforming 1 |
| 8318 | CDC45L | CDC45 cell division cycle 45-like (S. cerevisiae) |
| 54892 | NCAPG2 | non-SMC condensin II complex, subunit G2 |
| 7272 | TTK | TTK protein kinase |
| 157313 | CDCA2 | cell division cycle associated 2 |
| 11130 | ZWINT | ZW10 interactor |
| 283431 | GAS2L3 | growth arrest-specific 2 like 3 |
| 64151 | NCAPG | non-SMC condensin I complex, subunit G |
| 54443 | ANLN | anillin, actin binding protein |
| 6790 | AURKA | aurora kinase A |
| 51512 | GTSE1 | G-2 and S-phase expressed 1 |
| 990 | CDC6 | cell division cycle 6 homolog (S. cerevisiae) |
| 83461 | CDCA3 | cell division cycle associated 3 |
| 10460 | TACC3 | transforming, acidic coiled-coil containing protein 3 |
| 890 | CCNA2 | cyclin A2 |
| 55872 | PBK | PDZ binding kinase |
| 10615 | SPAG5 | sperm associated antigen 5 |
| 9493 | KIF23 | kinesin family member 23 |
| 55165 | CEP55 | centrosomal protein 55kDa |
| 56992 | KIF15 | kinesin family member 15 |
| 220042 | C11orf82 | chromosome 11 open reading frame 82 |
| 83540 | NUF2 | NUF2, NDC80 kinetochore complex component, homolog (S. cerevisiae) |
| 11113 | CIT | citron (rho-interacting, serine/threonine kinase 21) |
| 10051 | SMC4 | structural maintenance of chromosomes 4 |
| 1033 | CDKN3 | cyclin-dependent kinase inhibitor 3 |
| 991 | CDC20 | cell division cycle 20 homolog (S. cerevisiae) |
| 11065 | UBE2C | ubiquitin-conjugating enzyme E2C |
| 3479 | IGF1 | insulin-like growth factor 1 (somatomedin C) |
| 5933 | RBL1 | retinoblastoma-like 1 (p107) |
| 29128 | UHRF1 | ubiquitin-like with PHD and ring finger domains 1 |
| 993 | CDC25A | cell division cycle 25 homolog A (S. pombe) |
| 79733 | E2F8 | E2F transcription factor 8 |
| 151246 | SGOL2 | shugoshin-like 2 (S. pombe) |
| 9055 | PRC1 | protein regulator of cytokinesis 1 |
| 699 | BUB1 | BUB1 budding uninhibited by benzimidazoles 1 homolog (yeast) |
| 55143 | CDCA8 | cell division cycle associated 8 |
| 9134 | CCNE2 | cyclin E2 |
| 8914 | TIMELESS | timeless homolog (Drosophila) |
| 54821 | ERCC6L | excision repair cross-complementing rodent repair deficiency, complementation group 6-like |
| 9156 | EXO1 | exonuclease 1 |
| 9585 | KIF20B | kinesin family member 20B |

  
  
**MSCs batch 2 repressed and**GO:0005694**: 43 genes, expected 4.44219, P=7.71321e-30, P adjusted = 8.9791e-25

|  |  |  |
| --- | --- | --- |
| 9735 | KNTC1 | kinetochore associated 1 |
| 57405 | SPC25 | SPC25, NDC80 kinetochore complex component, homolog (S. cerevisiae) |
| 7153 | TOP2A | topoisomerase (DNA) II alpha 170kDa |
| 9212 | AURKB | aurora kinase B |
| 2177 | FANCD2 | Fanconi anemia, complementation group D2 |
| 2491 | CENPI | centromere protein I |
| 3070 | HELLS | helicase, lymphoid-specific |
| 4085 | MAD2L1 | MAD2 mitotic arrest deficient-like 1 (yeast) |
| 56852 | RAD18 | RAD18 homolog (S. cerevisiae) |
| 55215 | FANCI | Fanconi anemia, complementation group I |
| 332 | BIRC5 | baculoviral IAP repeat-containing 5 |
| 11004 | KIF2C | kinesin family member 2C |
| 701 | BUB1B | BUB1 budding uninhibited by benzimidazoles 1 homolog beta (yeast) |
| 113130 | CDCA5 | cell division cycle associated 5 |
| 79682 | MLF1IP | MLF1 interacting protein |
| 1062 | CENPE | centromere protein E, 312kDa |
| 5983 | RFC3 | replication factor C (activator 1) 3, 38kDa |
| 79019 | CENPM | centromere protein M |
| 10714 | POLD3 | polymerase (DNA-directed), delta 3, accessory subunit |
| 1063 | CENPF | centromere protein F, 350/400ka (mitosin) |
| 55839 | CENPN | centromere protein N |
| 4176 | MCM7 | minichromosome maintenance complex component 7 |
| 7112 | TMPO | thymopoietin |
| 23649 | POLA2 | polymerase (DNA directed), alpha 2 (70kD subunit) |
| 1058 | CENPA | centromere protein A |
| 10403 | NDC80 | NDC80 homolog, kinetochore complex component (S. cerevisiae) |
| 4171 | MCM2 | minichromosome maintenance complex component 2 |
| 220134 | C18orf24 | chromosome 18 open reading frame 24 |
| 11130 | ZWINT | ZW10 interactor |
| 2956 | MSH6 | mutS homolog 6 (E. coli) |
| 10615 | SPAG5 | sperm associated antigen 5 |
| 83540 | NUF2 | NUF2, NDC80 kinetochore complex component, homolog (S. cerevisiae) |
| 10051 | SMC4 | structural maintenance of chromosomes 4 |
| 5557 | PRIM1 | primase, DNA, polypeptide 1 (49kDa) |
| 64946 | CENPH | centromere protein H |
| 151246 | SGOL2 | shugoshin-like 2 (S. pombe) |
| 11339 | OIP5 | Opa interacting protein 5 |
| 699 | BUB1 | BUB1 budding uninhibited by benzimidazoles 1 homolog (yeast) |
| 55143 | CDCA8 | cell division cycle associated 8 |
| 8914 | TIMELESS | timeless homolog (Drosophila) |
| 54821 | ERCC6L | excision repair cross-complementing rodent repair deficiency, complementation group 6-like |
| 5985 | RFC5 | replication factor C (activator 1) 5, 36.5kDa |
| 3148 | HMGB2 | high-mobility group box 2 |

  
  
**MSCs batch 2 repressed and**GO:0044427**: 40 genes, expected 3.69976, P=1.143e-29, P adjusted = 1.33059e-24

|  |  |  |
| --- | --- | --- |
| 9735 | KNTC1 | kinetochore associated 1 |
| 57405 | SPC25 | SPC25, NDC80 kinetochore complex component, homolog (S. cerevisiae) |
| 9212 | AURKB | aurora kinase B |
| 2491 | CENPI | centromere protein I |
| 3070 | HELLS | helicase, lymphoid-specific |
| 4085 | MAD2L1 | MAD2 mitotic arrest deficient-like 1 (yeast) |
| 56852 | RAD18 | RAD18 homolog (S. cerevisiae) |
| 332 | BIRC5 | baculoviral IAP repeat-containing 5 |
| 11004 | KIF2C | kinesin family member 2C |
| 701 | BUB1B | BUB1 budding uninhibited by benzimidazoles 1 homolog beta (yeast) |
| 113130 | CDCA5 | cell division cycle associated 5 |
| 79682 | MLF1IP | MLF1 interacting protein |
| 1062 | CENPE | centromere protein E, 312kDa |
| 5983 | RFC3 | replication factor C (activator 1) 3, 38kDa |
| 79019 | CENPM | centromere protein M |
| 10714 | POLD3 | polymerase (DNA-directed), delta 3, accessory subunit |
| 1063 | CENPF | centromere protein F, 350/400ka (mitosin) |
| 55839 | CENPN | centromere protein N |
| 4176 | MCM7 | minichromosome maintenance complex component 7 |
| 7112 | TMPO | thymopoietin |
| 23649 | POLA2 | polymerase (DNA directed), alpha 2 (70kD subunit) |
| 1058 | CENPA | centromere protein A |
| 10403 | NDC80 | NDC80 homolog, kinetochore complex component (S. cerevisiae) |
| 4171 | MCM2 | minichromosome maintenance complex component 2 |
| 220134 | C18orf24 | chromosome 18 open reading frame 24 |
| 11130 | ZWINT | ZW10 interactor |
| 2956 | MSH6 | mutS homolog 6 (E. coli) |
| 10615 | SPAG5 | sperm associated antigen 5 |
| 83540 | NUF2 | NUF2, NDC80 kinetochore complex component, homolog (S. cerevisiae) |
| 10051 | SMC4 | structural maintenance of chromosomes 4 |
| 5557 | PRIM1 | primase, DNA, polypeptide 1 (49kDa) |
| 64946 | CENPH | centromere protein H |
| 151246 | SGOL2 | shugoshin-like 2 (S. pombe) |
| 11339 | OIP5 | Opa interacting protein 5 |
| 699 | BUB1 | BUB1 budding uninhibited by benzimidazoles 1 homolog (yeast) |
| 55143 | CDCA8 | cell division cycle associated 8 |
| 8914 | TIMELESS | timeless homolog (Drosophila) |
| 54821 | ERCC6L | excision repair cross-complementing rodent repair deficiency, complementation group 6-like |
| 5985 | RFC5 | replication factor C (activator 1) 5, 36.5kDa |
| 3148 | HMGB2 | high-mobility group box 2 |

  
  
**MSCs batch 2 repressed and**GO:0000775**: 25 genes, expected 1.05177, P=6.88323e-28, P adjusted = 8.01291e-23

|  |  |  |
| --- | --- | --- |
| 9735 | KNTC1 | kinetochore associated 1 |
| 57405 | SPC25 | SPC25, NDC80 kinetochore complex component, homolog (S. cerevisiae) |
| 9212 | AURKB | aurora kinase B |
| 2491 | CENPI | centromere protein I |
| 3070 | HELLS | helicase, lymphoid-specific |
| 4085 | MAD2L1 | MAD2 mitotic arrest deficient-like 1 (yeast) |
| 332 | BIRC5 | baculoviral IAP repeat-containing 5 |
| 11004 | KIF2C | kinesin family member 2C |
| 701 | BUB1B | BUB1 budding uninhibited by benzimidazoles 1 homolog beta (yeast) |
| 79682 | MLF1IP | MLF1 interacting protein |
| 1062 | CENPE | centromere protein E, 312kDa |
| 79019 | CENPM | centromere protein M |
| 1063 | CENPF | centromere protein F, 350/400ka (mitosin) |
| 55839 | CENPN | centromere protein N |
| 1058 | CENPA | centromere protein A |
| 10403 | NDC80 | NDC80 homolog, kinetochore complex component (S. cerevisiae) |
| 220134 | C18orf24 | chromosome 18 open reading frame 24 |
| 11130 | ZWINT | ZW10 interactor |
| 10615 | SPAG5 | sperm associated antigen 5 |
| 83540 | NUF2 | NUF2, NDC80 kinetochore complex component, homolog (S. cerevisiae) |
| 64946 | CENPH | centromere protein H |
| 151246 | SGOL2 | shugoshin-like 2 (S. pombe) |
| 699 | BUB1 | BUB1 budding uninhibited by benzimidazoles 1 homolog (yeast) |
| 55143 | CDCA8 | cell division cycle associated 8 |
| 54821 | ERCC6L | excision repair cross-complementing rodent repair deficiency, complementation group 6-like |

  
  
**MSCs batch 2 repressed and**GO:0005819**: 25 genes, expected 1.07652, P=1.33262e-27, P adjusted = 1.55134e-22

|  |  |  |
| --- | --- | --- |
| 9735 | KNTC1 | kinetochore associated 1 |
| 983 | CDC2 | cell division cycle 2, G1 to S and G2 to M |
| 9787 | DLGAP5 | discs, large (Drosophila) homolog-associated protein 5 |
| 26271 | FBXO5 | F-box protein 5 |
| 3832 | KIF11 | kinesin family member 11 |
| 4085 | MAD2L1 | MAD2 mitotic arrest deficient-like 1 (yeast) |
| 332 | BIRC5 | baculoviral IAP repeat-containing 5 |
| 701 | BUB1B | BUB1 budding uninhibited by benzimidazoles 1 homolog beta (yeast) |
| 29127 | RACGAP1 | Rac GTPase activating protein 1 |
| 259266 | ASPM | asp (abnormal spindle) homolog, microcephaly associated (Drosophila) |
| 1062 | CENPE | centromere protein E, 312kDa |
| 9928 | KIF14 | kinesin family member 14 |
| 22974 | TPX2 | TPX2, microtubule-associated, homolog (Xenopus laevis) |
| 1063 | CENPF | centromere protein F, 350/400ka (mitosin) |
| 220134 | C18orf24 | chromosome 18 open reading frame 24 |
| 7272 | TTK | TTK protein kinase |
| 6790 | AURKA | aurora kinase A |
| 990 | CDC6 | cell division cycle 6 homolog (S. cerevisiae) |
| 10615 | SPAG5 | sperm associated antigen 5 |
| 9493 | KIF23 | kinesin family member 23 |
| 56992 | KIF15 | kinesin family member 15 |
| 24137 | KIF4A | kinesin family member 4A |
| 991 | CDC20 | cell division cycle 20 homolog (S. cerevisiae) |
| 9055 | PRC1 | protein regulator of cytokinesis 1 |
| 699 | BUB1 | BUB1 budding uninhibited by benzimidazoles 1 homolog (yeast) |

  
  
**MSCs batch 2 repressed and**GO:0000777**: 17 genes, expected 0.494951, P=1.48737e-22, P adjusted = 1.73148e-17

|  |  |  |
| --- | --- | --- |
| 9735 | KNTC1 | kinetochore associated 1 |
| 57405 | SPC25 | SPC25, NDC80 kinetochore complex component, homolog (S. cerevisiae) |
| 701 | BUB1B | BUB1 budding uninhibited by benzimidazoles 1 homolog beta (yeast) |
| 79682 | MLF1IP | MLF1 interacting protein |
| 1062 | CENPE | centromere protein E, 312kDa |
| 79019 | CENPM | centromere protein M |
| 1063 | CENPF | centromere protein F, 350/400ka (mitosin) |
| 55839 | CENPN | centromere protein N |
| 1058 | CENPA | centromere protein A |
| 10403 | NDC80 | NDC80 homolog, kinetochore complex component (S. cerevisiae) |
| 220134 | C18orf24 | chromosome 18 open reading frame 24 |
| 11130 | ZWINT | ZW10 interactor |
| 10615 | SPAG5 | sperm associated antigen 5 |
| 83540 | NUF2 | NUF2, NDC80 kinetochore complex component, homolog (S. cerevisiae) |
| 64946 | CENPH | centromere protein H |
| 699 | BUB1 | BUB1 budding uninhibited by benzimidazoles 1 homolog (yeast) |
| 54821 | ERCC6L | excision repair cross-complementing rodent repair deficiency, complementation group 6-like |

  
  
**MSCs batch 2 repressed and**GO:0015630**: 40 genes, expected 5.70432, P=1.9056e-22, P adjusted = 2.21835e-17

|  |  |  |
| --- | --- | --- |
| 9735 | KNTC1 | kinetochore associated 1 |
| 983 | CDC2 | cell division cycle 2, G1 to S and G2 to M |
| 7153 | TOP2A | topoisomerase (DNA) II alpha 170kDa |
| 9787 | DLGAP5 | discs, large (Drosophila) homolog-associated protein 5 |
| 26271 | FBXO5 | F-box protein 5 |
| 10112 | KIF20A | kinesin family member 20A |
| 3832 | KIF11 | kinesin family member 11 |
| 891 | CCNB1 | cyclin B1 |
| 4085 | MAD2L1 | MAD2 mitotic arrest deficient-like 1 (yeast) |
| 332 | BIRC5 | baculoviral IAP repeat-containing 5 |
| 11004 | KIF2C | kinesin family member 2C |
| 701 | BUB1B | BUB1 budding uninhibited by benzimidazoles 1 homolog beta (yeast) |
| 29127 | RACGAP1 | Rac GTPase activating protein 1 |
| 259266 | ASPM | asp (abnormal spindle) homolog, microcephaly associated (Drosophila) |
| 1062 | CENPE | centromere protein E, 312kDa |
| 9928 | KIF14 | kinesin family member 14 |
| 4751 | NEK2 | NIMA (never in mitosis gene a)-related kinase 2 |
| 22974 | TPX2 | TPX2, microtubule-associated, homolog (Xenopus laevis) |
| 3925 | STMN1 | stathmin 1/oncoprotein 18 |
| 51203 | NUSAP1 | nucleolar and spindle associated protein 1 |
| 1063 | CENPF | centromere protein F, 350/400ka (mitosin) |
| 146909 | KIF18B | kinesin family member 18B |
| 220134 | C18orf24 | chromosome 18 open reading frame 24 |
| 9133 | CCNB2 | cyclin B2 |
| 8318 | CDC45L | CDC45 cell division cycle 45-like (S. cerevisiae) |
| 7272 | TTK | TTK protein kinase |
| 6790 | AURKA | aurora kinase A |
| 51512 | GTSE1 | G-2 and S-phase expressed 1 |
| 990 | CDC6 | cell division cycle 6 homolog (S. cerevisiae) |
| 10460 | TACC3 | transforming, acidic coiled-coil containing protein 3 |
| 10615 | SPAG5 | sperm associated antigen 5 |
| 9493 | KIF23 | kinesin family member 23 |
| 56992 | KIF15 | kinesin family member 15 |
| 24137 | KIF4A | kinesin family member 4A |
| 6780 | STAU1 | staufen, RNA binding protein, homolog 1 (Drosophila) |
| 991 | CDC20 | cell division cycle 20 homolog (S. cerevisiae) |
| 9055 | PRC1 | protein regulator of cytokinesis 1 |
| 699 | BUB1 | BUB1 budding uninhibited by benzimidazoles 1 homolog (yeast) |
| 81930 | KIF18A | kinesin family member 18A |
| 9585 | KIF20B | kinesin family member 20B |

  
  
**MSCs batch 2 repressed and**GO:0000779**: 17 genes, expected 0.507325, P=2.51322e-22, P adjusted = 2.92569e-17

|  |  |  |
| --- | --- | --- |
| 9735 | KNTC1 | kinetochore associated 1 |
| 57405 | SPC25 | SPC25, NDC80 kinetochore complex component, homolog (S. cerevisiae) |
| 701 | BUB1B | BUB1 budding uninhibited by benzimidazoles 1 homolog beta (yeast) |
| 79682 | MLF1IP | MLF1 interacting protein |
| 1062 | CENPE | centromere protein E, 312kDa |
| 79019 | CENPM | centromere protein M |
| 1063 | CENPF | centromere protein F, 350/400ka (mitosin) |
| 55839 | CENPN | centromere protein N |
| 1058 | CENPA | centromere protein A |
| 10403 | NDC80 | NDC80 homolog, kinetochore complex component (S. cerevisiae) |
| 220134 | C18orf24 | chromosome 18 open reading frame 24 |
| 11130 | ZWINT | ZW10 interactor |
| 10615 | SPAG5 | sperm associated antigen 5 |
| 83540 | NUF2 | NUF2, NDC80 kinetochore complex component, homolog (S. cerevisiae) |
| 64946 | CENPH | centromere protein H |
| 699 | BUB1 | BUB1 budding uninhibited by benzimidazoles 1 homolog (yeast) |
| 54821 | ERCC6L | excision repair cross-complementing rodent repair deficiency, complementation group 6-like |

  
  
**MSCs batch 2 repressed and**GO:0006259**: 40 genes, expected 5.92704, P=7.82332e-22, P adjusted = 9.10729e-17

|  |  |  |
| --- | --- | --- |
| 10635 | RAD51AP1 | RAD51 associated protein 1 |
| 7153 | TOP2A | topoisomerase (DNA) II alpha 170kDa |
| 2177 | FANCD2 | Fanconi anemia, complementation group D2 |
| 995 | CDC25C | cell division cycle 25 homolog C (S. pombe) |
| 3070 | HELLS | helicase, lymphoid-specific |
| 84057 | MND1 | meiotic nuclear divisions 1 homolog (S. cerevisiae) |
| 11168 | PSIP1 | PC4 and SFRS1 interacting protein 1 |
| 51514 | DTL | denticleless homolog (Drosophila) |
| 56852 | RAD18 | RAD18 homolog (S. cerevisiae) |
| 55215 | FANCI | Fanconi anemia, complementation group I |
| 5983 | RFC3 | replication factor C (activator 1) 3, 38kDa |
| 10714 | POLD3 | polymerase (DNA-directed), delta 3, accessory subunit |
| 4176 | MCM7 | minichromosome maintenance complex component 7 |
| 55388 | MCM10 | minichromosome maintenance complex component 10 |
| 6241 | RRM2 | ribonucleotide reductase M2 polypeptide |
| 23649 | POLA2 | polymerase (DNA directed), alpha 2 (70kD subunit) |
| 4171 | MCM2 | minichromosome maintenance complex component 2 |
| 9232 | PTTG1 | pituitary tumor-transforming 1 |
| 7083 | TK1 | thymidine kinase 1, soluble |
| 8318 | CDC45L | CDC45 cell division cycle 45-like (S. cerevisiae) |
| 10535 | RNASEH2A | ribonuclease H2, subunit A |
| 2956 | MSH6 | mutS homolog 6 (E. coli) |
| 7298 | TYMS | thymidylate synthetase |
| 23049 | SMG1 | PI-3-kinase-related kinase SMG-1 |
| 990 | CDC6 | cell division cycle 6 homolog (S. cerevisiae) |
| 51659 | GINS2 | GINS complex subunit 2 (Psf2 homolog) |
| 4174 | MCM5 | minichromosome maintenance complex component 5 |
| 3479 | IGF1 | insulin-like growth factor 1 (somatomedin C) |
| 29128 | UHRF1 | ubiquitin-like with PHD and ring finger domains 1 |
| 5557 | PRIM1 | primase, DNA, polypeptide 1 (49kDa) |
| 4173 | MCM4 | minichromosome maintenance complex component 4 |
| 993 | CDC25A | cell division cycle 25 homolog A (S. pombe) |
| 5591 | PRKDC | protein kinase, DNA-activated, catalytic polypeptide |
| 9134 | CCNE2 | cyclin E2 |
| 5985 | RFC5 | replication factor C (activator 1) 5, 36.5kDa |
| 55247 | NEIL3 | nei endonuclease VIII-like 3 (E. coli) |
| 9156 | EXO1 | exonuclease 1 |
| 3148 | HMGB2 | high-mobility group box 2 |
| 6240 | RRM1 | ribonucleotide reductase M1 |
| 10721 | POLQ | polymerase (DNA directed), theta |

  
  
**MSCs batch 2 repressed and**GO:0000776**: 18 genes, expected 0.655811, P=1.09651e-21, P adjusted = 1.27647e-16

|  |  |  |
| --- | --- | --- |
| 9735 | KNTC1 | kinetochore associated 1 |
| 57405 | SPC25 | SPC25, NDC80 kinetochore complex component, homolog (S. cerevisiae) |
| 4085 | MAD2L1 | MAD2 mitotic arrest deficient-like 1 (yeast) |
| 701 | BUB1B | BUB1 budding uninhibited by benzimidazoles 1 homolog beta (yeast) |
| 79682 | MLF1IP | MLF1 interacting protein |
| 1062 | CENPE | centromere protein E, 312kDa |
| 79019 | CENPM | centromere protein M |
| 1063 | CENPF | centromere protein F, 350/400ka (mitosin) |
| 55839 | CENPN | centromere protein N |
| 1058 | CENPA | centromere protein A |
| 10403 | NDC80 | NDC80 homolog, kinetochore complex component (S. cerevisiae) |
| 220134 | C18orf24 | chromosome 18 open reading frame 24 |
| 11130 | ZWINT | ZW10 interactor |
| 10615 | SPAG5 | sperm associated antigen 5 |
| 83540 | NUF2 | NUF2, NDC80 kinetochore complex component, homolog (S. cerevisiae) |
| 64946 | CENPH | centromere protein H |
| 699 | BUB1 | BUB1 budding uninhibited by benzimidazoles 1 homolog (yeast) |
| 54821 | ERCC6L | excision repair cross-complementing rodent repair deficiency, complementation group 6-like |

  
  
**MSCs batch 2 repressed and**GO:0006260**: 28 genes, expected 2.68511, P=1.52257e-20, P adjusted = 1.77245e-15

|  |  |  |
| --- | --- | --- |
| 7153 | TOP2A | topoisomerase (DNA) II alpha 170kDa |
| 995 | CDC25C | cell division cycle 25 homolog C (S. pombe) |
| 51514 | DTL | denticleless homolog (Drosophila) |
| 5983 | RFC3 | replication factor C (activator 1) 3, 38kDa |
| 10714 | POLD3 | polymerase (DNA-directed), delta 3, accessory subunit |
| 4176 | MCM7 | minichromosome maintenance complex component 7 |
| 55388 | MCM10 | minichromosome maintenance complex component 10 |
| 6241 | RRM2 | ribonucleotide reductase M2 polypeptide |
| 23649 | POLA2 | polymerase (DNA directed), alpha 2 (70kD subunit) |
| 4171 | MCM2 | minichromosome maintenance complex component 2 |
| 7083 | TK1 | thymidine kinase 1, soluble |
| 8318 | CDC45L | CDC45 cell division cycle 45-like (S. cerevisiae) |
| 10535 | RNASEH2A | ribonuclease H2, subunit A |
| 2956 | MSH6 | mutS homolog 6 (E. coli) |
| 7298 | TYMS | thymidylate synthetase |
| 990 | CDC6 | cell division cycle 6 homolog (S. cerevisiae) |
| 51659 | GINS2 | GINS complex subunit 2 (Psf2 homolog) |
| 4174 | MCM5 | minichromosome maintenance complex component 5 |
| 3479 | IGF1 | insulin-like growth factor 1 (somatomedin C) |
| 5557 | PRIM1 | primase, DNA, polypeptide 1 (49kDa) |
| 4173 | MCM4 | minichromosome maintenance complex component 4 |
| 993 | CDC25A | cell division cycle 25 homolog A (S. pombe) |
| 9134 | CCNE2 | cyclin E2 |
| 5985 | RFC5 | replication factor C (activator 1) 5, 36.5kDa |
| 9156 | EXO1 | exonuclease 1 |
| 3148 | HMGB2 | high-mobility group box 2 |
| 6240 | RRM1 | ribonucleotide reductase M1 |
| 10721 | POLQ | polymerase (DNA directed), theta |

  
  
**MSCs batch 2 repressed and**GO:0000793**: 19 genes, expected 1.10127, P=1.50734e-18, P adjusted = 1.75472e-13

|  |  |  |
| --- | --- | --- |
| 9735 | KNTC1 | kinetochore associated 1 |
| 57405 | SPC25 | SPC25, NDC80 kinetochore complex component, homolog (S. cerevisiae) |
| 701 | BUB1B | BUB1 budding uninhibited by benzimidazoles 1 homolog beta (yeast) |
| 79682 | MLF1IP | MLF1 interacting protein |
| 1062 | CENPE | centromere protein E, 312kDa |
| 79019 | CENPM | centromere protein M |
| 1063 | CENPF | centromere protein F, 350/400ka (mitosin) |
| 55839 | CENPN | centromere protein N |
| 1058 | CENPA | centromere protein A |
| 10403 | NDC80 | NDC80 homolog, kinetochore complex component (S. cerevisiae) |
| 220134 | C18orf24 | chromosome 18 open reading frame 24 |
| 11130 | ZWINT | ZW10 interactor |
| 10615 | SPAG5 | sperm associated antigen 5 |
| 83540 | NUF2 | NUF2, NDC80 kinetochore complex component, homolog (S. cerevisiae) |
| 10051 | SMC4 | structural maintenance of chromosomes 4 |
| 64946 | CENPH | centromere protein H |
| 699 | BUB1 | BUB1 budding uninhibited by benzimidazoles 1 homolog (yeast) |
| 54821 | ERCC6L | excision repair cross-complementing rodent repair deficiency, complementation group 6-like |
| 3148 | HMGB2 | high-mobility group box 2 |

  
  
**MSCs batch 2 repressed and**GO:0007346**: 20 genes, expected 1.29925, P=2.04183e-18, P adjusted = 2.37694e-13

|  |  |  |
| --- | --- | --- |
| 9735 | KNTC1 | kinetochore associated 1 |
| 9787 | DLGAP5 | discs, large (Drosophila) homolog-associated protein 5 |
| 26271 | FBXO5 | F-box protein 5 |
| 995 | CDC25C | cell division cycle 25 homolog C (S. pombe) |
| 4085 | MAD2L1 | MAD2 mitotic arrest deficient-like 1 (yeast) |
| 332 | BIRC5 | baculoviral IAP repeat-containing 5 |
| 701 | BUB1B | BUB1 budding uninhibited by benzimidazoles 1 homolog beta (yeast) |
| 4751 | NEK2 | NIMA (never in mitosis gene a)-related kinase 2 |
| 51203 | NUSAP1 | nucleolar and spindle associated protein 1 |
| 1063 | CENPF | centromere protein F, 350/400ka (mitosin) |
| 7272 | TTK | TTK protein kinase |
| 11130 | ZWINT | ZW10 interactor |
| 54443 | ANLN | anillin, actin binding protein |
| 51512 | GTSE1 | G-2 and S-phase expressed 1 |
| 990 | CDC6 | cell division cycle 6 homolog (S. cerevisiae) |
| 890 | CCNA2 | cyclin A2 |
| 11065 | UBE2C | ubiquitin-conjugating enzyme E2C |
| 3479 | IGF1 | insulin-like growth factor 1 (somatomedin C) |
| 699 | BUB1 | BUB1 budding uninhibited by benzimidazoles 1 homolog (yeast) |
| 9585 | KIF20B | kinesin family member 20B |

  
  
**MSCs batch 2 repressed and**GO:0044430**: 43 genes, expected 8.89675, P=6.73747e-18, P adjusted = 7.84322e-13

|  |  |  |
| --- | --- | --- |
| 9735 | KNTC1 | kinetochore associated 1 |
| 983 | CDC2 | cell division cycle 2, G1 to S and G2 to M |
| 7153 | TOP2A | topoisomerase (DNA) II alpha 170kDa |
| 9787 | DLGAP5 | discs, large (Drosophila) homolog-associated protein 5 |
| 26271 | FBXO5 | F-box protein 5 |
| 10112 | KIF20A | kinesin family member 20A |
| 3832 | KIF11 | kinesin family member 11 |
| 891 | CCNB1 | cyclin B1 |
| 4085 | MAD2L1 | MAD2 mitotic arrest deficient-like 1 (yeast) |
| 332 | BIRC5 | baculoviral IAP repeat-containing 5 |
| 11004 | KIF2C | kinesin family member 2C |
| 701 | BUB1B | BUB1 budding uninhibited by benzimidazoles 1 homolog beta (yeast) |
| 29127 | RACGAP1 | Rac GTPase activating protein 1 |
| 259266 | ASPM | asp (abnormal spindle) homolog, microcephaly associated (Drosophila) |
| 1062 | CENPE | centromere protein E, 312kDa |
| 9928 | KIF14 | kinesin family member 14 |
| 4751 | NEK2 | NIMA (never in mitosis gene a)-related kinase 2 |
| 22974 | TPX2 | TPX2, microtubule-associated, homolog (Xenopus laevis) |
| 3925 | STMN1 | stathmin 1/oncoprotein 18 |
| 51203 | NUSAP1 | nucleolar and spindle associated protein 1 |
| 1063 | CENPF | centromere protein F, 350/400ka (mitosin) |
| 146909 | KIF18B | kinesin family member 18B |
| 822 | CAPG | capping protein (actin filament), gelsolin-like |
| 220134 | C18orf24 | chromosome 18 open reading frame 24 |
| 8318 | CDC45L | CDC45 cell division cycle 45-like (S. cerevisiae) |
| 7272 | TTK | TTK protein kinase |
| 54443 | ANLN | anillin, actin binding protein |
| 6790 | AURKA | aurora kinase A |
| 51512 | GTSE1 | G-2 and S-phase expressed 1 |
| 990 | CDC6 | cell division cycle 6 homolog (S. cerevisiae) |
| 10460 | TACC3 | transforming, acidic coiled-coil containing protein 3 |
| 10615 | SPAG5 | sperm associated antigen 5 |
| 9493 | KIF23 | kinesin family member 23 |
| 56992 | KIF15 | kinesin family member 15 |
| 24137 | KIF4A | kinesin family member 4A |
| 4741 | NEFM | neurofilament, medium polypeptide |
| 6780 | STAU1 | staufen, RNA binding protein, homolog 1 (Drosophila) |
| 991 | CDC20 | cell division cycle 20 homolog (S. cerevisiae) |
| 9055 | PRC1 | protein regulator of cytokinesis 1 |
| 699 | BUB1 | BUB1 budding uninhibited by benzimidazoles 1 homolog (yeast) |
| 4001 | LMNB1 | lamin B1 |
| 81930 | KIF18A | kinesin family member 18A |
| 9585 | KIF20B | kinesin family member 20B |

  
  
**MSCs batch 2 repressed and**GO:0007059**: 16 genes, expected 0.791922, P=5.36904e-17, P adjusted = 6.25021e-12

|  |  |  |
| --- | --- | --- |
| 7153 | TOP2A | topoisomerase (DNA) II alpha 170kDa |
| 9787 | DLGAP5 | discs, large (Drosophila) homolog-associated protein 5 |
| 4085 | MAD2L1 | MAD2 mitotic arrest deficient-like 1 (yeast) |
| 113130 | CDCA5 | cell division cycle associated 5 |
| 1062 | CENPE | centromere protein E, 312kDa |
| 51203 | NUSAP1 | nucleolar and spindle associated protein 1 |
| 1063 | CENPF | centromere protein F, 350/400ka (mitosin) |
| 10403 | NDC80 | NDC80 homolog, kinetochore complex component (S. cerevisiae) |
| 23397 | NCAPH | non-SMC condensin I complex, subunit H |
| 9232 | PTTG1 | pituitary tumor-transforming 1 |
| 54892 | NCAPG2 | non-SMC condensin II complex, subunit G2 |
| 11130 | ZWINT | ZW10 interactor |
| 64151 | NCAPG | non-SMC condensin I complex, subunit G |
| 83540 | NUF2 | NUF2, NDC80 kinetochore complex component, homolog (S. cerevisiae) |
| 10051 | SMC4 | structural maintenance of chromosomes 4 |
| 151246 | SGOL2 | shugoshin-like 2 (S. pombe) |

  
  
**MSCs batch 2 repressed and**GO:0007051**: 12 genes, expected 0.346466, P=2.5245e-16, P adjusted = 2.93882e-11

|  |  |  |
| --- | --- | --- |
| 26271 | FBXO5 | F-box protein 5 |
| 3832 | KIF11 | kinesin family member 11 |
| 701 | BUB1B | BUB1 budding uninhibited by benzimidazoles 1 homolog beta (yeast) |
| 3925 | STMN1 | stathmin 1/oncoprotein 18 |
| 10403 | NDC80 | NDC80 homolog, kinetochore complex component (S. cerevisiae) |
| 7272 | TTK | TTK protein kinase |
| 11130 | ZWINT | ZW10 interactor |
| 6790 | AURKA | aurora kinase A |
| 10615 | SPAG5 | sperm associated antigen 5 |
| 9493 | KIF23 | kinesin family member 23 |
| 11065 | UBE2C | ubiquitin-conjugating enzyme E2C |
| 9055 | PRC1 | protein regulator of cytokinesis 1 |

  
  
**MSCs batch 2 repressed and**GO:0007017**: 24 genes, expected 2.85834, P=1.42104e-15, P adjusted = 1.65426e-10

|  |  |  |
| --- | --- | --- |
| 26271 | FBXO5 | F-box protein 5 |
| 10112 | KIF20A | kinesin family member 20A |
| 3832 | KIF11 | kinesin family member 11 |
| 11004 | KIF2C | kinesin family member 2C |
| 701 | BUB1B | BUB1 budding uninhibited by benzimidazoles 1 homolog beta (yeast) |
| 1062 | CENPE | centromere protein E, 312kDa |
| 9928 | KIF14 | kinesin family member 14 |
| 3925 | STMN1 | stathmin 1/oncoprotein 18 |
| 51203 | NUSAP1 | nucleolar and spindle associated protein 1 |
| 146909 | KIF18B | kinesin family member 18B |
| 10403 | NDC80 | NDC80 homolog, kinetochore complex component (S. cerevisiae) |
| 7272 | TTK | TTK protein kinase |
| 11130 | ZWINT | ZW10 interactor |
| 6790 | AURKA | aurora kinase A |
| 51512 | GTSE1 | G-2 and S-phase expressed 1 |
| 10615 | SPAG5 | sperm associated antigen 5 |
| 9493 | KIF23 | kinesin family member 23 |
| 56992 | KIF15 | kinesin family member 15 |
| 24137 | KIF4A | kinesin family member 4A |
| 4741 | NEFM | neurofilament, medium polypeptide |
| 11065 | UBE2C | ubiquitin-conjugating enzyme E2C |
| 9055 | PRC1 | protein regulator of cytokinesis 1 |
| 81930 | KIF18A | kinesin family member 18A |
| 9585 | KIF20B | kinesin family member 20B |

  
  
**MSCs batch 2 repressed and**GO:0051726**: 27 genes, expected 3.92249, P=3.26139e-15, P adjusted = 3.79664e-10

|  |  |  |
| --- | --- | --- |
| 9735 | KNTC1 | kinetochore associated 1 |
| 9787 | DLGAP5 | discs, large (Drosophila) homolog-associated protein 5 |
| 26271 | FBXO5 | F-box protein 5 |
| 995 | CDC25C | cell division cycle 25 homolog C (S. pombe) |
| 4085 | MAD2L1 | MAD2 mitotic arrest deficient-like 1 (yeast) |
| 332 | BIRC5 | baculoviral IAP repeat-containing 5 |
| 701 | BUB1B | BUB1 budding uninhibited by benzimidazoles 1 homolog beta (yeast) |
| 4751 | NEK2 | NIMA (never in mitosis gene a)-related kinase 2 |
| 51203 | NUSAP1 | nucleolar and spindle associated protein 1 |
| 1063 | CENPF | centromere protein F, 350/400ka (mitosin) |
| 8318 | CDC45L | CDC45 cell division cycle 45-like (S. cerevisiae) |
| 7272 | TTK | TTK protein kinase |
| 11130 | ZWINT | ZW10 interactor |
| 54443 | ANLN | anillin, actin binding protein |
| 51512 | GTSE1 | G-2 and S-phase expressed 1 |
| 990 | CDC6 | cell division cycle 6 homolog (S. cerevisiae) |
| 10460 | TACC3 | transforming, acidic coiled-coil containing protein 3 |
| 890 | CCNA2 | cyclin A2 |
| 1033 | CDKN3 | cyclin-dependent kinase inhibitor 3 |
| 11065 | UBE2C | ubiquitin-conjugating enzyme E2C |
| 3479 | IGF1 | insulin-like growth factor 1 (somatomedin C) |
| 5933 | RBL1 | retinoblastoma-like 1 (p107) |
| 993 | CDC25A | cell division cycle 25 homolog A (S. pombe) |
| 699 | BUB1 | BUB1 budding uninhibited by benzimidazoles 1 homolog (yeast) |
| 9134 | CCNE2 | cyclin E2 |
| 8914 | TIMELESS | timeless homolog (Drosophila) |
| 9585 | KIF20B | kinesin family member 20B |

  
  
**MSCs batch 2 repressed and**GO:0005856**: 50 genes, expected 14.3412, P=6.22832e-15, P adjusted = 7.25051e-10

|  |  |  |
| --- | --- | --- |
| 9735 | KNTC1 | kinetochore associated 1 |
| 983 | CDC2 | cell division cycle 2, G1 to S and G2 to M |
| 7153 | TOP2A | topoisomerase (DNA) II alpha 170kDa |
| 9787 | DLGAP5 | discs, large (Drosophila) homolog-associated protein 5 |
| 26271 | FBXO5 | F-box protein 5 |
| 10112 | KIF20A | kinesin family member 20A |
| 3832 | KIF11 | kinesin family member 11 |
| 891 | CCNB1 | cyclin B1 |
| 4085 | MAD2L1 | MAD2 mitotic arrest deficient-like 1 (yeast) |
| 332 | BIRC5 | baculoviral IAP repeat-containing 5 |
| 11004 | KIF2C | kinesin family member 2C |
| 701 | BUB1B | BUB1 budding uninhibited by benzimidazoles 1 homolog beta (yeast) |
| 11013 | TMSL8 | thymosin-like 8 |
| 29127 | RACGAP1 | Rac GTPase activating protein 1 |
| 259266 | ASPM | asp (abnormal spindle) homolog, microcephaly associated (Drosophila) |
| 1062 | CENPE | centromere protein E, 312kDa |
| 9928 | KIF14 | kinesin family member 14 |
| 4751 | NEK2 | NIMA (never in mitosis gene a)-related kinase 2 |
| 22974 | TPX2 | TPX2, microtubule-associated, homolog (Xenopus laevis) |
| 3925 | STMN1 | stathmin 1/oncoprotein 18 |
| 51203 | NUSAP1 | nucleolar and spindle associated protein 1 |
| 1063 | CENPF | centromere protein F, 350/400ka (mitosin) |
| 23136 | EPB41L3 | erythrocyte membrane protein band 4.1-like 3 |
| 146909 | KIF18B | kinesin family member 18B |
| 7223 | TRPC4 | transient receptor potential cation channel, subfamily C, member 4 |
| 822 | CAPG | capping protein (actin filament), gelsolin-like |
| 220134 | C18orf24 | chromosome 18 open reading frame 24 |
| 23397 | NCAPH | non-SMC condensin I complex, subunit H |
| 9133 | CCNB2 | cyclin B2 |
| 8318 | CDC45L | CDC45 cell division cycle 45-like (S. cerevisiae) |
| 7272 | TTK | TTK protein kinase |
| 339855 | KY | kyphoscoliosis peptidase |
| 54443 | ANLN | anillin, actin binding protein |
| 6790 | AURKA | aurora kinase A |
| 51512 | GTSE1 | G-2 and S-phase expressed 1 |
| 990 | CDC6 | cell division cycle 6 homolog (S. cerevisiae) |
| 10460 | TACC3 | transforming, acidic coiled-coil containing protein 3 |
| 10615 | SPAG5 | sperm associated antigen 5 |
| 9493 | KIF23 | kinesin family member 23 |
| 56992 | KIF15 | kinesin family member 15 |
| 24137 | KIF4A | kinesin family member 4A |
| 4741 | NEFM | neurofilament, medium polypeptide |
| 10391 | CORO2B | coronin, actin binding protein, 2B |
| 6780 | STAU1 | staufen, RNA binding protein, homolog 1 (Drosophila) |
| 991 | CDC20 | cell division cycle 20 homolog (S. cerevisiae) |
| 9055 | PRC1 | protein regulator of cytokinesis 1 |
| 699 | BUB1 | BUB1 budding uninhibited by benzimidazoles 1 homolog (yeast) |
| 4001 | LMNB1 | lamin B1 |
| 81930 | KIF18A | kinesin family member 18A |
| 9585 | KIF20B | kinesin family member 20B |

  
  
**MSCs batch 2 repressed and**GO:0000070**: 11 genes, expected 0.383587, P=5.68615e-14, P adjusted = 6.61936e-09

|  |  |  |
| --- | --- | --- |
| 9787 | DLGAP5 | discs, large (Drosophila) homolog-associated protein 5 |
| 4085 | MAD2L1 | MAD2 mitotic arrest deficient-like 1 (yeast) |
| 113130 | CDCA5 | cell division cycle associated 5 |
| 1062 | CENPE | centromere protein E, 312kDa |
| 51203 | NUSAP1 | nucleolar and spindle associated protein 1 |
| 10403 | NDC80 | NDC80 homolog, kinetochore complex component (S. cerevisiae) |
| 23397 | NCAPH | non-SMC condensin I complex, subunit H |
| 54892 | NCAPG2 | non-SMC condensin II complex, subunit G2 |
| 11130 | ZWINT | ZW10 interactor |
| 64151 | NCAPG | non-SMC condensin I complex, subunit G |
| 10051 | SMC4 | structural maintenance of chromosomes 4 |

  
  
**MSCs batch 2 repressed and**GO:0043234**: 60 genes, expected 21.0602, P=6.39835e-14, P adjusted = 7.44845e-09

|  |  |  |
| --- | --- | --- |
| 9735 | KNTC1 | kinetochore associated 1 |
| 57405 | SPC25 | SPC25, NDC80 kinetochore complex component, homolog (S. cerevisiae) |
| 7153 | TOP2A | topoisomerase (DNA) II alpha 170kDa |
| 10112 | KIF20A | kinesin family member 20A |
| 3832 | KIF11 | kinesin family member 11 |
| 4085 | MAD2L1 | MAD2 mitotic arrest deficient-like 1 (yeast) |
| 332 | BIRC5 | baculoviral IAP repeat-containing 5 |
| 11004 | KIF2C | kinesin family member 2C |
| 10762 | NUP50 | nucleoporin 50kDa |
| 701 | BUB1B | BUB1 budding uninhibited by benzimidazoles 1 homolog beta (yeast) |
| 113130 | CDCA5 | cell division cycle associated 5 |
| 79682 | MLF1IP | MLF1 interacting protein |
| 1062 | CENPE | centromere protein E, 312kDa |
| 2295 | FOXF2 | forkhead box F2 |
| 9928 | KIF14 | kinesin family member 14 |
| 5983 | RFC3 | replication factor C (activator 1) 3, 38kDa |
| 3676 | ITGA4 | integrin, alpha 4 (antigen CD49D, alpha 4 subunit of VLA-4 receptor) |
| 79019 | CENPM | centromere protein M |
| 10714 | POLD3 | polymerase (DNA-directed), delta 3, accessory subunit |
| 1063 | CENPF | centromere protein F, 350/400ka (mitosin) |
| 55839 | CENPN | centromere protein N |
| 4176 | MCM7 | minichromosome maintenance complex component 7 |
| 3122 | HLA-DRA | major histocompatibility complex, class II, DR alpha |
| 146909 | KIF18B | kinesin family member 18B |
| 7223 | TRPC4 | transient receptor potential cation channel, subfamily C, member 4 |
| 23649 | POLA2 | polymerase (DNA directed), alpha 2 (70kD subunit) |
| 1058 | CENPA | centromere protein A |
| 10403 | NDC80 | NDC80 homolog, kinetochore complex component (S. cerevisiae) |
| 154 | ADRB2 | adrenergic, beta-2-, receptor, surface |
| 822 | CAPG | capping protein (actin filament), gelsolin-like |
| 6328 | SCN3A | sodium channel, voltage-gated, type III, alpha subunit |
| 1439 | CSF2RB | colony stimulating factor 2 receptor, beta, low-affinity (granulocyte-macrophage) |
| 4171 | MCM2 | minichromosome maintenance complex component 2 |
| 220134 | C18orf24 | chromosome 18 open reading frame 24 |
| 2146 | EZH2 | enhancer of zeste homolog 2 (Drosophila) |
| 10875 | FGL2 | fibrinogen-like 2 |
| 11130 | ZWINT | ZW10 interactor |
| 2956 | MSH6 | mutS homolog 6 (E. coli) |
| 10615 | SPAG5 | sperm associated antigen 5 |
| 9493 | KIF23 | kinesin family member 23 |
| 8515 | ITGA10 | integrin, alpha 10 |
| 56992 | KIF15 | kinesin family member 15 |
| 24137 | KIF4A | kinesin family member 4A |
| 83540 | NUF2 | NUF2, NDC80 kinetochore complex component, homolog (S. cerevisiae) |
| 10051 | SMC4 | structural maintenance of chromosomes 4 |
| 6780 | STAU1 | staufen, RNA binding protein, homolog 1 (Drosophila) |
| 3479 | IGF1 | insulin-like growth factor 1 (somatomedin C) |
| 5933 | RBL1 | retinoblastoma-like 1 (p107) |
| 5557 | PRIM1 | primase, DNA, polypeptide 1 (49kDa) |
| 79733 | E2F8 | E2F transcription factor 8 |
| 64946 | CENPH | centromere protein H |
| 151246 | SGOL2 | shugoshin-like 2 (S. pombe) |
| 5591 | PRKDC | protein kinase, DNA-activated, catalytic polypeptide |
| 699 | BUB1 | BUB1 budding uninhibited by benzimidazoles 1 homolog (yeast) |
| 55143 | CDCA8 | cell division cycle associated 8 |
| 54821 | ERCC6L | excision repair cross-complementing rodent repair deficiency, complementation group 6-like |
| 5985 | RFC5 | replication factor C (activator 1) 5, 36.5kDa |
| 81930 | KIF18A | kinesin family member 18A |
| 9585 | KIF20B | kinesin family member 20B |
| 6240 | RRM1 | ribonucleotide reductase M1 |

  
  
**MSCs batch 2 repressed and**GO:0000819**: 11 genes, expected 0.395961, P=8.57067e-14, P adjusted = 9.97728e-09

|  |  |  |
| --- | --- | --- |
| 9787 | DLGAP5 | discs, large (Drosophila) homolog-associated protein 5 |
| 4085 | MAD2L1 | MAD2 mitotic arrest deficient-like 1 (yeast) |
| 113130 | CDCA5 | cell division cycle associated 5 |
| 1062 | CENPE | centromere protein E, 312kDa |
| 51203 | NUSAP1 | nucleolar and spindle associated protein 1 |
| 10403 | NDC80 | NDC80 homolog, kinetochore complex component (S. cerevisiae) |
| 23397 | NCAPH | non-SMC condensin I complex, subunit H |
| 54892 | NCAPG2 | non-SMC condensin II complex, subunit G2 |
| 11130 | ZWINT | ZW10 interactor |
| 64151 | NCAPG | non-SMC condensin I complex, subunit G |
| 10051 | SMC4 | structural maintenance of chromosomes 4 |

  
  
**MSCs batch 2 repressed and**GO:0000075**: 13 genes, expected 0.767175, P=5.65917e-13, P adjusted = 6.58795e-08

|  |  |  |
| --- | --- | --- |
| 9735 | KNTC1 | kinetochore associated 1 |
| 4085 | MAD2L1 | MAD2 mitotic arrest deficient-like 1 (yeast) |
| 332 | BIRC5 | baculoviral IAP repeat-containing 5 |
| 701 | BUB1B | BUB1 budding uninhibited by benzimidazoles 1 homolog beta (yeast) |
| 1063 | CENPF | centromere protein F, 350/400ka (mitosin) |
| 8318 | CDC45L | CDC45 cell division cycle 45-like (S. cerevisiae) |
| 7272 | TTK | TTK protein kinase |
| 11130 | ZWINT | ZW10 interactor |
| 51512 | GTSE1 | G-2 and S-phase expressed 1 |
| 990 | CDC6 | cell division cycle 6 homolog (S. cerevisiae) |
| 890 | CCNA2 | cyclin A2 |
| 699 | BUB1 | BUB1 budding uninhibited by benzimidazoles 1 homolog (yeast) |
| 9134 | CCNE2 | cyclin E2 |

  
  
**MSCs batch 2 repressed and**GO:0031981**: 46 genes, expected 13.9205, P=6.16398e-13, P adjusted = 7.17562e-08

|  |  |  |
| --- | --- | --- |
| 983 | CDC2 | cell division cycle 2, G1 to S and G2 to M |
| 7153 | TOP2A | topoisomerase (DNA) II alpha 170kDa |
| 26271 | FBXO5 | F-box protein 5 |
| 10112 | KIF20A | kinesin family member 20A |
| 891 | CCNB1 | cyclin B1 |
| 51422 | PRKAG2 | protein kinase, AMP-activated, gamma 2 non-catalytic subunit |
| 995 | CDC25C | cell division cycle 25 homolog C (S. pombe) |
| 332 | BIRC5 | baculoviral IAP repeat-containing 5 |
| 10762 | NUP50 | nucleoporin 50kDa |
| 2295 | FOXF2 | forkhead box F2 |
| 5983 | RFC3 | replication factor C (activator 1) 3, 38kDa |
| 27330 | RPS6KA6 | ribosomal protein S6 kinase, 90kDa, polypeptide 6 |
| 4288 | MKI67 | antigen identified by monoclonal antibody Ki-67 |
| 10714 | POLD3 | polymerase (DNA-directed), delta 3, accessory subunit |
| 1063 | CENPF | centromere protein F, 350/400ka (mitosin) |
| 4176 | MCM7 | minichromosome maintenance complex component 7 |
| 55388 | MCM10 | minichromosome maintenance complex component 10 |
| 23649 | POLA2 | polymerase (DNA directed), alpha 2 (70kD subunit) |
| 4171 | MCM2 | minichromosome maintenance complex component 2 |
| 8318 | CDC45L | CDC45 cell division cycle 45-like (S. cerevisiae) |
| 2146 | EZH2 | enhancer of zeste homolog 2 (Drosophila) |
| 145508 | C14orf145 | chromosome 14 open reading frame 145 |
| 55320 | C14orf106 | chromosome 14 open reading frame 106 |
| 990 | CDC6 | cell division cycle 6 homolog (S. cerevisiae) |
| 890 | CCNA2 | cyclin A2 |
| 55975 | KLHL7 | kelch-like 7 (Drosophila) |
| 9493 | KIF23 | kinesin family member 23 |
| 24137 | KIF4A | kinesin family member 4A |
| 4174 | MCM5 | minichromosome maintenance complex component 5 |
| 991 | CDC20 | cell division cycle 20 homolog (S. cerevisiae) |
| 11065 | UBE2C | ubiquitin-conjugating enzyme E2C |
| 5933 | RBL1 | retinoblastoma-like 1 (p107) |
| 5557 | PRIM1 | primase, DNA, polypeptide 1 (49kDa) |
| 4173 | MCM4 | minichromosome maintenance complex component 4 |
| 993 | CDC25A | cell division cycle 25 homolog A (S. pombe) |
| 79733 | E2F8 | E2F transcription factor 8 |
| 5591 | PRKDC | protein kinase, DNA-activated, catalytic polypeptide |
| 11339 | OIP5 | Opa interacting protein 5 |
| 9134 | CCNE2 | cyclin E2 |
| 8487 | SIP1 | survival of motor neuron protein interacting protein 1 |
| 4001 | LMNB1 | lamin B1 |
| 5985 | RFC5 | replication factor C (activator 1) 5, 36.5kDa |
| 9585 | KIF20B | kinesin family member 20B |
| 3148 | HMGB2 | high-mobility group box 2 |
| 7133 | TNFRSF1B | tumor necrosis factor receptor superfamily, member 1B |
| 10721 | POLQ | polymerase (DNA directed), theta |

  
  
**MSCs batch 2 repressed and**GO:0005654**: 35 genes, expected 8.51317, P=1.23679e-12, P adjusted = 1.43978e-07

|  |  |  |
| --- | --- | --- |
| 983 | CDC2 | cell division cycle 2, G1 to S and G2 to M |
| 7153 | TOP2A | topoisomerase (DNA) II alpha 170kDa |
| 26271 | FBXO5 | F-box protein 5 |
| 10112 | KIF20A | kinesin family member 20A |
| 891 | CCNB1 | cyclin B1 |
| 51422 | PRKAG2 | protein kinase, AMP-activated, gamma 2 non-catalytic subunit |
| 995 | CDC25C | cell division cycle 25 homolog C (S. pombe) |
| 10762 | NUP50 | nucleoporin 50kDa |
| 2295 | FOXF2 | forkhead box F2 |
| 5983 | RFC3 | replication factor C (activator 1) 3, 38kDa |
| 10714 | POLD3 | polymerase (DNA-directed), delta 3, accessory subunit |
| 4176 | MCM7 | minichromosome maintenance complex component 7 |
| 55388 | MCM10 | minichromosome maintenance complex component 10 |
| 23649 | POLA2 | polymerase (DNA directed), alpha 2 (70kD subunit) |
| 4171 | MCM2 | minichromosome maintenance complex component 2 |
| 8318 | CDC45L | CDC45 cell division cycle 45-like (S. cerevisiae) |
| 2146 | EZH2 | enhancer of zeste homolog 2 (Drosophila) |
| 990 | CDC6 | cell division cycle 6 homolog (S. cerevisiae) |
| 890 | CCNA2 | cyclin A2 |
| 9493 | KIF23 | kinesin family member 23 |
| 4174 | MCM5 | minichromosome maintenance complex component 5 |
| 991 | CDC20 | cell division cycle 20 homolog (S. cerevisiae) |
| 11065 | UBE2C | ubiquitin-conjugating enzyme E2C |
| 5933 | RBL1 | retinoblastoma-like 1 (p107) |
| 5557 | PRIM1 | primase, DNA, polypeptide 1 (49kDa) |
| 4173 | MCM4 | minichromosome maintenance complex component 4 |
| 993 | CDC25A | cell division cycle 25 homolog A (S. pombe) |
| 79733 | E2F8 | E2F transcription factor 8 |
| 5591 | PRKDC | protein kinase, DNA-activated, catalytic polypeptide |
| 11339 | OIP5 | Opa interacting protein 5 |
| 9134 | CCNE2 | cyclin E2 |
| 8487 | SIP1 | survival of motor neuron protein interacting protein 1 |
| 5985 | RFC5 | replication factor C (activator 1) 5, 36.5kDa |
| 9585 | KIF20B | kinesin family member 20B |
| 10721 | POLQ | polymerase (DNA directed), theta |

  
  
**MSCs batch 2 repressed and**GO:0000226**: 15 genes, expected 1.29925, P=3.3144e-12, P adjusted = 3.85836e-07

|  |  |  |
| --- | --- | --- |
| 26271 | FBXO5 | F-box protein 5 |
| 3832 | KIF11 | kinesin family member 11 |
| 11004 | KIF2C | kinesin family member 2C |
| 701 | BUB1B | BUB1 budding uninhibited by benzimidazoles 1 homolog beta (yeast) |
| 3925 | STMN1 | stathmin 1/oncoprotein 18 |
| 51203 | NUSAP1 | nucleolar and spindle associated protein 1 |
| 10403 | NDC80 | NDC80 homolog, kinetochore complex component (S. cerevisiae) |
| 7272 | TTK | TTK protein kinase |
| 11130 | ZWINT | ZW10 interactor |
| 6790 | AURKA | aurora kinase A |
| 10615 | SPAG5 | sperm associated antigen 5 |
| 9493 | KIF23 | kinesin family member 23 |
| 4741 | NEFM | neurofilament, medium polypeptide |
| 11065 | UBE2C | ubiquitin-conjugating enzyme E2C |
| 9055 | PRC1 | protein regulator of cytokinesis 1 |

  
  
**MSCs batch 2 repressed and**GO:0044428**: 52 genes, expected 18.2018, P=4.03015e-12, P adjusted = 4.69158e-07

|  |  |  |
| --- | --- | --- |
| 983 | CDC2 | cell division cycle 2, G1 to S and G2 to M |
| 7153 | TOP2A | topoisomerase (DNA) II alpha 170kDa |
| 26271 | FBXO5 | F-box protein 5 |
| 10112 | KIF20A | kinesin family member 20A |
| 891 | CCNB1 | cyclin B1 |
| 51422 | PRKAG2 | protein kinase, AMP-activated, gamma 2 non-catalytic subunit |
| 995 | CDC25C | cell division cycle 25 homolog C (S. pombe) |
| 51514 | DTL | denticleless homolog (Drosophila) |
| 332 | BIRC5 | baculoviral IAP repeat-containing 5 |
| 10762 | NUP50 | nucleoporin 50kDa |
| 701 | BUB1B | BUB1 budding uninhibited by benzimidazoles 1 homolog beta (yeast) |
| 2295 | FOXF2 | forkhead box F2 |
| 5983 | RFC3 | replication factor C (activator 1) 3, 38kDa |
| 27330 | RPS6KA6 | ribosomal protein S6 kinase, 90kDa, polypeptide 6 |
| 4288 | MKI67 | antigen identified by monoclonal antibody Ki-67 |
| 10714 | POLD3 | polymerase (DNA-directed), delta 3, accessory subunit |
| 1063 | CENPF | centromere protein F, 350/400ka (mitosin) |
| 4176 | MCM7 | minichromosome maintenance complex component 7 |
| 55388 | MCM10 | minichromosome maintenance complex component 10 |
| 7112 | TMPO | thymopoietin |
| 23649 | POLA2 | polymerase (DNA directed), alpha 2 (70kD subunit) |
| 4171 | MCM2 | minichromosome maintenance complex component 2 |
| 89795 | NAV3 | neuron navigator 3 |
| 8318 | CDC45L | CDC45 cell division cycle 45-like (S. cerevisiae) |
| 2146 | EZH2 | enhancer of zeste homolog 2 (Drosophila) |
| 145508 | C14orf145 | chromosome 14 open reading frame 145 |
| 2956 | MSH6 | mutS homolog 6 (E. coli) |
| 55320 | C14orf106 | chromosome 14 open reading frame 106 |
| 990 | CDC6 | cell division cycle 6 homolog (S. cerevisiae) |
| 890 | CCNA2 | cyclin A2 |
| 55975 | KLHL7 | kelch-like 7 (Drosophila) |
| 9493 | KIF23 | kinesin family member 23 |
| 24137 | KIF4A | kinesin family member 4A |
| 4174 | MCM5 | minichromosome maintenance complex component 5 |
| 991 | CDC20 | cell division cycle 20 homolog (S. cerevisiae) |
| 11065 | UBE2C | ubiquitin-conjugating enzyme E2C |
| 5933 | RBL1 | retinoblastoma-like 1 (p107) |
| 5557 | PRIM1 | primase, DNA, polypeptide 1 (49kDa) |
| 4173 | MCM4 | minichromosome maintenance complex component 4 |
| 993 | CDC25A | cell division cycle 25 homolog A (S. pombe) |
| 79733 | E2F8 | E2F transcription factor 8 |
| 5591 | PRKDC | protein kinase, DNA-activated, catalytic polypeptide |
| 11339 | OIP5 | Opa interacting protein 5 |
| 9134 | CCNE2 | cyclin E2 |
| 8487 | SIP1 | survival of motor neuron protein interacting protein 1 |
| 4001 | LMNB1 | lamin B1 |
| 8914 | TIMELESS | timeless homolog (Drosophila) |
| 5985 | RFC5 | replication factor C (activator 1) 5, 36.5kDa |
| 9585 | KIF20B | kinesin family member 20B |
| 3148 | HMGB2 | high-mobility group box 2 |
| 7133 | TNFRSF1B | tumor necrosis factor receptor superfamily, member 1B |
| 10721 | POLQ | polymerase (DNA directed), theta |

  
  
**MSCs batch 2 repressed and**GO:0006261**: 13 genes, expected 0.903286, P=5.20938e-12, P adjusted = 6.06434e-07

|  |  |  |
| --- | --- | --- |
| 5983 | RFC3 | replication factor C (activator 1) 3, 38kDa |
| 10714 | POLD3 | polymerase (DNA-directed), delta 3, accessory subunit |
| 4176 | MCM7 | minichromosome maintenance complex component 7 |
| 4171 | MCM2 | minichromosome maintenance complex component 2 |
| 8318 | CDC45L | CDC45 cell division cycle 45-like (S. cerevisiae) |
| 2956 | MSH6 | mutS homolog 6 (E. coli) |
| 990 | CDC6 | cell division cycle 6 homolog (S. cerevisiae) |
| 4174 | MCM5 | minichromosome maintenance complex component 5 |
| 5557 | PRIM1 | primase, DNA, polypeptide 1 (49kDa) |
| 4173 | MCM4 | minichromosome maintenance complex component 4 |
| 9134 | CCNE2 | cyclin E2 |
| 9156 | EXO1 | exonuclease 1 |
| 3148 | HMGB2 | high-mobility group box 2 |

  
  
**MSCs batch 2 repressed and**GO:0010564**: 12 genes, expected 0.754801, P=1.0078e-11, P adjusted = 1.1732e-06

|  |  |  |
| --- | --- | --- |
| 9735 | KNTC1 | kinetochore associated 1 |
| 9787 | DLGAP5 | discs, large (Drosophila) homolog-associated protein 5 |
| 26271 | FBXO5 | F-box protein 5 |
| 995 | CDC25C | cell division cycle 25 homolog C (S. pombe) |
| 332 | BIRC5 | baculoviral IAP repeat-containing 5 |
| 4751 | NEK2 | NIMA (never in mitosis gene a)-related kinase 2 |
| 51203 | NUSAP1 | nucleolar and spindle associated protein 1 |
| 54443 | ANLN | anillin, actin binding protein |
| 11065 | UBE2C | ubiquitin-conjugating enzyme E2C |
| 3479 | IGF1 | insulin-like growth factor 1 (somatomedin C) |
| 8914 | TIMELESS | timeless homolog (Drosophila) |
| 9585 | KIF20B | kinesin family member 20B |

  
  
**MSCs batch 2 repressed and**GO:0006974**: 23 genes, expected 3.93486, P=1.18848e-11, P adjusted = 1.38353e-06

|  |  |  |
| --- | --- | --- |
| 10635 | RAD51AP1 | RAD51 associated protein 1 |
| 7153 | TOP2A | topoisomerase (DNA) II alpha 170kDa |
| 2177 | FANCD2 | Fanconi anemia, complementation group D2 |
| 51514 | DTL | denticleless homolog (Drosophila) |
| 56852 | RAD18 | RAD18 homolog (S. cerevisiae) |
| 55215 | FANCI | Fanconi anemia, complementation group I |
| 5983 | RFC3 | replication factor C (activator 1) 3, 38kDa |
| 10714 | POLD3 | polymerase (DNA-directed), delta 3, accessory subunit |
| 4176 | MCM7 | minichromosome maintenance complex component 7 |
| 9232 | PTTG1 | pituitary tumor-transforming 1 |
| 2956 | MSH6 | mutS homolog 6 (E. coli) |
| 7298 | TYMS | thymidylate synthetase |
| 23049 | SMG1 | PI-3-kinase-related kinase SMG-1 |
| 51512 | GTSE1 | G-2 and S-phase expressed 1 |
| 890 | CCNA2 | cyclin A2 |
| 29128 | UHRF1 | ubiquitin-like with PHD and ring finger domains 1 |
| 5591 | PRKDC | protein kinase, DNA-activated, catalytic polypeptide |
| 8914 | TIMELESS | timeless homolog (Drosophila) |
| 5985 | RFC5 | replication factor C (activator 1) 5, 36.5kDa |
| 55247 | NEIL3 | nei endonuclease VIII-like 3 (E. coli) |
| 9156 | EXO1 | exonuclease 1 |
| 3148 | HMGB2 | high-mobility group box 2 |
| 10721 | POLQ | polymerase (DNA directed), theta |

  
  
**MSCs batch 2 repressed and**GO:0005874**: 20 genes, expected 2.90784, P=1.47229e-11, P adjusted = 1.71393e-06

|  |  |  |
| --- | --- | --- |
| 9735 | KNTC1 | kinetochore associated 1 |
| 983 | CDC2 | cell division cycle 2, G1 to S and G2 to M |
| 10112 | KIF20A | kinesin family member 20A |
| 3832 | KIF11 | kinesin family member 11 |
| 332 | BIRC5 | baculoviral IAP repeat-containing 5 |
| 11004 | KIF2C | kinesin family member 2C |
| 29127 | RACGAP1 | Rac GTPase activating protein 1 |
| 1062 | CENPE | centromere protein E, 312kDa |
| 9928 | KIF14 | kinesin family member 14 |
| 3925 | STMN1 | stathmin 1/oncoprotein 18 |
| 51203 | NUSAP1 | nucleolar and spindle associated protein 1 |
| 146909 | KIF18B | kinesin family member 18B |
| 51512 | GTSE1 | G-2 and S-phase expressed 1 |
| 10615 | SPAG5 | sperm associated antigen 5 |
| 9493 | KIF23 | kinesin family member 23 |
| 56992 | KIF15 | kinesin family member 15 |
| 24137 | KIF4A | kinesin family member 4A |
| 9055 | PRC1 | protein regulator of cytokinesis 1 |
| 81930 | KIF18A | kinesin family member 18A |
| 9585 | KIF20B | kinesin family member 20B |

  
  
**MSCs batch 2 repressed and**GO:0043233**: 49 genes, expected 17.6203, P=4.95298e-11, P adjusted = 5.76586e-06

|  |  |  |
| --- | --- | --- |
| 983 | CDC2 | cell division cycle 2, G1 to S and G2 to M |
| 7153 | TOP2A | topoisomerase (DNA) II alpha 170kDa |
| 26271 | FBXO5 | F-box protein 5 |
| 10112 | KIF20A | kinesin family member 20A |
| 891 | CCNB1 | cyclin B1 |
| 51422 | PRKAG2 | protein kinase, AMP-activated, gamma 2 non-catalytic subunit |
| 995 | CDC25C | cell division cycle 25 homolog C (S. pombe) |
| 332 | BIRC5 | baculoviral IAP repeat-containing 5 |
| 10762 | NUP50 | nucleoporin 50kDa |
| 2295 | FOXF2 | forkhead box F2 |
| 5983 | RFC3 | replication factor C (activator 1) 3, 38kDa |
| 27330 | RPS6KA6 | ribosomal protein S6 kinase, 90kDa, polypeptide 6 |
| 4288 | MKI67 | antigen identified by monoclonal antibody Ki-67 |
| 10714 | POLD3 | polymerase (DNA-directed), delta 3, accessory subunit |
| 1063 | CENPF | centromere protein F, 350/400ka (mitosin) |
| 4176 | MCM7 | minichromosome maintenance complex component 7 |
| 55388 | MCM10 | minichromosome maintenance complex component 10 |
| 23649 | POLA2 | polymerase (DNA directed), alpha 2 (70kD subunit) |
| 4171 | MCM2 | minichromosome maintenance complex component 2 |
| 8318 | CDC45L | CDC45 cell division cycle 45-like (S. cerevisiae) |
| 2146 | EZH2 | enhancer of zeste homolog 2 (Drosophila) |
| 9957 | HS3ST1 | heparan sulfate (glucosamine) 3-O-sulfotransferase 1 |
| 145508 | C14orf145 | chromosome 14 open reading frame 145 |
| 55320 | C14orf106 | chromosome 14 open reading frame 106 |
| 5743 | PTGS2 | prostaglandin-endoperoxide synthase 2 (prostaglandin G/H synthase and cyclooxygenase) |
| 990 | CDC6 | cell division cycle 6 homolog (S. cerevisiae) |
| 890 | CCNA2 | cyclin A2 |
| 55975 | KLHL7 | kelch-like 7 (Drosophila) |
| 9493 | KIF23 | kinesin family member 23 |
| 24137 | KIF4A | kinesin family member 4A |
| 4174 | MCM5 | minichromosome maintenance complex component 5 |
| 991 | CDC20 | cell division cycle 20 homolog (S. cerevisiae) |
| 11065 | UBE2C | ubiquitin-conjugating enzyme E2C |
| 3479 | IGF1 | insulin-like growth factor 1 (somatomedin C) |
| 5933 | RBL1 | retinoblastoma-like 1 (p107) |
| 5557 | PRIM1 | primase, DNA, polypeptide 1 (49kDa) |
| 4173 | MCM4 | minichromosome maintenance complex component 4 |
| 993 | CDC25A | cell division cycle 25 homolog A (S. pombe) |
| 79733 | E2F8 | E2F transcription factor 8 |
| 5591 | PRKDC | protein kinase, DNA-activated, catalytic polypeptide |
| 11339 | OIP5 | Opa interacting protein 5 |
| 9134 | CCNE2 | cyclin E2 |
| 8487 | SIP1 | survival of motor neuron protein interacting protein 1 |
| 4001 | LMNB1 | lamin B1 |
| 5985 | RFC5 | replication factor C (activator 1) 5, 36.5kDa |
| 9585 | KIF20B | kinesin family member 20B |
| 3148 | HMGB2 | high-mobility group box 2 |
| 7133 | TNFRSF1B | tumor necrosis factor receptor superfamily, member 1B |
| 10721 | POLQ | polymerase (DNA directed), theta |

  
  
**MSCs batch 2 repressed and**GO:0007088**: 10 genes, expected 0.507325, P=5.62741e-11, P adjusted = 6.55098e-06

|  |  |  |
| --- | --- | --- |
| 9735 | KNTC1 | kinetochore associated 1 |
| 9787 | DLGAP5 | discs, large (Drosophila) homolog-associated protein 5 |
| 995 | CDC25C | cell division cycle 25 homolog C (S. pombe) |
| 332 | BIRC5 | baculoviral IAP repeat-containing 5 |
| 4751 | NEK2 | NIMA (never in mitosis gene a)-related kinase 2 |
| 51203 | NUSAP1 | nucleolar and spindle associated protein 1 |
| 54443 | ANLN | anillin, actin binding protein |
| 11065 | UBE2C | ubiquitin-conjugating enzyme E2C |
| 3479 | IGF1 | insulin-like growth factor 1 (somatomedin C) |
| 9585 | KIF20B | kinesin family member 20B |

  
  
**MSCs batch 2 repressed and**GO:0005524**: 47 genes, expected 16.519, P=6.16171e-11, P adjusted = 7.17297e-06

|  |  |  |
| --- | --- | --- |
| 9833 | MELK | maternal embryonic leucine zipper kinase |
| 983 | CDC2 | cell division cycle 2, G1 to S and G2 to M |
| 7153 | TOP2A | topoisomerase (DNA) II alpha 170kDa |
| 9212 | AURKB | aurora kinase B |
| 10112 | KIF20A | kinesin family member 20A |
| 3832 | KIF11 | kinesin family member 11 |
| 3070 | HELLS | helicase, lymphoid-specific |
| 11004 | KIF2C | kinesin family member 2C |
| 5167 | ENPP1 | ectonucleotide pyrophosphatase/phosphodiesterase 1 |
| 701 | BUB1B | BUB1 budding uninhibited by benzimidazoles 1 homolog beta (yeast) |
| 29028 | ATAD2 | ATPase family, AAA domain containing 2 |
| 1062 | CENPE | centromere protein E, 312kDa |
| 9928 | KIF14 | kinesin family member 14 |
| 4751 | NEK2 | NIMA (never in mitosis gene a)-related kinase 2 |
| 22974 | TPX2 | TPX2, microtubule-associated, homolog (Xenopus laevis) |
| 10733 | PLK4 | polo-like kinase 4 (Drosophila) |
| 27330 | RPS6KA6 | ribosomal protein S6 kinase, 90kDa, polypeptide 6 |
| 4288 | MKI67 | antigen identified by monoclonal antibody Ki-67 |
| 4176 | MCM7 | minichromosome maintenance complex component 7 |
| 146909 | KIF18B | kinesin family member 18B |
| 63979 | FIGNL1 | fidgetin-like 1 |
| 4171 | MCM2 | minichromosome maintenance complex component 2 |
| 7083 | TK1 | thymidine kinase 1, soluble |
| 7272 | TTK | TTK protein kinase |
| 2956 | MSH6 | mutS homolog 6 (E. coli) |
| 6781 | STC1 | stanniocalcin 1 |
| 6790 | AURKA | aurora kinase A |
| 990 | CDC6 | cell division cycle 6 homolog (S. cerevisiae) |
| 55872 | PBK | PDZ binding kinase |
| 9493 | KIF23 | kinesin family member 23 |
| 56992 | KIF15 | kinesin family member 15 |
| 24137 | KIF4A | kinesin family member 4A |
| 11113 | CIT | citron (rho-interacting, serine/threonine kinase 21) |
| 4174 | MCM5 | minichromosome maintenance complex component 5 |
| 10051 | SMC4 | structural maintenance of chromosomes 4 |
| 26577 | PCOLCE2 | procollagen C-endopeptidase enhancer 2 |
| 9319 | TRIP13 | thyroid hormone receptor interactor 13 |
| 4173 | MCM4 | minichromosome maintenance complex component 4 |
| 203447 | NRK | Nik related kinase |
| 5591 | PRKDC | protein kinase, DNA-activated, catalytic polypeptide |
| 699 | BUB1 | BUB1 budding uninhibited by benzimidazoles 1 homolog (yeast) |
| 54821 | ERCC6L | excision repair cross-complementing rodent repair deficiency, complementation group 6-like |
| 5985 | RFC5 | replication factor C (activator 1) 5, 36.5kDa |
| 81930 | KIF18A | kinesin family member 18A |
| 9585 | KIF20B | kinesin family member 20B |
| 6240 | RRM1 | ribonucleotide reductase M1 |
| 10721 | POLQ | polymerase (DNA directed), theta |

  
  
**MSCs batch 2 repressed and**GO:0070013**: 48 genes, expected 17.2243, P=7.55935e-11, P adjusted = 8.79999e-06

|  |  |  |
| --- | --- | --- |
| 983 | CDC2 | cell division cycle 2, G1 to S and G2 to M |
| 7153 | TOP2A | topoisomerase (DNA) II alpha 170kDa |
| 26271 | FBXO5 | F-box protein 5 |
| 10112 | KIF20A | kinesin family member 20A |
| 891 | CCNB1 | cyclin B1 |
| 51422 | PRKAG2 | protein kinase, AMP-activated, gamma 2 non-catalytic subunit |
| 995 | CDC25C | cell division cycle 25 homolog C (S. pombe) |
| 332 | BIRC5 | baculoviral IAP repeat-containing 5 |
| 10762 | NUP50 | nucleoporin 50kDa |
| 2295 | FOXF2 | forkhead box F2 |
| 5983 | RFC3 | replication factor C (activator 1) 3, 38kDa |
| 27330 | RPS6KA6 | ribosomal protein S6 kinase, 90kDa, polypeptide 6 |
| 4288 | MKI67 | antigen identified by monoclonal antibody Ki-67 |
| 10714 | POLD3 | polymerase (DNA-directed), delta 3, accessory subunit |
| 1063 | CENPF | centromere protein F, 350/400ka (mitosin) |
| 4176 | MCM7 | minichromosome maintenance complex component 7 |
| 55388 | MCM10 | minichromosome maintenance complex component 10 |
| 23649 | POLA2 | polymerase (DNA directed), alpha 2 (70kD subunit) |
| 4171 | MCM2 | minichromosome maintenance complex component 2 |
| 8318 | CDC45L | CDC45 cell division cycle 45-like (S. cerevisiae) |
| 2146 | EZH2 | enhancer of zeste homolog 2 (Drosophila) |
| 9957 | HS3ST1 | heparan sulfate (glucosamine) 3-O-sulfotransferase 1 |
| 145508 | C14orf145 | chromosome 14 open reading frame 145 |
| 55320 | C14orf106 | chromosome 14 open reading frame 106 |
| 5743 | PTGS2 | prostaglandin-endoperoxide synthase 2 (prostaglandin G/H synthase and cyclooxygenase) |
| 990 | CDC6 | cell division cycle 6 homolog (S. cerevisiae) |
| 890 | CCNA2 | cyclin A2 |
| 55975 | KLHL7 | kelch-like 7 (Drosophila) |
| 9493 | KIF23 | kinesin family member 23 |
| 24137 | KIF4A | kinesin family member 4A |
| 4174 | MCM5 | minichromosome maintenance complex component 5 |
| 991 | CDC20 | cell division cycle 20 homolog (S. cerevisiae) |
| 11065 | UBE2C | ubiquitin-conjugating enzyme E2C |
| 5933 | RBL1 | retinoblastoma-like 1 (p107) |
| 5557 | PRIM1 | primase, DNA, polypeptide 1 (49kDa) |
| 4173 | MCM4 | minichromosome maintenance complex component 4 |
| 993 | CDC25A | cell division cycle 25 homolog A (S. pombe) |
| 79733 | E2F8 | E2F transcription factor 8 |
| 5591 | PRKDC | protein kinase, DNA-activated, catalytic polypeptide |
| 11339 | OIP5 | Opa interacting protein 5 |
| 9134 | CCNE2 | cyclin E2 |
| 8487 | SIP1 | survival of motor neuron protein interacting protein 1 |
| 4001 | LMNB1 | lamin B1 |
| 5985 | RFC5 | replication factor C (activator 1) 5, 36.5kDa |
| 9585 | KIF20B | kinesin family member 20B |
| 3148 | HMGB2 | high-mobility group box 2 |
| 7133 | TNFRSF1B | tumor necrosis factor receptor superfamily, member 1B |
| 10721 | POLQ | polymerase (DNA directed), theta |

  
  
**MSCs batch 2 repressed and**GO:0032559**: 47 genes, expected 16.7417, P=9.68011e-11, P adjusted = 1.12688e-05

|  |  |  |
| --- | --- | --- |
| 9833 | MELK | maternal embryonic leucine zipper kinase |
| 983 | CDC2 | cell division cycle 2, G1 to S and G2 to M |
| 7153 | TOP2A | topoisomerase (DNA) II alpha 170kDa |
| 9212 | AURKB | aurora kinase B |
| 10112 | KIF20A | kinesin family member 20A |
| 3832 | KIF11 | kinesin family member 11 |
| 3070 | HELLS | helicase, lymphoid-specific |
| 11004 | KIF2C | kinesin family member 2C |
| 5167 | ENPP1 | ectonucleotide pyrophosphatase/phosphodiesterase 1 |
| 701 | BUB1B | BUB1 budding uninhibited by benzimidazoles 1 homolog beta (yeast) |
| 29028 | ATAD2 | ATPase family, AAA domain containing 2 |
| 1062 | CENPE | centromere protein E, 312kDa |
| 9928 | KIF14 | kinesin family member 14 |
| 4751 | NEK2 | NIMA (never in mitosis gene a)-related kinase 2 |
| 22974 | TPX2 | TPX2, microtubule-associated, homolog (Xenopus laevis) |
| 10733 | PLK4 | polo-like kinase 4 (Drosophila) |
| 27330 | RPS6KA6 | ribosomal protein S6 kinase, 90kDa, polypeptide 6 |
| 4288 | MKI67 | antigen identified by monoclonal antibody Ki-67 |
| 4176 | MCM7 | minichromosome maintenance complex component 7 |
| 146909 | KIF18B | kinesin family member 18B |
| 63979 | FIGNL1 | fidgetin-like 1 |
| 4171 | MCM2 | minichromosome maintenance complex component 2 |
| 7083 | TK1 | thymidine kinase 1, soluble |
| 7272 | TTK | TTK protein kinase |
| 2956 | MSH6 | mutS homolog 6 (E. coli) |
| 6781 | STC1 | stanniocalcin 1 |
| 6790 | AURKA | aurora kinase A |
| 990 | CDC6 | cell division cycle 6 homolog (S. cerevisiae) |
| 55872 | PBK | PDZ binding kinase |
| 9493 | KIF23 | kinesin family member 23 |
| 56992 | KIF15 | kinesin family member 15 |
| 24137 | KIF4A | kinesin family member 4A |
| 11113 | CIT | citron (rho-interacting, serine/threonine kinase 21) |
| 4174 | MCM5 | minichromosome maintenance complex component 5 |
| 10051 | SMC4 | structural maintenance of chromosomes 4 |
| 26577 | PCOLCE2 | procollagen C-endopeptidase enhancer 2 |
| 9319 | TRIP13 | thyroid hormone receptor interactor 13 |
| 4173 | MCM4 | minichromosome maintenance complex component 4 |
| 203447 | NRK | Nik related kinase |
| 5591 | PRKDC | protein kinase, DNA-activated, catalytic polypeptide |
| 699 | BUB1 | BUB1 budding uninhibited by benzimidazoles 1 homolog (yeast) |
| 54821 | ERCC6L | excision repair cross-complementing rodent repair deficiency, complementation group 6-like |
| 5985 | RFC5 | replication factor C (activator 1) 5, 36.5kDa |
| 81930 | KIF18A | kinesin family member 18A |
| 9585 | KIF20B | kinesin family member 20B |
| 6240 | RRM1 | ribonucleotide reductase M1 |
| 10721 | POLQ | polymerase (DNA directed), theta |

  
  
**MSCs batch 2 repressed and**GO:0031974**: 49 genes, expected 18.0286, P=1.09899e-10, P adjusted = 1.27936e-05

|  |  |  |
| --- | --- | --- |
| 983 | CDC2 | cell division cycle 2, G1 to S and G2 to M |
| 7153 | TOP2A | topoisomerase (DNA) II alpha 170kDa |
| 26271 | FBXO5 | F-box protein 5 |
| 10112 | KIF20A | kinesin family member 20A |
| 891 | CCNB1 | cyclin B1 |
| 51422 | PRKAG2 | protein kinase, AMP-activated, gamma 2 non-catalytic subunit |
| 995 | CDC25C | cell division cycle 25 homolog C (S. pombe) |
| 332 | BIRC5 | baculoviral IAP repeat-containing 5 |
| 10762 | NUP50 | nucleoporin 50kDa |
| 2295 | FOXF2 | forkhead box F2 |
| 5983 | RFC3 | replication factor C (activator 1) 3, 38kDa |
| 27330 | RPS6KA6 | ribosomal protein S6 kinase, 90kDa, polypeptide 6 |
| 4288 | MKI67 | antigen identified by monoclonal antibody Ki-67 |
| 10714 | POLD3 | polymerase (DNA-directed), delta 3, accessory subunit |
| 1063 | CENPF | centromere protein F, 350/400ka (mitosin) |
| 4176 | MCM7 | minichromosome maintenance complex component 7 |
| 55388 | MCM10 | minichromosome maintenance complex component 10 |
| 23649 | POLA2 | polymerase (DNA directed), alpha 2 (70kD subunit) |
| 4171 | MCM2 | minichromosome maintenance complex component 2 |
| 8318 | CDC45L | CDC45 cell division cycle 45-like (S. cerevisiae) |
| 2146 | EZH2 | enhancer of zeste homolog 2 (Drosophila) |
| 9957 | HS3ST1 | heparan sulfate (glucosamine) 3-O-sulfotransferase 1 |
| 145508 | C14orf145 | chromosome 14 open reading frame 145 |
| 55320 | C14orf106 | chromosome 14 open reading frame 106 |
| 5743 | PTGS2 | prostaglandin-endoperoxide synthase 2 (prostaglandin G/H synthase and cyclooxygenase) |
| 990 | CDC6 | cell division cycle 6 homolog (S. cerevisiae) |
| 890 | CCNA2 | cyclin A2 |
| 55975 | KLHL7 | kelch-like 7 (Drosophila) |
| 9493 | KIF23 | kinesin family member 23 |
| 24137 | KIF4A | kinesin family member 4A |
| 4174 | MCM5 | minichromosome maintenance complex component 5 |
| 991 | CDC20 | cell division cycle 20 homolog (S. cerevisiae) |
| 11065 | UBE2C | ubiquitin-conjugating enzyme E2C |
| 3479 | IGF1 | insulin-like growth factor 1 (somatomedin C) |
| 5933 | RBL1 | retinoblastoma-like 1 (p107) |
| 5557 | PRIM1 | primase, DNA, polypeptide 1 (49kDa) |
| 4173 | MCM4 | minichromosome maintenance complex component 4 |
| 993 | CDC25A | cell division cycle 25 homolog A (S. pombe) |
| 79733 | E2F8 | E2F transcription factor 8 |
| 5591 | PRKDC | protein kinase, DNA-activated, catalytic polypeptide |
| 11339 | OIP5 | Opa interacting protein 5 |
| 9134 | CCNE2 | cyclin E2 |
| 8487 | SIP1 | survival of motor neuron protein interacting protein 1 |
| 4001 | LMNB1 | lamin B1 |
| 5985 | RFC5 | replication factor C (activator 1) 5, 36.5kDa |
| 9585 | KIF20B | kinesin family member 20B |
| 3148 | HMGB2 | high-mobility group box 2 |
| 7133 | TNFRSF1B | tumor necrosis factor receptor superfamily, member 1B |
| 10721 | POLQ | polymerase (DNA directed), theta |

  
  
**MSCs batch 2 repressed and**GO:0051276**: 23 genes, expected 4.54118, P=2.10004e-10, P adjusted = 2.4447e-05

|  |  |  |
| --- | --- | --- |
| 7153 | TOP2A | topoisomerase (DNA) II alpha 170kDa |
| 9787 | DLGAP5 | discs, large (Drosophila) homolog-associated protein 5 |
| 3070 | HELLS | helicase, lymphoid-specific |
| 4085 | MAD2L1 | MAD2 mitotic arrest deficient-like 1 (yeast) |
| 113130 | CDCA5 | cell division cycle associated 5 |
| 1062 | CENPE | centromere protein E, 312kDa |
| 51203 | NUSAP1 | nucleolar and spindle associated protein 1 |
| 1063 | CENPF | centromere protein F, 350/400ka (mitosin) |
| 1058 | CENPA | centromere protein A |
| 10403 | NDC80 | NDC80 homolog, kinetochore complex component (S. cerevisiae) |
| 4171 | MCM2 | minichromosome maintenance complex component 2 |
| 23397 | NCAPH | non-SMC condensin I complex, subunit H |
| 9232 | PTTG1 | pituitary tumor-transforming 1 |
| 54892 | NCAPG2 | non-SMC condensin II complex, subunit G2 |
| 2146 | EZH2 | enhancer of zeste homolog 2 (Drosophila) |
| 11130 | ZWINT | ZW10 interactor |
| 64151 | NCAPG | non-SMC condensin I complex, subunit G |
| 10051 | SMC4 | structural maintenance of chromosomes 4 |
| 5933 | RBL1 | retinoblastoma-like 1 (p107) |
| 64946 | CENPH | centromere protein H |
| 5591 | PRKDC | protein kinase, DNA-activated, catalytic polypeptide |
| 7468 | WHSC1 | Wolf-Hirschhorn syndrome candidate 1 |
| 3148 | HMGB2 | high-mobility group box 2 |

  
  
**MSCs batch 2 repressed and**GO:0007093**: 9 genes, expected 0.420709, P=2.35856e-10, P adjusted = 2.74564e-05

|  |  |  |
| --- | --- | --- |
| 9735 | KNTC1 | kinetochore associated 1 |
| 4085 | MAD2L1 | MAD2 mitotic arrest deficient-like 1 (yeast) |
| 701 | BUB1B | BUB1 budding uninhibited by benzimidazoles 1 homolog beta (yeast) |
| 1063 | CENPF | centromere protein F, 350/400ka (mitosin) |
| 7272 | TTK | TTK protein kinase |
| 11130 | ZWINT | ZW10 interactor |
| 51512 | GTSE1 | G-2 and S-phase expressed 1 |
| 890 | CCNA2 | cyclin A2 |
| 699 | BUB1 | BUB1 budding uninhibited by benzimidazoles 1 homolog (yeast) |

  
  
**MSCs batch 2 repressed and**GO:0051716**: 22 genes, expected 4.30608, P=4.48624e-10, P adjusted = 5.22252e-05

|  |  |  |
| --- | --- | --- |
| 10635 | RAD51AP1 | RAD51 associated protein 1 |
| 7153 | TOP2A | topoisomerase (DNA) II alpha 170kDa |
| 2177 | FANCD2 | Fanconi anemia, complementation group D2 |
| 3162 | HMOX1 | heme oxygenase (decycling) 1 |
| 56852 | RAD18 | RAD18 homolog (S. cerevisiae) |
| 55215 | FANCI | Fanconi anemia, complementation group I |
| 5167 | ENPP1 | ectonucleotide pyrophosphatase/phosphodiesterase 1 |
| 5983 | RFC3 | replication factor C (activator 1) 3, 38kDa |
| 10714 | POLD3 | polymerase (DNA-directed), delta 3, accessory subunit |
| 9232 | PTTG1 | pituitary tumor-transforming 1 |
| 2956 | MSH6 | mutS homolog 6 (E. coli) |
| 7298 | TYMS | thymidylate synthetase |
| 23049 | SMG1 | PI-3-kinase-related kinase SMG-1 |
| 51512 | GTSE1 | G-2 and S-phase expressed 1 |
| 890 | CCNA2 | cyclin A2 |
| 29128 | UHRF1 | ubiquitin-like with PHD and ring finger domains 1 |
| 5591 | PRKDC | protein kinase, DNA-activated, catalytic polypeptide |
| 5985 | RFC5 | replication factor C (activator 1) 5, 36.5kDa |
| 55247 | NEIL3 | nei endonuclease VIII-like 3 (E. coli) |
| 9156 | EXO1 | exonuclease 1 |
| 3148 | HMGB2 | high-mobility group box 2 |
| 10721 | POLQ | polymerase (DNA directed), theta |

  
  
**MSCs batch 2 repressed and**GO:0034984**: 20 genes, expected 3.5389, P=4.97885e-10, P adjusted = 5.79597e-05

|  |  |  |
| --- | --- | --- |
| 10635 | RAD51AP1 | RAD51 associated protein 1 |
| 7153 | TOP2A | topoisomerase (DNA) II alpha 170kDa |
| 2177 | FANCD2 | Fanconi anemia, complementation group D2 |
| 56852 | RAD18 | RAD18 homolog (S. cerevisiae) |
| 55215 | FANCI | Fanconi anemia, complementation group I |
| 5983 | RFC3 | replication factor C (activator 1) 3, 38kDa |
| 10714 | POLD3 | polymerase (DNA-directed), delta 3, accessory subunit |
| 9232 | PTTG1 | pituitary tumor-transforming 1 |
| 2956 | MSH6 | mutS homolog 6 (E. coli) |
| 7298 | TYMS | thymidylate synthetase |
| 23049 | SMG1 | PI-3-kinase-related kinase SMG-1 |
| 51512 | GTSE1 | G-2 and S-phase expressed 1 |
| 890 | CCNA2 | cyclin A2 |
| 29128 | UHRF1 | ubiquitin-like with PHD and ring finger domains 1 |
| 5591 | PRKDC | protein kinase, DNA-activated, catalytic polypeptide |
| 5985 | RFC5 | replication factor C (activator 1) 5, 36.5kDa |
| 55247 | NEIL3 | nei endonuclease VIII-like 3 (E. coli) |
| 9156 | EXO1 | exonuclease 1 |
| 3148 | HMGB2 | high-mobility group box 2 |
| 10721 | POLQ | polymerase (DNA directed), theta |

  
  
**MSCs batch 2 repressed and**GO:0030554**: 47 genes, expected 17.6079, P=5.17068e-10, P adjusted = 6.0193e-05

|  |  |  |
| --- | --- | --- |
| 9833 | MELK | maternal embryonic leucine zipper kinase |
| 983 | CDC2 | cell division cycle 2, G1 to S and G2 to M |
| 7153 | TOP2A | topoisomerase (DNA) II alpha 170kDa |
| 9212 | AURKB | aurora kinase B |
| 10112 | KIF20A | kinesin family member 20A |
| 3832 | KIF11 | kinesin family member 11 |
| 3070 | HELLS | helicase, lymphoid-specific |
| 11004 | KIF2C | kinesin family member 2C |
| 5167 | ENPP1 | ectonucleotide pyrophosphatase/phosphodiesterase 1 |
| 701 | BUB1B | BUB1 budding uninhibited by benzimidazoles 1 homolog beta (yeast) |
| 29028 | ATAD2 | ATPase family, AAA domain containing 2 |
| 1062 | CENPE | centromere protein E, 312kDa |
| 9928 | KIF14 | kinesin family member 14 |
| 4751 | NEK2 | NIMA (never in mitosis gene a)-related kinase 2 |
| 22974 | TPX2 | TPX2, microtubule-associated, homolog (Xenopus laevis) |
| 10733 | PLK4 | polo-like kinase 4 (Drosophila) |
| 27330 | RPS6KA6 | ribosomal protein S6 kinase, 90kDa, polypeptide 6 |
| 4288 | MKI67 | antigen identified by monoclonal antibody Ki-67 |
| 4176 | MCM7 | minichromosome maintenance complex component 7 |
| 146909 | KIF18B | kinesin family member 18B |
| 63979 | FIGNL1 | fidgetin-like 1 |
| 4171 | MCM2 | minichromosome maintenance complex component 2 |
| 7083 | TK1 | thymidine kinase 1, soluble |
| 7272 | TTK | TTK protein kinase |
| 2956 | MSH6 | mutS homolog 6 (E. coli) |
| 6781 | STC1 | stanniocalcin 1 |
| 6790 | AURKA | aurora kinase A |
| 990 | CDC6 | cell division cycle 6 homolog (S. cerevisiae) |
| 55872 | PBK | PDZ binding kinase |
| 9493 | KIF23 | kinesin family member 23 |
| 56992 | KIF15 | kinesin family member 15 |
| 24137 | KIF4A | kinesin family member 4A |
| 11113 | CIT | citron (rho-interacting, serine/threonine kinase 21) |
| 4174 | MCM5 | minichromosome maintenance complex component 5 |
| 10051 | SMC4 | structural maintenance of chromosomes 4 |
| 26577 | PCOLCE2 | procollagen C-endopeptidase enhancer 2 |
| 9319 | TRIP13 | thyroid hormone receptor interactor 13 |
| 4173 | MCM4 | minichromosome maintenance complex component 4 |
| 203447 | NRK | Nik related kinase |
| 5591 | PRKDC | protein kinase, DNA-activated, catalytic polypeptide |
| 699 | BUB1 | BUB1 budding uninhibited by benzimidazoles 1 homolog (yeast) |
| 54821 | ERCC6L | excision repair cross-complementing rodent repair deficiency, complementation group 6-like |
| 5985 | RFC5 | replication factor C (activator 1) 5, 36.5kDa |
| 81930 | KIF18A | kinesin family member 18A |
| 9585 | KIF20B | kinesin family member 20B |
| 6240 | RRM1 | ribonucleotide reductase M1 |
| 10721 | POLQ | polymerase (DNA directed), theta |

  
  
**MSCs batch 2 repressed and**GO:0000922**: 8 genes, expected 0.321718, P=6.33288e-10, P adjusted = 7.37223e-05

|  |  |  |
| --- | --- | --- |
| 9735 | KNTC1 | kinetochore associated 1 |
| 9787 | DLGAP5 | discs, large (Drosophila) homolog-associated protein 5 |
| 3832 | KIF11 | kinesin family member 11 |
| 4085 | MAD2L1 | MAD2 mitotic arrest deficient-like 1 (yeast) |
| 259266 | ASPM | asp (abnormal spindle) homolog, microcephaly associated (Drosophila) |
| 22974 | TPX2 | TPX2, microtubule-associated, homolog (Xenopus laevis) |
| 1063 | CENPF | centromere protein F, 350/400ka (mitosin) |
| 699 | BUB1 | BUB1 budding uninhibited by benzimidazoles 1 homolog (yeast) |

  
  
**MSCs batch 2 repressed and**GO:0033554**: 21 genes, expected 4.1081, P=1.12459e-09, P adjusted = 0.000130915

|  |  |  |
| --- | --- | --- |
| 10635 | RAD51AP1 | RAD51 associated protein 1 |
| 7153 | TOP2A | topoisomerase (DNA) II alpha 170kDa |
| 2177 | FANCD2 | Fanconi anemia, complementation group D2 |
| 3162 | HMOX1 | heme oxygenase (decycling) 1 |
| 56852 | RAD18 | RAD18 homolog (S. cerevisiae) |
| 55215 | FANCI | Fanconi anemia, complementation group I |
| 5983 | RFC3 | replication factor C (activator 1) 3, 38kDa |
| 10714 | POLD3 | polymerase (DNA-directed), delta 3, accessory subunit |
| 9232 | PTTG1 | pituitary tumor-transforming 1 |
| 2956 | MSH6 | mutS homolog 6 (E. coli) |
| 7298 | TYMS | thymidylate synthetase |
| 23049 | SMG1 | PI-3-kinase-related kinase SMG-1 |
| 51512 | GTSE1 | G-2 and S-phase expressed 1 |
| 890 | CCNA2 | cyclin A2 |
| 29128 | UHRF1 | ubiquitin-like with PHD and ring finger domains 1 |
| 5591 | PRKDC | protein kinase, DNA-activated, catalytic polypeptide |
| 5985 | RFC5 | replication factor C (activator 1) 5, 36.5kDa |
| 55247 | NEIL3 | nei endonuclease VIII-like 3 (E. coli) |
| 9156 | EXO1 | exonuclease 1 |
| 3148 | HMGB2 | high-mobility group box 2 |
| 10721 | POLQ | polymerase (DNA directed), theta |

  
  
**MSCs batch 2 repressed and**GO:0005876**: 7 genes, expected 0.247476, P=2.75979e-09, P adjusted = 0.000321273

|  |  |  |
| --- | --- | --- |
| 9735 | KNTC1 | kinetochore associated 1 |
| 983 | CDC2 | cell division cycle 2, G1 to S and G2 to M |
| 3832 | KIF11 | kinesin family member 11 |
| 332 | BIRC5 | baculoviral IAP repeat-containing 5 |
| 10615 | SPAG5 | sperm associated antigen 5 |
| 24137 | KIF4A | kinesin family member 4A |
| 9055 | PRC1 | protein regulator of cytokinesis 1 |

  
  
**MSCs batch 2 repressed and**GO:0032555**: 50 genes, expected 20.5529, P=3.06094e-09, P adjusted = 0.000356331

|  |  |  |
| --- | --- | --- |
| 9833 | MELK | maternal embryonic leucine zipper kinase |
| 983 | CDC2 | cell division cycle 2, G1 to S and G2 to M |
| 7153 | TOP2A | topoisomerase (DNA) II alpha 170kDa |
| 9212 | AURKB | aurora kinase B |
| 10112 | KIF20A | kinesin family member 20A |
| 3832 | KIF11 | kinesin family member 11 |
| 3070 | HELLS | helicase, lymphoid-specific |
| 8654 | PDE5A | phosphodiesterase 5A, cGMP-specific |
| 11004 | KIF2C | kinesin family member 2C |
| 5167 | ENPP1 | ectonucleotide pyrophosphatase/phosphodiesterase 1 |
| 701 | BUB1B | BUB1 budding uninhibited by benzimidazoles 1 homolog beta (yeast) |
| 29028 | ATAD2 | ATPase family, AAA domain containing 2 |
| 1062 | CENPE | centromere protein E, 312kDa |
| 9928 | KIF14 | kinesin family member 14 |
| 4751 | NEK2 | NIMA (never in mitosis gene a)-related kinase 2 |
| 22974 | TPX2 | TPX2, microtubule-associated, homolog (Xenopus laevis) |
| 10733 | PLK4 | polo-like kinase 4 (Drosophila) |
| 27330 | RPS6KA6 | ribosomal protein S6 kinase, 90kDa, polypeptide 6 |
| 4288 | MKI67 | antigen identified by monoclonal antibody Ki-67 |
| 4176 | MCM7 | minichromosome maintenance complex component 7 |
| 146909 | KIF18B | kinesin family member 18B |
| 63979 | FIGNL1 | fidgetin-like 1 |
| 4171 | MCM2 | minichromosome maintenance complex component 2 |
| 7083 | TK1 | thymidine kinase 1, soluble |
| 7272 | TTK | TTK protein kinase |
| 2956 | MSH6 | mutS homolog 6 (E. coli) |
| 6781 | STC1 | stanniocalcin 1 |
| 6790 | AURKA | aurora kinase A |
| 990 | CDC6 | cell division cycle 6 homolog (S. cerevisiae) |
| 55872 | PBK | PDZ binding kinase |
| 9493 | KIF23 | kinesin family member 23 |
| 56992 | KIF15 | kinesin family member 15 |
| 24137 | KIF4A | kinesin family member 4A |
| 11113 | CIT | citron (rho-interacting, serine/threonine kinase 21) |
| 4174 | MCM5 | minichromosome maintenance complex component 5 |
| 10051 | SMC4 | structural maintenance of chromosomes 4 |
| 26577 | PCOLCE2 | procollagen C-endopeptidase enhancer 2 |
| 9319 | TRIP13 | thyroid hormone receptor interactor 13 |
| 4173 | MCM4 | minichromosome maintenance complex component 4 |
| 203447 | NRK | Nik related kinase |
| 5591 | PRKDC | protein kinase, DNA-activated, catalytic polypeptide |
| 4599 | MX1 | myxovirus (influenza virus) resistance 1, interferon-inducible protein p78 (mouse) |
| 699 | BUB1 | BUB1 budding uninhibited by benzimidazoles 1 homolog (yeast) |
| 2669 | GEM | GTP binding protein overexpressed in skeletal muscle |
| 54821 | ERCC6L | excision repair cross-complementing rodent repair deficiency, complementation group 6-like |
| 5985 | RFC5 | replication factor C (activator 1) 5, 36.5kDa |
| 81930 | KIF18A | kinesin family member 18A |
| 9585 | KIF20B | kinesin family member 20B |
| 6240 | RRM1 | ribonucleotide reductase M1 |
| 10721 | POLQ | polymerase (DNA directed), theta |

  
  
**MSCs batch 2 repressed and**GO:0032553**: 50 genes, expected 20.5529, P=3.06094e-09, P adjusted = 0.000356331

|  |  |  |
| --- | --- | --- |
| 9833 | MELK | maternal embryonic leucine zipper kinase |
| 983 | CDC2 | cell division cycle 2, G1 to S and G2 to M |
| 7153 | TOP2A | topoisomerase (DNA) II alpha 170kDa |
| 9212 | AURKB | aurora kinase B |
| 10112 | KIF20A | kinesin family member 20A |
| 3832 | KIF11 | kinesin family member 11 |
| 3070 | HELLS | helicase, lymphoid-specific |
| 8654 | PDE5A | phosphodiesterase 5A, cGMP-specific |
| 11004 | KIF2C | kinesin family member 2C |
| 5167 | ENPP1 | ectonucleotide pyrophosphatase/phosphodiesterase 1 |
| 701 | BUB1B | BUB1 budding uninhibited by benzimidazoles 1 homolog beta (yeast) |
| 29028 | ATAD2 | ATPase family, AAA domain containing 2 |
| 1062 | CENPE | centromere protein E, 312kDa |
| 9928 | KIF14 | kinesin family member 14 |
| 4751 | NEK2 | NIMA (never in mitosis gene a)-related kinase 2 |
| 22974 | TPX2 | TPX2, microtubule-associated, homolog (Xenopus laevis) |
| 10733 | PLK4 | polo-like kinase 4 (Drosophila) |
| 27330 | RPS6KA6 | ribosomal protein S6 kinase, 90kDa, polypeptide 6 |
| 4288 | MKI67 | antigen identified by monoclonal antibody Ki-67 |
| 4176 | MCM7 | minichromosome maintenance complex component 7 |
| 146909 | KIF18B | kinesin family member 18B |
| 63979 | FIGNL1 | fidgetin-like 1 |
| 4171 | MCM2 | minichromosome maintenance complex component 2 |
| 7083 | TK1 | thymidine kinase 1, soluble |
| 7272 | TTK | TTK protein kinase |
| 2956 | MSH6 | mutS homolog 6 (E. coli) |
| 6781 | STC1 | stanniocalcin 1 |
| 6790 | AURKA | aurora kinase A |
| 990 | CDC6 | cell division cycle 6 homolog (S. cerevisiae) |
| 55872 | PBK | PDZ binding kinase |
| 9493 | KIF23 | kinesin family member 23 |
| 56992 | KIF15 | kinesin family member 15 |
| 24137 | KIF4A | kinesin family member 4A |
| 11113 | CIT | citron (rho-interacting, serine/threonine kinase 21) |
| 4174 | MCM5 | minichromosome maintenance complex component 5 |
| 10051 | SMC4 | structural maintenance of chromosomes 4 |
| 26577 | PCOLCE2 | procollagen C-endopeptidase enhancer 2 |
| 9319 | TRIP13 | thyroid hormone receptor interactor 13 |
| 4173 | MCM4 | minichromosome maintenance complex component 4 |
| 203447 | NRK | Nik related kinase |
| 5591 | PRKDC | protein kinase, DNA-activated, catalytic polypeptide |
| 4599 | MX1 | myxovirus (influenza virus) resistance 1, interferon-inducible protein p78 (mouse) |
| 699 | BUB1 | BUB1 budding uninhibited by benzimidazoles 1 homolog (yeast) |
| 2669 | GEM | GTP binding protein overexpressed in skeletal muscle |
| 54821 | ERCC6L | excision repair cross-complementing rodent repair deficiency, complementation group 6-like |
| 5985 | RFC5 | replication factor C (activator 1) 5, 36.5kDa |
| 81930 | KIF18A | kinesin family member 18A |
| 9585 | KIF20B | kinesin family member 20B |
| 6240 | RRM1 | ribonucleotide reductase M1 |
| 10721 | POLQ | polymerase (DNA directed), theta |

  
  
**MSCs batch 2 repressed and**GO:0006281**: 18 genes, expected 3.16769, P=3.45937e-09, P adjusted = 0.000402712

|  |  |  |
| --- | --- | --- |
| 10635 | RAD51AP1 | RAD51 associated protein 1 |
| 7153 | TOP2A | topoisomerase (DNA) II alpha 170kDa |
| 2177 | FANCD2 | Fanconi anemia, complementation group D2 |
| 56852 | RAD18 | RAD18 homolog (S. cerevisiae) |
| 55215 | FANCI | Fanconi anemia, complementation group I |
| 5983 | RFC3 | replication factor C (activator 1) 3, 38kDa |
| 10714 | POLD3 | polymerase (DNA-directed), delta 3, accessory subunit |
| 9232 | PTTG1 | pituitary tumor-transforming 1 |
| 2956 | MSH6 | mutS homolog 6 (E. coli) |
| 7298 | TYMS | thymidylate synthetase |
| 23049 | SMG1 | PI-3-kinase-related kinase SMG-1 |
| 29128 | UHRF1 | ubiquitin-like with PHD and ring finger domains 1 |
| 5591 | PRKDC | protein kinase, DNA-activated, catalytic polypeptide |
| 5985 | RFC5 | replication factor C (activator 1) 5, 36.5kDa |
| 55247 | NEIL3 | nei endonuclease VIII-like 3 (E. coli) |
| 9156 | EXO1 | exonuclease 1 |
| 3148 | HMGB2 | high-mobility group box 2 |
| 10721 | POLQ | polymerase (DNA directed), theta |

  
  
**MSCs batch 2 repressed and**GO:0003777**: 11 genes, expected 0.977529, P=3.59778e-09, P adjusted = 0.000418825

|  |  |  |
| --- | --- | --- |
| 10112 | KIF20A | kinesin family member 20A |
| 3832 | KIF11 | kinesin family member 11 |
| 11004 | KIF2C | kinesin family member 2C |
| 1062 | CENPE | centromere protein E, 312kDa |
| 9928 | KIF14 | kinesin family member 14 |
| 146909 | KIF18B | kinesin family member 18B |
| 9493 | KIF23 | kinesin family member 23 |
| 56992 | KIF15 | kinesin family member 15 |
| 24137 | KIF4A | kinesin family member 4A |
| 81930 | KIF18A | kinesin family member 18A |
| 9585 | KIF20B | kinesin family member 20B |

  
  
**MSCs batch 2 repressed and**GO:0007018**: 12 genes, expected 1.23738, P=3.96896e-09, P adjusted = 0.000462035

|  |  |  |
| --- | --- | --- |
| 10112 | KIF20A | kinesin family member 20A |
| 3832 | KIF11 | kinesin family member 11 |
| 11004 | KIF2C | kinesin family member 2C |
| 1062 | CENPE | centromere protein E, 312kDa |
| 9928 | KIF14 | kinesin family member 14 |
| 146909 | KIF18B | kinesin family member 18B |
| 9493 | KIF23 | kinesin family member 23 |
| 56992 | KIF15 | kinesin family member 15 |
| 24137 | KIF4A | kinesin family member 4A |
| 4741 | NEFM | neurofilament, medium polypeptide |
| 81930 | KIF18A | kinesin family member 18A |
| 9585 | KIF20B | kinesin family member 20B |

  
  
**MSCs batch 2 repressed and**GO:0031577**: 5 genes, expected 0.0866165, P=5.73651e-09, P adjusted = 0.000667798

|  |  |  |
| --- | --- | --- |
| 4085 | MAD2L1 | MAD2 mitotic arrest deficient-like 1 (yeast) |
| 332 | BIRC5 | baculoviral IAP repeat-containing 5 |
| 1063 | CENPF | centromere protein F, 350/400ka (mitosin) |
| 7272 | TTK | TTK protein kinase |
| 699 | BUB1 | BUB1 budding uninhibited by benzimidazoles 1 homolog (yeast) |

  
  
**MSCs batch 2 repressed and**GO:0030261**: 7 genes, expected 0.284597, P=8.45557e-09, P adjusted = 0.000984329

|  |  |  |
| --- | --- | --- |
| 7153 | TOP2A | topoisomerase (DNA) II alpha 170kDa |
| 113130 | CDCA5 | cell division cycle associated 5 |
| 51203 | NUSAP1 | nucleolar and spindle associated protein 1 |
| 23397 | NCAPH | non-SMC condensin I complex, subunit H |
| 54892 | NCAPG2 | non-SMC condensin II complex, subunit G2 |
| 64151 | NCAPG | non-SMC condensin I complex, subunit G |
| 10051 | SMC4 | structural maintenance of chromosomes 4 |

  
  
**MSCs batch 2 repressed and**GO:0006270**: 7 genes, expected 0.284597, P=8.45557e-09, P adjusted = 0.000984329

|  |  |  |
| --- | --- | --- |
| 4176 | MCM7 | minichromosome maintenance complex component 7 |
| 4171 | MCM2 | minichromosome maintenance complex component 2 |
| 8318 | CDC45L | CDC45 cell division cycle 45-like (S. cerevisiae) |
| 990 | CDC6 | cell division cycle 6 homolog (S. cerevisiae) |
| 4174 | MCM5 | minichromosome maintenance complex component 5 |
| 4173 | MCM4 | minichromosome maintenance complex component 4 |
| 9134 | CCNE2 | cyclin E2 |

  
  
**MSCs batch 2 repressed and**GO:0017076**: 50 genes, expected 21.4438, P=1.2359e-08, P adjusted = 0.00143873

|  |  |  |
| --- | --- | --- |
| 9833 | MELK | maternal embryonic leucine zipper kinase |
| 983 | CDC2 | cell division cycle 2, G1 to S and G2 to M |
| 7153 | TOP2A | topoisomerase (DNA) II alpha 170kDa |
| 9212 | AURKB | aurora kinase B |
| 10112 | KIF20A | kinesin family member 20A |
| 3832 | KIF11 | kinesin family member 11 |
| 3070 | HELLS | helicase, lymphoid-specific |
| 8654 | PDE5A | phosphodiesterase 5A, cGMP-specific |
| 11004 | KIF2C | kinesin family member 2C |
| 5167 | ENPP1 | ectonucleotide pyrophosphatase/phosphodiesterase 1 |
| 701 | BUB1B | BUB1 budding uninhibited by benzimidazoles 1 homolog beta (yeast) |
| 29028 | ATAD2 | ATPase family, AAA domain containing 2 |
| 1062 | CENPE | centromere protein E, 312kDa |
| 9928 | KIF14 | kinesin family member 14 |
| 4751 | NEK2 | NIMA (never in mitosis gene a)-related kinase 2 |
| 22974 | TPX2 | TPX2, microtubule-associated, homolog (Xenopus laevis) |
| 10733 | PLK4 | polo-like kinase 4 (Drosophila) |
| 27330 | RPS6KA6 | ribosomal protein S6 kinase, 90kDa, polypeptide 6 |
| 4288 | MKI67 | antigen identified by monoclonal antibody Ki-67 |
| 4176 | MCM7 | minichromosome maintenance complex component 7 |
| 146909 | KIF18B | kinesin family member 18B |
| 63979 | FIGNL1 | fidgetin-like 1 |
| 4171 | MCM2 | minichromosome maintenance complex component 2 |
| 7083 | TK1 | thymidine kinase 1, soluble |
| 7272 | TTK | TTK protein kinase |
| 2956 | MSH6 | mutS homolog 6 (E. coli) |
| 6781 | STC1 | stanniocalcin 1 |
| 6790 | AURKA | aurora kinase A |
| 990 | CDC6 | cell division cycle 6 homolog (S. cerevisiae) |
| 55872 | PBK | PDZ binding kinase |
| 9493 | KIF23 | kinesin family member 23 |
| 56992 | KIF15 | kinesin family member 15 |
| 24137 | KIF4A | kinesin family member 4A |
| 11113 | CIT | citron (rho-interacting, serine/threonine kinase 21) |
| 4174 | MCM5 | minichromosome maintenance complex component 5 |
| 10051 | SMC4 | structural maintenance of chromosomes 4 |
| 26577 | PCOLCE2 | procollagen C-endopeptidase enhancer 2 |
| 9319 | TRIP13 | thyroid hormone receptor interactor 13 |
| 4173 | MCM4 | minichromosome maintenance complex component 4 |
| 203447 | NRK | Nik related kinase |
| 5591 | PRKDC | protein kinase, DNA-activated, catalytic polypeptide |
| 4599 | MX1 | myxovirus (influenza virus) resistance 1, interferon-inducible protein p78 (mouse) |
| 699 | BUB1 | BUB1 budding uninhibited by benzimidazoles 1 homolog (yeast) |
| 2669 | GEM | GTP binding protein overexpressed in skeletal muscle |
| 54821 | ERCC6L | excision repair cross-complementing rodent repair deficiency, complementation group 6-like |
| 5985 | RFC5 | replication factor C (activator 1) 5, 36.5kDa |
| 81930 | KIF18A | kinesin family member 18A |
| 9585 | KIF20B | kinesin family member 20B |
| 6240 | RRM1 | ribonucleotide reductase M1 |
| 10721 | POLQ | polymerase (DNA directed), theta |

  
  
**MSCs batch 2 repressed and**GO:0051303**: 5 genes, expected 0.0989903, P=1.51428e-08, P adjusted = 0.0017628

|  |  |  |
| --- | --- | --- |
| 9787 | DLGAP5 | discs, large (Drosophila) homolog-associated protein 5 |
| 332 | BIRC5 | baculoviral IAP repeat-containing 5 |
| 113130 | CDCA5 | cell division cycle associated 5 |
| 1062 | CENPE | centromere protein E, 312kDa |
| 1063 | CENPF | centromere protein F, 350/400ka (mitosin) |

  
  
**MSCs batch 2 repressed and**GO:0050000**: 5 genes, expected 0.0989903, P=1.51428e-08, P adjusted = 0.0017628

|  |  |  |
| --- | --- | --- |
| 9787 | DLGAP5 | discs, large (Drosophila) homolog-associated protein 5 |
| 332 | BIRC5 | baculoviral IAP repeat-containing 5 |
| 113130 | CDCA5 | cell division cycle associated 5 |
| 1062 | CENPE | centromere protein E, 312kDa |
| 1063 | CENPF | centromere protein F, 350/400ka (mitosin) |

  
  
**MSCs batch 2 repressed and**GO:0006996**: 32 genes, expected 10.4064, P=1.68919e-08, P adjusted = 0.00196641

|  |  |  |
| --- | --- | --- |
| 7153 | TOP2A | topoisomerase (DNA) II alpha 170kDa |
| 9787 | DLGAP5 | discs, large (Drosophila) homolog-associated protein 5 |
| 26271 | FBXO5 | F-box protein 5 |
| 3832 | KIF11 | kinesin family member 11 |
| 3070 | HELLS | helicase, lymphoid-specific |
| 4085 | MAD2L1 | MAD2 mitotic arrest deficient-like 1 (yeast) |
| 11004 | KIF2C | kinesin family member 2C |
| 113130 | CDCA5 | cell division cycle associated 5 |
| 11013 | TMSL8 | thymosin-like 8 |
| 1062 | CENPE | centromere protein E, 312kDa |
| 51203 | NUSAP1 | nucleolar and spindle associated protein 1 |
| 1063 | CENPF | centromere protein F, 350/400ka (mitosin) |
| 23136 | EPB41L3 | erythrocyte membrane protein band 4.1-like 3 |
| 2537 | IFI6 | interferon, alpha-inducible protein 6 |
| 1058 | CENPA | centromere protein A |
| 10403 | NDC80 | NDC80 homolog, kinetochore complex component (S. cerevisiae) |
| 822 | CAPG | capping protein (actin filament), gelsolin-like |
| 4171 | MCM2 | minichromosome maintenance complex component 2 |
| 23397 | NCAPH | non-SMC condensin I complex, subunit H |
| 9232 | PTTG1 | pituitary tumor-transforming 1 |
| 54892 | NCAPG2 | non-SMC condensin II complex, subunit G2 |
| 2146 | EZH2 | enhancer of zeste homolog 2 (Drosophila) |
| 11130 | ZWINT | ZW10 interactor |
| 64151 | NCAPG | non-SMC condensin I complex, subunit G |
| 54443 | ANLN | anillin, actin binding protein |
| 24137 | KIF4A | kinesin family member 4A |
| 10051 | SMC4 | structural maintenance of chromosomes 4 |
| 5933 | RBL1 | retinoblastoma-like 1 (p107) |
| 64946 | CENPH | centromere protein H |
| 5591 | PRKDC | protein kinase, DNA-activated, catalytic polypeptide |
| 7468 | WHSC1 | Wolf-Hirschhorn syndrome candidate 1 |
| 3148 | HMGB2 | high-mobility group box 2 |

  
  
**MSCs batch 2 repressed and**GO:0005875**: 12 genes, expected 1.41061, P=1.79116e-08, P adjusted = 0.00208512

|  |  |  |
| --- | --- | --- |
| 10112 | KIF20A | kinesin family member 20A |
| 3832 | KIF11 | kinesin family member 11 |
| 11004 | KIF2C | kinesin family member 2C |
| 1062 | CENPE | centromere protein E, 312kDa |
| 9928 | KIF14 | kinesin family member 14 |
| 146909 | KIF18B | kinesin family member 18B |
| 9493 | KIF23 | kinesin family member 23 |
| 56992 | KIF15 | kinesin family member 15 |
| 24137 | KIF4A | kinesin family member 4A |
| 6780 | STAU1 | staufen, RNA binding protein, homolog 1 (Drosophila) |
| 81930 | KIF18A | kinesin family member 18A |
| 9585 | KIF20B | kinesin family member 20B |

  
  
**MSCs batch 2 repressed and**GO:0006323**: 11 genes, expected 1.16314, P=2.33482e-08, P adjusted = 0.00271801

|  |  |  |
| --- | --- | --- |
| 7153 | TOP2A | topoisomerase (DNA) II alpha 170kDa |
| 3070 | HELLS | helicase, lymphoid-specific |
| 113130 | CDCA5 | cell division cycle associated 5 |
| 51203 | NUSAP1 | nucleolar and spindle associated protein 1 |
| 1058 | CENPA | centromere protein A |
| 4171 | MCM2 | minichromosome maintenance complex component 2 |
| 23397 | NCAPH | non-SMC condensin I complex, subunit H |
| 54892 | NCAPG2 | non-SMC condensin II complex, subunit G2 |
| 64151 | NCAPG | non-SMC condensin I complex, subunit G |
| 10051 | SMC4 | structural maintenance of chromosomes 4 |
| 3148 | HMGB2 | high-mobility group box 2 |

  
  
**MSCs batch 2 repressed and**GO:0030705**: 12 genes, expected 1.52198, P=4.22839e-08, P adjusted = 0.00492235

|  |  |  |
| --- | --- | --- |
| 10112 | KIF20A | kinesin family member 20A |
| 3832 | KIF11 | kinesin family member 11 |
| 11004 | KIF2C | kinesin family member 2C |
| 1062 | CENPE | centromere protein E, 312kDa |
| 9928 | KIF14 | kinesin family member 14 |
| 146909 | KIF18B | kinesin family member 18B |
| 9493 | KIF23 | kinesin family member 23 |
| 56992 | KIF15 | kinesin family member 15 |
| 24137 | KIF4A | kinesin family member 4A |
| 4741 | NEFM | neurofilament, medium polypeptide |
| 81930 | KIF18A | kinesin family member 18A |
| 9585 | KIF20B | kinesin family member 20B |

  
  
**MSCs batch 2 repressed and**GO:0007076**: 6 genes, expected 0.222728, P=5.54073e-08, P adjusted = 0.00645008

|  |  |  |
| --- | --- | --- |
| 113130 | CDCA5 | cell division cycle associated 5 |
| 51203 | NUSAP1 | nucleolar and spindle associated protein 1 |
| 23397 | NCAPH | non-SMC condensin I complex, subunit H |
| 54892 | NCAPG2 | non-SMC condensin II complex, subunit G2 |
| 64151 | NCAPG | non-SMC condensin I complex, subunit G |
| 10051 | SMC4 | structural maintenance of chromosomes 4 |

  
  
**MSCs batch 2 repressed and**GO:0000166**: 52 genes, expected 24.5743, P=1.41753e-07, P adjusted = 0.0165017

|  |  |  |
| --- | --- | --- |
| 9833 | MELK | maternal embryonic leucine zipper kinase |
| 983 | CDC2 | cell division cycle 2, G1 to S and G2 to M |
| 7153 | TOP2A | topoisomerase (DNA) II alpha 170kDa |
| 9212 | AURKB | aurora kinase B |
| 10112 | KIF20A | kinesin family member 20A |
| 3832 | KIF11 | kinesin family member 11 |
| 3070 | HELLS | helicase, lymphoid-specific |
| 8654 | PDE5A | phosphodiesterase 5A, cGMP-specific |
| 11004 | KIF2C | kinesin family member 2C |
| 5167 | ENPP1 | ectonucleotide pyrophosphatase/phosphodiesterase 1 |
| 701 | BUB1B | BUB1 budding uninhibited by benzimidazoles 1 homolog beta (yeast) |
| 29028 | ATAD2 | ATPase family, AAA domain containing 2 |
| 1062 | CENPE | centromere protein E, 312kDa |
| 9928 | KIF14 | kinesin family member 14 |
| 5983 | RFC3 | replication factor C (activator 1) 3, 38kDa |
| 4751 | NEK2 | NIMA (never in mitosis gene a)-related kinase 2 |
| 22974 | TPX2 | TPX2, microtubule-associated, homolog (Xenopus laevis) |
| 10733 | PLK4 | polo-like kinase 4 (Drosophila) |
| 27330 | RPS6KA6 | ribosomal protein S6 kinase, 90kDa, polypeptide 6 |
| 4288 | MKI67 | antigen identified by monoclonal antibody Ki-67 |
| 4176 | MCM7 | minichromosome maintenance complex component 7 |
| 146909 | KIF18B | kinesin family member 18B |
| 63979 | FIGNL1 | fidgetin-like 1 |
| 4171 | MCM2 | minichromosome maintenance complex component 2 |
| 89795 | NAV3 | neuron navigator 3 |
| 7083 | TK1 | thymidine kinase 1, soluble |
| 7272 | TTK | TTK protein kinase |
| 2956 | MSH6 | mutS homolog 6 (E. coli) |
| 6781 | STC1 | stanniocalcin 1 |
| 6790 | AURKA | aurora kinase A |
| 990 | CDC6 | cell division cycle 6 homolog (S. cerevisiae) |
| 55872 | PBK | PDZ binding kinase |
| 9493 | KIF23 | kinesin family member 23 |
| 56992 | KIF15 | kinesin family member 15 |
| 24137 | KIF4A | kinesin family member 4A |
| 11113 | CIT | citron (rho-interacting, serine/threonine kinase 21) |
| 4174 | MCM5 | minichromosome maintenance complex component 5 |
| 10051 | SMC4 | structural maintenance of chromosomes 4 |
| 26577 | PCOLCE2 | procollagen C-endopeptidase enhancer 2 |
| 9319 | TRIP13 | thyroid hormone receptor interactor 13 |
| 4173 | MCM4 | minichromosome maintenance complex component 4 |
| 203447 | NRK | Nik related kinase |
| 5591 | PRKDC | protein kinase, DNA-activated, catalytic polypeptide |
| 4599 | MX1 | myxovirus (influenza virus) resistance 1, interferon-inducible protein p78 (mouse) |
| 699 | BUB1 | BUB1 budding uninhibited by benzimidazoles 1 homolog (yeast) |
| 2669 | GEM | GTP binding protein overexpressed in skeletal muscle |
| 54821 | ERCC6L | excision repair cross-complementing rodent repair deficiency, complementation group 6-like |
| 5985 | RFC5 | replication factor C (activator 1) 5, 36.5kDa |
| 81930 | KIF18A | kinesin family member 18A |
| 9585 | KIF20B | kinesin family member 20B |
| 6240 | RRM1 | ribonucleotide reductase M1 |
| 10721 | POLQ | polymerase (DNA directed), theta |

  
  
**MSCs batch 2 repressed and**GO:0007052**: 5 genes, expected 0.148485, P=2.05644e-07, P adjusted = 0.0239394

|  |  |  |
| --- | --- | --- |
| 3832 | KIF11 | kinesin family member 11 |
| 3925 | STMN1 | stathmin 1/oncoprotein 18 |
| 7272 | TTK | TTK protein kinase |
| 9493 | KIF23 | kinesin family member 23 |
| 9055 | PRC1 | protein regulator of cytokinesis 1 |

  
  
**MSCs batch 2 repressed and**GO:0007094**: 4 genes, expected 0.0742427, P=3.3671e-07, P adjusted = 0.0391971

|  |  |  |
| --- | --- | --- |
| 4085 | MAD2L1 | MAD2 mitotic arrest deficient-like 1 (yeast) |
| 1063 | CENPF | centromere protein F, 350/400ka (mitosin) |
| 7272 | TTK | TTK protein kinase |
| 699 | BUB1 | BUB1 budding uninhibited by benzimidazoles 1 homolog (yeast) |

  
  
**MSCs batch 2 repressed and**GO:0048015**: 10 genes, expected 1.23738, P=4.58644e-07, P adjusted = 0.0533916

|  |  |  |
| --- | --- | --- |
| 7153 | TOP2A | topoisomerase (DNA) II alpha 170kDa |
| 701 | BUB1B | BUB1 budding uninhibited by benzimidazoles 1 homolog beta (yeast) |
| 10403 | NDC80 | NDC80 homolog, kinetochore complex component (S. cerevisiae) |
| 11130 | ZWINT | ZW10 interactor |
| 7298 | TYMS | thymidylate synthetase |
| 6790 | AURKA | aurora kinase A |
| 10615 | SPAG5 | sperm associated antigen 5 |
| 11065 | UBE2C | ubiquitin-conjugating enzyme E2C |
| 3479 | IGF1 | insulin-like growth factor 1 (somatomedin C) |
| 3148 | HMGB2 | high-mobility group box 2 |

  
  
**MSCs batch 2 repressed and**GO:0045840**: 5 genes, expected 0.185607, P=7.56373e-07, P adjusted = 0.0880509

|  |  |  |
| --- | --- | --- |
| 9787 | DLGAP5 | discs, large (Drosophila) homolog-associated protein 5 |
| 332 | BIRC5 | baculoviral IAP repeat-containing 5 |
| 51203 | NUSAP1 | nucleolar and spindle associated protein 1 |
| 11065 | UBE2C | ubiquitin-conjugating enzyme E2C |
| 3479 | IGF1 | insulin-like growth factor 1 (somatomedin C) |

  
  
**MSCs batch 2 repressed and**GO:0003774**: 11 genes, expected 1.7447, P=1.4923e-06, P adjusted = 0.173722

|  |  |  |
| --- | --- | --- |
| 10112 | KIF20A | kinesin family member 20A |
| 3832 | KIF11 | kinesin family member 11 |
| 11004 | KIF2C | kinesin family member 2C |
| 1062 | CENPE | centromere protein E, 312kDa |
| 9928 | KIF14 | kinesin family member 14 |
| 146909 | KIF18B | kinesin family member 18B |
| 9493 | KIF23 | kinesin family member 23 |
| 56992 | KIF15 | kinesin family member 15 |
| 24137 | KIF4A | kinesin family member 4A |
| 81930 | KIF18A | kinesin family member 18A |
| 9585 | KIF20B | kinesin family member 20B |

  
  
**MSCs batch 2 repressed and**GO:0005657**: 6 genes, expected 0.383587, P=1.92028e-06, P adjusted = 0.223543

|  |  |  |
| --- | --- | --- |
| 5983 | RFC3 | replication factor C (activator 1) 3, 38kDa |
| 10714 | POLD3 | polymerase (DNA-directed), delta 3, accessory subunit |
| 4176 | MCM7 | minichromosome maintenance complex component 7 |
| 23649 | POLA2 | polymerase (DNA directed), alpha 2 (70kD subunit) |
| 5557 | PRIM1 | primase, DNA, polypeptide 1 (49kDa) |
| 5985 | RFC5 | replication factor C (activator 1) 5, 36.5kDa |

  
  
**MSCs batch 2 repressed and**GO:0051325**: 9 genes, expected 1.16314, P=2.50849e-06, P adjusted = 0.292018

|  |  |  |
| --- | --- | --- |
| 891 | CCNB1 | cyclin B1 |
| 995 | CDC25C | cell division cycle 25 homolog C (S. pombe) |
| 332 | BIRC5 | baculoviral IAP repeat-containing 5 |
| 113130 | CDCA5 | cell division cycle associated 5 |
| 1063 | CENPF | centromere protein F, 350/400ka (mitosin) |
| 51512 | GTSE1 | G-2 and S-phase expressed 1 |
| 990 | CDC6 | cell division cycle 6 homolog (S. cerevisiae) |
| 1033 | CDKN3 | cyclin-dependent kinase inhibitor 3 |
| 8914 | TIMELESS | timeless homolog (Drosophila) |

  
  
**MSCs batch 2 repressed and**GO:0008283**: 27 genes, expected 9.9609, P=2.73342e-06, P adjusted = 0.318203

|  |  |  |
| --- | --- | --- |
| 9787 | DLGAP5 | discs, large (Drosophila) homolog-associated protein 5 |
| 995 | CDC25C | cell division cycle 25 homolog C (S. pombe) |
| 3070 | HELLS | helicase, lymphoid-specific |
| 3162 | HMOX1 | heme oxygenase (decycling) 1 |
| 11004 | KIF2C | kinesin family member 2C |
| 701 | BUB1B | BUB1 budding uninhibited by benzimidazoles 1 homolog beta (yeast) |
| 2250 | FGF5 | fibroblast growth factor 5 |
| 29127 | RACGAP1 | Rac GTPase activating protein 1 |
| 259266 | ASPM | asp (abnormal spindle) homolog, microcephaly associated (Drosophila) |
| 22974 | TPX2 | TPX2, microtubule-associated, homolog (Xenopus laevis) |
| 646 | BNC1 | basonuclin 1 |
| 4288 | MKI67 | antigen identified by monoclonal antibody Ki-67 |
| 1063 | CENPF | centromere protein F, 350/400ka (mitosin) |
| 8519 | IFITM1 | interferon induced transmembrane protein 1 (9-27) |
| 6941 | TCF19 | transcription factor 19 (SC1) |
| 54892 | NCAPG2 | non-SMC condensin II complex, subunit G2 |
| 83879 | CDCA7 | cell division cycle associated 7 |
| 7272 | TTK | TTK protein kinase |
| 990 | CDC6 | cell division cycle 6 homolog (S. cerevisiae) |
| 5918 | RARRES1 | retinoic acid receptor responder (tazarotene induced) 1 |
| 56992 | KIF15 | kinesin family member 15 |
| 1033 | CDKN3 | cyclin-dependent kinase inhibitor 3 |
| 3479 | IGF1 | insulin-like growth factor 1 (somatomedin C) |
| 29128 | UHRF1 | ubiquitin-like with PHD and ring finger domains 1 |
| 993 | CDC25A | cell division cycle 25 homolog A (S. pombe) |
| 699 | BUB1 | BUB1 budding uninhibited by benzimidazoles 1 homolog (yeast) |
| 8914 | TIMELESS | timeless homolog (Drosophila) |

  
  
**MSCs batch 2 repressed and**GO:0000785**: 11 genes, expected 1.93031, P=4.01098e-06, P adjusted = 0.466926

|  |  |  |
| --- | --- | --- |
| 3070 | HELLS | helicase, lymphoid-specific |
| 56852 | RAD18 | RAD18 homolog (S. cerevisiae) |
| 1063 | CENPF | centromere protein F, 350/400ka (mitosin) |
| 4176 | MCM7 | minichromosome maintenance complex component 7 |
| 7112 | TMPO | thymopoietin |
| 1058 | CENPA | centromere protein A |
| 4171 | MCM2 | minichromosome maintenance complex component 2 |
| 2956 | MSH6 | mutS homolog 6 (E. coli) |
| 11339 | OIP5 | Opa interacting protein 5 |
| 8914 | TIMELESS | timeless homolog (Drosophila) |
| 3148 | HMGB2 | high-mobility group box 2 |

  
  
**MSCs batch 2 repressed and**GO:0006950**: 35 genes, expected 15.4796, P=5.20398e-06, P adjusted = 0.605806

|  |  |  |
| --- | --- | --- |
| 10635 | RAD51AP1 | RAD51 associated protein 1 |
| 7153 | TOP2A | topoisomerase (DNA) II alpha 170kDa |
| 2177 | FANCD2 | Fanconi anemia, complementation group D2 |
| 3162 | HMOX1 | heme oxygenase (decycling) 1 |
| 51514 | DTL | denticleless homolog (Drosophila) |
| 5742 | PTGS1 | prostaglandin-endoperoxide synthase 1 (prostaglandin G/H synthase and cyclooxygenase) |
| 56852 | RAD18 | RAD18 homolog (S. cerevisiae) |
| 55215 | FANCI | Fanconi anemia, complementation group I |
| 5983 | RFC3 | replication factor C (activator 1) 3, 38kDa |
| 10714 | POLD3 | polymerase (DNA-directed), delta 3, accessory subunit |
| 4176 | MCM7 | minichromosome maintenance complex component 7 |
| 7056 | THBD | thrombomodulin |
| 154 | ADRB2 | adrenergic, beta-2-, receptor, surface |
| 9232 | PTTG1 | pituitary tumor-transforming 1 |
| 2956 | MSH6 | mutS homolog 6 (E. coli) |
| 7298 | TYMS | thymidylate synthetase |
| 23049 | SMG1 | PI-3-kinase-related kinase SMG-1 |
| 5743 | PTGS2 | prostaglandin-endoperoxide synthase 2 (prostaglandin G/H synthase and cyclooxygenase) |
| 51512 | GTSE1 | G-2 and S-phase expressed 1 |
| 10460 | TACC3 | transforming, acidic coiled-coil containing protein 3 |
| 890 | CCNA2 | cyclin A2 |
| 7980 | TFPI2 | tissue factor pathway inhibitor 2 |
| 7716 | VEZF1 | vascular endothelial zinc finger 1 |
| 3479 | IGF1 | insulin-like growth factor 1 (somatomedin C) |
| 29128 | UHRF1 | ubiquitin-like with PHD and ring finger domains 1 |
| 5591 | PRKDC | protein kinase, DNA-activated, catalytic polypeptide |
| 4599 | MX1 | myxovirus (influenza virus) resistance 1, interferon-inducible protein p78 (mouse) |
| 136 | ADORA2B | adenosine A2b receptor |
| 8914 | TIMELESS | timeless homolog (Drosophila) |
| 5985 | RFC5 | replication factor C (activator 1) 5, 36.5kDa |
| 55247 | NEIL3 | nei endonuclease VIII-like 3 (E. coli) |
| 9156 | EXO1 | exonuclease 1 |
| 3148 | HMGB2 | high-mobility group box 2 |
| 7133 | TNFRSF1B | tumor necrosis factor receptor superfamily, member 1B |
| 10721 | POLQ | polymerase (DNA directed), theta |

  
  
**MSCs batch 2 repressed and**GO:0007096**: 4 genes, expected 0.136112, P=7.05426e-06, P adjusted = 0.821201

|  |  |  |
| --- | --- | --- |
| 9735 | KNTC1 | kinetochore associated 1 |
| 332 | BIRC5 | baculoviral IAP repeat-containing 5 |
| 54443 | ANLN | anillin, actin binding protein |
| 11065 | UBE2C | ubiquitin-conjugating enzyme E2C |

  
  
**MSCs batch 2 repressed and**GO:0000940**: 3 genes, expected 0.0494951, P=7.41997e-06, P adjusted = 0.863774

|  |  |  |
| --- | --- | --- |
| 701 | BUB1B | BUB1 budding uninhibited by benzimidazoles 1 homolog beta (yeast) |
| 1062 | CENPE | centromere protein E, 312kDa |
| 1063 | CENPF | centromere protein F, 350/400ka (mitosin) |

  
  
**MSCs batch 2 repressed and**GO:0034508**: 3 genes, expected 0.0494951, P=7.41997e-06, P adjusted = 0.863774

|  |  |  |
| --- | --- | --- |
| 3070 | HELLS | helicase, lymphoid-specific |
| 1062 | CENPE | centromere protein E, 312kDa |
| 1063 | CENPF | centromere protein F, 350/400ka (mitosin) |

  
  
**MSCs batch 2 repressed and**GO:0051383**: 3 genes, expected 0.0494951, P=7.41997e-06, P adjusted = 0.863774

|  |  |  |
| --- | --- | --- |
| 1062 | CENPE | centromere protein E, 312kDa |
| 1063 | CENPF | centromere protein F, 350/400ka (mitosin) |
| 64946 | CENPH | centromere protein H |

  
  
**MSCs batch 2 repressed and**GO:0005815**: 12 genes, expected 2.52425, P=9.48666e-06, P adjusted = 1

|  |  |  |
| --- | --- | --- |
| 7153 | TOP2A | topoisomerase (DNA) II alpha 170kDa |
| 9787 | DLGAP5 | discs, large (Drosophila) homolog-associated protein 5 |
| 891 | CCNB1 | cyclin B1 |
| 4085 | MAD2L1 | MAD2 mitotic arrest deficient-like 1 (yeast) |
| 332 | BIRC5 | baculoviral IAP repeat-containing 5 |
| 4751 | NEK2 | NIMA (never in mitosis gene a)-related kinase 2 |
| 8318 | CDC45L | CDC45 cell division cycle 45-like (S. cerevisiae) |
| 6790 | AURKA | aurora kinase A |
| 10460 | TACC3 | transforming, acidic coiled-coil containing protein 3 |
| 56992 | KIF15 | kinesin family member 15 |
| 699 | BUB1 | BUB1 budding uninhibited by benzimidazoles 1 homolog (yeast) |
| 9585 | KIF20B | kinesin family member 20B |

  
  
**MSCs batch 2 repressed and**GO:0010458**: 4 genes, expected 0.148485, P=1.04786e-05, P adjusted = 1

|  |  |  |
| --- | --- | --- |
| 9735 | KNTC1 | kinetochore associated 1 |
| 332 | BIRC5 | baculoviral IAP repeat-containing 5 |
| 54443 | ANLN | anillin, actin binding protein |
| 11065 | UBE2C | ubiquitin-conjugating enzyme E2C |

  
  
**MSCs batch 2 repressed and**GO:0051640**: 7 genes, expected 0.767175, P=1.12476e-05, P adjusted = 1

|  |  |  |
| --- | --- | --- |
| 9787 | DLGAP5 | discs, large (Drosophila) homolog-associated protein 5 |
| 332 | BIRC5 | baculoviral IAP repeat-containing 5 |
| 113130 | CDCA5 | cell division cycle associated 5 |
| 259266 | ASPM | asp (abnormal spindle) homolog, microcephaly associated (Drosophila) |
| 1062 | CENPE | centromere protein E, 312kDa |
| 51203 | NUSAP1 | nucleolar and spindle associated protein 1 |
| 1063 | CENPF | centromere protein F, 350/400ka (mitosin) |

  
  
**MSCs batch 2 repressed and**GO:0051329**: 8 genes, expected 1.10127, P=1.46294e-05, P adjusted = 1

|  |  |  |
| --- | --- | --- |
| 891 | CCNB1 | cyclin B1 |
| 995 | CDC25C | cell division cycle 25 homolog C (S. pombe) |
| 332 | BIRC5 | baculoviral IAP repeat-containing 5 |
| 113130 | CDCA5 | cell division cycle associated 5 |
| 1063 | CENPF | centromere protein F, 350/400ka (mitosin) |
| 51512 | GTSE1 | G-2 and S-phase expressed 1 |
| 990 | CDC6 | cell division cycle 6 homolog (S. cerevisiae) |
| 1033 | CDKN3 | cyclin-dependent kinase inhibitor 3 |

  
  
**MSCs batch 2 repressed and**GO:0051310**: 3 genes, expected 0.0618689, P=1.83799e-05, P adjusted = 1

|  |  |  |
| --- | --- | --- |
| 113130 | CDCA5 | cell division cycle associated 5 |
| 1062 | CENPE | centromere protein E, 312kDa |
| 1063 | CENPF | centromere protein F, 350/400ka (mitosin) |

  
  
**MSCs batch 2 repressed and**GO:0051439**: 7 genes, expected 0.829044, P=1.88718e-05, P adjusted = 1

|  |  |  |
| --- | --- | --- |
| 983 | CDC2 | cell division cycle 2, G1 to S and G2 to M |
| 26271 | FBXO5 | F-box protein 5 |
| 891 | CCNB1 | cyclin B1 |
| 4085 | MAD2L1 | MAD2 mitotic arrest deficient-like 1 (yeast) |
| 701 | BUB1B | BUB1 budding uninhibited by benzimidazoles 1 homolog beta (yeast) |
| 991 | CDC20 | cell division cycle 20 homolog (S. cerevisiae) |
| 11065 | UBE2C | ubiquitin-conjugating enzyme E2C |

  
  
**MSCs batch 2 repressed and**GO:0051438**: 7 genes, expected 0.890913, P=3.03331e-05, P adjusted = 1

|  |  |  |
| --- | --- | --- |
| 983 | CDC2 | cell division cycle 2, G1 to S and G2 to M |
| 26271 | FBXO5 | F-box protein 5 |
| 891 | CCNB1 | cyclin B1 |
| 4085 | MAD2L1 | MAD2 mitotic arrest deficient-like 1 (yeast) |
| 701 | BUB1B | BUB1 budding uninhibited by benzimidazoles 1 homolog beta (yeast) |
| 991 | CDC20 | cell division cycle 20 homolog (S. cerevisiae) |
| 11065 | UBE2C | ubiquitin-conjugating enzyme E2C |

  
  
**MSCs batch 2 repressed and**GO:0045787**: 5 genes, expected 0.383587, P=3.6402e-05, P adjusted = 1

|  |  |  |
| --- | --- | --- |
| 9787 | DLGAP5 | discs, large (Drosophila) homolog-associated protein 5 |
| 332 | BIRC5 | baculoviral IAP repeat-containing 5 |
| 51203 | NUSAP1 | nucleolar and spindle associated protein 1 |
| 11065 | UBE2C | ubiquitin-conjugating enzyme E2C |
| 3479 | IGF1 | insulin-like growth factor 1 (somatomedin C) |

  
  
**MSCs batch 2 repressed and**GO:0043596**: 4 genes, expected 0.197981, P=3.70528e-05, P adjusted = 1

|  |  |  |
| --- | --- | --- |
| 10714 | POLD3 | polymerase (DNA-directed), delta 3, accessory subunit |
| 4176 | MCM7 | minichromosome maintenance complex component 7 |
| 23649 | POLA2 | polymerase (DNA directed), alpha 2 (70kD subunit) |
| 5557 | PRIM1 | primase, DNA, polypeptide 1 (49kDa) |

  
  
**MSCs batch 2 repressed and**GO:0051656**: 6 genes, expected 0.631063, P=3.81933e-05, P adjusted = 1

|  |  |  |
| --- | --- | --- |
| 9787 | DLGAP5 | discs, large (Drosophila) homolog-associated protein 5 |
| 332 | BIRC5 | baculoviral IAP repeat-containing 5 |
| 113130 | CDCA5 | cell division cycle associated 5 |
| 1062 | CENPE | centromere protein E, 312kDa |
| 51203 | NUSAP1 | nucleolar and spindle associated protein 1 |
| 1063 | CENPF | centromere protein F, 350/400ka (mitosin) |

  
  
**MSCs batch 2 repressed and**GO:0044454**: 7 genes, expected 0.928034, P=3.96042e-05, P adjusted = 1

|  |  |  |
| --- | --- | --- |
| 10714 | POLD3 | polymerase (DNA-directed), delta 3, accessory subunit |
| 4176 | MCM7 | minichromosome maintenance complex component 7 |
| 23649 | POLA2 | polymerase (DNA directed), alpha 2 (70kD subunit) |
| 4171 | MCM2 | minichromosome maintenance complex component 2 |
| 2956 | MSH6 | mutS homolog 6 (E. coli) |
| 5557 | PRIM1 | primase, DNA, polypeptide 1 (49kDa) |
| 8914 | TIMELESS | timeless homolog (Drosophila) |

  
  
**MSCs batch 2 repressed and**GO:0051340**: 7 genes, expected 0.928034, P=3.96042e-05, P adjusted = 1

|  |  |  |
| --- | --- | --- |
| 983 | CDC2 | cell division cycle 2, G1 to S and G2 to M |
| 26271 | FBXO5 | F-box protein 5 |
| 891 | CCNB1 | cyclin B1 |
| 4085 | MAD2L1 | MAD2 mitotic arrest deficient-like 1 (yeast) |
| 701 | BUB1B | BUB1 budding uninhibited by benzimidazoles 1 homolog beta (yeast) |
| 991 | CDC20 | cell division cycle 20 homolog (S. cerevisiae) |
| 11065 | UBE2C | ubiquitin-conjugating enzyme E2C |

  
  
**MSCs batch 2 repressed and**GO:0005871**: 4 genes, expected 0.210354, P=4.79837e-05, P adjusted = 1

|  |  |  |
| --- | --- | --- |
| 3832 | KIF11 | kinesin family member 11 |
| 11004 | KIF2C | kinesin family member 2C |
| 9493 | KIF23 | kinesin family member 23 |
| 56992 | KIF15 | kinesin family member 15 |

  
  
**MSCs batch 2 repressed and**GO:0000228**: 8 genes, expected 1.29925, P=4.87366e-05, P adjusted = 1

|  |  |  |
| --- | --- | --- |
| 10714 | POLD3 | polymerase (DNA-directed), delta 3, accessory subunit |
| 4176 | MCM7 | minichromosome maintenance complex component 7 |
| 23649 | POLA2 | polymerase (DNA directed), alpha 2 (70kD subunit) |
| 4171 | MCM2 | minichromosome maintenance complex component 2 |
| 2956 | MSH6 | mutS homolog 6 (E. coli) |
| 5557 | PRIM1 | primase, DNA, polypeptide 1 (49kDa) |
| 8914 | TIMELESS | timeless homolog (Drosophila) |
| 3148 | HMGB2 | high-mobility group box 2 |

  
  
**MSCs batch 2 repressed and**GO:0000910**: 5 genes, expected 0.420709, P=5.78369e-05, P adjusted = 1

|  |  |  |
| --- | --- | --- |
| 332 | BIRC5 | baculoviral IAP repeat-containing 5 |
| 29127 | RACGAP1 | Rac GTPase activating protein 1 |
| 51203 | NUSAP1 | nucleolar and spindle associated protein 1 |
| 54443 | ANLN | anillin, actin binding protein |
| 9055 | PRC1 | protein regulator of cytokinesis 1 |

  
  
**MSCs batch 2 repressed and**GO:0019932**: 11 genes, expected 2.72223, P=9.79473e-05, P adjusted = 1

|  |  |  |
| --- | --- | --- |
| 7153 | TOP2A | topoisomerase (DNA) II alpha 170kDa |
| 701 | BUB1B | BUB1 budding uninhibited by benzimidazoles 1 homolog beta (yeast) |
| 10403 | NDC80 | NDC80 homolog, kinetochore complex component (S. cerevisiae) |
| 154 | ADRB2 | adrenergic, beta-2-, receptor, surface |
| 11130 | ZWINT | ZW10 interactor |
| 7298 | TYMS | thymidylate synthetase |
| 6790 | AURKA | aurora kinase A |
| 10615 | SPAG5 | sperm associated antigen 5 |
| 11065 | UBE2C | ubiquitin-conjugating enzyme E2C |
| 3479 | IGF1 | insulin-like growth factor 1 (somatomedin C) |
| 3148 | HMGB2 | high-mobility group box 2 |

  
  
**MSCs batch 2 induced and**GO:0048731**: 47 genes, expected 15.7977, P=6.28008e-12, P adjusted = 7.31077e-07

|  |  |  |
| --- | --- | --- |
| 1382 | CRABP2 | cellular retinoic acid binding protein 2 |
| 348 | APOE | apolipoprotein E |
| 3280 | HES1 | hairy and enhancer of split 1, (Drosophila) |
| 84525 | HOPX | HOP homeobox |
| 4879 | NPPB | natriuretic peptide precursor B |
| 2261 | FGFR3 | fibroblast growth factor receptor 3 |
| 23493 | HEY2 | hairy/enhancer-of-split related with YRPW motif 2 |
| 5493 | PPL | periplakin |
| 56130 | PCDHB6 | protocadherin beta 6 |
| 11341 | SCRG1 | scrapie responsive protein 1 |
| 3714 | JAG2 | jagged 2 |
| 10570 | DPYSL4 | dihydropyrimidinase-like 4 |
| 249 | ALPL | alkaline phosphatase, liver/bone/kidney |
| 26508 | HEYL | hairy/enhancer-of-split related with YRPW motif-like |
| 2487 | FRZB | frizzled-related protein |
| 2596 | GAP43 | growth associated protein 43 |
| 8313 | AXIN2 | axin 2 |
| 57801 | HES4 | hairy and enhancer of split 4 (Drosophila) |
| 1306 | COL15A1 | collagen, type XV, alpha 1 |
| 2824 | GPM6B | glycoprotein M6B |
| 652 | BMP4 | bone morphogenetic protein 4 |
| 51176 | LEF1 | lymphoid enhancer-binding factor 1 |
| 50964 | SOST | sclerosteosis |
| 7852 | CXCR4 | chemokine (C-X-C motif) receptor 4 |
| 3872 | KRT17 | keratin 17 |
| 9935 | MAFB | v-maf musculoaponeurotic fibrosarcoma oncogene homolog B (avian) |
| 9118 | INA | internexin neuronal intermediate filament protein, alpha |
| 9806 | SPOCK2 | sparc/osteonectin, cwcv and kazal-like domains proteoglycan (testican) 2 |
| 3213 | HOXB3 | homeobox B3 |
| 51083 | GAL | galanin prepropeptide |
| 3481 | IGF2 | insulin-like growth factor 2 (somatomedin A) |
| 30812 | SOX8 | SRY (sex determining region Y)-box 8 |
| 4744 | NEFH | neurofilament, heavy polypeptide |
| 10202 | DHRS2 | dehydrogenase/reductase (SDR family) member 2 |
| 3955 | LFNG | LFNG O-fucosylpeptide 3-beta-N-acetylglucosaminyltransferase |
| 56126 | PCDHB10 | protocadherin beta 10 |
| 182 | JAG1 | jagged 1 (Alagille syndrome) |
| 1134 | CHRNA1 | cholinergic receptor, nicotinic, alpha 1 (muscle) |
| 3589 | IL11 | interleukin 11 |
| 5734 | PTGER4 | prostaglandin E receptor 4 (subtype EP4) |
| 80303 | EFHD1 | EF-hand domain family, member D1 |
| 3897 | L1CAM | L1 cell adhesion molecule |
| 6586 | SLIT3 | slit homolog 3 (Drosophila) |
| 10417 | SPON2 | spondin 2, extracellular matrix protein |
| 9355 | LHX2 | LIM homeobox 2 |
| 596 | BCL2 | B-cell CLL/lymphoma 2 |
| 3861 | KRT14 | keratin 14 |

  
  
**MSCs batch 2 induced and**GO:0005576**: 46 genes, expected 16.0554, P=4.04832e-11, P adjusted = 4.71273e-06

|  |  |  |
| --- | --- | --- |
| 348 | APOE | apolipoprotein E |
| 4879 | NPPB | natriuretic peptide precursor B |
| 55118 | CRTAC1 | cartilage acidic protein 1 |
| 27151 | CPAMD8 | C3 and PZP-like, alpha-2-macroglobulin domain containing 8 |
| 5730 | PTGDS | prostaglandin D2 synthase 21kDa (brain) |
| 9547 | CXCL14 | chemokine (C-X-C motif) ligand 14 |
| 2239 | GPC4 | glypican 4 |
| 11341 | SCRG1 | scrapie responsive protein 1 |
| 6422 | SFRP1 | secreted frizzled-related protein 1 |
| 25891 | DKFZP586H2123 | regeneration associated muscle protease |
| 4885 | NPTX2 | neuronal pentraxin II |
| 25878 | MXRA5 | matrix-remodelling associated 5 |
| 2487 | FRZB | frizzled-related protein |
| 718 | C3 | complement component 3 |
| 1306 | COL15A1 | collagen, type XV, alpha 1 |
| 1191 | CLU | clusterin |
| 652 | BMP4 | bone morphogenetic protein 4 |
| 7292 | TNFSF4 | tumor necrosis factor (ligand) superfamily, member 4 |
| 157869 | RPESP | RPE-spondin |
| 7481 | WNT11 | wingless-type MMTV integration site family, member 11 |
| 50964 | SOST | sclerosteosis |
| 9806 | SPOCK2 | sparc/osteonectin, cwcv and kazal-like domains proteoglycan (testican) 2 |
| 2263 | FGFR2 | fibroblast growth factor receptor 2 |
| 8788 | DLK1 | delta-like 1 homolog (Drosophila) |
| 51083 | GAL | galanin prepropeptide |
| 3481 | IGF2 | insulin-like growth factor 2 (somatomedin A) |
| 55959 | SULF2 | sulfatase 2 |
| 90139 | TSPAN18 | tetraspanin 18 |
| 256691 | MAMDC2 | MAM domain containing 2 |
| 834 | CASP1 | caspase 1, apoptosis-related cysteine peptidase (interleukin 1, beta, convertase) |
| 3955 | LFNG | LFNG O-fucosylpeptide 3-beta-N-acetylglucosaminyltransferase |
| 182 | JAG1 | jagged 1 (Alagille syndrome) |
| 9388 | LIPG | lipase, endothelial |
| 1525 | CXADR | coxsackie virus and adenovirus receptor |
| 3589 | IL11 | interleukin 11 |
| 5919 | RARRES2 | retinoic acid receptor responder (tazarotene induced) 2 |
| 83872 | HMCN1 | hemicentin 1 |
| 1299 | COL9A3 | collagen, type IX, alpha 3 |
| 6586 | SLIT3 | slit homolog 3 (Drosophila) |
| 11197 | WIF1 | WNT inhibitory factor 1 |
| 10417 | SPON2 | spondin 2, extracellular matrix protein |
| 8532 | CPZ | carboxypeptidase Z |
| 7093 | TLL2 | tolloid-like 2 |
| 3977 | LIFR | leukemia inhibitory factor receptor alpha |
| 80310 | PDGFD | platelet derived growth factor D |
| 2266 | FGG | fibrinogen gamma chain |

  
  
**MSCs batch 2 induced and**GO:0007399**: 26 genes, expected 6.67442, P=3.06728e-09, P adjusted = 0.000357069

|  |  |  |
| --- | --- | --- |
| 348 | APOE | apolipoprotein E |
| 3280 | HES1 | hairy and enhancer of split 1, (Drosophila) |
| 2261 | FGFR3 | fibroblast growth factor receptor 3 |
| 23493 | HEY2 | hairy/enhancer-of-split related with YRPW motif 2 |
| 56130 | PCDHB6 | protocadherin beta 6 |
| 11341 | SCRG1 | scrapie responsive protein 1 |
| 10570 | DPYSL4 | dihydropyrimidinase-like 4 |
| 26508 | HEYL | hairy/enhancer-of-split related with YRPW motif-like |
| 2596 | GAP43 | growth associated protein 43 |
| 57801 | HES4 | hairy and enhancer of split 4 (Drosophila) |
| 2824 | GPM6B | glycoprotein M6B |
| 652 | BMP4 | bone morphogenetic protein 4 |
| 51176 | LEF1 | lymphoid enhancer-binding factor 1 |
| 7852 | CXCR4 | chemokine (C-X-C motif) receptor 4 |
| 9118 | INA | internexin neuronal intermediate filament protein, alpha |
| 9806 | SPOCK2 | sparc/osteonectin, cwcv and kazal-like domains proteoglycan (testican) 2 |
| 51083 | GAL | galanin prepropeptide |
| 30812 | SOX8 | SRY (sex determining region Y)-box 8 |
| 4744 | NEFH | neurofilament, heavy polypeptide |
| 56126 | PCDHB10 | protocadherin beta 10 |
| 182 | JAG1 | jagged 1 (Alagille syndrome) |
| 80303 | EFHD1 | EF-hand domain family, member D1 |
| 3897 | L1CAM | L1 cell adhesion molecule |
| 6586 | SLIT3 | slit homolog 3 (Drosophila) |
| 10417 | SPON2 | spondin 2, extracellular matrix protein |
| 9355 | LHX2 | LIM homeobox 2 |

  
  
**MSCs batch 2 induced and**GO:0009888**: 17 genes, expected 3.01039, P=1.01235e-08, P adjusted = 0.0011785

|  |  |  |
| --- | --- | --- |
| 1382 | CRABP2 | cellular retinoic acid binding protein 2 |
| 3280 | HES1 | hairy and enhancer of split 1, (Drosophila) |
| 84525 | HOPX | HOP homeobox |
| 2261 | FGFR3 | fibroblast growth factor receptor 3 |
| 5493 | PPL | periplakin |
| 3714 | JAG2 | jagged 2 |
| 249 | ALPL | alkaline phosphatase, liver/bone/kidney |
| 8313 | AXIN2 | axin 2 |
| 652 | BMP4 | bone morphogenetic protein 4 |
| 51176 | LEF1 | lymphoid enhancer-binding factor 1 |
| 50964 | SOST | sclerosteosis |
| 3872 | KRT17 | keratin 17 |
| 182 | JAG1 | jagged 1 (Alagille syndrome) |
| 5734 | PTGER4 | prostaglandin E receptor 4 (subtype EP4) |
| 9355 | LHX2 | LIM homeobox 2 |
| 596 | BCL2 | B-cell CLL/lymphoma 2 |
| 3861 | KRT14 | keratin 14 |

  
  
**MSCs batch 2 induced and**GO:0048869**: 34 genes, expected 11.8206, P=2.13789e-08, P adjusted = 0.00248876

|  |  |  |
| --- | --- | --- |
| 348 | APOE | apolipoprotein E |
| 3280 | HES1 | hairy and enhancer of split 1, (Drosophila) |
| 84525 | HOPX | HOP homeobox |
| 2261 | FGFR3 | fibroblast growth factor receptor 3 |
| 23493 | HEY2 | hairy/enhancer-of-split related with YRPW motif 2 |
| 5493 | PPL | periplakin |
| 6422 | SFRP1 | secreted frizzled-related protein 1 |
| 3714 | JAG2 | jagged 2 |
| 2487 | FRZB | frizzled-related protein |
| 2596 | GAP43 | growth associated protein 43 |
| 8313 | AXIN2 | axin 2 |
| 57801 | HES4 | hairy and enhancer of split 4 (Drosophila) |
| 1306 | COL15A1 | collagen, type XV, alpha 1 |
| 2824 | GPM6B | glycoprotein M6B |
| 652 | BMP4 | bone morphogenetic protein 4 |
| 1475 | CSTA | cystatin A (stefin A) |
| 51176 | LEF1 | lymphoid enhancer-binding factor 1 |
| 7852 | CXCR4 | chemokine (C-X-C motif) receptor 4 |
| 9935 | MAFB | v-maf musculoaponeurotic fibrosarcoma oncogene homolog B (avian) |
| 9118 | INA | internexin neuronal intermediate filament protein, alpha |
| 9806 | SPOCK2 | sparc/osteonectin, cwcv and kazal-like domains proteoglycan (testican) 2 |
| 360 | AQP3 | aquaporin 3 (Gill blood group) |
| 30812 | SOX8 | SRY (sex determining region Y)-box 8 |
| 10202 | DHRS2 | dehydrogenase/reductase (SDR family) member 2 |
| 182 | JAG1 | jagged 1 (Alagille syndrome) |
| 1134 | CHRNA1 | cholinergic receptor, nicotinic, alpha 1 (muscle) |
| 3589 | IL11 | interleukin 11 |
| 80303 | EFHD1 | EF-hand domain family, member D1 |
| 3897 | L1CAM | L1 cell adhesion molecule |
| 6586 | SLIT3 | slit homolog 3 (Drosophila) |
| 10417 | SPON2 | spondin 2, extracellular matrix protein |
| 1739 | DLG1 | discs, large homolog 1 (Drosophila) |
| 7093 | TLL2 | tolloid-like 2 |
| 58480 | RHOU | ras homolog gene family, member U |

  
  
**MSCs batch 2 induced and**GO:0030154**: 32 genes, expected 10.7711, P=2.81577e-08, P adjusted = 0.00327789

|  |  |  |
| --- | --- | --- |
| 348 | APOE | apolipoprotein E |
| 3280 | HES1 | hairy and enhancer of split 1, (Drosophila) |
| 84525 | HOPX | HOP homeobox |
| 2261 | FGFR3 | fibroblast growth factor receptor 3 |
| 23493 | HEY2 | hairy/enhancer-of-split related with YRPW motif 2 |
| 5493 | PPL | periplakin |
| 6422 | SFRP1 | secreted frizzled-related protein 1 |
| 3714 | JAG2 | jagged 2 |
| 2487 | FRZB | frizzled-related protein |
| 2596 | GAP43 | growth associated protein 43 |
| 8313 | AXIN2 | axin 2 |
| 57801 | HES4 | hairy and enhancer of split 4 (Drosophila) |
| 1306 | COL15A1 | collagen, type XV, alpha 1 |
| 2824 | GPM6B | glycoprotein M6B |
| 652 | BMP4 | bone morphogenetic protein 4 |
| 1475 | CSTA | cystatin A (stefin A) |
| 51176 | LEF1 | lymphoid enhancer-binding factor 1 |
| 7852 | CXCR4 | chemokine (C-X-C motif) receptor 4 |
| 9935 | MAFB | v-maf musculoaponeurotic fibrosarcoma oncogene homolog B (avian) |
| 9118 | INA | internexin neuronal intermediate filament protein, alpha |
| 9806 | SPOCK2 | sparc/osteonectin, cwcv and kazal-like domains proteoglycan (testican) 2 |
| 360 | AQP3 | aquaporin 3 (Gill blood group) |
| 30812 | SOX8 | SRY (sex determining region Y)-box 8 |
| 10202 | DHRS2 | dehydrogenase/reductase (SDR family) member 2 |
| 182 | JAG1 | jagged 1 (Alagille syndrome) |
| 1134 | CHRNA1 | cholinergic receptor, nicotinic, alpha 1 (muscle) |
| 3589 | IL11 | interleukin 11 |
| 80303 | EFHD1 | EF-hand domain family, member D1 |
| 3897 | L1CAM | L1 cell adhesion molecule |
| 6586 | SLIT3 | slit homolog 3 (Drosophila) |
| 10417 | SPON2 | spondin 2, extracellular matrix protein |
| 7093 | TLL2 | tolloid-like 2 |

  
  
**MSCs batch 2 induced and**GO:0048729**: 8 genes, expected 0.736488, P=7.30652e-07, P adjusted = 0.0850567

|  |  |  |
| --- | --- | --- |
| 3280 | HES1 | hairy and enhancer of split 1, (Drosophila) |
| 2261 | FGFR3 | fibroblast growth factor receptor 3 |
| 5493 | PPL | periplakin |
| 3714 | JAG2 | jagged 2 |
| 652 | BMP4 | bone morphogenetic protein 4 |
| 51176 | LEF1 | lymphoid enhancer-binding factor 1 |
| 182 | JAG1 | jagged 1 (Alagille syndrome) |
| 596 | BCL2 | B-cell CLL/lymphoma 2 |

  
  
**MSCs batch 2 induced and**GO:0044421**: 22 genes, expected 6.80331, P=1.31681e-06, P adjusted = 0.153293

|  |  |  |
| --- | --- | --- |
| 348 | APOE | apolipoprotein E |
| 4879 | NPPB | natriuretic peptide precursor B |
| 9547 | CXCL14 | chemokine (C-X-C motif) ligand 14 |
| 2239 | GPC4 | glypican 4 |
| 11341 | SCRG1 | scrapie responsive protein 1 |
| 6422 | SFRP1 | secreted frizzled-related protein 1 |
| 1306 | COL15A1 | collagen, type XV, alpha 1 |
| 1191 | CLU | clusterin |
| 652 | BMP4 | bone morphogenetic protein 4 |
| 7292 | TNFSF4 | tumor necrosis factor (ligand) superfamily, member 4 |
| 7481 | WNT11 | wingless-type MMTV integration site family, member 11 |
| 9806 | SPOCK2 | sparc/osteonectin, cwcv and kazal-like domains proteoglycan (testican) 2 |
| 8788 | DLK1 | delta-like 1 homolog (Drosophila) |
| 55959 | SULF2 | sulfatase 2 |
| 90139 | TSPAN18 | tetraspanin 18 |
| 3589 | IL11 | interleukin 11 |
| 83872 | HMCN1 | hemicentin 1 |
| 1299 | COL9A3 | collagen, type IX, alpha 3 |
| 6586 | SLIT3 | slit homolog 3 (Drosophila) |
| 10417 | SPON2 | spondin 2, extracellular matrix protein |
| 8532 | CPZ | carboxypeptidase Z |
| 2266 | FGG | fibrinogen gamma chain |

  
  
**MSCs batch 2 induced and**GO:0048513**: 30 genes, expected 11.793, P=2.14223e-06, P adjusted = 0.249381

|  |  |  |
| --- | --- | --- |
| 1382 | CRABP2 | cellular retinoic acid binding protein 2 |
| 3280 | HES1 | hairy and enhancer of split 1, (Drosophila) |
| 84525 | HOPX | HOP homeobox |
| 4879 | NPPB | natriuretic peptide precursor B |
| 2261 | FGFR3 | fibroblast growth factor receptor 3 |
| 23493 | HEY2 | hairy/enhancer-of-split related with YRPW motif 2 |
| 5493 | PPL | periplakin |
| 3714 | JAG2 | jagged 2 |
| 249 | ALPL | alkaline phosphatase, liver/bone/kidney |
| 2487 | FRZB | frizzled-related protein |
| 8313 | AXIN2 | axin 2 |
| 1306 | COL15A1 | collagen, type XV, alpha 1 |
| 652 | BMP4 | bone morphogenetic protein 4 |
| 51176 | LEF1 | lymphoid enhancer-binding factor 1 |
| 50964 | SOST | sclerosteosis |
| 7852 | CXCR4 | chemokine (C-X-C motif) receptor 4 |
| 3872 | KRT17 | keratin 17 |
| 9935 | MAFB | v-maf musculoaponeurotic fibrosarcoma oncogene homolog B (avian) |
| 3213 | HOXB3 | homeobox B3 |
| 3481 | IGF2 | insulin-like growth factor 2 (somatomedin A) |
| 10202 | DHRS2 | dehydrogenase/reductase (SDR family) member 2 |
| 3955 | LFNG | LFNG O-fucosylpeptide 3-beta-N-acetylglucosaminyltransferase |
| 182 | JAG1 | jagged 1 (Alagille syndrome) |
| 1134 | CHRNA1 | cholinergic receptor, nicotinic, alpha 1 (muscle) |
| 3589 | IL11 | interleukin 11 |
| 5734 | PTGER4 | prostaglandin E receptor 4 (subtype EP4) |
| 6586 | SLIT3 | slit homolog 3 (Drosophila) |
| 9355 | LHX2 | LIM homeobox 2 |
| 596 | BCL2 | B-cell CLL/lymphoma 2 |
| 3861 | KRT14 | keratin 14 |

  
  
**MSCs batch 2 induced and**GO:0007166**: 32 genes, expected 13.1095, P=2.24971e-06, P adjusted = 0.261893

|  |  |  |
| --- | --- | --- |
| 348 | APOE | apolipoprotein E |
| 1028 | CDKN1C | cyclin-dependent kinase inhibitor 1C (p57, Kip2) |
| 4879 | NPPB | natriuretic peptide precursor B |
| 2261 | FGFR3 | fibroblast growth factor receptor 3 |
| 23493 | HEY2 | hairy/enhancer-of-split related with YRPW motif 2 |
| 6422 | SFRP1 | secreted frizzled-related protein 1 |
| 3714 | JAG2 | jagged 2 |
| 26508 | HEYL | hairy/enhancer-of-split related with YRPW motif-like |
| 2487 | FRZB | frizzled-related protein |
| 2596 | GAP43 | growth associated protein 43 |
| 8313 | AXIN2 | axin 2 |
| 718 | C3 | complement component 3 |
| 652 | BMP4 | bone morphogenetic protein 4 |
| 84634 | KISS1R | KISS1 receptor |
| 27065 | D4S234E | DNA segment on chromosome 4 (unique) 234 expressed sequence |
| 7481 | WNT11 | wingless-type MMTV integration site family, member 11 |
| 51176 | LEF1 | lymphoid enhancer-binding factor 1 |
| 50964 | SOST | sclerosteosis |
| 7852 | CXCR4 | chemokine (C-X-C motif) receptor 4 |
| 2263 | FGFR2 | fibroblast growth factor receptor 2 |
| 51083 | GAL | galanin prepropeptide |
| 3481 | IGF2 | insulin-like growth factor 2 (somatomedin A) |
| 3680 | ITGA9 | integrin, alpha 9 |
| 59352 | LGR6 | leucine-rich repeat-containing G protein-coupled receptor 6 |
| 182 | JAG1 | jagged 1 (Alagille syndrome) |
| 9568 | GABBR2 | gamma-aminobutyric acid (GABA) B receptor, 2 |
| 5734 | PTGER4 | prostaglandin E receptor 4 (subtype EP4) |
| 11197 | WIF1 | WNT inhibitory factor 1 |
| 8532 | CPZ | carboxypeptidase Z |
| 8549 | LGR5 | leucine-rich repeat-containing G protein-coupled receptor 5 |
| 3977 | LIFR | leukemia inhibitory factor receptor alpha |
| 51655 | RASD1 | RAS, dexamethasone-induced 1 |

  
  
**MSCs batch 2 induced and**GO:0008544**: 9 genes, expected 1.28885, P=6.08278e-06, P adjusted = 0.708108

|  |  |  |
| --- | --- | --- |
| 1382 | CRABP2 | cellular retinoic acid binding protein 2 |
| 3280 | HES1 | hairy and enhancer of split 1, (Drosophila) |
| 2261 | FGFR3 | fibroblast growth factor receptor 3 |
| 5493 | PPL | periplakin |
| 3714 | JAG2 | jagged 2 |
| 3872 | KRT17 | keratin 17 |
| 182 | JAG1 | jagged 1 (Alagille syndrome) |
| 596 | BCL2 | B-cell CLL/lymphoma 2 |
| 3861 | KRT14 | keratin 14 |

  
  
**MSCs batch 2 induced and**GO:0007398**: 9 genes, expected 1.38091, P=1.06495e-05, P adjusted = 1

|  |  |  |
| --- | --- | --- |
| 1382 | CRABP2 | cellular retinoic acid binding protein 2 |
| 3280 | HES1 | hairy and enhancer of split 1, (Drosophila) |
| 2261 | FGFR3 | fibroblast growth factor receptor 3 |
| 5493 | PPL | periplakin |
| 3714 | JAG2 | jagged 2 |
| 3872 | KRT17 | keratin 17 |
| 182 | JAG1 | jagged 1 (Alagille syndrome) |
| 596 | BCL2 | B-cell CLL/lymphoma 2 |
| 3861 | KRT14 | keratin 14 |

  
  
**MSCs batch 2 induced and**GO:0048730**: 6 genes, expected 0.524748, P=1.3799e-05, P adjusted = 1

|  |  |  |
| --- | --- | --- |
| 3280 | HES1 | hairy and enhancer of split 1, (Drosophila) |
| 2261 | FGFR3 | fibroblast growth factor receptor 3 |
| 5493 | PPL | periplakin |
| 3714 | JAG2 | jagged 2 |
| 182 | JAG1 | jagged 1 (Alagille syndrome) |
| 596 | BCL2 | B-cell CLL/lymphoma 2 |

  
  
**MSCs batch 2 induced and**GO:0060113**: 4 genes, expected 0.16571, P=1.92374e-05, P adjusted = 1

|  |  |  |
| --- | --- | --- |
| 3280 | HES1 | hairy and enhancer of split 1, (Drosophila) |
| 2261 | FGFR3 | fibroblast growth factor receptor 3 |
| 3714 | JAG2 | jagged 2 |
| 182 | JAG1 | jagged 1 (Alagille syndrome) |

  
  
**MSCs batch 2 induced and**GO:0005509**: 22 genes, expected 8.13819, P=2.24823e-05, P adjusted = 1

|  |  |  |
| --- | --- | --- |
| 55118 | CRTAC1 | cartilage acidic protein 1 |
| 56130 | PCDHB6 | protocadherin beta 6 |
| 3714 | JAG2 | jagged 2 |
| 4885 | NPTX2 | neuronal pentraxin II |
| 164633 | CABP7 | calcium binding protein 7 |
| 56129 | PCDHB7 | protocadherin beta 7 |
| 11240 | PADI2 | peptidyl arginine deiminase, type II |
| 117248 | GALNTL2 | UDP-N-acetyl-alpha-D-galactosamine:polypeptide N-acetylgalactosaminyltransferase-like 2 |
| 9806 | SPOCK2 | sparc/osteonectin, cwcv and kazal-like domains proteoglycan (testican) 2 |
| 8788 | DLK1 | delta-like 1 homolog (Drosophila) |
| 57134 | MAN1C1 | mannosidase, alpha, class 1C, member 1 |
| 9752 | PCDHA9 | protocadherin alpha 9 |
| 3680 | ITGA9 | integrin, alpha 9 |
| 55959 | SULF2 | sulfatase 2 |
| 56126 | PCDHB10 | protocadherin beta 10 |
| 182 | JAG1 | jagged 1 (Alagille syndrome) |
| 83872 | HMCN1 | hemicentin 1 |
| 80303 | EFHD1 | EF-hand domain family, member D1 |
| 6586 | SLIT3 | slit homolog 3 (Drosophila) |
| 7093 | TLL2 | tolloid-like 2 |
| 57452 | GALNTL1 | UDP-N-acetyl-alpha-D-galactosamine:polypeptide N-acetylgalactosaminyltransferase-like 1 |
| 2266 | FGG | fibrinogen gamma chain |

  
  
**MSCs batch 2 induced and**GO:0042490**: 4 genes, expected 0.184122, P=3.00234e-05, P adjusted = 1

|  |  |  |
| --- | --- | --- |
| 3280 | HES1 | hairy and enhancer of split 1, (Drosophila) |
| 2261 | FGFR3 | fibroblast growth factor receptor 3 |
| 3714 | JAG2 | jagged 2 |
| 182 | JAG1 | jagged 1 (Alagille syndrome) |

  
  
**MSCs batch 2 induced and**GO:0035315**: 4 genes, expected 0.193328, P=3.68215e-05, P adjusted = 1

|  |  |  |
| --- | --- | --- |
| 3280 | HES1 | hairy and enhancer of split 1, (Drosophila) |
| 2261 | FGFR3 | fibroblast growth factor receptor 3 |
| 3714 | JAG2 | jagged 2 |
| 182 | JAG1 | jagged 1 (Alagille syndrome) |

  
  
**MSCs batch 2 induced and**GO:0008283**: 20 genes, expected 7.41091, P=5.55562e-05, P adjusted = 1

|  |  |  |
| --- | --- | --- |
| 348 | APOE | apolipoprotein E |
| 3280 | HES1 | hairy and enhancer of split 1, (Drosophila) |
| 1028 | CDKN1C | cyclin-dependent kinase inhibitor 1C (p57, Kip2) |
| 1396 | CRIP1 | cysteine-rich protein 1 (intestinal) |
| 2261 | FGFR3 | fibroblast growth factor receptor 3 |
| 27074 | LAMP3 | lysosomal-associated membrane protein 3 |
| 2239 | GPC4 | glypican 4 |
| 3714 | JAG2 | jagged 2 |
| 8313 | AXIN2 | axin 2 |
| 7292 | TNFSF4 | tumor necrosis factor (ligand) superfamily, member 4 |
| 84634 | KISS1R | KISS1 receptor |
| 7852 | CXCR4 | chemokine (C-X-C motif) receptor 4 |
| 3481 | IGF2 | insulin-like growth factor 2 (somatomedin A) |
| 10202 | DHRS2 | dehydrogenase/reductase (SDR family) member 2 |
| 182 | JAG1 | jagged 1 (Alagille syndrome) |
| 3589 | IL11 | interleukin 11 |
| 1739 | DLG1 | discs, large homolog 1 (Drosophila) |
| 596 | BCL2 | B-cell CLL/lymphoma 2 |
| 3977 | LIFR | leukemia inhibitory factor receptor alpha |
| 80310 | PDGFD | platelet derived growth factor D |

  
  
**MSCs batch 2 induced and**GO:0009913**: 5 genes, expected 0.432687, P=7.02493e-05, P adjusted = 1

|  |  |  |
| --- | --- | --- |
| 3280 | HES1 | hairy and enhancer of split 1, (Drosophila) |
| 2261 | FGFR3 | fibroblast growth factor receptor 3 |
| 5493 | PPL | periplakin |
| 3714 | JAG2 | jagged 2 |
| 182 | JAG1 | jagged 1 (Alagille syndrome) |

  
  
**MSCs batch 2 induced and**GO:0048839**: 5 genes, expected 0.441893, P=7.78367e-05, P adjusted = 1

|  |  |  |
| --- | --- | --- |
| 3280 | HES1 | hairy and enhancer of split 1, (Drosophila) |
| 2261 | FGFR3 | fibroblast growth factor receptor 3 |
| 3714 | JAG2 | jagged 2 |
| 9935 | MAFB | v-maf musculoaponeurotic fibrosarcoma oncogene homolog B (avian) |
| 182 | JAG1 | jagged 1 (Alagille syndrome) |

  
  
**MSCs batch 2 induced and**GO:0045595**: 9 genes, expected 1.78598, P=8.0718e-05, P adjusted = 1

|  |  |  |
| --- | --- | --- |
| 3280 | HES1 | hairy and enhancer of split 1, (Drosophila) |
| 84525 | HOPX | HOP homeobox |
| 2261 | FGFR3 | fibroblast growth factor receptor 3 |
| 8313 | AXIN2 | axin 2 |
| 652 | BMP4 | bone morphogenetic protein 4 |
| 9935 | MAFB | v-maf musculoaponeurotic fibrosarcoma oncogene homolog B (avian) |
| 9806 | SPOCK2 | sparc/osteonectin, cwcv and kazal-like domains proteoglycan (testican) 2 |
| 360 | AQP3 | aquaporin 3 (Gill blood group) |
| 182 | JAG1 | jagged 1 (Alagille syndrome) |

  
**MSCs batch 3 repressed and**GO:0005576**: 95 genes, expected 26.1548, P=5.5823e-30, P adjusted = 6.49847e-25

|  |  |  |
| --- | --- | --- |
| 6387 | CXCL12 | chemokine (C-X-C motif) ligand 12 (stromal cell-derived factor 1) |
| 5069 | PAPPA | pregnancy-associated plasma protein A, pappalysin 1 |
| 5197 | PF4V1 | platelet factor 4 variant 1 |
| 10418 | SPON1 | spondin 1, extracellular matrix protein |
| 1301 | COL11A1 | collagen, type XI, alpha 1 |
| 4582 | MUC1 | mucin 1, cell surface associated |
| 2247 | FGF2 | fibroblast growth factor 2 (basic) |
| 8076 | MFAP5 | microfibrillar associated protein 5 |
| 4811 | NID1 | nidogen 1 |
| 5673 | PSG5 | pregnancy specific beta-1-glycoprotein 5 |
| 7057 | THBS1 | thrombospondin 1 |
| 11341 | SCRG1 | scrapie responsive protein 1 |
| 1284 | COL4A2 | collagen, type IV, alpha 2 |
| 1282 | COL4A1 | collagen, type IV, alpha 1 |
| 182 | JAG1 | jagged 1 (Alagille syndrome) |
| 11098 | PRSS23 | protease, serine, 23 |
| 1490 | CTGF | connective tissue growth factor |
| 2192 | FBLN1 | fibulin 1 |
| 2200 | FBN1 | fibrillin 1 |
| 1404 | HAPLN1 | hyaluronan and proteoglycan link protein 1 |
| 629 | CFB | complement factor B |
| 1306 | COL15A1 | collagen, type XV, alpha 1 |
| 7058 | THBS2 | thrombospondin 2 |
| 8839 | WISP2 | WNT1 inducible signaling pathway protein 2 |
| 652 | BMP4 | bone morphogenetic protein 4 |
| 7123 | CLEC3B | C-type lectin domain family 3, member B |
| 9358 | ITGBL1 | integrin, beta-like 1 (with EGF-like repeat domains) |
| 4982 | TNFRSF11B | tumor necrosis factor receptor superfamily, member 11b |
| 4015 | LOX | lysyl oxidase |
| 6424 | SFRP4 | secreted frizzled-related protein 4 |
| 51232 | CRIM1 | cysteine rich transmembrane BMP regulator 1 (chordin-like) |
| 1842 | ECM2 | extracellular matrix protein 2, female organ and adipocyte specific |
| 183 | AGT | angiotensinogen (serpin peptidase inhibitor, clade A, member 8) |
| 10468 | FST | follistatin |
| 5648 | MASP1 | mannan-binding lectin serine peptidase 1 (C4/C2 activating component of Ra-reactive factor) |
| 7412 | VCAM1 | vascular cell adhesion molecule 1 |
| 6318 | SERPINB4 | serpin peptidase inhibitor, clade B (ovalbumin), member 4 |
| 284 | ANGPT1 | angiopoietin 1 |
| 4653 | MYOC | myocilin, trabecular meshwork inducible glucocorticoid response |
| 10516 | FBLN5 | fibulin 5 |
| 12 | SERPINA3 | serpin peptidase inhibitor, clade A (alpha-1 antiproteinase, antitrypsin), member 3 |
| 55801 | IL26 | interleukin 26 |
| 1278 | COL1A2 | collagen, type I, alpha 2 |
| 57493 | HEG1 | HEG homolog 1 (zebrafish) |
| 1289 | COL5A1 | collagen, type V, alpha 1 |
| 3929 | LBP | lipopolysaccharide binding protein |
| 5549 | PRELP | proline/arginine-rich end leucine-rich repeat protein |
| 176 | ACAN | aggrecan |
| 1303 | COL12A1 | collagen, type XII, alpha 1 |
| 151887 | CCDC80 | coiled-coil domain containing 80 |
| 1277 | COL1A1 | collagen, type I, alpha 1 |
| 3339 | HSPG2 | heparan sulfate proteoglycan 2 |
| 2202 | EFEMP1 | EGF-containing fibulin-like extracellular matrix protein 1 |
| 10875 | FGL2 | fibrinogen-like 2 |
| 81578 | COL21A1 | collagen, type XXI, alpha 1 |
| 9635 | CLCA2 | CLCA family member 2, chloride channel regulator |
| 8532 | CPZ | carboxypeptidase Z |
| 5168 | ENPP2 | ectonucleotide pyrophosphatase/phosphodiesterase 2 |
| 6347 | CCL2 | chemokine (C-C motif) ligand 2 |
| 5054 | SERPINE1 | serpin peptidase inhibitor, clade E (nexin, plasminogen activator inhibitor type 1), member 1 |
| 7049 | TGFBR3 | transforming growth factor, beta receptor III |
| 84623 | KIRREL3 | kin of IRRE like 3 (Drosophila) |
| 1116 | CHI3L1 | chitinase 3-like 1 (cartilage glycoprotein-39) |
| 79987 | SVEP1 | sushi, von Willebrand factor type A, EGF and pentraxin domain containing 1 |
| 5806 | PTX3 | pentraxin-related gene, rapidly induced by IL-1 beta |
| 1281 | COL3A1 | collagen, type III, alpha 1 |
| 6678 | SPARC | secreted protein, acidic, cysteine-rich (osteonectin) |
| 4856 | NOV | nephroblastoma overexpressed gene |
| 4803 | NGF | nerve growth factor (beta polypeptide) |
| 127435 | PODN | podocan |
| 1634 | DCN | decorin |
| 25878 | MXRA5 | matrix-remodelling associated 5 |
| 1462 | VCAN | versican |
| 718 | C3 | complement component 3 |
| 716 | C1S | complement component 1, s subcomponent |
| 3485 | IGFBP2 | insulin-like growth factor binding protein 2, 36kDa |
| 2252 | FGF7 | fibroblast growth factor 7 (keratinocyte growth factor) |
| 5678 | PSG9 | pregnancy specific beta-1-glycoprotein 9 |
| 6372 | CXCL6 | chemokine (C-X-C motif) ligand 6 (granulocyte chemotactic protein 2) |
| 3512 | IGJ | immunoglobulin J polypeptide, linker protein for immunoglobulin alpha and mu polypeptides |
| 2263 | FGFR2 | fibroblast growth factor receptor 2 |
| 3575 | IL7R | interleukin 7 receptor |
| 79875 | THSD4 | thrombospondin, type I, domain containing 4 |
| 165 | AEBP1 | AE binding protein 1 |
| 10082 | GPC6 | glypican 6 |
| 3381 | IBSP | integrin-binding sialoprotein |
| 6590 | SLPI | secretory leukocyte peptidase inhibitor |
| 2331 | FMOD | fibromodulin |
| 715 | C1R | complement component 1, r subcomponent |
| 3918 | LAMC2 | laminin, gamma 2 |
| 1290 | COL5A2 | collagen, type V, alpha 2 |
| 4256 | MGP | matrix Gla protein |
| 23213 | SULF1 | sulfatase 1 |
| 1906 | EDN1 | endothelin 1 |
| 80310 | PDGFD | platelet derived growth factor D |

  
  
**MSCs batch 3 repressed and**GO:0031012**: 38 genes, expected 4.51411, P=3.79761e-24, P adjusted = 4.42087e-19

|  |  |  |
| --- | --- | --- |
| 10418 | SPON1 | spondin 1, extracellular matrix protein |
| 1301 | COL11A1 | collagen, type XI, alpha 1 |
| 8076 | MFAP5 | microfibrillar associated protein 5 |
| 4811 | NID1 | nidogen 1 |
| 1284 | COL4A2 | collagen, type IV, alpha 2 |
| 1282 | COL4A1 | collagen, type IV, alpha 1 |
| 1490 | CTGF | connective tissue growth factor |
| 2192 | FBLN1 | fibulin 1 |
| 2200 | FBN1 | fibrillin 1 |
| 1404 | HAPLN1 | hyaluronan and proteoglycan link protein 1 |
| 1306 | COL15A1 | collagen, type XV, alpha 1 |
| 652 | BMP4 | bone morphogenetic protein 4 |
| 4982 | TNFRSF11B | tumor necrosis factor receptor superfamily, member 11b |
| 4015 | LOX | lysyl oxidase |
| 1842 | ECM2 | extracellular matrix protein 2, female organ and adipocyte specific |
| 10516 | FBLN5 | fibulin 5 |
| 1278 | COL1A2 | collagen, type I, alpha 2 |
| 1289 | COL5A1 | collagen, type V, alpha 1 |
| 5549 | PRELP | proline/arginine-rich end leucine-rich repeat protein |
| 176 | ACAN | aggrecan |
| 1303 | COL12A1 | collagen, type XII, alpha 1 |
| 1277 | COL1A1 | collagen, type I, alpha 1 |
| 3339 | HSPG2 | heparan sulfate proteoglycan 2 |
| 2202 | EFEMP1 | EGF-containing fibulin-like extracellular matrix protein 1 |
| 81578 | COL21A1 | collagen, type XXI, alpha 1 |
| 8532 | CPZ | carboxypeptidase Z |
| 1116 | CHI3L1 | chitinase 3-like 1 (cartilage glycoprotein-39) |
| 1281 | COL3A1 | collagen, type III, alpha 1 |
| 6678 | SPARC | secreted protein, acidic, cysteine-rich (osteonectin) |
| 127435 | PODN | podocan |
| 1634 | DCN | decorin |
| 1462 | VCAN | versican |
| 79875 | THSD4 | thrombospondin, type I, domain containing 4 |
| 10082 | GPC6 | glypican 6 |
| 2331 | FMOD | fibromodulin |
| 3918 | LAMC2 | laminin, gamma 2 |
| 1290 | COL5A2 | collagen, type V, alpha 2 |
| 4256 | MGP | matrix Gla protein |

  
  
**MSCs batch 3 repressed and**GO:0005578**: 37 genes, expected 4.31914, P=8.40076e-24, P adjusted = 9.7795e-19

|  |  |  |
| --- | --- | --- |
| 10418 | SPON1 | spondin 1, extracellular matrix protein |
| 1301 | COL11A1 | collagen, type XI, alpha 1 |
| 8076 | MFAP5 | microfibrillar associated protein 5 |
| 4811 | NID1 | nidogen 1 |
| 1284 | COL4A2 | collagen, type IV, alpha 2 |
| 1282 | COL4A1 | collagen, type IV, alpha 1 |
| 1490 | CTGF | connective tissue growth factor |
| 2192 | FBLN1 | fibulin 1 |
| 2200 | FBN1 | fibrillin 1 |
| 1404 | HAPLN1 | hyaluronan and proteoglycan link protein 1 |
| 1306 | COL15A1 | collagen, type XV, alpha 1 |
| 652 | BMP4 | bone morphogenetic protein 4 |
| 4982 | TNFRSF11B | tumor necrosis factor receptor superfamily, member 11b |
| 4015 | LOX | lysyl oxidase |
| 1842 | ECM2 | extracellular matrix protein 2, female organ and adipocyte specific |
| 10516 | FBLN5 | fibulin 5 |
| 1278 | COL1A2 | collagen, type I, alpha 2 |
| 1289 | COL5A1 | collagen, type V, alpha 1 |
| 5549 | PRELP | proline/arginine-rich end leucine-rich repeat protein |
| 176 | ACAN | aggrecan |
| 1303 | COL12A1 | collagen, type XII, alpha 1 |
| 1277 | COL1A1 | collagen, type I, alpha 1 |
| 3339 | HSPG2 | heparan sulfate proteoglycan 2 |
| 2202 | EFEMP1 | EGF-containing fibulin-like extracellular matrix protein 1 |
| 81578 | COL21A1 | collagen, type XXI, alpha 1 |
| 8532 | CPZ | carboxypeptidase Z |
| 1116 | CHI3L1 | chitinase 3-like 1 (cartilage glycoprotein-39) |
| 1281 | COL3A1 | collagen, type III, alpha 1 |
| 6678 | SPARC | secreted protein, acidic, cysteine-rich (osteonectin) |
| 127435 | PODN | podocan |
| 1634 | DCN | decorin |
| 1462 | VCAN | versican |
| 10082 | GPC6 | glypican 6 |
| 2331 | FMOD | fibromodulin |
| 3918 | LAMC2 | laminin, gamma 2 |
| 1290 | COL5A2 | collagen, type V, alpha 2 |
| 4256 | MGP | matrix Gla protein |

  
  
**MSCs batch 3 repressed and**GO:0044421**: 54 genes, expected 11.0828, P=2.21362e-22, P adjusted = 2.57692e-17

|  |  |  |
| --- | --- | --- |
| 6387 | CXCL12 | chemokine (C-X-C motif) ligand 12 (stromal cell-derived factor 1) |
| 10418 | SPON1 | spondin 1, extracellular matrix protein |
| 1301 | COL11A1 | collagen, type XI, alpha 1 |
| 2247 | FGF2 | fibroblast growth factor 2 (basic) |
| 8076 | MFAP5 | microfibrillar associated protein 5 |
| 4811 | NID1 | nidogen 1 |
| 11341 | SCRG1 | scrapie responsive protein 1 |
| 1284 | COL4A2 | collagen, type IV, alpha 2 |
| 1282 | COL4A1 | collagen, type IV, alpha 1 |
| 1490 | CTGF | connective tissue growth factor |
| 2192 | FBLN1 | fibulin 1 |
| 2200 | FBN1 | fibrillin 1 |
| 1404 | HAPLN1 | hyaluronan and proteoglycan link protein 1 |
| 1306 | COL15A1 | collagen, type XV, alpha 1 |
| 652 | BMP4 | bone morphogenetic protein 4 |
| 4982 | TNFRSF11B | tumor necrosis factor receptor superfamily, member 11b |
| 4015 | LOX | lysyl oxidase |
| 6424 | SFRP4 | secreted frizzled-related protein 4 |
| 1842 | ECM2 | extracellular matrix protein 2, female organ and adipocyte specific |
| 183 | AGT | angiotensinogen (serpin peptidase inhibitor, clade A, member 8) |
| 7412 | VCAM1 | vascular cell adhesion molecule 1 |
| 4653 | MYOC | myocilin, trabecular meshwork inducible glucocorticoid response |
| 10516 | FBLN5 | fibulin 5 |
| 55801 | IL26 | interleukin 26 |
| 1278 | COL1A2 | collagen, type I, alpha 2 |
| 1289 | COL5A1 | collagen, type V, alpha 1 |
| 3929 | LBP | lipopolysaccharide binding protein |
| 5549 | PRELP | proline/arginine-rich end leucine-rich repeat protein |
| 176 | ACAN | aggrecan |
| 1303 | COL12A1 | collagen, type XII, alpha 1 |
| 1277 | COL1A1 | collagen, type I, alpha 1 |
| 3339 | HSPG2 | heparan sulfate proteoglycan 2 |
| 2202 | EFEMP1 | EGF-containing fibulin-like extracellular matrix protein 1 |
| 10875 | FGL2 | fibrinogen-like 2 |
| 81578 | COL21A1 | collagen, type XXI, alpha 1 |
| 8532 | CPZ | carboxypeptidase Z |
| 6347 | CCL2 | chemokine (C-C motif) ligand 2 |
| 7049 | TGFBR3 | transforming growth factor, beta receptor III |
| 1116 | CHI3L1 | chitinase 3-like 1 (cartilage glycoprotein-39) |
| 1281 | COL3A1 | collagen, type III, alpha 1 |
| 6678 | SPARC | secreted protein, acidic, cysteine-rich (osteonectin) |
| 127435 | PODN | podocan |
| 1634 | DCN | decorin |
| 1462 | VCAN | versican |
| 3485 | IGFBP2 | insulin-like growth factor binding protein 2, 36kDa |
| 6372 | CXCL6 | chemokine (C-X-C motif) ligand 6 (granulocyte chemotactic protein 2) |
| 79875 | THSD4 | thrombospondin, type I, domain containing 4 |
| 10082 | GPC6 | glypican 6 |
| 2331 | FMOD | fibromodulin |
| 3918 | LAMC2 | laminin, gamma 2 |
| 1290 | COL5A2 | collagen, type V, alpha 2 |
| 4256 | MGP | matrix Gla protein |
| 23213 | SULF1 | sulfatase 1 |
| 1906 | EDN1 | endothelin 1 |

  
  
**MSCs batch 3 repressed and**GO:0044420**: 18 genes, expected 1.28974, P=5.41324e-16, P adjusted = 6.30166e-11

|  |  |  |
| --- | --- | --- |
| 1301 | COL11A1 | collagen, type XI, alpha 1 |
| 8076 | MFAP5 | microfibrillar associated protein 5 |
| 4811 | NID1 | nidogen 1 |
| 1284 | COL4A2 | collagen, type IV, alpha 2 |
| 1282 | COL4A1 | collagen, type IV, alpha 1 |
| 2200 | FBN1 | fibrillin 1 |
| 1306 | COL15A1 | collagen, type XV, alpha 1 |
| 4015 | LOX | lysyl oxidase |
| 1278 | COL1A2 | collagen, type I, alpha 2 |
| 1289 | COL5A1 | collagen, type V, alpha 1 |
| 176 | ACAN | aggrecan |
| 1303 | COL12A1 | collagen, type XII, alpha 1 |
| 1277 | COL1A1 | collagen, type I, alpha 1 |
| 3339 | HSPG2 | heparan sulfate proteoglycan 2 |
| 1281 | COL3A1 | collagen, type III, alpha 1 |
| 6678 | SPARC | secreted protein, acidic, cysteine-rich (osteonectin) |
| 3918 | LAMC2 | laminin, gamma 2 |
| 1290 | COL5A2 | collagen, type V, alpha 2 |

  
  
**MSCs batch 3 repressed and**GO:0005201**: 17 genes, expected 1.22976, P=4.22436e-15, P adjusted = 4.91766e-10

|  |  |  |
| --- | --- | --- |
| 1301 | COL11A1 | collagen, type XI, alpha 1 |
| 8076 | MFAP5 | microfibrillar associated protein 5 |
| 1284 | COL4A2 | collagen, type IV, alpha 2 |
| 1282 | COL4A1 | collagen, type IV, alpha 1 |
| 2192 | FBLN1 | fibulin 1 |
| 2200 | FBN1 | fibrillin 1 |
| 1306 | COL15A1 | collagen, type XV, alpha 1 |
| 1278 | COL1A2 | collagen, type I, alpha 2 |
| 1289 | COL5A1 | collagen, type V, alpha 1 |
| 5549 | PRELP | proline/arginine-rich end leucine-rich repeat protein |
| 176 | ACAN | aggrecan |
| 1303 | COL12A1 | collagen, type XII, alpha 1 |
| 1277 | COL1A1 | collagen, type I, alpha 1 |
| 1116 | CHI3L1 | chitinase 3-like 1 (cartilage glycoprotein-39) |
| 1281 | COL3A1 | collagen, type III, alpha 1 |
| 1290 | COL5A2 | collagen, type V, alpha 2 |
| 4256 | MGP | matrix Gla protein |

  
  
**MSCs batch 3 repressed and**GO:0048513**: 59 genes, expected 19.2112, P=7.38183e-15, P adjusted = 8.59334e-10

|  |  |  |
| --- | --- | --- |
| 6876 | TAGLN | transgelin |
| 1301 | COL11A1 | collagen, type XI, alpha 1 |
| 2247 | FGF2 | fibroblast growth factor 2 (basic) |
| 1948 | EFNB2 | ephrin-B2 |
| 1284 | COL4A2 | collagen, type IV, alpha 2 |
| 182 | JAG1 | jagged 1 (Alagille syndrome) |
| 1490 | CTGF | connective tissue growth factor |
| 2200 | FBN1 | fibrillin 1 |
| 388 | RHOB | ras homolog gene family, member B |
| 7538 | ZFP36 | zinc finger protein 36, C3H type, homolog (mouse) |
| 1306 | COL15A1 | collagen, type XV, alpha 1 |
| 652 | BMP4 | bone morphogenetic protein 4 |
| 2627 | GATA6 | GATA binding protein 6 |
| 7123 | CLEC3B | C-type lectin domain family 3, member B |
| 4982 | TNFRSF11B | tumor necrosis factor receptor superfamily, member 11b |
| 4015 | LOX | lysyl oxidase |
| 183 | AGT | angiotensinogen (serpin peptidase inhibitor, clade A, member 8) |
| 283078 | MKX | mohawk homeobox |
| 10468 | FST | follistatin |
| 7412 | VCAM1 | vascular cell adhesion molecule 1 |
| 284 | ANGPT1 | angiopoietin 1 |
| 8434 | RECK | reversion-inducing-cysteine-rich protein with kazal motifs |
| 1278 | COL1A2 | collagen, type I, alpha 2 |
| 5549 | PRELP | proline/arginine-rich end leucine-rich repeat protein |
| 176 | ACAN | aggrecan |
| 1303 | COL12A1 | collagen, type XII, alpha 1 |
| 9201 | DCLK1 | doublecortin-like kinase 1 |
| 1277 | COL1A1 | collagen, type I, alpha 1 |
| 4883 | NPR3 | natriuretic peptide receptor C/guanylate cyclase C (atrionatriuretic peptide receptor C) |
| 4232 | MEST | mesoderm specific transcript homolog (mouse) |
| 6347 | CCL2 | chemokine (C-C motif) ligand 2 |
| 8613 | PPAP2B | phosphatidic acid phosphatase type 2B |
| 5054 | SERPINE1 | serpin peptidase inhibitor, clade E (nexin, plasminogen activator inhibitor type 1), member 1 |
| 7049 | TGFBR3 | transforming growth factor, beta receptor III |
| 3861 | KRT14 | keratin 14 |
| 84623 | KIRREL3 | kin of IRRE like 3 (Drosophila) |
| 6444 | SGCD | sarcoglycan, delta (35kDa dystrophin-associated glycoprotein) |
| 1281 | COL3A1 | collagen, type III, alpha 1 |
| 2273 | FHL1 | four and a half LIM domains 1 |
| 6678 | SPARC | secreted protein, acidic, cysteine-rich (osteonectin) |
| 56956 | LHX9 | LIM homeobox 9 |
| 2619 | GAS1 | growth arrest-specific 1 |
| 1756 | DMD | dystrophin |
| 1634 | DCN | decorin |
| 83439 | TCF7L1 | transcription factor 7-like 1 (T-cell specific, HMG-box) |
| 1462 | VCAN | versican |
| 130497 | OSR1 | odd-skipped related 1 (Drosophila) |
| 7357 | UGCG | UDP-glucose ceramide glucosyltransferase |
| 2252 | FGF7 | fibroblast growth factor 7 (keratinocyte growth factor) |
| 3575 | IL7R | interleukin 7 receptor |
| 2131 | EXT1 | exostoses (multiple) 1 |
| 3381 | IBSP | integrin-binding sialoprotein |
| 7046 | TGFBR1 | transforming growth factor, beta receptor 1 |
| 3918 | LAMC2 | laminin, gamma 2 |
| 1290 | COL5A2 | collagen, type V, alpha 2 |
| 4256 | MGP | matrix Gla protein |
| 22801 | ITGA11 | integrin, alpha 11 |
| 1906 | EDN1 | endothelin 1 |
| 3885 | KRT34 | keratin 34 |

  
  
**MSCs batch 3 repressed and**GO:0048731**: 67 genes, expected 25.7349, P=2.21107e-13, P adjusted = 2.57395e-08

|  |  |  |
| --- | --- | --- |
| 6876 | TAGLN | transgelin |
| 1301 | COL11A1 | collagen, type XI, alpha 1 |
| 2247 | FGF2 | fibroblast growth factor 2 (basic) |
| 1948 | EFNB2 | ephrin-B2 |
| 11341 | SCRG1 | scrapie responsive protein 1 |
| 1284 | COL4A2 | collagen, type IV, alpha 2 |
| 182 | JAG1 | jagged 1 (Alagille syndrome) |
| 1490 | CTGF | connective tissue growth factor |
| 2200 | FBN1 | fibrillin 1 |
| 388 | RHOB | ras homolog gene family, member B |
| 7538 | ZFP36 | zinc finger protein 36, C3H type, homolog (mouse) |
| 1306 | COL15A1 | collagen, type XV, alpha 1 |
| 2824 | GPM6B | glycoprotein M6B |
| 652 | BMP4 | bone morphogenetic protein 4 |
| 2627 | GATA6 | GATA binding protein 6 |
| 7123 | CLEC3B | C-type lectin domain family 3, member B |
| 4982 | TNFRSF11B | tumor necrosis factor receptor superfamily, member 11b |
| 4015 | LOX | lysyl oxidase |
| 51232 | CRIM1 | cysteine rich transmembrane BMP regulator 1 (chordin-like) |
| 183 | AGT | angiotensinogen (serpin peptidase inhibitor, clade A, member 8) |
| 283078 | MKX | mohawk homeobox |
| 10468 | FST | follistatin |
| 7412 | VCAM1 | vascular cell adhesion molecule 1 |
| 284 | ANGPT1 | angiopoietin 1 |
| 8434 | RECK | reversion-inducing-cysteine-rich protein with kazal motifs |
| 1278 | COL1A2 | collagen, type I, alpha 2 |
| 5549 | PRELP | proline/arginine-rich end leucine-rich repeat protein |
| 176 | ACAN | aggrecan |
| 1303 | COL12A1 | collagen, type XII, alpha 1 |
| 9201 | DCLK1 | doublecortin-like kinase 1 |
| 1277 | COL1A1 | collagen, type I, alpha 1 |
| 4883 | NPR3 | natriuretic peptide receptor C/guanylate cyclase C (atrionatriuretic peptide receptor C) |
| 4232 | MEST | mesoderm specific transcript homolog (mouse) |
| 6347 | CCL2 | chemokine (C-C motif) ligand 2 |
| 8613 | PPAP2B | phosphatidic acid phosphatase type 2B |
| 5054 | SERPINE1 | serpin peptidase inhibitor, clade E (nexin, plasminogen activator inhibitor type 1), member 1 |
| 7049 | TGFBR3 | transforming growth factor, beta receptor III |
| 3861 | KRT14 | keratin 14 |
| 84623 | KIRREL3 | kin of IRRE like 3 (Drosophila) |
| 6444 | SGCD | sarcoglycan, delta (35kDa dystrophin-associated glycoprotein) |
| 1281 | COL3A1 | collagen, type III, alpha 1 |
| 2273 | FHL1 | four and a half LIM domains 1 |
| 6678 | SPARC | secreted protein, acidic, cysteine-rich (osteonectin) |
| 56956 | LHX9 | LIM homeobox 9 |
| 4803 | NGF | nerve growth factor (beta polypeptide) |
| 2619 | GAS1 | growth arrest-specific 1 |
| 9369 | NRXN3 | neurexin 3 |
| 1756 | DMD | dystrophin |
| 1634 | DCN | decorin |
| 83439 | TCF7L1 | transcription factor 7-like 1 (T-cell specific, HMG-box) |
| 288 | ANK3 | ankyrin 3, node of Ranvier (ankyrin G) |
| 1462 | VCAN | versican |
| 130497 | OSR1 | odd-skipped related 1 (Drosophila) |
| 7357 | UGCG | UDP-glucose ceramide glucosyltransferase |
| 2252 | FGF7 | fibroblast growth factor 7 (keratinocyte growth factor) |
| 3575 | IL7R | interleukin 7 receptor |
| 2353 | FOS | v-fos FBJ murine osteosarcoma viral oncogene homolog |
| 2131 | EXT1 | exostoses (multiple) 1 |
| 3381 | IBSP | integrin-binding sialoprotein |
| 7046 | TGFBR1 | transforming growth factor, beta receptor 1 |
| 3918 | LAMC2 | laminin, gamma 2 |
| 1290 | COL5A2 | collagen, type V, alpha 2 |
| 4256 | MGP | matrix Gla protein |
| 9915 | ARNT2 | aryl-hydrocarbon receptor nuclear translocator 2 |
| 22801 | ITGA11 | integrin, alpha 11 |
| 1906 | EDN1 | endothelin 1 |
| 3885 | KRT34 | keratin 34 |

  
  
**MSCs batch 3 repressed and**GO:0001871**: 18 genes, expected 1.82964, P=3.27112e-13, P adjusted = 3.80798e-08

|  |  |  |
| --- | --- | --- |
| 5197 | PF4V1 | platelet factor 4 variant 1 |
| 2247 | FGF2 | fibroblast growth factor 2 (basic) |
| 7057 | THBS1 | thrombospondin 1 |
| 1490 | CTGF | connective tissue growth factor |
| 1404 | HAPLN1 | hyaluronan and proteoglycan link protein 1 |
| 10894 | LYVE1 | lymphatic vessel endothelial hyaluronan receptor 1 |
| 7058 | THBS2 | thrombospondin 2 |
| 652 | BMP4 | bone morphogenetic protein 4 |
| 1289 | COL5A1 | collagen, type V, alpha 1 |
| 3929 | LBP | lipopolysaccharide binding protein |
| 176 | ACAN | aggrecan |
| 7049 | TGFBR3 | transforming growth factor, beta receptor III |
| 5806 | PTX3 | pentraxin-related gene, rapidly induced by IL-1 beta |
| 1462 | VCAN | versican |
| 6372 | CXCL6 | chemokine (C-X-C motif) ligand 6 (granulocyte chemotactic protein 2) |
| 143903 | LAYN | layilin |
| 2263 | FGFR2 | fibroblast growth factor receptor 2 |
| 3918 | LAMC2 | laminin, gamma 2 |

  
  
**MSCs batch 3 repressed and**GO:0030247**: 17 genes, expected 1.60468, P=4.32034e-13, P adjusted = 5.02939e-08

|  |  |  |
| --- | --- | --- |
| 5197 | PF4V1 | platelet factor 4 variant 1 |
| 2247 | FGF2 | fibroblast growth factor 2 (basic) |
| 7057 | THBS1 | thrombospondin 1 |
| 1490 | CTGF | connective tissue growth factor |
| 1404 | HAPLN1 | hyaluronan and proteoglycan link protein 1 |
| 10894 | LYVE1 | lymphatic vessel endothelial hyaluronan receptor 1 |
| 7058 | THBS2 | thrombospondin 2 |
| 652 | BMP4 | bone morphogenetic protein 4 |
| 1289 | COL5A1 | collagen, type V, alpha 1 |
| 176 | ACAN | aggrecan |
| 7049 | TGFBR3 | transforming growth factor, beta receptor III |
| 5806 | PTX3 | pentraxin-related gene, rapidly induced by IL-1 beta |
| 1462 | VCAN | versican |
| 6372 | CXCL6 | chemokine (C-X-C motif) ligand 6 (granulocyte chemotactic protein 2) |
| 143903 | LAYN | layilin |
| 2263 | FGFR2 | fibroblast growth factor receptor 2 |
| 3918 | LAMC2 | laminin, gamma 2 |

  
  
**MSCs batch 3 repressed and**GO:0007155**: 40 genes, expected 10.8129, P=9.4332e-13, P adjusted = 1.09814e-07

|  |  |  |
| --- | --- | --- |
| 6387 | CXCL12 | chemokine (C-X-C motif) ligand 12 (stromal cell-derived factor 1) |
| 10418 | SPON1 | spondin 1, extracellular matrix protein |
| 1301 | COL11A1 | collagen, type XI, alpha 1 |
| 4162 | MCAM | melanoma cell adhesion molecule |
| 4811 | NID1 | nidogen 1 |
| 7057 | THBS1 | thrombospondin 1 |
| 58494 | JAM2 | junctional adhesion molecule 2 |
| 1490 | CTGF | connective tissue growth factor |
| 388 | RHOB | ras homolog gene family, member B |
| 1404 | HAPLN1 | hyaluronan and proteoglycan link protein 1 |
| 1306 | COL15A1 | collagen, type XV, alpha 1 |
| 10894 | LYVE1 | lymphatic vessel endothelial hyaluronan receptor 1 |
| 7058 | THBS2 | thrombospondin 2 |
| 8839 | WISP2 | WNT1 inducible signaling pathway protein 2 |
| 9358 | ITGBL1 | integrin, beta-like 1 (with EGF-like repeat domains) |
| 1842 | ECM2 | extracellular matrix protein 2, female organ and adipocyte specific |
| 183 | AGT | angiotensinogen (serpin peptidase inhibitor, clade A, member 8) |
| 7412 | VCAM1 | vascular cell adhesion molecule 1 |
| 10516 | FBLN5 | fibulin 5 |
| 1289 | COL5A1 | collagen, type V, alpha 1 |
| 176 | ACAN | aggrecan |
| 1303 | COL12A1 | collagen, type XII, alpha 1 |
| 3339 | HSPG2 | heparan sulfate proteoglycan 2 |
| 81578 | COL21A1 | collagen, type XXI, alpha 1 |
| 9635 | CLCA2 | CLCA family member 2, chloride channel regulator |
| 6347 | CCL2 | chemokine (C-C motif) ligand 2 |
| 7049 | TGFBR3 | transforming growth factor, beta receptor III |
| 79987 | SVEP1 | sushi, von Willebrand factor type A, EGF and pentraxin domain containing 1 |
| 1281 | COL3A1 | collagen, type III, alpha 1 |
| 5792 | PTPRF | protein tyrosine phosphatase, receptor type, F |
| 9369 | NRXN3 | neurexin 3 |
| 1462 | VCAN | versican |
| 57575 | PCDH10 | protocadherin 10 |
| 3696 | ITGB8 | integrin, beta 8 |
| 165 | AEBP1 | AE binding protein 1 |
| 3381 | IBSP | integrin-binding sialoprotein |
| 3918 | LAMC2 | laminin, gamma 2 |
| 4256 | MGP | matrix Gla protein |
| 22801 | ITGA11 | integrin, alpha 11 |
| 4739 | NEDD9 | neural precursor cell expressed, developmentally down-regulated 9 |

  
  
**MSCs batch 3 repressed and**GO:0022610**: 40 genes, expected 10.8129, P=9.4332e-13, P adjusted = 1.09814e-07

|  |  |  |
| --- | --- | --- |
| 6387 | CXCL12 | chemokine (C-X-C motif) ligand 12 (stromal cell-derived factor 1) |
| 10418 | SPON1 | spondin 1, extracellular matrix protein |
| 1301 | COL11A1 | collagen, type XI, alpha 1 |
| 4162 | MCAM | melanoma cell adhesion molecule |
| 4811 | NID1 | nidogen 1 |
| 7057 | THBS1 | thrombospondin 1 |
| 58494 | JAM2 | junctional adhesion molecule 2 |
| 1490 | CTGF | connective tissue growth factor |
| 388 | RHOB | ras homolog gene family, member B |
| 1404 | HAPLN1 | hyaluronan and proteoglycan link protein 1 |
| 1306 | COL15A1 | collagen, type XV, alpha 1 |
| 10894 | LYVE1 | lymphatic vessel endothelial hyaluronan receptor 1 |
| 7058 | THBS2 | thrombospondin 2 |
| 8839 | WISP2 | WNT1 inducible signaling pathway protein 2 |
| 9358 | ITGBL1 | integrin, beta-like 1 (with EGF-like repeat domains) |
| 1842 | ECM2 | extracellular matrix protein 2, female organ and adipocyte specific |
| 183 | AGT | angiotensinogen (serpin peptidase inhibitor, clade A, member 8) |
| 7412 | VCAM1 | vascular cell adhesion molecule 1 |
| 10516 | FBLN5 | fibulin 5 |
| 1289 | COL5A1 | collagen, type V, alpha 1 |
| 176 | ACAN | aggrecan |
| 1303 | COL12A1 | collagen, type XII, alpha 1 |
| 3339 | HSPG2 | heparan sulfate proteoglycan 2 |
| 81578 | COL21A1 | collagen, type XXI, alpha 1 |
| 9635 | CLCA2 | CLCA family member 2, chloride channel regulator |
| 6347 | CCL2 | chemokine (C-C motif) ligand 2 |
| 7049 | TGFBR3 | transforming growth factor, beta receptor III |
| 79987 | SVEP1 | sushi, von Willebrand factor type A, EGF and pentraxin domain containing 1 |
| 1281 | COL3A1 | collagen, type III, alpha 1 |
| 5792 | PTPRF | protein tyrosine phosphatase, receptor type, F |
| 9369 | NRXN3 | neurexin 3 |
| 1462 | VCAN | versican |
| 57575 | PCDH10 | protocadherin 10 |
| 3696 | ITGB8 | integrin, beta 8 |
| 165 | AEBP1 | AE binding protein 1 |
| 3381 | IBSP | integrin-binding sialoprotein |
| 3918 | LAMC2 | laminin, gamma 2 |
| 4256 | MGP | matrix Gla protein |
| 22801 | ITGA11 | integrin, alpha 11 |
| 4739 | NEDD9 | neural precursor cell expressed, developmentally down-regulated 9 |

  
  
**MSCs batch 3 repressed and**GO:0005581**: 11 genes, expected 0.524896, P=2.18196e-12, P adjusted = 2.54006e-07

|  |  |  |
| --- | --- | --- |
| 1301 | COL11A1 | collagen, type XI, alpha 1 |
| 1284 | COL4A2 | collagen, type IV, alpha 2 |
| 1282 | COL4A1 | collagen, type IV, alpha 1 |
| 1306 | COL15A1 | collagen, type XV, alpha 1 |
| 4015 | LOX | lysyl oxidase |
| 1278 | COL1A2 | collagen, type I, alpha 2 |
| 1289 | COL5A1 | collagen, type V, alpha 1 |
| 1303 | COL12A1 | collagen, type XII, alpha 1 |
| 1277 | COL1A1 | collagen, type I, alpha 1 |
| 1281 | COL3A1 | collagen, type III, alpha 1 |
| 1290 | COL5A2 | collagen, type V, alpha 2 |

  
  
**MSCs batch 3 repressed and**GO:0005539**: 16 genes, expected 1.54469, P=3.06246e-12, P adjusted = 3.56508e-07

|  |  |  |
| --- | --- | --- |
| 5197 | PF4V1 | platelet factor 4 variant 1 |
| 2247 | FGF2 | fibroblast growth factor 2 (basic) |
| 7057 | THBS1 | thrombospondin 1 |
| 1490 | CTGF | connective tissue growth factor |
| 1404 | HAPLN1 | hyaluronan and proteoglycan link protein 1 |
| 10894 | LYVE1 | lymphatic vessel endothelial hyaluronan receptor 1 |
| 7058 | THBS2 | thrombospondin 2 |
| 652 | BMP4 | bone morphogenetic protein 4 |
| 1289 | COL5A1 | collagen, type V, alpha 1 |
| 176 | ACAN | aggrecan |
| 7049 | TGFBR3 | transforming growth factor, beta receptor III |
| 1462 | VCAN | versican |
| 6372 | CXCL6 | chemokine (C-X-C motif) ligand 6 (granulocyte chemotactic protein 2) |
| 143903 | LAYN | layilin |
| 2263 | FGFR2 | fibroblast growth factor receptor 2 |
| 3918 | LAMC2 | laminin, gamma 2 |

  
  
**MSCs batch 3 repressed and**GO:0005198**: 34 genes, expected 9.71808, P=2.43099e-10, P adjusted = 2.82997e-05

|  |  |  |
| --- | --- | --- |
| 1301 | COL11A1 | collagen, type XI, alpha 1 |
| 3884 | KRT33B | keratin 33B |
| 8076 | MFAP5 | microfibrillar associated protein 5 |
| 7057 | THBS1 | thrombospondin 1 |
| 1284 | COL4A2 | collagen, type IV, alpha 2 |
| 1282 | COL4A1 | collagen, type IV, alpha 1 |
| 182 | JAG1 | jagged 1 (Alagille syndrome) |
| 2192 | FBLN1 | fibulin 1 |
| 2200 | FBN1 | fibrillin 1 |
| 1306 | COL15A1 | collagen, type XV, alpha 1 |
| 7058 | THBS2 | thrombospondin 2 |
| 59 | ACTA2 | actin, alpha 2, smooth muscle, aorta |
| 3883 | KRT33A | keratin 33A |
| 4653 | MYOC | myocilin, trabecular meshwork inducible glucocorticoid response |
| 1278 | COL1A2 | collagen, type I, alpha 2 |
| 1289 | COL5A1 | collagen, type V, alpha 1 |
| 2312 | FLG | filaggrin |
| 5549 | PRELP | proline/arginine-rich end leucine-rich repeat protein |
| 176 | ACAN | aggrecan |
| 1303 | COL12A1 | collagen, type XII, alpha 1 |
| 1277 | COL1A1 | collagen, type I, alpha 1 |
| 81578 | COL21A1 | collagen, type XXI, alpha 1 |
| 3875 | KRT18 | keratin 18 |
| 7168 | TPM1 | tropomyosin 1 (alpha) |
| 3861 | KRT14 | keratin 14 |
| 1116 | CHI3L1 | chitinase 3-like 1 (cartilage glycoprotein-39) |
| 1281 | COL3A1 | collagen, type III, alpha 1 |
| 3892 | KRT86 | keratin 86 |
| 3855 | KRT7 | keratin 7 |
| 1756 | DMD | dystrophin |
| 1290 | COL5A2 | collagen, type V, alpha 2 |
| 4256 | MGP | matrix Gla protein |
| 10398 | MYL9 | myosin, light chain 9, regulatory |
| 3885 | KRT34 | keratin 34 |

  
  
**MSCs batch 3 repressed and**GO:0001501**: 16 genes, expected 2.47451, P=4.02851e-09, P adjusted = 0.000468967

|  |  |  |
| --- | --- | --- |
| 1301 | COL11A1 | collagen, type XI, alpha 1 |
| 1490 | CTGF | connective tissue growth factor |
| 2200 | FBN1 | fibrillin 1 |
| 652 | BMP4 | bone morphogenetic protein 4 |
| 7123 | CLEC3B | C-type lectin domain family 3, member B |
| 4982 | TNFRSF11B | tumor necrosis factor receptor superfamily, member 11b |
| 1278 | COL1A2 | collagen, type I, alpha 2 |
| 5549 | PRELP | proline/arginine-rich end leucine-rich repeat protein |
| 176 | ACAN | aggrecan |
| 1303 | COL12A1 | collagen, type XII, alpha 1 |
| 1277 | COL1A1 | collagen, type I, alpha 1 |
| 4883 | NPR3 | natriuretic peptide receptor C/guanylate cyclase C (atrionatriuretic peptide receptor C) |
| 2131 | EXT1 | exostoses (multiple) 1 |
| 7046 | TGFBR1 | transforming growth factor, beta receptor 1 |
| 4256 | MGP | matrix Gla protein |
| 1906 | EDN1 | endothelin 1 |

  
  
**MSCs batch 3 repressed and**GO:0009888**: 22 genes, expected 4.90403, P=5.1573e-09, P adjusted = 0.000600371

|  |  |  |
| --- | --- | --- |
| 1301 | COL11A1 | collagen, type XI, alpha 1 |
| 182 | JAG1 | jagged 1 (Alagille syndrome) |
| 1490 | CTGF | connective tissue growth factor |
| 652 | BMP4 | bone morphogenetic protein 4 |
| 10468 | FST | follistatin |
| 176 | ACAN | aggrecan |
| 1277 | COL1A1 | collagen, type I, alpha 1 |
| 4232 | MEST | mesoderm specific transcript homolog (mouse) |
| 3861 | KRT14 | keratin 14 |
| 1281 | COL3A1 | collagen, type III, alpha 1 |
| 6678 | SPARC | secreted protein, acidic, cysteine-rich (osteonectin) |
| 83439 | TCF7L1 | transcription factor 7-like 1 (T-cell specific, HMG-box) |
| 130497 | OSR1 | odd-skipped related 1 (Drosophila) |
| 7357 | UGCG | UDP-glucose ceramide glucosyltransferase |
| 2252 | FGF7 | fibroblast growth factor 7 (keratinocyte growth factor) |
| 2131 | EXT1 | exostoses (multiple) 1 |
| 3381 | IBSP | integrin-binding sialoprotein |
| 3918 | LAMC2 | laminin, gamma 2 |
| 1290 | COL5A2 | collagen, type V, alpha 2 |
| 4256 | MGP | matrix Gla protein |
| 1906 | EDN1 | endothelin 1 |
| 3885 | KRT34 | keratin 34 |

  
  
**MSCs batch 3 repressed and**GO:0030246**: 20 genes, expected 4.15418, P=7.96566e-09, P adjusted = 0.000927298

|  |  |  |
| --- | --- | --- |
| 5197 | PF4V1 | platelet factor 4 variant 1 |
| 2247 | FGF2 | fibroblast growth factor 2 (basic) |
| 7057 | THBS1 | thrombospondin 1 |
| 1490 | CTGF | connective tissue growth factor |
| 1404 | HAPLN1 | hyaluronan and proteoglycan link protein 1 |
| 10894 | LYVE1 | lymphatic vessel endothelial hyaluronan receptor 1 |
| 7058 | THBS2 | thrombospondin 2 |
| 652 | BMP4 | bone morphogenetic protein 4 |
| 7123 | CLEC3B | C-type lectin domain family 3, member B |
| 5648 | MASP1 | mannan-binding lectin serine peptidase 1 (C4/C2 activating component of Ra-reactive factor) |
| 1289 | COL5A1 | collagen, type V, alpha 1 |
| 176 | ACAN | aggrecan |
| 7049 | TGFBR3 | transforming growth factor, beta receptor III |
| 1116 | CHI3L1 | chitinase 3-like 1 (cartilage glycoprotein-39) |
| 5806 | PTX3 | pentraxin-related gene, rapidly induced by IL-1 beta |
| 1462 | VCAN | versican |
| 6372 | CXCL6 | chemokine (C-X-C motif) ligand 6 (granulocyte chemotactic protein 2) |
| 143903 | LAYN | layilin |
| 2263 | FGFR2 | fibroblast growth factor receptor 2 |
| 3918 | LAMC2 | laminin, gamma 2 |

  
  
**MSCs batch 3 repressed and**GO:0030198**: 10 genes, expected 0.839834, P=9.60568e-09, P adjusted = 0.00111822

|  |  |  |
| --- | --- | --- |
| 1301 | COL11A1 | collagen, type XI, alpha 1 |
| 1284 | COL4A2 | collagen, type IV, alpha 2 |
| 4015 | LOX | lysyl oxidase |
| 183 | AGT | angiotensinogen (serpin peptidase inhibitor, clade A, member 8) |
| 8434 | RECK | reversion-inducing-cysteine-rich protein with kazal motifs |
| 176 | ACAN | aggrecan |
| 1303 | COL12A1 | collagen, type XII, alpha 1 |
| 1281 | COL3A1 | collagen, type III, alpha 1 |
| 7046 | TGFBR1 | transforming growth factor, beta receptor 1 |
| 1290 | COL5A2 | collagen, type V, alpha 2 |

  
  
**MSCs batch 3 repressed and**GO:0030199**: 7 genes, expected 0.299941, P=1.04421e-08, P adjusted = 0.00121559

|  |  |  |
| --- | --- | --- |
| 1301 | COL11A1 | collagen, type XI, alpha 1 |
| 4015 | LOX | lysyl oxidase |
| 176 | ACAN | aggrecan |
| 1303 | COL12A1 | collagen, type XII, alpha 1 |
| 1281 | COL3A1 | collagen, type III, alpha 1 |
| 7046 | TGFBR1 | transforming growth factor, beta receptor 1 |
| 1290 | COL5A2 | collagen, type V, alpha 2 |

  
  
**MSCs batch 3 repressed and**GO:0008201**: 11 genes, expected 1.21476, P=3.43722e-08, P adjusted = 0.00400134

|  |  |  |
| --- | --- | --- |
| 5197 | PF4V1 | platelet factor 4 variant 1 |
| 2247 | FGF2 | fibroblast growth factor 2 (basic) |
| 7057 | THBS1 | thrombospondin 1 |
| 1490 | CTGF | connective tissue growth factor |
| 7058 | THBS2 | thrombospondin 2 |
| 652 | BMP4 | bone morphogenetic protein 4 |
| 1289 | COL5A1 | collagen, type V, alpha 1 |
| 7049 | TGFBR3 | transforming growth factor, beta receptor III |
| 6372 | CXCL6 | chemokine (C-X-C motif) ligand 6 (granulocyte chemotactic protein 2) |
| 2263 | FGFR2 | fibroblast growth factor receptor 2 |
| 3918 | LAMC2 | laminin, gamma 2 |

  
  
**MSCs batch 3 repressed and**GO:0001944**: 16 genes, expected 2.99941, P=6.25196e-08, P adjusted = 0.00727803

|  |  |  |
| --- | --- | --- |
| 2247 | FGF2 | fibroblast growth factor 2 (basic) |
| 1948 | EFNB2 | ephrin-B2 |
| 1284 | COL4A2 | collagen, type IV, alpha 2 |
| 182 | JAG1 | jagged 1 (Alagille syndrome) |
| 1490 | CTGF | connective tissue growth factor |
| 388 | RHOB | ras homolog gene family, member B |
| 1306 | COL15A1 | collagen, type XV, alpha 1 |
| 652 | BMP4 | bone morphogenetic protein 4 |
| 4015 | LOX | lysyl oxidase |
| 183 | AGT | angiotensinogen (serpin peptidase inhibitor, clade A, member 8) |
| 284 | ANGPT1 | angiopoietin 1 |
| 8434 | RECK | reversion-inducing-cysteine-rich protein with kazal motifs |
| 8613 | PPAP2B | phosphatidic acid phosphatase type 2B |
| 5054 | SERPINE1 | serpin peptidase inhibitor, clade E (nexin, plasminogen activator inhibitor type 1), member 1 |
| 7046 | TGFBR1 | transforming growth factor, beta receptor 1 |
| 1906 | EDN1 | endothelin 1 |

  
  
**MSCs batch 3 repressed and**GO:0005583**: 5 genes, expected 0.134973, P=8.80496e-08, P adjusted = 0.01025

|  |  |  |
| --- | --- | --- |
| 1301 | COL11A1 | collagen, type XI, alpha 1 |
| 1278 | COL1A2 | collagen, type I, alpha 2 |
| 1289 | COL5A1 | collagen, type V, alpha 1 |
| 1281 | COL3A1 | collagen, type III, alpha 1 |
| 1290 | COL5A2 | collagen, type V, alpha 2 |

  
  
**MSCs batch 3 repressed and**GO:0009611**: 23 genes, expected 6.28376, P=1.00396e-07, P adjusted = 0.0116873

|  |  |  |
| --- | --- | --- |
| 57088 | PLSCR4 | phospholipid scramblase 4 |
| 1490 | CTGF | connective tissue growth factor |
| 2200 | FBN1 | fibrillin 1 |
| 7538 | ZFP36 | zinc finger protein 36, C3H type, homolog (mouse) |
| 629 | CFB | complement factor B |
| 10894 | LYVE1 | lymphatic vessel endothelial hyaluronan receptor 1 |
| 183 | AGT | angiotensinogen (serpin peptidase inhibitor, clade A, member 8) |
| 2687 | GGT5 | gamma-glutamyltransferase 5 |
| 5648 | MASP1 | mannan-binding lectin serine peptidase 1 (C4/C2 activating component of Ra-reactive factor) |
| 10516 | FBLN5 | fibulin 5 |
| 12 | SERPINA3 | serpin peptidase inhibitor, clade A (alpha-1 antiproteinase, antitrypsin), member 3 |
| 3929 | LBP | lipopolysaccharide binding protein |
| 6347 | CCL2 | chemokine (C-C motif) ligand 2 |
| 5054 | SERPINE1 | serpin peptidase inhibitor, clade E (nexin, plasminogen activator inhibitor type 1), member 1 |
| 5806 | PTX3 | pentraxin-related gene, rapidly induced by IL-1 beta |
| 1281 | COL3A1 | collagen, type III, alpha 1 |
| 718 | C3 | complement component 3 |
| 716 | C1S | complement component 1, s subcomponent |
| 2252 | FGF7 | fibroblast growth factor 7 (keratinocyte growth factor) |
| 6372 | CXCL6 | chemokine (C-X-C motif) ligand 6 (granulocyte chemotactic protein 2) |
| 2353 | FOS | v-fos FBJ murine osteosarcoma viral oncogene homolog |
| 715 | C1R | complement component 1, r subcomponent |
| 1906 | EDN1 | endothelin 1 |

  
  
**MSCs batch 3 repressed and**GO:0009605**: 29 genes, expected 9.53811, P=1.17027e-07, P adjusted = 0.0136233

|  |  |  |
| --- | --- | --- |
| 6387 | CXCL12 | chemokine (C-X-C motif) ligand 12 (stromal cell-derived factor 1) |
| 51554 | CCRL1 | chemokine (C-C motif) receptor-like 1 |
| 2247 | FGF2 | fibroblast growth factor 2 (basic) |
| 57088 | PLSCR4 | phospholipid scramblase 4 |
| 1490 | CTGF | connective tissue growth factor |
| 2200 | FBN1 | fibrillin 1 |
| 7538 | ZFP36 | zinc finger protein 36, C3H type, homolog (mouse) |
| 629 | CFB | complement factor B |
| 10894 | LYVE1 | lymphatic vessel endothelial hyaluronan receptor 1 |
| 183 | AGT | angiotensinogen (serpin peptidase inhibitor, clade A, member 8) |
| 2687 | GGT5 | gamma-glutamyltransferase 5 |
| 5648 | MASP1 | mannan-binding lectin serine peptidase 1 (C4/C2 activating component of Ra-reactive factor) |
| 10516 | FBLN5 | fibulin 5 |
| 12 | SERPINA3 | serpin peptidase inhibitor, clade A (alpha-1 antiproteinase, antitrypsin), member 3 |
| 3929 | LBP | lipopolysaccharide binding protein |
| 6751 | SSTR1 | somatostatin receptor 1 |
| 5168 | ENPP2 | ectonucleotide pyrophosphatase/phosphodiesterase 2 |
| 6347 | CCL2 | chemokine (C-C motif) ligand 2 |
| 5054 | SERPINE1 | serpin peptidase inhibitor, clade E (nexin, plasminogen activator inhibitor type 1), member 1 |
| 5806 | PTX3 | pentraxin-related gene, rapidly induced by IL-1 beta |
| 1281 | COL3A1 | collagen, type III, alpha 1 |
| 718 | C3 | complement component 3 |
| 716 | C1S | complement component 1, s subcomponent |
| 2252 | FGF7 | fibroblast growth factor 7 (keratinocyte growth factor) |
| 6372 | CXCL6 | chemokine (C-X-C motif) ligand 6 (granulocyte chemotactic protein 2) |
| 84557 | MAP1LC3A | microtubule-associated protein 1 light chain 3 alpha |
| 2353 | FOS | v-fos FBJ murine osteosarcoma viral oncogene homolog |
| 715 | C1R | complement component 1, r subcomponent |
| 1906 | EDN1 | endothelin 1 |

  
  
**MSCs batch 3 repressed and**GO:0001568**: 15 genes, expected 2.93942, P=2.89008e-07, P adjusted = 0.033644

|  |  |  |
| --- | --- | --- |
| 2247 | FGF2 | fibroblast growth factor 2 (basic) |
| 1284 | COL4A2 | collagen, type IV, alpha 2 |
| 182 | JAG1 | jagged 1 (Alagille syndrome) |
| 1490 | CTGF | connective tissue growth factor |
| 388 | RHOB | ras homolog gene family, member B |
| 1306 | COL15A1 | collagen, type XV, alpha 1 |
| 652 | BMP4 | bone morphogenetic protein 4 |
| 4015 | LOX | lysyl oxidase |
| 183 | AGT | angiotensinogen (serpin peptidase inhibitor, clade A, member 8) |
| 284 | ANGPT1 | angiopoietin 1 |
| 8434 | RECK | reversion-inducing-cysteine-rich protein with kazal motifs |
| 8613 | PPAP2B | phosphatidic acid phosphatase type 2B |
| 5054 | SERPINE1 | serpin peptidase inhibitor, clade E (nexin, plasminogen activator inhibitor type 1), member 1 |
| 7046 | TGFBR1 | transforming growth factor, beta receptor 1 |
| 1906 | EDN1 | endothelin 1 |

  
  
**MSCs batch 3 repressed and**GO:0005102**: 31 genes, expected 11.2028, P=3.34319e-07, P adjusted = 0.0389187

|  |  |  |
| --- | --- | --- |
| 6387 | CXCL12 | chemokine (C-X-C motif) ligand 12 (stromal cell-derived factor 1) |
| 5197 | PF4V1 | platelet factor 4 variant 1 |
| 4582 | MUC1 | mucin 1, cell surface associated |
| 2247 | FGF2 | fibroblast growth factor 2 (basic) |
| 1948 | EFNB2 | ephrin-B2 |
| 182 | JAG1 | jagged 1 (Alagille syndrome) |
| 1490 | CTGF | connective tissue growth factor |
| 6281 | S100A10 | S100 calcium binding protein A10 |
| 652 | BMP4 | bone morphogenetic protein 4 |
| 4982 | TNFRSF11B | tumor necrosis factor receptor superfamily, member 11b |
| 1842 | ECM2 | extracellular matrix protein 2, female organ and adipocyte specific |
| 183 | AGT | angiotensinogen (serpin peptidase inhibitor, clade A, member 8) |
| 7412 | VCAM1 | vascular cell adhesion molecule 1 |
| 284 | ANGPT1 | angiopoietin 1 |
| 10516 | FBLN5 | fibulin 5 |
| 55801 | IL26 | interleukin 26 |
| 3929 | LBP | lipopolysaccharide binding protein |
| 10875 | FGL2 | fibrinogen-like 2 |
| 6347 | CCL2 | chemokine (C-C motif) ligand 2 |
| 7049 | TGFBR3 | transforming growth factor, beta receptor III |
| 1281 | COL3A1 | collagen, type III, alpha 1 |
| 4856 | NOV | nephroblastoma overexpressed gene |
| 4803 | NGF | nerve growth factor (beta polypeptide) |
| 654466 | KGFLP2 | keratinocyte growth factor-like protein 2 |
| 718 | C3 | complement component 3 |
| 2252 | FGF7 | fibroblast growth factor 7 (keratinocyte growth factor) |
| 6372 | CXCL6 | chemokine (C-X-C motif) ligand 6 (granulocyte chemotactic protein 2) |
| 7046 | TGFBR1 | transforming growth factor, beta receptor 1 |
| 9915 | ARNT2 | aryl-hydrocarbon receptor nuclear translocator 2 |
| 1906 | EDN1 | endothelin 1 |
| 80310 | PDGFD | platelet derived growth factor D |

  
  
**MSCs batch 3 repressed and**GO:0043062**: 11 genes, expected 1.5297, P=3.82117e-07, P adjusted = 0.044483

|  |  |  |
| --- | --- | --- |
| 1301 | COL11A1 | collagen, type XI, alpha 1 |
| 1284 | COL4A2 | collagen, type IV, alpha 2 |
| 4015 | LOX | lysyl oxidase |
| 183 | AGT | angiotensinogen (serpin peptidase inhibitor, clade A, member 8) |
| 8434 | RECK | reversion-inducing-cysteine-rich protein with kazal motifs |
| 176 | ACAN | aggrecan |
| 1303 | COL12A1 | collagen, type XII, alpha 1 |
| 1281 | COL3A1 | collagen, type III, alpha 1 |
| 288 | ANK3 | ankyrin 3, node of Ranvier (ankyrin G) |
| 7046 | TGFBR1 | transforming growth factor, beta receptor 1 |
| 1290 | COL5A2 | collagen, type V, alpha 2 |

  
  
**MSCs batch 3 repressed and**GO:0007160**: 10 genes, expected 1.28974, P=6.42512e-07, P adjusted = 0.0747961

|  |  |  |
| --- | --- | --- |
| 4811 | NID1 | nidogen 1 |
| 1490 | CTGF | connective tissue growth factor |
| 10894 | LYVE1 | lymphatic vessel endothelial hyaluronan receptor 1 |
| 9358 | ITGBL1 | integrin, beta-like 1 (with EGF-like repeat domains) |
| 1842 | ECM2 | extracellular matrix protein 2, female organ and adipocyte specific |
| 183 | AGT | angiotensinogen (serpin peptidase inhibitor, clade A, member 8) |
| 10516 | FBLN5 | fibulin 5 |
| 1281 | COL3A1 | collagen, type III, alpha 1 |
| 3696 | ITGB8 | integrin, beta 8 |
| 22801 | ITGA11 | integrin, alpha 11 |

  
  
**MSCs batch 3 repressed and**GO:0007166**: 46 genes, expected 21.3558, P=6.64912e-07, P adjusted = 0.0774038

|  |  |  |
| --- | --- | --- |
| 6387 | CXCL12 | chemokine (C-X-C motif) ligand 12 (stromal cell-derived factor 1) |
| 51554 | CCRL1 | chemokine (C-C motif) receptor-like 1 |
| 5021 | OXTR | oxytocin receptor |
| 2247 | FGF2 | fibroblast growth factor 2 (basic) |
| 182 | JAG1 | jagged 1 (Alagille syndrome) |
| 4919 | ROR1 | receptor tyrosine kinase-like orphan receptor 1 |
| 1490 | CTGF | connective tissue growth factor |
| 652 | BMP4 | bone morphogenetic protein 4 |
| 9358 | ITGBL1 | integrin, beta-like 1 (with EGF-like repeat domains) |
| 4015 | LOX | lysyl oxidase |
| 6424 | SFRP4 | secreted frizzled-related protein 4 |
| 183 | AGT | angiotensinogen (serpin peptidase inhibitor, clade A, member 8) |
| 10468 | FST | follistatin |
| 1278 | COL1A2 | collagen, type I, alpha 2 |
| 3929 | LBP | lipopolysaccharide binding protein |
| 9315 | C5orf13 | chromosome 5 open reading frame 13 |
| 1439 | CSF2RB | colony stimulating factor 2 receptor, beta, low-affinity (granulocyte-macrophage) |
| 64750 | SMURF2 | SMAD specific E3 ubiquitin protein ligase 2 |
| 6751 | SSTR1 | somatostatin receptor 1 |
| 150 | ADRA2A | adrenergic, alpha-2A-, receptor |
| 3875 | KRT18 | keratin 18 |
| 8532 | CPZ | carboxypeptidase Z |
| 80332 | ADAM33 | ADAM metallopeptidase domain 33 |
| 5168 | ENPP2 | ectonucleotide pyrophosphatase/phosphodiesterase 2 |
| 6347 | CCL2 | chemokine (C-C motif) ligand 2 |
| 8613 | PPAP2B | phosphatidic acid phosphatase type 2B |
| 7049 | TGFBR3 | transforming growth factor, beta receptor III |
| 9590 | AKAP12 | A kinase (PRKA) anchor protein 12 |
| 1281 | COL3A1 | collagen, type III, alpha 1 |
| 6678 | SPARC | secreted protein, acidic, cysteine-rich (osteonectin) |
| 5792 | PTPRF | protein tyrosine phosphatase, receptor type, F |
| 4803 | NGF | nerve growth factor (beta polypeptide) |
| 83439 | TCF7L1 | transcription factor 7-like 1 (T-cell specific, HMG-box) |
| 196883 | ADCY4 | adenylate cyclase 4 |
| 718 | C3 | complement component 3 |
| 2252 | FGF7 | fibroblast growth factor 7 (keratinocyte growth factor) |
| 9052 | GPRC5A | G protein-coupled receptor, family C, group 5, member A |
| 2263 | FGFR2 | fibroblast growth factor receptor 2 |
| 3575 | IL7R | interleukin 7 receptor |
| 3696 | ITGB8 | integrin, beta 8 |
| 7046 | TGFBR1 | transforming growth factor, beta receptor 1 |
| 2331 | FMOD | fibromodulin |
| 10149 | GPR64 | G protein-coupled receptor 64 |
| 22801 | ITGA11 | integrin, alpha 11 |
| 4739 | NEDD9 | neural precursor cell expressed, developmentally down-regulated 9 |
| 1906 | EDN1 | endothelin 1 |

  
  
**MSCs batch 3 repressed and**GO:0004867**: 10 genes, expected 1.31974, P=7.97685e-07, P adjusted = 0.0928602

|  |  |  |
| --- | --- | --- |
| 51232 | CRIM1 | cysteine rich transmembrane BMP regulator 1 (chordin-like) |
| 183 | AGT | angiotensinogen (serpin peptidase inhibitor, clade A, member 8) |
| 6318 | SERPINB4 | serpin peptidase inhibitor, clade B (ovalbumin), member 4 |
| 8434 | RECK | reversion-inducing-cysteine-rich protein with kazal motifs |
| 12 | SERPINA3 | serpin peptidase inhibitor, clade A (alpha-1 antiproteinase, antitrypsin), member 3 |
| 8710 | SERPINB7 | serpin peptidase inhibitor, clade B (ovalbumin), member 7 |
| 5054 | SERPINE1 | serpin peptidase inhibitor, clade E (nexin, plasminogen activator inhibitor type 1), member 1 |
| 6317 | SERPINB3 | serpin peptidase inhibitor, clade B (ovalbumin), member 3 |
| 5272 | SERPINB9 | serpin peptidase inhibitor, clade B (ovalbumin), member 9 |
| 6590 | SLPI | secretory leukocyte peptidase inhibitor |

  
  
**MSCs batch 3 repressed and**GO:0007167**: 19 genes, expected 5.039, P=8.56711e-07, P adjusted = 0.0997315

|  |  |  |
| --- | --- | --- |
| 2247 | FGF2 | fibroblast growth factor 2 (basic) |
| 4919 | ROR1 | receptor tyrosine kinase-like orphan receptor 1 |
| 1490 | CTGF | connective tissue growth factor |
| 652 | BMP4 | bone morphogenetic protein 4 |
| 4015 | LOX | lysyl oxidase |
| 183 | AGT | angiotensinogen (serpin peptidase inhibitor, clade A, member 8) |
| 10468 | FST | follistatin |
| 1278 | COL1A2 | collagen, type I, alpha 2 |
| 9315 | C5orf13 | chromosome 5 open reading frame 13 |
| 64750 | SMURF2 | SMAD specific E3 ubiquitin protein ligase 2 |
| 7049 | TGFBR3 | transforming growth factor, beta receptor III |
| 1281 | COL3A1 | collagen, type III, alpha 1 |
| 6678 | SPARC | secreted protein, acidic, cysteine-rich (osteonectin) |
| 5792 | PTPRF | protein tyrosine phosphatase, receptor type, F |
| 4803 | NGF | nerve growth factor (beta polypeptide) |
| 2252 | FGF7 | fibroblast growth factor 7 (keratinocyte growth factor) |
| 2263 | FGFR2 | fibroblast growth factor receptor 2 |
| 7046 | TGFBR1 | transforming growth factor, beta receptor 1 |
| 2331 | FMOD | fibromodulin |

  
  
**MSCs batch 3 repressed and**GO:0005604**: 8 genes, expected 0.779846, P=9.90032e-07, P adjusted = 0.115252

|  |  |  |
| --- | --- | --- |
| 4811 | NID1 | nidogen 1 |
| 1284 | COL4A2 | collagen, type IV, alpha 2 |
| 1282 | COL4A1 | collagen, type IV, alpha 1 |
| 2200 | FBN1 | fibrillin 1 |
| 176 | ACAN | aggrecan |
| 3339 | HSPG2 | heparan sulfate proteoglycan 2 |
| 6678 | SPARC | secreted protein, acidic, cysteine-rich (osteonectin) |
| 3918 | LAMC2 | laminin, gamma 2 |

  
  
**MSCs batch 3 repressed and**GO:0031589**: 10 genes, expected 1.36473, P=1.09171e-06, P adjusted = 0.127088

|  |  |  |
| --- | --- | --- |
| 4811 | NID1 | nidogen 1 |
| 1490 | CTGF | connective tissue growth factor |
| 10894 | LYVE1 | lymphatic vessel endothelial hyaluronan receptor 1 |
| 9358 | ITGBL1 | integrin, beta-like 1 (with EGF-like repeat domains) |
| 1842 | ECM2 | extracellular matrix protein 2, female organ and adipocyte specific |
| 183 | AGT | angiotensinogen (serpin peptidase inhibitor, clade A, member 8) |
| 10516 | FBLN5 | fibulin 5 |
| 1281 | COL3A1 | collagen, type III, alpha 1 |
| 3696 | ITGB8 | integrin, beta 8 |
| 22801 | ITGA11 | integrin, alpha 11 |

  
  
**MSCs batch 3 repressed and**GO:0004866**: 12 genes, expected 2.06959, P=1.17812e-06, P adjusted = 0.137148

|  |  |  |
| --- | --- | --- |
| 7057 | THBS1 | thrombospondin 1 |
| 51232 | CRIM1 | cysteine rich transmembrane BMP regulator 1 (chordin-like) |
| 183 | AGT | angiotensinogen (serpin peptidase inhibitor, clade A, member 8) |
| 6318 | SERPINB4 | serpin peptidase inhibitor, clade B (ovalbumin), member 4 |
| 8434 | RECK | reversion-inducing-cysteine-rich protein with kazal motifs |
| 12 | SERPINA3 | serpin peptidase inhibitor, clade A (alpha-1 antiproteinase, antitrypsin), member 3 |
| 8710 | SERPINB7 | serpin peptidase inhibitor, clade B (ovalbumin), member 7 |
| 5054 | SERPINE1 | serpin peptidase inhibitor, clade E (nexin, plasminogen activator inhibitor type 1), member 1 |
| 718 | C3 | complement component 3 |
| 6317 | SERPINB3 | serpin peptidase inhibitor, clade B (ovalbumin), member 3 |
| 5272 | SERPINB9 | serpin peptidase inhibitor, clade B (ovalbumin), member 9 |
| 6590 | SLPI | secretory leukocyte peptidase inhibitor |

  
  
**MSCs batch 3 repressed and**GO:0030414**: 12 genes, expected 2.12958, P=1.59648e-06, P adjusted = 0.185849

|  |  |  |
| --- | --- | --- |
| 7057 | THBS1 | thrombospondin 1 |
| 51232 | CRIM1 | cysteine rich transmembrane BMP regulator 1 (chordin-like) |
| 183 | AGT | angiotensinogen (serpin peptidase inhibitor, clade A, member 8) |
| 6318 | SERPINB4 | serpin peptidase inhibitor, clade B (ovalbumin), member 4 |
| 8434 | RECK | reversion-inducing-cysteine-rich protein with kazal motifs |
| 12 | SERPINA3 | serpin peptidase inhibitor, clade A (alpha-1 antiproteinase, antitrypsin), member 3 |
| 8710 | SERPINB7 | serpin peptidase inhibitor, clade B (ovalbumin), member 7 |
| 5054 | SERPINE1 | serpin peptidase inhibitor, clade E (nexin, plasminogen activator inhibitor type 1), member 1 |
| 718 | C3 | complement component 3 |
| 6317 | SERPINB3 | serpin peptidase inhibitor, clade B (ovalbumin), member 3 |
| 5272 | SERPINB9 | serpin peptidase inhibitor, clade B (ovalbumin), member 9 |
| 6590 | SLPI | secretory leukocyte peptidase inhibitor |

  
  
**MSCs batch 3 repressed and**GO:0009887**: 21 genes, expected 6.52371, P=2.87387e-06, P adjusted = 0.334553

|  |  |  |
| --- | --- | --- |
| 2247 | FGF2 | fibroblast growth factor 2 (basic) |
| 1948 | EFNB2 | ephrin-B2 |
| 1284 | COL4A2 | collagen, type IV, alpha 2 |
| 182 | JAG1 | jagged 1 (Alagille syndrome) |
| 1490 | CTGF | connective tissue growth factor |
| 388 | RHOB | ras homolog gene family, member B |
| 1306 | COL15A1 | collagen, type XV, alpha 1 |
| 652 | BMP4 | bone morphogenetic protein 4 |
| 2627 | GATA6 | GATA binding protein 6 |
| 4982 | TNFRSF11B | tumor necrosis factor receptor superfamily, member 11b |
| 183 | AGT | angiotensinogen (serpin peptidase inhibitor, clade A, member 8) |
| 10468 | FST | follistatin |
| 284 | ANGPT1 | angiopoietin 1 |
| 6347 | CCL2 | chemokine (C-C motif) ligand 2 |
| 5054 | SERPINE1 | serpin peptidase inhibitor, clade E (nexin, plasminogen activator inhibitor type 1), member 1 |
| 7049 | TGFBR3 | transforming growth factor, beta receptor III |
| 2273 | FHL1 | four and a half LIM domains 1 |
| 2619 | GAS1 | growth arrest-specific 1 |
| 1634 | DCN | decorin |
| 7046 | TGFBR1 | transforming growth factor, beta receptor 1 |
| 1906 | EDN1 | endothelin 1 |

  
  
**MSCs batch 3 repressed and**GO:0019838**: 9 genes, expected 1.24475, P=4.23805e-06, P adjusted = 0.49336

|  |  |  |
| --- | --- | --- |
| 1490 | CTGF | connective tissue growth factor |
| 8839 | WISP2 | WNT1 inducible signaling pathway protein 2 |
| 51232 | CRIM1 | cysteine rich transmembrane BMP regulator 1 (chordin-like) |
| 1439 | CSF2RB | colony stimulating factor 2 receptor, beta, low-affinity (granulocyte-macrophage) |
| 7049 | TGFBR3 | transforming growth factor, beta receptor III |
| 4856 | NOV | nephroblastoma overexpressed gene |
| 3485 | IGFBP2 | insulin-like growth factor binding protein 2, 36kDa |
| 3575 | IL7R | interleukin 7 receptor |
| 7046 | TGFBR1 | transforming growth factor, beta receptor 1 |

  
  
**MSCs batch 3 repressed and**GO:0004857**: 15 genes, expected 3.68927, P=5.00303e-06, P adjusted = 0.582413

|  |  |  |
| --- | --- | --- |
| 7057 | THBS1 | thrombospondin 1 |
| 5570 | PKIB | protein kinase (cAMP-dependent, catalytic) inhibitor beta |
| 51232 | CRIM1 | cysteine rich transmembrane BMP regulator 1 (chordin-like) |
| 183 | AGT | angiotensinogen (serpin peptidase inhibitor, clade A, member 8) |
| 6318 | SERPINB4 | serpin peptidase inhibitor, clade B (ovalbumin), member 4 |
| 8434 | RECK | reversion-inducing-cysteine-rich protein with kazal motifs |
| 12 | SERPINA3 | serpin peptidase inhibitor, clade A (alpha-1 antiproteinase, antitrypsin), member 3 |
| 8710 | SERPINB7 | serpin peptidase inhibitor, clade B (ovalbumin), member 7 |
| 5054 | SERPINE1 | serpin peptidase inhibitor, clade E (nexin, plasminogen activator inhibitor type 1), member 1 |
| 80183 | C13orf18 | chromosome 13 open reading frame 18 |
| 718 | C3 | complement component 3 |
| 9749 | PHACTR2 | phosphatase and actin regulator 2 |
| 6317 | SERPINB3 | serpin peptidase inhibitor, clade B (ovalbumin), member 3 |
| 5272 | SERPINB9 | serpin peptidase inhibitor, clade B (ovalbumin), member 9 |
| 6590 | SLPI | secretory leukocyte peptidase inhibitor |

  
  
**MSCs batch 3 repressed and**GO:0005540**: 5 genes, expected 0.269947, P=5.35806e-06, P adjusted = 0.623743

|  |  |  |
| --- | --- | --- |
| 1404 | HAPLN1 | hyaluronan and proteoglycan link protein 1 |
| 10894 | LYVE1 | lymphatic vessel endothelial hyaluronan receptor 1 |
| 176 | ACAN | aggrecan |
| 1462 | VCAN | versican |
| 143903 | LAYN | layilin |

  
  
**MSCs batch 3 repressed and**GO:0008544**: 11 genes, expected 2.09958, P=8.78675e-06, P adjusted = 1

|  |  |  |
| --- | --- | --- |
| 182 | JAG1 | jagged 1 (Alagille syndrome) |
| 1490 | CTGF | connective tissue growth factor |
| 10468 | FST | follistatin |
| 1277 | COL1A1 | collagen, type I, alpha 1 |
| 3861 | KRT14 | keratin 14 |
| 1281 | COL3A1 | collagen, type III, alpha 1 |
| 7357 | UGCG | UDP-glucose ceramide glucosyltransferase |
| 2252 | FGF7 | fibroblast growth factor 7 (keratinocyte growth factor) |
| 3918 | LAMC2 | laminin, gamma 2 |
| 1290 | COL5A2 | collagen, type V, alpha 2 |
| 3885 | KRT34 | keratin 34 |

  
  
**MSCs batch 3 repressed and**GO:0005615**: 21 genes, expected 7.0636, P=9.75746e-06, P adjusted = 1

|  |  |  |
| --- | --- | --- |
| 6387 | CXCL12 | chemokine (C-X-C motif) ligand 12 (stromal cell-derived factor 1) |
| 2247 | FGF2 | fibroblast growth factor 2 (basic) |
| 11341 | SCRG1 | scrapie responsive protein 1 |
| 2192 | FBLN1 | fibulin 1 |
| 2200 | FBN1 | fibrillin 1 |
| 652 | BMP4 | bone morphogenetic protein 4 |
| 6424 | SFRP4 | secreted frizzled-related protein 4 |
| 183 | AGT | angiotensinogen (serpin peptidase inhibitor, clade A, member 8) |
| 7412 | VCAM1 | vascular cell adhesion molecule 1 |
| 4653 | MYOC | myocilin, trabecular meshwork inducible glucocorticoid response |
| 55801 | IL26 | interleukin 26 |
| 3929 | LBP | lipopolysaccharide binding protein |
| 10875 | FGL2 | fibrinogen-like 2 |
| 6347 | CCL2 | chemokine (C-C motif) ligand 2 |
| 7049 | TGFBR3 | transforming growth factor, beta receptor III |
| 1116 | CHI3L1 | chitinase 3-like 1 (cartilage glycoprotein-39) |
| 1281 | COL3A1 | collagen, type III, alpha 1 |
| 3485 | IGFBP2 | insulin-like growth factor binding protein 2, 36kDa |
| 6372 | CXCL6 | chemokine (C-X-C motif) ligand 6 (granulocyte chemotactic protein 2) |
| 23213 | SULF1 | sulfatase 1 |
| 1906 | EDN1 | endothelin 1 |

  
  
**MSCs batch 3 repressed and**GO:0048514**: 12 genes, expected 2.57949, P=1.16353e-05, P adjusted = 1

|  |  |  |
| --- | --- | --- |
| 2247 | FGF2 | fibroblast growth factor 2 (basic) |
| 1284 | COL4A2 | collagen, type IV, alpha 2 |
| 182 | JAG1 | jagged 1 (Alagille syndrome) |
| 1490 | CTGF | connective tissue growth factor |
| 388 | RHOB | ras homolog gene family, member B |
| 1306 | COL15A1 | collagen, type XV, alpha 1 |
| 652 | BMP4 | bone morphogenetic protein 4 |
| 183 | AGT | angiotensinogen (serpin peptidase inhibitor, clade A, member 8) |
| 284 | ANGPT1 | angiopoietin 1 |
| 5054 | SERPINE1 | serpin peptidase inhibitor, clade E (nexin, plasminogen activator inhibitor type 1), member 1 |
| 7046 | TGFBR1 | transforming growth factor, beta receptor 1 |
| 1906 | EDN1 | endothelin 1 |

  
  
**MSCs batch 3 repressed and**GO:0001502**: 4 genes, expected 0.164967, P=1.50615e-05, P adjusted = 1

|  |  |  |
| --- | --- | --- |
| 1301 | COL11A1 | collagen, type XI, alpha 1 |
| 1490 | CTGF | connective tissue growth factor |
| 176 | ACAN | aggrecan |
| 4256 | MGP | matrix Gla protein |

  
  
**MSCs batch 3 repressed and**GO:0007398**: 11 genes, expected 2.24955, P=1.69008e-05, P adjusted = 1

|  |  |  |
| --- | --- | --- |
| 182 | JAG1 | jagged 1 (Alagille syndrome) |
| 1490 | CTGF | connective tissue growth factor |
| 10468 | FST | follistatin |
| 1277 | COL1A1 | collagen, type I, alpha 1 |
| 3861 | KRT14 | keratin 14 |
| 1281 | COL3A1 | collagen, type III, alpha 1 |
| 7357 | UGCG | UDP-glucose ceramide glucosyltransferase |
| 2252 | FGF7 | fibroblast growth factor 7 (keratinocyte growth factor) |
| 3918 | LAMC2 | laminin, gamma 2 |
| 1290 | COL5A2 | collagen, type V, alpha 2 |
| 3885 | KRT34 | keratin 34 |

  
  
**MSCs batch 3 repressed and**GO:0007229**: 7 genes, expected 0.839834, P=1.96778e-05, P adjusted = 1

|  |  |  |
| --- | --- | --- |
| 1490 | CTGF | connective tissue growth factor |
| 9358 | ITGBL1 | integrin, beta-like 1 (with EGF-like repeat domains) |
| 80332 | ADAM33 | ADAM metallopeptidase domain 33 |
| 1281 | COL3A1 | collagen, type III, alpha 1 |
| 3696 | ITGB8 | integrin, beta 8 |
| 22801 | ITGA11 | integrin, alpha 11 |
| 4739 | NEDD9 | neural precursor cell expressed, developmentally down-regulated 9 |

  
  
**MSCs batch 3 repressed and**GO:0051216**: 6 genes, expected 0.599881, P=2.7058e-05, P adjusted = 1

|  |  |  |
| --- | --- | --- |
| 1301 | COL11A1 | collagen, type XI, alpha 1 |
| 1490 | CTGF | connective tissue growth factor |
| 652 | BMP4 | bone morphogenetic protein 4 |
| 176 | ACAN | aggrecan |
| 4256 | MGP | matrix Gla protein |
| 1906 | EDN1 | endothelin 1 |

  
  
**MSCs batch 3 repressed and**GO:0005520**: 5 genes, expected 0.374926, P=3.04828e-05, P adjusted = 1

|  |  |  |
| --- | --- | --- |
| 1490 | CTGF | connective tissue growth factor |
| 8839 | WISP2 | WNT1 inducible signaling pathway protein 2 |
| 51232 | CRIM1 | cysteine rich transmembrane BMP regulator 1 (chordin-like) |
| 4856 | NOV | nephroblastoma overexpressed gene |
| 3485 | IGFBP2 | insulin-like growth factor binding protein 2, 36kDa |

  
  
**MSCs batch 3 repressed and**GO:0006928**: 18 genes, expected 6.16378, P=5.34203e-05, P adjusted = 1

|  |  |  |
| --- | --- | --- |
| 182 | JAG1 | jagged 1 (Alagille syndrome) |
| 1490 | CTGF | connective tissue growth factor |
| 10894 | LYVE1 | lymphatic vessel endothelial hyaluronan receptor 1 |
| 183 | AGT | angiotensinogen (serpin peptidase inhibitor, clade A, member 8) |
| 7412 | VCAM1 | vascular cell adhesion molecule 1 |
| 9201 | DCLK1 | doublecortin-like kinase 1 |
| 150 | ADRA2A | adrenergic, alpha-2A-, receptor |
| 5168 | ENPP2 | ectonucleotide pyrophosphatase/phosphodiesterase 2 |
| 6347 | CCL2 | chemokine (C-C motif) ligand 2 |
| 7168 | TPM1 | tropomyosin 1 (alpha) |
| 8613 | PPAP2B | phosphatidic acid phosphatase type 2B |
| 7049 | TGFBR3 | transforming growth factor, beta receptor III |
| 9369 | NRXN3 | neurexin 3 |
| 288 | ANK3 | ankyrin 3, node of Ranvier (ankyrin G) |
| 800 | CALD1 | caldesmon 1 |
| 7046 | TGFBR1 | transforming growth factor, beta receptor 1 |
| 22801 | ITGA11 | integrin, alpha 11 |
| 1906 | EDN1 | endothelin 1 |

  
  
**MSCs batch 3 repressed and**GO:0051674**: 18 genes, expected 6.16378, P=5.34203e-05, P adjusted = 1

|  |  |  |
| --- | --- | --- |
| 182 | JAG1 | jagged 1 (Alagille syndrome) |
| 1490 | CTGF | connective tissue growth factor |
| 10894 | LYVE1 | lymphatic vessel endothelial hyaluronan receptor 1 |
| 183 | AGT | angiotensinogen (serpin peptidase inhibitor, clade A, member 8) |
| 7412 | VCAM1 | vascular cell adhesion molecule 1 |
| 9201 | DCLK1 | doublecortin-like kinase 1 |
| 150 | ADRA2A | adrenergic, alpha-2A-, receptor |
| 5168 | ENPP2 | ectonucleotide pyrophosphatase/phosphodiesterase 2 |
| 6347 | CCL2 | chemokine (C-C motif) ligand 2 |
| 7168 | TPM1 | tropomyosin 1 (alpha) |
| 8613 | PPAP2B | phosphatidic acid phosphatase type 2B |
| 7049 | TGFBR3 | transforming growth factor, beta receptor III |
| 9369 | NRXN3 | neurexin 3 |
| 288 | ANK3 | ankyrin 3, node of Ranvier (ankyrin G) |
| 800 | CALD1 | caldesmon 1 |
| 7046 | TGFBR1 | transforming growth factor, beta receptor 1 |
| 22801 | ITGA11 | integrin, alpha 11 |
| 1906 | EDN1 | endothelin 1 |

  
  
**MSCs batch 3 repressed and**GO:0001525**: 10 genes, expected 2.12958, P=5.76531e-05, P adjusted = 1

|  |  |  |
| --- | --- | --- |
| 2247 | FGF2 | fibroblast growth factor 2 (basic) |
| 1284 | COL4A2 | collagen, type IV, alpha 2 |
| 182 | JAG1 | jagged 1 (Alagille syndrome) |
| 1490 | CTGF | connective tissue growth factor |
| 388 | RHOB | ras homolog gene family, member B |
| 1306 | COL15A1 | collagen, type XV, alpha 1 |
| 652 | BMP4 | bone morphogenetic protein 4 |
| 284 | ANGPT1 | angiopoietin 1 |
| 5054 | SERPINE1 | serpin peptidase inhibitor, clade E (nexin, plasminogen activator inhibitor type 1), member 1 |
| 1906 | EDN1 | endothelin 1 |

  
  
**MSCs batch 3 repressed and**GO:0006959**: 7 genes, expected 1.0048, P=6.41091e-05, P adjusted = 1

|  |  |  |
| --- | --- | --- |
| 629 | CFB | complement factor B |
| 5648 | MASP1 | mannan-binding lectin serine peptidase 1 (C4/C2 activating component of Ra-reactive factor) |
| 6347 | CCL2 | chemokine (C-C motif) ligand 2 |
| 718 | C3 | complement component 3 |
| 716 | C1S | complement component 1, s subcomponent |
| 715 | C1R | complement component 1, r subcomponent |
| 683 | BST1 | bone marrow stromal cell antigen 1 |

  
  
**MSCs batch 3 repressed and**GO:0002526**: 7 genes, expected 1.04979, P=8.50514e-05, P adjusted = 1

|  |  |  |
| --- | --- | --- |
| 629 | CFB | complement factor B |
| 5648 | MASP1 | mannan-binding lectin serine peptidase 1 (C4/C2 activating component of Ra-reactive factor) |
| 12 | SERPINA3 | serpin peptidase inhibitor, clade A (alpha-1 antiproteinase, antitrypsin), member 3 |
| 3929 | LBP | lipopolysaccharide binding protein |
| 718 | C3 | complement component 3 |
| 716 | C1S | complement component 1, s subcomponent |
| 715 | C1R | complement component 1, r subcomponent |

  
  
**MSCs batch 3 induced and**GO:0006950**: 50 genes, expected 26.0057, P=7.02667e-06, P adjusted = 0.817988

|  |  |  |
| --- | --- | --- |
| 26353 | HSPB8 | heat shock 22kDa protein 8 |
| 650 | BMP2 | bone morphogenetic protein 2 |
| 3162 | HMOX1 | heme oxygenase (decycling) 1 |
| 10344 | CCL26 | chemokine (C-C motif) ligand 26 |
| 3303 | HSPA1A | heat shock 70kDa protein 1A |
| 8942 | KYNU | kynureninase (L-kynurenine hydrolase) |
| 5328 | PLAU | plasminogen activator, urokinase |
| 7056 | THBD | thrombomodulin |
| 64764 | CREB3L2 | cAMP responsive element binding protein 3-like 2 |
| 3576 | IL8 | interleukin 8 |
| 54962 | TIPIN | TIMELESS interacting protein |
| 3553 | IL1B | interleukin 1, beta |
| 9616 | RNF7 | ring finger protein 7 |
| 10808 | HSPH1 | heat shock 105kDa/110kDa protein 1 |
| 3310 | HSPA6 | heat shock 70kDa protein 6 (HSP70B') |
| 133396 | IL31RA | interleukin 31 receptor A |
| 3301 | DNAJA1 | DnaJ (Hsp40) homolog, subfamily A, member 1 |
| 140809 | SRXN1 | sulfiredoxin 1 homolog (S. cerevisiae) |
| 2167 | FABP4 | fatty acid binding protein 4, adipocyte |
| 6421 | SFPQ | splicing factor proline/glutamine-rich (polypyrimidine tract binding protein associated) |
| 7980 | TFPI2 | tissue factor pathway inhibitor 2 |
| 10912 | GADD45G | growth arrest and DNA-damage-inducible, gamma |
| 9448 | MAP4K4 | mitogen-activated protein kinase kinase kinase kinase 4 |
| 131566 | DCBLD2 | discoidin, CUB and LCCL domain containing 2 |
| 81035 | COLEC12 | collectin sub-family member 12 |
| 3320 | HSP90AA1 | heat shock protein 90kDa alpha (cytosolic), class A member 1 |
| 2878 | GPX3 | glutathione peroxidase 3 (plasma) |
| 1839 | HBEGF | heparin-binding EGF-like growth factor |
| 5743 | PTGS2 | prostaglandin-endoperoxide synthase 2 (prostaglandin G/H synthase and cyclooxygenase) |
| 84525 | HOPX | HOP homeobox |
| 23645 | PPP1R15A | protein phosphatase 1, regulatory (inhibitor) subunit 15A |
| 3656 | IRAK2 | interleukin-1 receptor-associated kinase 2 |
| 1728 | NQO1 | NAD(P)H dehydrogenase, quinone 1 |
| 8893 | EIF2B5 | eukaryotic translation initiation factor 2B, subunit 5 epsilon, 82kDa |
| 3589 | IL11 | interleukin 11 |
| 4864 | NPC1 | Niemann-Pick disease, type C1 |
| 2069 | EREG | epiregulin |
| 10963 | STIP1 | stress-induced-phosphoprotein 1 |
| 1909 | EDNRA | endothelin receptor type A |
| 2150 | F2RL1 | coagulation factor II (thrombin) receptor-like 1 |
| 3673 | ITGA2 | integrin, alpha 2 (CD49B, alpha 2 subunit of VLA-2 receptor) |
| 1649 | DDIT3 | DNA-damage-inducible transcript 3 |
| 3783 | KCNN4 | potassium intermediate/small conductance calcium-activated channel, subfamily N, member 4 |
| 9577 | BRE | brain and reproductive organ-expressed (TNFRSF1A modulator) |
| 133746 | JMY | junction-mediating and regulatory protein |
| 8111 | GPR68 | G protein-coupled receptor 68 |
| 3337 | DNAJB1 | DnaJ (Hsp40) homolog, subfamily B, member 1 |
| 3106 | HLA-B | major histocompatibility complex, class I, B |
| 11057 | ABHD2 | abhydrolase domain containing 2 |
| 22824 | HSPA4L | heat shock 70kDa protein 4-like |

  
  
**MSCs batch 3 induced and**GO:0006986**: 8 genes, expected 1.12255, P=1.46978e-05, P adjusted = 1

|  |  |  |
| --- | --- | --- |
| 64764 | CREB3L2 | cAMP responsive element binding protein 3-like 2 |
| 10808 | HSPH1 | heat shock 105kDa/110kDa protein 1 |
| 3310 | HSPA6 | heat shock 70kDa protein 6 (HSP70B') |
| 3301 | DNAJA1 | DnaJ (Hsp40) homolog, subfamily A, member 1 |
| 3320 | HSP90AA1 | heat shock protein 90kDa alpha (cytosolic), class A member 1 |
| 1649 | DDIT3 | DNA-damage-inducible transcript 3 |
| 3337 | DNAJB1 | DnaJ (Hsp40) homolog, subfamily B, member 1 |
| 22824 | HSPA4L | heat shock 70kDa protein 4-like |

  
  
**MSCs batch 3 induced and**GO:0051789**: 8 genes, expected 1.12255, P=1.46978e-05, P adjusted = 1

|  |  |  |
| --- | --- | --- |
| 64764 | CREB3L2 | cAMP responsive element binding protein 3-like 2 |
| 10808 | HSPH1 | heat shock 105kDa/110kDa protein 1 |
| 3310 | HSPA6 | heat shock 70kDa protein 6 (HSP70B') |
| 3301 | DNAJA1 | DnaJ (Hsp40) homolog, subfamily A, member 1 |
| 3320 | HSP90AA1 | heat shock protein 90kDa alpha (cytosolic), class A member 1 |
| 1649 | DDIT3 | DNA-damage-inducible transcript 3 |
| 3337 | DNAJB1 | DnaJ (Hsp40) homolog, subfamily B, member 1 |
| 22824 | HSPA4L | heat shock 70kDa protein 4-like |

  
  
**MSCs batch 3 induced and**GO:0042221**: 27 genes, expected 11.9947, P=8.24229e-05, P adjusted = 1

|  |  |  |
| --- | --- | --- |
| 3162 | HMOX1 | heme oxygenase (decycling) 1 |
| 10344 | CCL26 | chemokine (C-C motif) ligand 26 |
| 8942 | KYNU | kynureninase (L-kynurenine hydrolase) |
| 5328 | PLAU | plasminogen activator, urokinase |
| 64764 | CREB3L2 | cAMP responsive element binding protein 3-like 2 |
| 3576 | IL8 | interleukin 8 |
| 3553 | IL1B | interleukin 1, beta |
| 9616 | RNF7 | ring finger protein 7 |
| 10808 | HSPH1 | heat shock 105kDa/110kDa protein 1 |
| 3310 | HSPA6 | heat shock 70kDa protein 6 (HSP70B') |
| 3301 | DNAJA1 | DnaJ (Hsp40) homolog, subfamily A, member 1 |
| 140809 | SRXN1 | sulfiredoxin 1 homolog (S. cerevisiae) |
| 81035 | COLEC12 | collectin sub-family member 12 |
| 3320 | HSP90AA1 | heat shock protein 90kDa alpha (cytosolic), class A member 1 |
| 2878 | GPX3 | glutathione peroxidase 3 (plasma) |
| 10202 | DHRS2 | dehydrogenase/reductase (SDR family) member 2 |
| 5743 | PTGS2 | prostaglandin-endoperoxide synthase 2 (prostaglandin G/H synthase and cyclooxygenase) |
| 4501 | MT1X | metallothionein 1X |
| 5573 | PRKAR1A | protein kinase, cAMP-dependent, regulatory, type I, alpha (tissue specific extinguisher 1) |
| 1728 | NQO1 | NAD(P)H dehydrogenase, quinone 1 |
| 2357 | FPR1 | formyl peptide receptor 1 |
| 8893 | EIF2B5 | eukaryotic translation initiation factor 2B, subunit 5 epsilon, 82kDa |
| 1645 | AKR1C1 | aldo-keto reductase family 1, member C1 (dihydrodiol dehydrogenase 1; 20-alpha (3-alpha)-hydroxysteroid dehydrogenase) |
| 1649 | DDIT3 | DNA-damage-inducible transcript 3 |
| 5898 | RALA | v-ral simian leukemia viral oncogene homolog A (ras related) |
| 3337 | DNAJB1 | DnaJ (Hsp40) homolog, subfamily B, member 1 |
| 22824 | HSPA4L | heat shock 70kDa protein 4-like |

  
  
**MSCs batch 3 induced and**GO:0048731**: 59 genes, expected 35.6721, P=8.66654e-05, P adjusted = 1

|  |  |  |
| --- | --- | --- |
| 650 | BMP2 | bone morphogenetic protein 2 |
| 56172 | ANKH | ankylosis, progressive homolog (mouse) |
| 3162 | HMOX1 | heme oxygenase (decycling) 1 |
| 3400 | ID4 | inhibitor of DNA binding 4, dominant negative helix-loop-helix protein |
| 92737 | DNER | delta/notch-like EGF repeat containing |
| 64399 | HHIP | hedgehog interacting protein |
| 3909 | LAMA3 | laminin, alpha 3 |
| 29116 | MYLIP | myosin regulatory light chain interacting protein |
| 23210 | JMJD6 | jumonji domain containing 6 |
| 4929 | NR4A2 | nuclear receptor subfamily 4, group A, member 2 |
| 10763 | NES | nestin |
| 3576 | IL8 | interleukin 8 |
| 50861 | STMN3 | stathmin-like 3 |
| 25937 | WWTR1 | WW domain containing transcription regulator 1 |
| 6926 | TBX3 | T-box 3 |
| 133396 | IL31RA | interleukin 31 receptor A |
| 1382 | CRABP2 | cellular retinoic acid binding protein 2 |
| 83478 | ARHGAP24 | Rho GTPase activating protein 24 |
| 4884 | NPTX1 | neuronal pentraxin I |
| 8877 | SPHK1 | sphingosine kinase 1 |
| 23184 | MESDC2 | mesoderm development candidate 2 |
| 9118 | INA | internexin neuronal intermediate filament protein, alpha |
| 4860 | NP | nucleoside phosphorylase |
| 30812 | SOX8 | SRY (sex determining region Y)-box 8 |
| 10202 | DHRS2 | dehydrogenase/reductase (SDR family) member 2 |
| 1839 | HBEGF | heparin-binding EGF-like growth factor |
| 94234 | FOXQ1 | forkhead box Q1 |
| 55859 | BEX1 | brain expressed, X-linked 1 |
| 6526 | SLC5A3 | solute carrier family 5 (sodium/myo-inositol cotransporter), member 3 |
| 5228 | PGF | placental growth factor |
| 4207 | MEF2B | myocyte enhancer factor 2B |
| 5743 | PTGS2 | prostaglandin-endoperoxide synthase 2 (prostaglandin G/H synthase and cyclooxygenase) |
| 84525 | HOPX | HOP homeobox |
| 10439 | OLFM1 | olfactomedin 1 |
| 2294 | FOXF1 | forkhead box F1 |
| 1427 | CRYGS | crystallin, gamma S |
| 5573 | PRKAR1A | protein kinase, cAMP-dependent, regulatory, type I, alpha (tissue specific extinguisher 1) |
| 3280 | HES1 | hairy and enhancer of split 1, (Drosophila) |
| 3475 | IFRD1 | interferon-related developmental regulator 1 |
| 6809 | STX3 | syntaxin 3 |
| 10018 | BCL2L11 | BCL2-like 11 (apoptosis facilitator) |
| 56243 | KIAA1217 | KIAA1217 |
| 8893 | EIF2B5 | eukaryotic translation initiation factor 2B, subunit 5 epsilon, 82kDa |
| 10019 | SH2B3 | SH2B adaptor protein 3 |
| 23237 | ARC | activity-regulated cytoskeleton-associated protein |
| 23462 | HEY1 | hairy/enhancer-of-split related with YRPW motif 1 |
| 3589 | IL11 | interleukin 11 |
| 2069 | EREG | epiregulin |
| 3910 | LAMA4 | laminin, alpha 4 |
| 3976 | LIF | leukemia inhibitory factor (cholinergic differentiation factor) |
| 1305 | COL13A1 | collagen, type XIII, alpha 1 |
| 6696 | SPP1 | secreted phosphoprotein 1 |
| 1909 | EDNRA | endothelin receptor type A |
| 3673 | ITGA2 | integrin, alpha 2 (CD49B, alpha 2 subunit of VLA-2 receptor) |
| 2047 | EPHB1 | EPH receptor B1 |
| 4693 | NDP | Norrie disease (pseudoglioma) |
| 10253 | SPRY2 | sprouty homolog 2 (Drosophila) |
| 3897 | L1CAM | L1 cell adhesion molecule |
| 1746 | DLX2 | distal-less homeobox 2 |

  
  
**MSCs batch 3 induced and**GO:0042060**: 11 genes, expected 2.70244, P=8.71762e-05, P adjusted = 1

|  |  |  |
| --- | --- | --- |
| 3162 | HMOX1 | heme oxygenase (decycling) 1 |
| 5328 | PLAU | plasminogen activator, urokinase |
| 7056 | THBD | thrombomodulin |
| 7980 | TFPI2 | tissue factor pathway inhibitor 2 |
| 131566 | DCBLD2 | discoidin, CUB and LCCL domain containing 2 |
| 1839 | HBEGF | heparin-binding EGF-like growth factor |
| 84525 | HOPX | HOP homeobox |
| 3589 | IL11 | interleukin 11 |
| 2069 | EREG | epiregulin |
| 2150 | F2RL1 | coagulation factor II (thrombin) receptor-like 1 |
| 3673 | ITGA2 | integrin, alpha 2 (CD49B, alpha 2 subunit of VLA-2 receptor) |

  
  
**MSCs batch 4 repressed and**GO:0007565**: 6 genes, expected 0.330529, P=9.63851e-07, P adjusted = 0.112204

|  |  |  |
| --- | --- | --- |
| 5676 | PSG7 | pregnancy specific beta-1-glycoprotein 7 |
| 5021 | OXTR | oxytocin receptor |
| 5673 | PSG5 | pregnancy specific beta-1-glycoprotein 5 |
| 5678 | PSG9 | pregnancy specific beta-1-glycoprotein 9 |
| 5675 | PSG6 | pregnancy specific beta-1-glycoprotein 6 |
| 5672 | PSG4 | pregnancy specific beta-1-glycoprotein 4 |

  
  
**MSCs batch 4 repressed and**GO:0005576**: 23 genes, expected 9.14987, P=2.94286e-05, P adjusted = 1

|  |  |  |
| --- | --- | --- |
| 4057 | LTF | lactotransferrin |
| 79875 | THSD4 | thrombospondin, type I, domain containing 4 |
| 1301 | COL11A1 | collagen, type XI, alpha 1 |
| 5676 | PSG7 | pregnancy specific beta-1-glycoprotein 7 |
| 3953 | LEPR | leptin receptor |
| 8076 | MFAP5 | microfibrillar associated protein 5 |
| 5673 | PSG5 | pregnancy specific beta-1-glycoprotein 5 |
| 84171 | LOXL4 | lysyl oxidase-like 4 |
| 8492 | PRSS12 | protease, serine, 12 (neurotrypsin, motopsin) |
| 222663 | SCUBE3 | signal peptide, CUB domain, EGF-like 3 |
| 2252 | FGF7 | fibroblast growth factor 7 (keratinocyte growth factor) |
| 5678 | PSG9 | pregnancy specific beta-1-glycoprotein 9 |
| 4982 | TNFRSF11B | tumor necrosis factor receptor superfamily, member 11b |
| 129804 | FBLN7 | fibulin 7 |
| 5649 | RELN | reelin |
| 3488 | IGFBP5 | insulin-like growth factor binding protein 5 |
| 5675 | PSG6 | pregnancy specific beta-1-glycoprotein 6 |
| 5672 | PSG4 | pregnancy specific beta-1-glycoprotein 4 |
| 3484 | IGFBP1 | insulin-like growth factor binding protein 1 |
| 1303 | COL12A1 | collagen, type XII, alpha 1 |
| 3553 | IL1B | interleukin 1, beta |
| 147372 | CCBE1 | collagen and calcium binding EGF domains 1 |
| 84623 | KIRREL3 | kin of IRRE like 3 (Drosophila) |

  
  
**MSCs batch 4 induced and**GO:0005576**: 71 genes, expected 22.2704, P=3.9445e-19, P adjusted = 4.59187e-14

|  |  |  |
| --- | --- | --- |
| 8840 | WISP1 | WNT1 inducible signaling pathway protein 1 |
| 6387 | CXCL12 | chemokine (C-X-C motif) ligand 12 (stromal cell-derived factor 1) |
| 80144 | FRAS1 | Fraser syndrome 1 |
| 9241 | NOG | noggin |
| 55118 | CRTAC1 | cartilage acidic protein 1 |
| 5730 | PTGDS | prostaglandin D2 synthase 21kDa (brain) |
| 4147 | MATN2 | matrilin 2 |
| 11341 | SCRG1 | scrapie responsive protein 1 |
| 5122 | PCSK1 | proprotein convertase subtilisin/kexin type 1 |
| 2201 | FBN2 | fibrillin 2 |
| 5655 | KLK10 | kallikrein-related peptidase 10 |
| 3383 | ICAM1 | intercellular adhesion molecule 1 |
| 7425 | VGF | VGF nerve growth factor inducible |
| 1191 | CLU | clusterin |
| 652 | BMP4 | bone morphogenetic protein 4 |
| 157869 | RPESP | RPE-spondin |
| 2487 | FRZB | frizzled-related protein |
| 3671 | ISLR | immunoglobulin superfamily containing leucine-rich repeat |
| 64399 | HHIP | hedgehog interacting protein |
| 50509 | COL5A3 | collagen, type V, alpha 3 |
| 2 | A2M | alpha-2-macroglobulin |
| 8788 | DLK1 | delta-like 1 homolog (Drosophila) |
| 22854 | NTNG1 | netrin G1 |
| 64856 | VWA1 | von Willebrand factor A domain containing 1 |
| 3481 | IGF2 | insulin-like growth factor 2 (somatomedin A) |
| 55801 | IL26 | interleukin 26 |
| 55959 | SULF2 | sulfatase 2 |
| 89932 | PAPLN | papilin, proteoglycan-like sulfated glycoprotein |
| 256691 | MAMDC2 | MAM domain containing 2 |
| 9244 | CRLF1 | cytokine receptor-like factor 1 |
| 348 | APOE | apolipoprotein E |
| 5157 | PDGFRL | platelet-derived growth factor receptor-like |
| 3589 | IL11 | interleukin 11 |
| 1299 | COL9A3 | collagen, type IX, alpha 3 |
| 10417 | SPON2 | spondin 2, extracellular matrix protein |
| 8532 | CPZ | carboxypeptidase Z |
| 6347 | CCL2 | chemokine (C-C motif) ligand 2 |
| 2069 | EREG | epiregulin |
| 1469 | CST1 | cystatin SN |
| 5046 | PCSK6 | proprotein convertase subtilisin/kexin type 6 |
| 1314 | COPA | coatomer protein complex, subunit alpha |
| 4879 | NPPB | natriuretic peptide precursor B |
| 3977 | LIFR | leukemia inhibitory factor receptor alpha |
| 54360 | CYTL1 | cytokine-like 1 |
| 23671 | TMEFF2 | transmembrane protein with EGF-like and two follistatin-like domains 2 |
| 28514 | DLL1 | delta-like 1 (Drosophila) |
| 9547 | CXCL14 | chemokine (C-X-C motif) ligand 14 |
| 374946 | C1orf187 | chromosome 1 open reading frame 187 |
| 1116 | CHI3L1 | chitinase 3-like 1 (cartilage glycoprotein-39) |
| 3911 | LAMA5 | laminin, alpha 5 |
| 4885 | NPTX2 | neuronal pentraxin II |
| 718 | C3 | complement component 3 |
| 58189 | WFDC1 | WAP four-disulfide core domain 1 |
| 50964 | SOST | sclerosteosis |
| 9806 | SPOCK2 | sparc/osteonectin, cwcv and kazal-like domains proteoglycan (testican) 2 |
| 2263 | FGFR2 | fibroblast growth factor receptor 2 |
| 84966 | IGSF21 | immunoglobin superfamily, member 21 |
| 1081 | CGA | glycoprotein hormones, alpha polypeptide |
| 2690 | GHR | growth hormone receptor |
| 90139 | TSPAN18 | tetraspanin 18 |
| 57124 | CD248 | CD248 molecule, endosialin |
| 11009 | IL24 | interleukin 24 |
| 3955 | LFNG | LFNG O-fucosylpeptide 3-beta-N-acetylglucosaminyltransferase |
| 1525 | CXADR | coxsackie virus and adenovirus receptor |
| 9388 | LIPG | lipase, endothelial |
| 1277 | COL1A1 | collagen, type I, alpha 1 |
| 1311 | COMP | cartilage oligomeric matrix protein |
| 23767 | FLRT3 | fibronectin leucine rich transmembrane protein 3 |
| 3730 | KAL1 | Kallmann syndrome 1 sequence |
| 2042 | EPHA3 | EPH receptor A3 |
| 80310 | PDGFD | platelet derived growth factor D |

  
  
**MSCs batch 4 induced and**GO:0044421**: 42 genes, expected 9.43684, P=3.40484e-16, P adjusted = 3.96364e-11

|  |  |  |
| --- | --- | --- |
| 6387 | CXCL12 | chemokine (C-X-C motif) ligand 12 (stromal cell-derived factor 1) |
| 80144 | FRAS1 | Fraser syndrome 1 |
| 9241 | NOG | noggin |
| 4147 | MATN2 | matrilin 2 |
| 11341 | SCRG1 | scrapie responsive protein 1 |
| 5122 | PCSK1 | proprotein convertase subtilisin/kexin type 1 |
| 2201 | FBN2 | fibrillin 2 |
| 3383 | ICAM1 | intercellular adhesion molecule 1 |
| 7425 | VGF | VGF nerve growth factor inducible |
| 1191 | CLU | clusterin |
| 652 | BMP4 | bone morphogenetic protein 4 |
| 50509 | COL5A3 | collagen, type V, alpha 3 |
| 8788 | DLK1 | delta-like 1 homolog (Drosophila) |
| 22854 | NTNG1 | netrin G1 |
| 64856 | VWA1 | von Willebrand factor A domain containing 1 |
| 55801 | IL26 | interleukin 26 |
| 55959 | SULF2 | sulfatase 2 |
| 89932 | PAPLN | papilin, proteoglycan-like sulfated glycoprotein |
| 9244 | CRLF1 | cytokine receptor-like factor 1 |
| 348 | APOE | apolipoprotein E |
| 3589 | IL11 | interleukin 11 |
| 1299 | COL9A3 | collagen, type IX, alpha 3 |
| 10417 | SPON2 | spondin 2, extracellular matrix protein |
| 8532 | CPZ | carboxypeptidase Z |
| 6347 | CCL2 | chemokine (C-C motif) ligand 2 |
| 2069 | EREG | epiregulin |
| 5046 | PCSK6 | proprotein convertase subtilisin/kexin type 6 |
| 1314 | COPA | coatomer protein complex, subunit alpha |
| 4879 | NPPB | natriuretic peptide precursor B |
| 54360 | CYTL1 | cytokine-like 1 |
| 9547 | CXCL14 | chemokine (C-X-C motif) ligand 14 |
| 1116 | CHI3L1 | chitinase 3-like 1 (cartilage glycoprotein-39) |
| 3911 | LAMA5 | laminin, alpha 5 |
| 58189 | WFDC1 | WAP four-disulfide core domain 1 |
| 9806 | SPOCK2 | sparc/osteonectin, cwcv and kazal-like domains proteoglycan (testican) 2 |
| 90139 | TSPAN18 | tetraspanin 18 |
| 57124 | CD248 | CD248 molecule, endosialin |
| 11009 | IL24 | interleukin 24 |
| 1277 | COL1A1 | collagen, type I, alpha 1 |
| 1311 | COMP | cartilage oligomeric matrix protein |
| 23767 | FLRT3 | fibronectin leucine rich transmembrane protein 3 |
| 3730 | KAL1 | Kallmann syndrome 1 sequence |

  
  
**MSCs batch 4 induced and**GO:0048731**: 61 genes, expected 21.9129, P=1.16613e-13, P adjusted = 1.35752e-08

|  |  |  |
| --- | --- | --- |
| 84525 | HOPX | HOP homeobox |
| 3785 | KCNQ2 | potassium voltage-gated channel, KQT-like subfamily, member 2 |
| 10439 | OLFM1 | olfactomedin 1 |
| 9241 | NOG | noggin |
| 1949 | EFNB3 | ephrin-B3 |
| 4804 | NGFR | nerve growth factor receptor (TNFR superfamily, member 16) |
| 3280 | HES1 | hairy and enhancer of split 1, (Drosophila) |
| 11341 | SCRG1 | scrapie responsive protein 1 |
| 1397 | CRIP2 | cysteine-rich protein 2 |
| 26508 | HEYL | hairy/enhancer-of-split related with YRPW motif-like |
| 7425 | VGF | VGF nerve growth factor inducible |
| 2824 | GPM6B | glycoprotein M6B |
| 652 | BMP4 | bone morphogenetic protein 4 |
| 6599 | SMARCC1 | SWI/SNF related, matrix associated, actin dependent regulator of chromatin, subfamily c, member 1 |
| 92737 | DNER | delta/notch-like EGF repeat containing |
| 4761 | NEUROD2 | neurogenic differentiation 2 |
| 2487 | FRZB | frizzled-related protein |
| 7852 | CXCR4 | chemokine (C-X-C motif) receptor 4 |
| 64399 | HHIP | hedgehog interacting protein |
| 50509 | COL5A3 | collagen, type V, alpha 3 |
| 22854 | NTNG1 | netrin G1 |
| 3481 | IGF2 | insulin-like growth factor 2 (somatomedin A) |
| 2304 | FOXE1 | forkhead box E1 (thyroid transcription factor 2) |
| 10763 | NES | nestin |
| 348 | APOE | apolipoprotein E |
| 1134 | CHRNA1 | cholinergic receptor, nicotinic, alpha 1 (muscle) |
| 3589 | IL11 | interleukin 11 |
| 50861 | STMN3 | stathmin-like 3 |
| 10417 | SPON2 | spondin 2, extracellular matrix protein |
| 9355 | LHX2 | LIM homeobox 2 |
| 596 | BCL2 | B-cell CLL/lymphoma 2 |
| 6347 | CCL2 | chemokine (C-C motif) ligand 2 |
| 6840 | SVIL | supervillin |
| 2069 | EREG | epiregulin |
| 5076 | PAX2 | paired box 2 |
| 1382 | CRABP2 | cellular retinoic acid binding protein 2 |
| 4879 | NPPB | natriuretic peptide precursor B |
| 5979 | RET | ret proto-oncogene |
| 2261 | FGFR3 | fibroblast growth factor receptor 3 |
| 54360 | CYTL1 | cytokine-like 1 |
| 5493 | PPL | periplakin |
| 28514 | DLL1 | delta-like 1 (Drosophila) |
| 7098 | TLR3 | toll-like receptor 3 |
| 3911 | LAMA5 | laminin, alpha 5 |
| 6664 | SOX11 | SRY (sex determining region Y)-box 11 |
| 6591 | SNAI2 | snail homolog 2 (Drosophila) |
| 2047 | EPHB1 | EPH receptor B1 |
| 50964 | SOST | sclerosteosis |
| 9806 | SPOCK2 | sparc/osteonectin, cwcv and kazal-like domains proteoglycan (testican) 2 |
| 30812 | SOX8 | SRY (sex determining region Y)-box 8 |
| 2690 | GHR | growth hormone receptor |
| 3955 | LFNG | LFNG O-fucosylpeptide 3-beta-N-acetylglucosaminyltransferase |
| 3800 | KIF5C | kinesin family member 5C |
| 1277 | COL1A1 | collagen, type I, alpha 1 |
| 1311 | COMP | cartilage oligomeric matrix protein |
| 80303 | EFHD1 | EF-hand domain family, member D1 |
| 3730 | KAL1 | Kallmann syndrome 1 sequence |
| 3897 | L1CAM | L1 cell adhesion molecule |
| 972 | CD74 | CD74 molecule, major histocompatibility complex, class II invariant chain |
| 4744 | NEFH | neurofilament, heavy polypeptide |
| 1400 | CRMP1 | collapsin response mediator protein 1 |

  
  
**MSCs batch 4 induced and**GO:0031226**: 47 genes, expected 14.6469, P=1.00692e-12, P adjusted = 1.17217e-07

|  |  |  |
| --- | --- | --- |
| 2150 | F2RL1 | coagulation factor II (thrombin) receptor-like 1 |
| 3785 | KCNQ2 | potassium voltage-gated channel, KQT-like subfamily, member 2 |
| 9289 | GPR56 | G protein-coupled receptor 56 |
| 1949 | EFNB3 | ephrin-B3 |
| 4804 | NGFR | nerve growth factor receptor (TNFR superfamily, member 16) |
| 6326 | SCN2A | sodium channel, voltage-gated, type II, alpha subunit |
| 3655 | ITGA6 | integrin, alpha 6 |
| 3383 | ICAM1 | intercellular adhesion molecule 1 |
| 2043 | EPHA4 | EPH receptor A4 |
| 7852 | CXCR4 | chemokine (C-X-C motif) receptor 4 |
| 6533 | SLC6A6 | solute carrier family 6 (neurotransmitter transporter, taurine), member 6 |
| 64399 | HHIP | hedgehog interacting protein |
| 6330 | SCN4B | sodium channel, voltage-gated, type IV, beta |
| 22854 | NTNG1 | netrin G1 |
| 358 | AQP1 | aquaporin 1 (Colton blood group) |
| 6572 | SLC18A3 | solute carrier family 18 (vesicular acetylcholine), member 3 |
| 1134 | CHRNA1 | cholinergic receptor, nicotinic, alpha 1 (muscle) |
| 3759 | KCNJ2 | potassium inwardly-rectifying channel, subfamily J, member 2 |
| 2069 | EREG | epiregulin |
| 3752 | KCND3 | potassium voltage-gated channel, Shal-related subfamily, member 3 |
| 3977 | LIFR | leukemia inhibitory factor receptor alpha |
| 2261 | FGFR3 | fibroblast growth factor receptor 3 |
| 28514 | DLL1 | delta-like 1 (Drosophila) |
| 9427 | ECEL1 | endothelin converting enzyme-like 1 |
| 7098 | TLR3 | toll-like receptor 3 |
| 1942 | EFNA1 | ephrin-A1 |
| 2047 | EPHB1 | EPH receptor B1 |
| 3122 | HLA-DRA | major histocompatibility complex, class II, DR alpha |
| 5733 | PTGER3 | prostaglandin E receptor 3 (subtype EP3) |
| 131566 | DCBLD2 | discoidin, CUB and LCCL domain containing 2 |
| 5099 | PCDH7 | protocadherin 7 |
| 50632 | CALY | calcyon neuron-specific vesicular protein |
| 10723 | SLC12A7 | solute carrier family 12 (potassium/chloride transporters), member 7 |
| 23531 | MMD | monocyte to macrophage differentiation-associated |
| 6529 | SLC6A1 | solute carrier family 6 (neurotransmitter transporter, GABA), member 1 |
| 7433 | VIPR1 | vasoactive intestinal peptide receptor 1 |
| 3680 | ITGA9 | integrin, alpha 9 |
| 2690 | GHR | growth hormone receptor |
| 5789 | PTPRD | protein tyrosine phosphatase, receptor type, D |
| 1903 | S1PR3 | sphingosine-1-phosphate receptor 3 |
| 3113 | HLA-DPA1 | major histocompatibility complex, class II, DP alpha 1 |
| 9568 | GABBR2 | gamma-aminobutyric acid (GABA) B receptor, 2 |
| 55890 | GPRC5C | G protein-coupled receptor, family C, group 5, member C |
| 1525 | CXADR | coxsackie virus and adenovirus receptor |
| 23767 | FLRT3 | fibronectin leucine rich transmembrane protein 3 |
| 2042 | EPHA3 | EPH receptor A3 |
| 7805 | LAPTM5 | lysosomal multispanning membrane protein 5 |

  
  
**MSCs batch 4 induced and**GO:0005887**: 46 genes, expected 14.4681, P=2.52228e-12, P adjusted = 2.93623e-07

|  |  |  |
| --- | --- | --- |
| 2150 | F2RL1 | coagulation factor II (thrombin) receptor-like 1 |
| 3785 | KCNQ2 | potassium voltage-gated channel, KQT-like subfamily, member 2 |
| 9289 | GPR56 | G protein-coupled receptor 56 |
| 1949 | EFNB3 | ephrin-B3 |
| 4804 | NGFR | nerve growth factor receptor (TNFR superfamily, member 16) |
| 6326 | SCN2A | sodium channel, voltage-gated, type II, alpha subunit |
| 3655 | ITGA6 | integrin, alpha 6 |
| 3383 | ICAM1 | intercellular adhesion molecule 1 |
| 2043 | EPHA4 | EPH receptor A4 |
| 7852 | CXCR4 | chemokine (C-X-C motif) receptor 4 |
| 6533 | SLC6A6 | solute carrier family 6 (neurotransmitter transporter, taurine), member 6 |
| 64399 | HHIP | hedgehog interacting protein |
| 6330 | SCN4B | sodium channel, voltage-gated, type IV, beta |
| 358 | AQP1 | aquaporin 1 (Colton blood group) |
| 6572 | SLC18A3 | solute carrier family 18 (vesicular acetylcholine), member 3 |
| 1134 | CHRNA1 | cholinergic receptor, nicotinic, alpha 1 (muscle) |
| 3759 | KCNJ2 | potassium inwardly-rectifying channel, subfamily J, member 2 |
| 2069 | EREG | epiregulin |
| 3752 | KCND3 | potassium voltage-gated channel, Shal-related subfamily, member 3 |
| 3977 | LIFR | leukemia inhibitory factor receptor alpha |
| 2261 | FGFR3 | fibroblast growth factor receptor 3 |
| 28514 | DLL1 | delta-like 1 (Drosophila) |
| 9427 | ECEL1 | endothelin converting enzyme-like 1 |
| 7098 | TLR3 | toll-like receptor 3 |
| 1942 | EFNA1 | ephrin-A1 |
| 2047 | EPHB1 | EPH receptor B1 |
| 3122 | HLA-DRA | major histocompatibility complex, class II, DR alpha |
| 5733 | PTGER3 | prostaglandin E receptor 3 (subtype EP3) |
| 131566 | DCBLD2 | discoidin, CUB and LCCL domain containing 2 |
| 5099 | PCDH7 | protocadherin 7 |
| 50632 | CALY | calcyon neuron-specific vesicular protein |
| 10723 | SLC12A7 | solute carrier family 12 (potassium/chloride transporters), member 7 |
| 23531 | MMD | monocyte to macrophage differentiation-associated |
| 6529 | SLC6A1 | solute carrier family 6 (neurotransmitter transporter, GABA), member 1 |
| 7433 | VIPR1 | vasoactive intestinal peptide receptor 1 |
| 3680 | ITGA9 | integrin, alpha 9 |
| 2690 | GHR | growth hormone receptor |
| 5789 | PTPRD | protein tyrosine phosphatase, receptor type, D |
| 1903 | S1PR3 | sphingosine-1-phosphate receptor 3 |
| 3113 | HLA-DPA1 | major histocompatibility complex, class II, DP alpha 1 |
| 9568 | GABBR2 | gamma-aminobutyric acid (GABA) B receptor, 2 |
| 55890 | GPRC5C | G protein-coupled receptor, family C, group 5, member C |
| 1525 | CXADR | coxsackie virus and adenovirus receptor |
| 23767 | FLRT3 | fibronectin leucine rich transmembrane protein 3 |
| 2042 | EPHA3 | EPH receptor A3 |
| 7805 | LAPTM5 | lysosomal multispanning membrane protein 5 |

  
  
**MSCs batch 4 induced and**GO:0044459**: 57 genes, expected 22.3343, P=3.01106e-11, P adjusted = 3.50523e-06

|  |  |  |
| --- | --- | --- |
| 2150 | F2RL1 | coagulation factor II (thrombin) receptor-like 1 |
| 3785 | KCNQ2 | potassium voltage-gated channel, KQT-like subfamily, member 2 |
| 9289 | GPR56 | G protein-coupled receptor 56 |
| 1949 | EFNB3 | ephrin-B3 |
| 4804 | NGFR | nerve growth factor receptor (TNFR superfamily, member 16) |
| 6326 | SCN2A | sodium channel, voltage-gated, type II, alpha subunit |
| 3655 | ITGA6 | integrin, alpha 6 |
| 3383 | ICAM1 | intercellular adhesion molecule 1 |
| 2043 | EPHA4 | EPH receptor A4 |
| 7852 | CXCR4 | chemokine (C-X-C motif) receptor 4 |
| 6533 | SLC6A6 | solute carrier family 6 (neurotransmitter transporter, taurine), member 6 |
| 64399 | HHIP | hedgehog interacting protein |
| 360 | AQP3 | aquaporin 3 (Gill blood group) |
| 6330 | SCN4B | sodium channel, voltage-gated, type IV, beta |
| 22854 | NTNG1 | netrin G1 |
| 358 | AQP1 | aquaporin 1 (Colton blood group) |
| 6572 | SLC18A3 | solute carrier family 18 (vesicular acetylcholine), member 3 |
| 1134 | CHRNA1 | cholinergic receptor, nicotinic, alpha 1 (muscle) |
| 3759 | KCNJ2 | potassium inwardly-rectifying channel, subfamily J, member 2 |
| 2069 | EREG | epiregulin |
| 3123 | HLA-DRB1 | major histocompatibility complex, class II, DR beta 1 |
| 3752 | KCND3 | potassium voltage-gated channel, Shal-related subfamily, member 3 |
| 3977 | LIFR | leukemia inhibitory factor receptor alpha |
| 2261 | FGFR3 | fibroblast growth factor receptor 3 |
| 3728 | JUP | junction plakoglobin |
| 5493 | PPL | periplakin |
| 28514 | DLL1 | delta-like 1 (Drosophila) |
| 9427 | ECEL1 | endothelin converting enzyme-like 1 |
| 7098 | TLR3 | toll-like receptor 3 |
| 3119 | HLA-DQB1 | major histocompatibility complex, class II, DQ beta 1 |
| 1942 | EFNA1 | ephrin-A1 |
| 2047 | EPHB1 | EPH receptor B1 |
| 3122 | HLA-DRA | major histocompatibility complex, class II, DR alpha |
| 5733 | PTGER3 | prostaglandin E receptor 3 (subtype EP3) |
| 131566 | DCBLD2 | discoidin, CUB and LCCL domain containing 2 |
| 5099 | PCDH7 | protocadherin 7 |
| 50632 | CALY | calcyon neuron-specific vesicular protein |
| 10723 | SLC12A7 | solute carrier family 12 (potassium/chloride transporters), member 7 |
| 23531 | MMD | monocyte to macrophage differentiation-associated |
| 6529 | SLC6A1 | solute carrier family 6 (neurotransmitter transporter, GABA), member 1 |
| 7433 | VIPR1 | vasoactive intestinal peptide receptor 1 |
| 3680 | ITGA9 | integrin, alpha 9 |
| 2690 | GHR | growth hormone receptor |
| 5789 | PTPRD | protein tyrosine phosphatase, receptor type, D |
| 23504 | RIMBP2 | RIMS binding protein 2 |
| 8913 | CACNA1G | calcium channel, voltage-dependent, T type, alpha 1G subunit |
| 1903 | S1PR3 | sphingosine-1-phosphate receptor 3 |
| 3113 | HLA-DPA1 | major histocompatibility complex, class II, DP alpha 1 |
| 9568 | GABBR2 | gamma-aminobutyric acid (GABA) B receptor, 2 |
| 55890 | GPRC5C | G protein-coupled receptor, family C, group 5, member C |
| 1525 | CXADR | coxsackie virus and adenovirus receptor |
| 9783 | RIMS3 | regulating synaptic membrane exocytosis 3 |
| 9368 | SLC9A3R1 | solute carrier family 9 (sodium/hydrogen exchanger), member 3 regulator 1 |
| 23767 | FLRT3 | fibronectin leucine rich transmembrane protein 3 |
| 2042 | EPHA3 | EPH receptor A3 |
| 58480 | RHOU | ras homolog gene family, member U |
| 7805 | LAPTM5 | lysosomal multispanning membrane protein 5 |

  
  
**MSCs batch 4 induced and**GO:0007166**: 49 genes, expected 18.1841, P=1.57437e-10, P adjusted = 1.83275e-05

|  |  |  |
| --- | --- | --- |
| 2150 | F2RL1 | coagulation factor II (thrombin) receptor-like 1 |
| 8840 | WISP1 | WNT1 inducible signaling pathway protein 1 |
| 6387 | CXCL12 | chemokine (C-X-C motif) ligand 12 (stromal cell-derived factor 1) |
| 9241 | NOG | noggin |
| 9289 | GPR56 | G protein-coupled receptor 56 |
| 1949 | EFNB3 | ephrin-B3 |
| 23220 | DTX4 | deltex 4 homolog (Drosophila) |
| 3655 | ITGA6 | integrin, alpha 6 |
| 26508 | HEYL | hairy/enhancer-of-split related with YRPW motif-like |
| 57007 | CXCR7 | chemokine (C-X-C motif) receptor 7 |
| 652 | BMP4 | bone morphogenetic protein 4 |
| 2043 | EPHA4 | EPH receptor A4 |
| 27065 | D4S234E | DNA segment on chromosome 4 (unique) 234 expressed sequence |
| 92737 | DNER | delta/notch-like EGF repeat containing |
| 2487 | FRZB | frizzled-related protein |
| 7852 | CXCR4 | chemokine (C-X-C motif) receptor 4 |
| 57537 | SORCS2 | sortilin-related VPS10 domain containing receptor 2 |
| 64399 | HHIP | hedgehog interacting protein |
| 3481 | IGF2 | insulin-like growth factor 2 (somatomedin A) |
| 8651 | SOCS1 | suppressor of cytokine signaling 1 |
| 348 | APOE | apolipoprotein E |
| 3875 | KRT18 | keratin 18 |
| 8532 | CPZ | carboxypeptidase Z |
| 6347 | CCL2 | chemokine (C-C motif) ligand 2 |
| 2069 | EREG | epiregulin |
| 51655 | RASD1 | RAS, dexamethasone-induced 1 |
| 5046 | PCSK6 | proprotein convertase subtilisin/kexin type 6 |
| 1028 | CDKN1C | cyclin-dependent kinase inhibitor 1C (p57, Kip2) |
| 4879 | NPPB | natriuretic peptide precursor B |
| 3977 | LIFR | leukemia inhibitory factor receptor alpha |
| 2261 | FGFR3 | fibroblast growth factor receptor 3 |
| 28514 | DLL1 | delta-like 1 (Drosophila) |
| 9427 | ECEL1 | endothelin converting enzyme-like 1 |
| 2047 | EPHB1 | EPH receptor B1 |
| 718 | C3 | complement component 3 |
| 5733 | PTGER3 | prostaglandin E receptor 3 (subtype EP3) |
| 84634 | KISS1R | KISS1 receptor |
| 50964 | SOST | sclerosteosis |
| 50632 | CALY | calcyon neuron-specific vesicular protein |
| 2263 | FGFR2 | fibroblast growth factor receptor 2 |
| 7433 | VIPR1 | vasoactive intestinal peptide receptor 1 |
| 3680 | ITGA9 | integrin, alpha 9 |
| 9021 | SOCS3 | suppressor of cytokine signaling 3 |
| 5789 | PTPRD | protein tyrosine phosphatase, receptor type, D |
| 1903 | S1PR3 | sphingosine-1-phosphate receptor 3 |
| 9568 | GABBR2 | gamma-aminobutyric acid (GABA) B receptor, 2 |
| 55890 | GPRC5C | G protein-coupled receptor, family C, group 5, member C |
| 9368 | SLC9A3R1 | solute carrier family 9 (sodium/hydrogen exchanger), member 3 regulator 1 |
| 2042 | EPHA3 | EPH receptor A3 |

  
  
**MSCs batch 4 induced and**GO:0007399**: 33 genes, expected 9.25807, P=2.53531e-10, P adjusted = 2.9514e-05

|  |  |  |
| --- | --- | --- |
| 3785 | KCNQ2 | potassium voltage-gated channel, KQT-like subfamily, member 2 |
| 10439 | OLFM1 | olfactomedin 1 |
| 9241 | NOG | noggin |
| 1949 | EFNB3 | ephrin-B3 |
| 4804 | NGFR | nerve growth factor receptor (TNFR superfamily, member 16) |
| 3280 | HES1 | hairy and enhancer of split 1, (Drosophila) |
| 11341 | SCRG1 | scrapie responsive protein 1 |
| 26508 | HEYL | hairy/enhancer-of-split related with YRPW motif-like |
| 2824 | GPM6B | glycoprotein M6B |
| 652 | BMP4 | bone morphogenetic protein 4 |
| 92737 | DNER | delta/notch-like EGF repeat containing |
| 4761 | NEUROD2 | neurogenic differentiation 2 |
| 7852 | CXCR4 | chemokine (C-X-C motif) receptor 4 |
| 64399 | HHIP | hedgehog interacting protein |
| 22854 | NTNG1 | netrin G1 |
| 10763 | NES | nestin |
| 348 | APOE | apolipoprotein E |
| 50861 | STMN3 | stathmin-like 3 |
| 10417 | SPON2 | spondin 2, extracellular matrix protein |
| 9355 | LHX2 | LIM homeobox 2 |
| 5076 | PAX2 | paired box 2 |
| 5979 | RET | ret proto-oncogene |
| 2261 | FGFR3 | fibroblast growth factor receptor 3 |
| 6664 | SOX11 | SRY (sex determining region Y)-box 11 |
| 2047 | EPHB1 | EPH receptor B1 |
| 9806 | SPOCK2 | sparc/osteonectin, cwcv and kazal-like domains proteoglycan (testican) 2 |
| 30812 | SOX8 | SRY (sex determining region Y)-box 8 |
| 3800 | KIF5C | kinesin family member 5C |
| 80303 | EFHD1 | EF-hand domain family, member D1 |
| 3730 | KAL1 | Kallmann syndrome 1 sequence |
| 3897 | L1CAM | L1 cell adhesion molecule |
| 4744 | NEFH | neurofilament, heavy polypeptide |
| 1400 | CRMP1 | collapsin response mediator protein 1 |

  
  
**MSCs batch 4 induced and**GO:0005615**: 26 genes, expected 6.01455, P=4.19841e-10, P adjusted = 4.88745e-05

|  |  |  |
| --- | --- | --- |
| 6387 | CXCL12 | chemokine (C-X-C motif) ligand 12 (stromal cell-derived factor 1) |
| 9241 | NOG | noggin |
| 11341 | SCRG1 | scrapie responsive protein 1 |
| 5122 | PCSK1 | proprotein convertase subtilisin/kexin type 1 |
| 3383 | ICAM1 | intercellular adhesion molecule 1 |
| 7425 | VGF | VGF nerve growth factor inducible |
| 1191 | CLU | clusterin |
| 652 | BMP4 | bone morphogenetic protein 4 |
| 8788 | DLK1 | delta-like 1 homolog (Drosophila) |
| 55801 | IL26 | interleukin 26 |
| 55959 | SULF2 | sulfatase 2 |
| 9244 | CRLF1 | cytokine receptor-like factor 1 |
| 348 | APOE | apolipoprotein E |
| 3589 | IL11 | interleukin 11 |
| 6347 | CCL2 | chemokine (C-C motif) ligand 2 |
| 2069 | EREG | epiregulin |
| 5046 | PCSK6 | proprotein convertase subtilisin/kexin type 6 |
| 1314 | COPA | coatomer protein complex, subunit alpha |
| 4879 | NPPB | natriuretic peptide precursor B |
| 54360 | CYTL1 | cytokine-like 1 |
| 9547 | CXCL14 | chemokine (C-X-C motif) ligand 14 |
| 1116 | CHI3L1 | chitinase 3-like 1 (cartilage glycoprotein-39) |
| 58189 | WFDC1 | WAP four-disulfide core domain 1 |
| 90139 | TSPAN18 | tetraspanin 18 |
| 11009 | IL24 | interleukin 24 |
| 3730 | KAL1 | Kallmann syndrome 1 sequence |

  
  
**MSCs batch 4 induced and**GO:0031012**: 20 genes, expected 3.84369, P=2.10197e-09, P adjusted = 0.000244695

|  |  |  |
| --- | --- | --- |
| 80144 | FRAS1 | Fraser syndrome 1 |
| 4147 | MATN2 | matrilin 2 |
| 2201 | FBN2 | fibrillin 2 |
| 652 | BMP4 | bone morphogenetic protein 4 |
| 50509 | COL5A3 | collagen, type V, alpha 3 |
| 22854 | NTNG1 | netrin G1 |
| 64856 | VWA1 | von Willebrand factor A domain containing 1 |
| 89932 | PAPLN | papilin, proteoglycan-like sulfated glycoprotein |
| 1299 | COL9A3 | collagen, type IX, alpha 3 |
| 10417 | SPON2 | spondin 2, extracellular matrix protein |
| 8532 | CPZ | carboxypeptidase Z |
| 5046 | PCSK6 | proprotein convertase subtilisin/kexin type 6 |
| 1116 | CHI3L1 | chitinase 3-like 1 (cartilage glycoprotein-39) |
| 3911 | LAMA5 | laminin, alpha 5 |
| 9806 | SPOCK2 | sparc/osteonectin, cwcv and kazal-like domains proteoglycan (testican) 2 |
| 57124 | CD248 | CD248 molecule, endosialin |
| 1277 | COL1A1 | collagen, type I, alpha 1 |
| 1311 | COMP | cartilage oligomeric matrix protein |
| 23767 | FLRT3 | fibronectin leucine rich transmembrane protein 3 |
| 3730 | KAL1 | Kallmann syndrome 1 sequence |

  
  
**MSCs batch 4 induced and**GO:0005578**: 19 genes, expected 3.67769, P=6.04837e-09, P adjusted = 0.000704103

|  |  |  |
| --- | --- | --- |
| 80144 | FRAS1 | Fraser syndrome 1 |
| 4147 | MATN2 | matrilin 2 |
| 2201 | FBN2 | fibrillin 2 |
| 652 | BMP4 | bone morphogenetic protein 4 |
| 50509 | COL5A3 | collagen, type V, alpha 3 |
| 22854 | NTNG1 | netrin G1 |
| 64856 | VWA1 | von Willebrand factor A domain containing 1 |
| 89932 | PAPLN | papilin, proteoglycan-like sulfated glycoprotein |
| 1299 | COL9A3 | collagen, type IX, alpha 3 |
| 10417 | SPON2 | spondin 2, extracellular matrix protein |
| 8532 | CPZ | carboxypeptidase Z |
| 1116 | CHI3L1 | chitinase 3-like 1 (cartilage glycoprotein-39) |
| 3911 | LAMA5 | laminin, alpha 5 |
| 9806 | SPOCK2 | sparc/osteonectin, cwcv and kazal-like domains proteoglycan (testican) 2 |
| 57124 | CD248 | CD248 molecule, endosialin |
| 1277 | COL1A1 | collagen, type I, alpha 1 |
| 1311 | COMP | cartilage oligomeric matrix protein |
| 23767 | FLRT3 | fibronectin leucine rich transmembrane protein 3 |
| 3730 | KAL1 | Kallmann syndrome 1 sequence |

  
  
**MSCs batch 4 induced and**GO:0005509**: 33 genes, expected 11.2885, P=3.40011e-08, P adjusted = 0.00395813

|  |  |  |
| --- | --- | --- |
| 11240 | PADI2 | peptidyl arginine deiminase, type II |
| 80144 | FRAS1 | Fraser syndrome 1 |
| 57685 | CACHD1 | cache domain containing 1 |
| 55118 | CRTAC1 | cartilage acidic protein 1 |
| 1013 | CDH15 | cadherin 15, M-cadherin (myotubule) |
| 4147 | MATN2 | matrilin 2 |
| 5122 | PCSK1 | proprotein convertase subtilisin/kexin type 1 |
| 2201 | FBN2 | fibrillin 2 |
| 3655 | ITGA6 | integrin, alpha 6 |
| 92737 | DNER | delta/notch-like EGF repeat containing |
| 5101 | PCDH9 | protocadherin 9 |
| 55033 | FKBP14 | FK506 binding protein 14, 22 kDa |
| 9254 | CACNA2D2 | calcium channel, voltage-dependent, alpha 2/delta subunit 2 |
| 8788 | DLK1 | delta-like 1 homolog (Drosophila) |
| 55959 | SULF2 | sulfatase 2 |
| 1008 | CDH10 | cadherin 10, type 2 (T2-cadherin) |
| 6840 | SVIL | supervillin |
| 5046 | PCSK6 | proprotein convertase subtilisin/kexin type 6 |
| 94015 | TTYH2 | tweety homolog 2 (Drosophila) |
| 1314 | COPA | coatomer protein complex, subunit alpha |
| 5979 | RET | ret proto-oncogene |
| 28514 | DLL1 | delta-like 1 (Drosophila) |
| 4885 | NPTX2 | neuronal pentraxin II |
| 164633 | CABP7 | calcium binding protein 7 |
| 1953 | MEGF6 | multiple EGF-like-domains 6 |
| 81035 | COLEC12 | collectin sub-family member 12 |
| 5099 | PCDH7 | protocadherin 7 |
| 9806 | SPOCK2 | sparc/osteonectin, cwcv and kazal-like domains proteoglycan (testican) 2 |
| 3680 | ITGA9 | integrin, alpha 9 |
| 57124 | CD248 | CD248 molecule, endosialin |
| 8913 | CACNA1G | calcium channel, voltage-dependent, T type, alpha 1G subunit |
| 1311 | COMP | cartilage oligomeric matrix protein |
| 80303 | EFHD1 | EF-hand domain family, member D1 |

  
  
**MSCs batch 4 induced and**GO:0007155**: 29 genes, expected 9.20699, P=5.12269e-08, P adjusted = 0.00596342

|  |  |  |
| --- | --- | --- |
| 8840 | WISP1 | WNT1 inducible signaling pathway protein 1 |
| 6387 | CXCL12 | chemokine (C-X-C motif) ligand 12 (stromal cell-derived factor 1) |
| 9241 | NOG | noggin |
| 9289 | GPR56 | G protein-coupled receptor 56 |
| 1013 | CDH15 | cadherin 15, M-cadherin (myotubule) |
| 3280 | HES1 | hairy and enhancer of split 1, (Drosophila) |
| 4162 | MCAM | melanoma cell adhesion molecule |
| 3655 | ITGA6 | integrin, alpha 6 |
| 3383 | ICAM1 | intercellular adhesion molecule 1 |
| 5101 | PCDH9 | protocadherin 9 |
| 3671 | ISLR | immunoglobulin superfamily containing leucine-rich repeat |
| 50509 | COL5A3 | collagen, type V, alpha 3 |
| 199731 | CADM4 | cell adhesion molecule 4 |
| 10154 | PLXNC1 | plexin C1 |
| 10417 | SPON2 | spondin 2, extracellular matrix protein |
| 6347 | CCL2 | chemokine (C-C motif) ligand 2 |
| 1008 | CDH10 | cadherin 10, type 2 (T2-cadherin) |
| 5979 | RET | ret proto-oncogene |
| 3728 | JUP | junction plakoglobin |
| 28514 | DLL1 | delta-like 1 (Drosophila) |
| 3911 | LAMA5 | laminin, alpha 5 |
| 131566 | DCBLD2 | discoidin, CUB and LCCL domain containing 2 |
| 5099 | PCDH7 | protocadherin 7 |
| 3680 | ITGA9 | integrin, alpha 9 |
| 1525 | CXADR | coxsackie virus and adenovirus receptor |
| 1311 | COMP | cartilage oligomeric matrix protein |
| 23767 | FLRT3 | fibronectin leucine rich transmembrane protein 3 |
| 3730 | KAL1 | Kallmann syndrome 1 sequence |
| 3897 | L1CAM | L1 cell adhesion molecule |

  
  
**MSCs batch 4 induced and**GO:0022610**: 29 genes, expected 9.20699, P=5.12269e-08, P adjusted = 0.00596342

|  |  |  |
| --- | --- | --- |
| 8840 | WISP1 | WNT1 inducible signaling pathway protein 1 |
| 6387 | CXCL12 | chemokine (C-X-C motif) ligand 12 (stromal cell-derived factor 1) |
| 9241 | NOG | noggin |
| 9289 | GPR56 | G protein-coupled receptor 56 |
| 1013 | CDH15 | cadherin 15, M-cadherin (myotubule) |
| 3280 | HES1 | hairy and enhancer of split 1, (Drosophila) |
| 4162 | MCAM | melanoma cell adhesion molecule |
| 3655 | ITGA6 | integrin, alpha 6 |
| 3383 | ICAM1 | intercellular adhesion molecule 1 |
| 5101 | PCDH9 | protocadherin 9 |
| 3671 | ISLR | immunoglobulin superfamily containing leucine-rich repeat |
| 50509 | COL5A3 | collagen, type V, alpha 3 |
| 199731 | CADM4 | cell adhesion molecule 4 |
| 10154 | PLXNC1 | plexin C1 |
| 10417 | SPON2 | spondin 2, extracellular matrix protein |
| 6347 | CCL2 | chemokine (C-C motif) ligand 2 |
| 1008 | CDH10 | cadherin 10, type 2 (T2-cadherin) |
| 5979 | RET | ret proto-oncogene |
| 3728 | JUP | junction plakoglobin |
| 28514 | DLL1 | delta-like 1 (Drosophila) |
| 3911 | LAMA5 | laminin, alpha 5 |
| 131566 | DCBLD2 | discoidin, CUB and LCCL domain containing 2 |
| 5099 | PCDH7 | protocadherin 7 |
| 3680 | ITGA9 | integrin, alpha 9 |
| 1525 | CXADR | coxsackie virus and adenovirus receptor |
| 1311 | COMP | cartilage oligomeric matrix protein |
| 23767 | FLRT3 | fibronectin leucine rich transmembrane protein 3 |
| 3730 | KAL1 | Kallmann syndrome 1 sequence |
| 3897 | L1CAM | L1 cell adhesion molecule |

  
  
**MSCs batch 4 induced and**GO:0005102**: 29 genes, expected 9.539, P=1.089e-07, P adjusted = 0.0126773

|  |  |  |
| --- | --- | --- |
| 2150 | F2RL1 | coagulation factor II (thrombin) receptor-like 1 |
| 6387 | CXCL12 | chemokine (C-X-C motif) ligand 12 (stromal cell-derived factor 1) |
| 1949 | EFNB3 | ephrin-B3 |
| 7425 | VGF | VGF nerve growth factor inducible |
| 652 | BMP4 | bone morphogenetic protein 4 |
| 27065 | D4S234E | DNA segment on chromosome 4 (unique) 234 expressed sequence |
| 92737 | DNER | delta/notch-like EGF repeat containing |
| 3481 | IGF2 | insulin-like growth factor 2 (somatomedin A) |
| 8651 | SOCS1 | suppressor of cytokine signaling 1 |
| 55801 | IL26 | interleukin 26 |
| 10154 | PLXNC1 | plexin C1 |
| 348 | APOE | apolipoprotein E |
| 3589 | IL11 | interleukin 11 |
| 6347 | CCL2 | chemokine (C-C motif) ligand 2 |
| 2069 | EREG | epiregulin |
| 1314 | COPA | coatomer protein complex, subunit alpha |
| 4879 | NPPB | natriuretic peptide precursor B |
| 54360 | CYTL1 | cytokine-like 1 |
| 28514 | DLL1 | delta-like 1 (Drosophila) |
| 9547 | CXCL14 | chemokine (C-X-C motif) ligand 14 |
| 3911 | LAMA5 | laminin, alpha 5 |
| 1942 | EFNA1 | ephrin-A1 |
| 718 | C3 | complement component 3 |
| 50632 | CALY | calcyon neuron-specific vesicular protein |
| 1081 | CGA | glycoprotein hormones, alpha polypeptide |
| 11009 | IL24 | interleukin 24 |
| 9368 | SLC9A3R1 | solute carrier family 9 (sodium/hydrogen exchanger), member 3 regulator 1 |
| 972 | CD74 | CD74 molecule, major histocompatibility complex, class II invariant chain |
| 80310 | PDGFD | platelet derived growth factor D |

  
  
**MSCs batch 4 induced and**GO:0048699**: 17 genes, expected 3.53722, P=1.11154e-07, P adjusted = 0.0129397

|  |  |  |
| --- | --- | --- |
| 9241 | NOG | noggin |
| 1949 | EFNB3 | ephrin-B3 |
| 4804 | NGFR | nerve growth factor receptor (TNFR superfamily, member 16) |
| 3280 | HES1 | hairy and enhancer of split 1, (Drosophila) |
| 92737 | DNER | delta/notch-like EGF repeat containing |
| 4761 | NEUROD2 | neurogenic differentiation 2 |
| 7852 | CXCR4 | chemokine (C-X-C motif) receptor 4 |
| 64399 | HHIP | hedgehog interacting protein |
| 22854 | NTNG1 | netrin G1 |
| 348 | APOE | apolipoprotein E |
| 50861 | STMN3 | stathmin-like 3 |
| 10417 | SPON2 | spondin 2, extracellular matrix protein |
| 5076 | PAX2 | paired box 2 |
| 5979 | RET | ret proto-oncogene |
| 3800 | KIF5C | kinesin family member 5C |
| 80303 | EFHD1 | EF-hand domain family, member D1 |
| 3730 | KAL1 | Kallmann syndrome 1 sequence |

  
  
**MSCs batch 4 induced and**GO:0048468**: 22 genes, expected 6.19333, P=3.11133e-07, P adjusted = 0.0362196

|  |  |  |
| --- | --- | --- |
| 9241 | NOG | noggin |
| 1949 | EFNB3 | ephrin-B3 |
| 4804 | NGFR | nerve growth factor receptor (TNFR superfamily, member 16) |
| 3280 | HES1 | hairy and enhancer of split 1, (Drosophila) |
| 652 | BMP4 | bone morphogenetic protein 4 |
| 92737 | DNER | delta/notch-like EGF repeat containing |
| 4761 | NEUROD2 | neurogenic differentiation 2 |
| 7852 | CXCR4 | chemokine (C-X-C motif) receptor 4 |
| 64399 | HHIP | hedgehog interacting protein |
| 22854 | NTNG1 | netrin G1 |
| 348 | APOE | apolipoprotein E |
| 1134 | CHRNA1 | cholinergic receptor, nicotinic, alpha 1 (muscle) |
| 50861 | STMN3 | stathmin-like 3 |
| 10417 | SPON2 | spondin 2, extracellular matrix protein |
| 2069 | EREG | epiregulin |
| 5076 | PAX2 | paired box 2 |
| 5979 | RET | ret proto-oncogene |
| 3911 | LAMA5 | laminin, alpha 5 |
| 30812 | SOX8 | SRY (sex determining region Y)-box 8 |
| 3800 | KIF5C | kinesin family member 5C |
| 80303 | EFHD1 | EF-hand domain family, member D1 |
| 3730 | KAL1 | Kallmann syndrome 1 sequence |

  
  
**MSCs batch 4 induced and**GO:0022008**: 17 genes, expected 3.80539, P=3.1443e-07, P adjusted = 0.0366034

|  |  |  |
| --- | --- | --- |
| 9241 | NOG | noggin |
| 1949 | EFNB3 | ephrin-B3 |
| 4804 | NGFR | nerve growth factor receptor (TNFR superfamily, member 16) |
| 3280 | HES1 | hairy and enhancer of split 1, (Drosophila) |
| 92737 | DNER | delta/notch-like EGF repeat containing |
| 4761 | NEUROD2 | neurogenic differentiation 2 |
| 7852 | CXCR4 | chemokine (C-X-C motif) receptor 4 |
| 64399 | HHIP | hedgehog interacting protein |
| 22854 | NTNG1 | netrin G1 |
| 348 | APOE | apolipoprotein E |
| 50861 | STMN3 | stathmin-like 3 |
| 10417 | SPON2 | spondin 2, extracellular matrix protein |
| 5076 | PAX2 | paired box 2 |
| 5979 | RET | ret proto-oncogene |
| 3800 | KIF5C | kinesin family member 5C |
| 80303 | EFHD1 | EF-hand domain family, member D1 |
| 3730 | KAL1 | Kallmann syndrome 1 sequence |

  
  
**MSCs batch 4 induced and**GO:0048513**: 39 genes, expected 16.358, P=3.85273e-07, P adjusted = 0.0448505

|  |  |  |
| --- | --- | --- |
| 84525 | HOPX | HOP homeobox |
| 9241 | NOG | noggin |
| 4804 | NGFR | nerve growth factor receptor (TNFR superfamily, member 16) |
| 3280 | HES1 | hairy and enhancer of split 1, (Drosophila) |
| 1397 | CRIP2 | cysteine-rich protein 2 |
| 7425 | VGF | VGF nerve growth factor inducible |
| 652 | BMP4 | bone morphogenetic protein 4 |
| 6599 | SMARCC1 | SWI/SNF related, matrix associated, actin dependent regulator of chromatin, subfamily c, member 1 |
| 92737 | DNER | delta/notch-like EGF repeat containing |
| 2487 | FRZB | frizzled-related protein |
| 7852 | CXCR4 | chemokine (C-X-C motif) receptor 4 |
| 64399 | HHIP | hedgehog interacting protein |
| 50509 | COL5A3 | collagen, type V, alpha 3 |
| 3481 | IGF2 | insulin-like growth factor 2 (somatomedin A) |
| 2304 | FOXE1 | forkhead box E1 (thyroid transcription factor 2) |
| 1134 | CHRNA1 | cholinergic receptor, nicotinic, alpha 1 (muscle) |
| 3589 | IL11 | interleukin 11 |
| 9355 | LHX2 | LIM homeobox 2 |
| 596 | BCL2 | B-cell CLL/lymphoma 2 |
| 6347 | CCL2 | chemokine (C-C motif) ligand 2 |
| 6840 | SVIL | supervillin |
| 2069 | EREG | epiregulin |
| 5076 | PAX2 | paired box 2 |
| 1382 | CRABP2 | cellular retinoic acid binding protein 2 |
| 4879 | NPPB | natriuretic peptide precursor B |
| 5979 | RET | ret proto-oncogene |
| 2261 | FGFR3 | fibroblast growth factor receptor 3 |
| 54360 | CYTL1 | cytokine-like 1 |
| 5493 | PPL | periplakin |
| 28514 | DLL1 | delta-like 1 (Drosophila) |
| 7098 | TLR3 | toll-like receptor 3 |
| 3911 | LAMA5 | laminin, alpha 5 |
| 6591 | SNAI2 | snail homolog 2 (Drosophila) |
| 50964 | SOST | sclerosteosis |
| 2690 | GHR | growth hormone receptor |
| 3955 | LFNG | LFNG O-fucosylpeptide 3-beta-N-acetylglucosaminyltransferase |
| 1277 | COL1A1 | collagen, type I, alpha 1 |
| 1311 | COMP | cartilage oligomeric matrix protein |
| 972 | CD74 | CD74 molecule, major histocompatibility complex, class II invariant chain |

  
  
**MSCs batch 4 induced and**GO:0004872**: 40 genes, expected 17.0732, P=4.19603e-07, P adjusted = 0.0488468

|  |  |  |
| --- | --- | --- |
| 2150 | F2RL1 | coagulation factor II (thrombin) receptor-like 1 |
| 79971 | GPR177 | G protein-coupled receptor 177 |
| 9289 | GPR56 | G protein-coupled receptor 56 |
| 1949 | EFNB3 | ephrin-B3 |
| 4804 | NGFR | nerve growth factor receptor (TNFR superfamily, member 16) |
| 3655 | ITGA6 | integrin, alpha 6 |
| 3383 | ICAM1 | intercellular adhesion molecule 1 |
| 57007 | CXCR7 | chemokine (C-X-C motif) receptor 7 |
| 2043 | EPHA4 | EPH receptor A4 |
| 92737 | DNER | delta/notch-like EGF repeat containing |
| 7852 | CXCR4 | chemokine (C-X-C motif) receptor 4 |
| 57537 | SORCS2 | sortilin-related VPS10 domain containing receptor 2 |
| 10154 | PLXNC1 | plexin C1 |
| 9244 | CRLF1 | cytokine receptor-like factor 1 |
| 90249 | UNC5A | unc-5 homolog A (C. elegans) |
| 1134 | CHRNA1 | cholinergic receptor, nicotinic, alpha 1 (muscle) |
| 5157 | PDGFRL | platelet-derived growth factor receptor-like |
| 3977 | LIFR | leukemia inhibitory factor receptor alpha |
| 5979 | RET | ret proto-oncogene |
| 2261 | FGFR3 | fibroblast growth factor receptor 3 |
| 7098 | TLR3 | toll-like receptor 3 |
| 3911 | LAMA5 | laminin, alpha 5 |
| 3119 | HLA-DQB1 | major histocompatibility complex, class II, DQ beta 1 |
| 2047 | EPHB1 | EPH receptor B1 |
| 3122 | HLA-DRA | major histocompatibility complex, class II, DR alpha |
| 5733 | PTGER3 | prostaglandin E receptor 3 (subtype EP3) |
| 84634 | KISS1R | KISS1 receptor |
| 81035 | COLEC12 | collectin sub-family member 12 |
| 2263 | FGFR2 | fibroblast growth factor receptor 2 |
| 23531 | MMD | monocyte to macrophage differentiation-associated |
| 7433 | VIPR1 | vasoactive intestinal peptide receptor 1 |
| 3680 | ITGA9 | integrin, alpha 9 |
| 2690 | GHR | growth hormone receptor |
| 5789 | PTPRD | protein tyrosine phosphatase, receptor type, D |
| 1903 | S1PR3 | sphingosine-1-phosphate receptor 3 |
| 3113 | HLA-DPA1 | major histocompatibility complex, class II, DP alpha 1 |
| 9568 | GABBR2 | gamma-aminobutyric acid (GABA) B receptor, 2 |
| 55890 | GPRC5C | G protein-coupled receptor, family C, group 5, member C |
| 1525 | CXADR | coxsackie virus and adenovirus receptor |
| 2042 | EPHA3 | EPH receptor A3 |

  
  
**MSCs batch 4 induced and**GO:0004888**: 29 genes, expected 10.4457, P=7.13354e-07, P adjusted = 0.083043

|  |  |  |
| --- | --- | --- |
| 9289 | GPR56 | G protein-coupled receptor 56 |
| 1949 | EFNB3 | ephrin-B3 |
| 4804 | NGFR | nerve growth factor receptor (TNFR superfamily, member 16) |
| 3383 | ICAM1 | intercellular adhesion molecule 1 |
| 2043 | EPHA4 | EPH receptor A4 |
| 92737 | DNER | delta/notch-like EGF repeat containing |
| 7852 | CXCR4 | chemokine (C-X-C motif) receptor 4 |
| 57537 | SORCS2 | sortilin-related VPS10 domain containing receptor 2 |
| 1134 | CHRNA1 | cholinergic receptor, nicotinic, alpha 1 (muscle) |
| 5157 | PDGFRL | platelet-derived growth factor receptor-like |
| 3977 | LIFR | leukemia inhibitory factor receptor alpha |
| 5979 | RET | ret proto-oncogene |
| 2261 | FGFR3 | fibroblast growth factor receptor 3 |
| 7098 | TLR3 | toll-like receptor 3 |
| 3119 | HLA-DQB1 | major histocompatibility complex, class II, DQ beta 1 |
| 2047 | EPHB1 | EPH receptor B1 |
| 3122 | HLA-DRA | major histocompatibility complex, class II, DR alpha |
| 5733 | PTGER3 | prostaglandin E receptor 3 (subtype EP3) |
| 84634 | KISS1R | KISS1 receptor |
| 81035 | COLEC12 | collectin sub-family member 12 |
| 2263 | FGFR2 | fibroblast growth factor receptor 2 |
| 7433 | VIPR1 | vasoactive intestinal peptide receptor 1 |
| 2690 | GHR | growth hormone receptor |
| 5789 | PTPRD | protein tyrosine phosphatase, receptor type, D |
| 1903 | S1PR3 | sphingosine-1-phosphate receptor 3 |
| 3113 | HLA-DPA1 | major histocompatibility complex, class II, DP alpha 1 |
| 9568 | GABBR2 | gamma-aminobutyric acid (GABA) B receptor, 2 |
| 55890 | GPRC5C | G protein-coupled receptor, family C, group 5, member C |
| 2042 | EPHA3 | EPH receptor A3 |

  
  
**MSCs batch 4 induced and**GO:0048523**: 36 genes, expected 14.9023, P=8.41039e-07, P adjusted = 0.097907

|  |  |  |
| --- | --- | --- |
| 84525 | HOPX | HOP homeobox |
| 9241 | NOG | noggin |
| 1917 | EEF1A2 | eukaryotic translation elongation factor 1 alpha 2 |
| 3280 | HES1 | hairy and enhancer of split 1, (Drosophila) |
| 5655 | KLK10 | kallikrein-related peptidase 10 |
| 652 | BMP4 | bone morphogenetic protein 4 |
| 2487 | FRZB | frizzled-related protein |
| 9774 | BCLAF1 | BCL2-associated transcription factor 1 |
| 64399 | HHIP | hedgehog interacting protein |
| 199731 | CADM4 | cell adhesion molecule 4 |
| 10643 | IGF2BP3 | insulin-like growth factor 2 mRNA binding protein 3 |
| 8651 | SOCS1 | suppressor of cytokine signaling 1 |
| 2304 | FOXE1 | forkhead box E1 (thyroid transcription factor 2) |
| 348 | APOE | apolipoprotein E |
| 3589 | IL11 | interleukin 11 |
| 50861 | STMN3 | stathmin-like 3 |
| 3875 | KRT18 | keratin 18 |
| 596 | BCL2 | B-cell CLL/lymphoma 2 |
| 6347 | CCL2 | chemokine (C-C motif) ligand 2 |
| 6840 | SVIL | supervillin |
| 2069 | EREG | epiregulin |
| 5046 | PCSK6 | proprotein convertase subtilisin/kexin type 6 |
| 5076 | PAX2 | paired box 2 |
| 1028 | CDKN1C | cyclin-dependent kinase inhibitor 1C (p57, Kip2) |
| 4879 | NPPB | natriuretic peptide precursor B |
| 2261 | FGFR3 | fibroblast growth factor receptor 3 |
| 28514 | DLL1 | delta-like 1 (Drosophila) |
| 7098 | TLR3 | toll-like receptor 3 |
| 6591 | SNAI2 | snail homolog 2 (Drosophila) |
| 84634 | KISS1R | KISS1 receptor |
| 58189 | WFDC1 | WAP four-disulfide core domain 1 |
| 50964 | SOST | sclerosteosis |
| 131566 | DCBLD2 | discoidin, CUB and LCCL domain containing 2 |
| 5296 | PIK3R2 | phosphoinositide-3-kinase, regulatory subunit 2 (beta) |
| 9021 | SOCS3 | suppressor of cytokine signaling 3 |
| 972 | CD74 | CD74 molecule, major histocompatibility complex, class II invariant chain |

  
  
**MSCs batch 4 induced and**GO:0030154**: 36 genes, expected 14.9406, P=8.93187e-07, P adjusted = 0.103978

|  |  |  |
| --- | --- | --- |
| 84525 | HOPX | HOP homeobox |
| 9241 | NOG | noggin |
| 1949 | EFNB3 | ephrin-B3 |
| 4804 | NGFR | nerve growth factor receptor (TNFR superfamily, member 16) |
| 3280 | HES1 | hairy and enhancer of split 1, (Drosophila) |
| 2824 | GPM6B | glycoprotein M6B |
| 652 | BMP4 | bone morphogenetic protein 4 |
| 92737 | DNER | delta/notch-like EGF repeat containing |
| 4761 | NEUROD2 | neurogenic differentiation 2 |
| 2487 | FRZB | frizzled-related protein |
| 7852 | CXCR4 | chemokine (C-X-C motif) receptor 4 |
| 64399 | HHIP | hedgehog interacting protein |
| 360 | AQP3 | aquaporin 3 (Gill blood group) |
| 22854 | NTNG1 | netrin G1 |
| 8651 | SOCS1 | suppressor of cytokine signaling 1 |
| 348 | APOE | apolipoprotein E |
| 1134 | CHRNA1 | cholinergic receptor, nicotinic, alpha 1 (muscle) |
| 3589 | IL11 | interleukin 11 |
| 50861 | STMN3 | stathmin-like 3 |
| 10417 | SPON2 | spondin 2, extracellular matrix protein |
[truncated: 46,453 more chars]
